# Interplay between ligand field strength and the nephelauxetic effect in chromium(iii) complexes with anionic amido ligands

**DOI:** 10.1039/d5sc09069e

**Published:** 2026-02-19

**Authors:** P. Yaltseva, B. Wittwer, D. Leitner, F. R. Neururer, F. Tambornino, A. Schmidt, D. Munz, O. S. Wenger, S. Hohloch

**Affiliations:** a Department of Chemistry, University of Basel St. Johanns-Ring 19 4056 Basel Switzerland oliver.wenger@unibas.ch; b Department of General, Inorganic and Theoretical Chemistry, University of Innsbruck Innrain 80–82 6020 Innsbruck Austria Stephan.Hohloch@uibk.ac.at; c Department of Chemistry, Philipps-Universität Marburg Hans-Meerwein-Strasse 4 35032 Marburg Germany; d Coordination Chemistry, Saarland University, Campus C4.1 66123 Saarbrücken Germany dominik.munz@uni-saarland.de

## Abstract

Incorporation of the nephelauxetic effect into ligand design enabled red-shifting of spin-flip transitions of Cr^III^ and Mn^IV^ complexes into the near-infrared region. Using carbazolide complexes as a model, we present a strategy for tuning the ratio of ligand field strength to the Racah parameter *B* by combining a covalent carbazolide core with variable σ-donor ligand “side arms.” Substitution of pyridine, as in [Cr(L^py^)_2_]^+^ ([L^py^]^−^ = 3,6-di-*tert*-butyl-1,8-di(pyridin-2-yl)carbazol-9-ide), with stronger σ-donors such as *N*-heterocyclic or mesoionic carbenes in [Cr(L^NHC^)_2_]^+^ or [Cr(L^MIC^)_2_]^+^ ([L^NHC^]^−^ = 3,6-di-*tert*-butyl-1,8-bis(imidazolin-2-yliden-1-yl)carbazolide and [L^MIC^]^−^ = 3,6-di-*tert*-butyl-1,8-bis(4,5,6,7-tetrahydro-2*H*-[1,2,3]triazolo[1,5-*a*]pyridin-2-yl)-carbazol-9-ide) increased the ligand field strength from 17 500 to 24 400 cm^−1^, with only a modest rise in B from 550 to 600 cm^−1^. This balance favors near-infrared spin-flip transitions while extending their excited-state lifetimes. Despite these advances, carbazolide-based ligands exhibit also drawbacks, including low-lying charge-transfer states and geometric distortions, which limit lifetimes and prevent emission, contrasting with other near-infrared-emissive Cr^III^ systems. Additionally, we demonstrate an approach for estimating energies of dark, low-energy spin-flip states in Cr^III^ complexes *via* photoinduced electron transfer and Rehm–Weller analysis. Our results offer guidance on balancing ligand field strength and metal–ligand bond covalency to optimize the photophysical and photochemical properties of first-row transition metal complexes.

## Abstract 2

Carbazolide-based pincer ligands bearing NHCs/MICs enhance ligand field strength in Cr^III^ complexes with minimal impact on the Racah parameter *B*, maintaining NIR-active spin-flip excited states and extending their lifetimes, establishing design principles in first-row transition metal photophysics.

## Graphical abstract

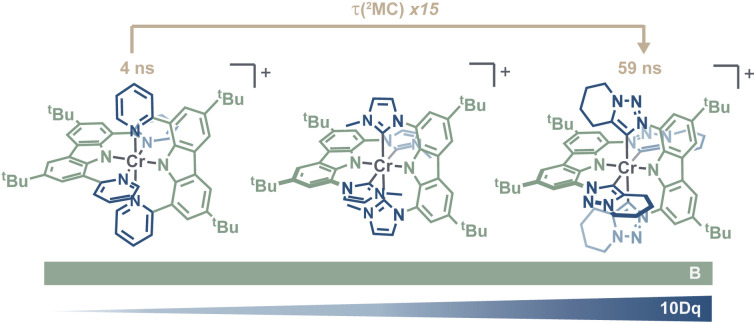

## Introduction

The 3d^3^ electronic configuration is particularly significant in first-row transition metals, such as in Cr^III^ and Mn^IV^, as it gives rise to metal-centered ‘spin-flip’ doublet excited states (^2^MC), characterized by minimal structural distortion and prolonged lifetimes.^1^ Octahedral Cr^III^ α,α′-diimine and α,α′,α″-triimine complexes, often referred to as molecular rubies, have been known for decades for their red-visible emission from these ^2^MC excited states (^2^T_1_/^2^E in octahedral geometry), with lifetimes reaching up to milliseconds.^2–4^ These spin-flip transitions occur within the t_2g_ metal orbital set, leading to only weak excited state distortion relative to the ground state and consequently slow deactivation rates. This behavior stands in stark contrast to that of triplet and quintet metal-centered states in 3d^6^ complexes (*e.g.* Co^III^, Fe^II^, Cr^0^, Mn^I^), where the population of an antibonding e_g_ metal orbital results in significant geometric distortion and faster excited-state deactivation.^5,6^

In Cr^III^ complexes, excited-state lifetimes and emission quantum yields can be enhanced by suppressing the repopulation of Jahn–Teller distorted quartet metal-centered excited states (^4^MC) *via* back-intersystem crossing from the luminescent ^2^MC states.^7^ A key strategy to achieve this relies on increasing the ligand field strength (10 Dq), thereby widening the energy gap between the quartet and doublet metal-centered manifolds.^7,8^ One effective approach to enhance the ligand field strength is the optimization of the chelating ligand bite angles towards an ideal octahedral coordination environment.^8,9^ The effects of the bite-angle optimization have been extensively studied in Ru^II^ complexes,^9,10^ and were later applied to Cr^III^ polypyridines and Co^III^ complexes to fine-tune their photophysical properties.^3,4,11–13^ These modifications have expanded their application potential in various fields, including photon upconversion,^14^ photoredox catalysis,^15,16^ circularly polarized luminescence emitters,^11,17^ and ratiometric pH optical sensors.^12,18^

**Fig. 1:**
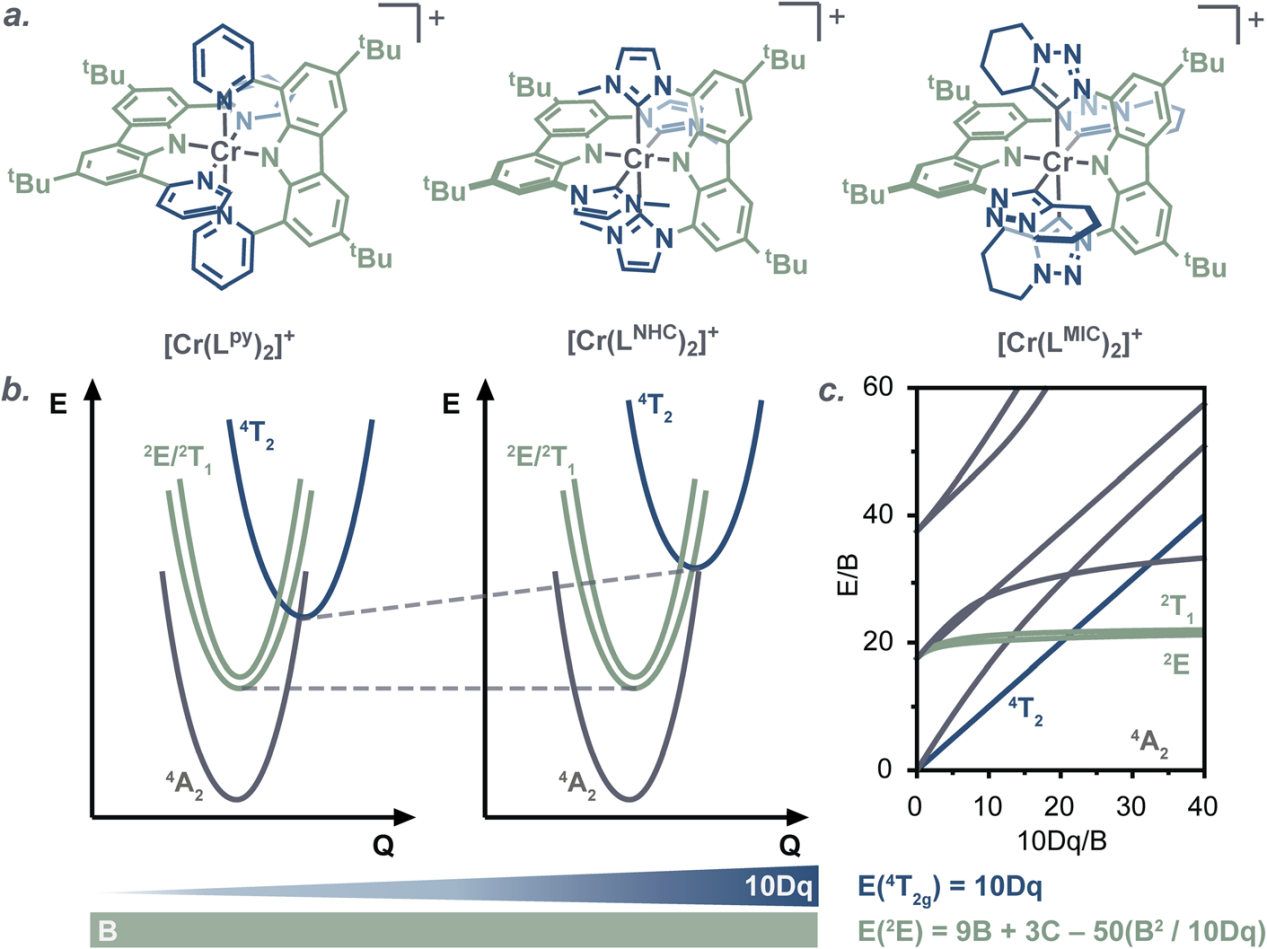
(a) Complex [Cr(L^py^)_2_]^+^ reported previously by our group,^21^ and complexes [Cr(L^NHC^)_2_]^+^ and [Cr(L^MIC^)_2_]^+^ investigated in this work; (b) schematic energy diagram showing the influence of the main electronic effects in the complex series; (c) Tanabe–Sugano diagram for octahedral d^3^ complexes.

According to the Tanabe–Sugano formalism (Fig. 1c),^19–21^ an increase in 10 Dq effectively destabilizes the ^4^MC states (among which ^4^T_2_ is most relevant) while having only a weak effect on the energies of the lowest ^2^MC states (^2^E, ^2^T_1_). This underscores a fundamental limitation of ligand design strategies that rely solely on the polypyridine systems.^22,23^ Consequently, some of the research focus in recent years has shifted toward gaining control over the ^2^MC excited-state energies by exploring novel ligand frameworks.^22,24–27^ Shifting the spin-forbidden electronic transitions further into the near-infrared-II region (NIR-II, between 1000 and 1700 nm) seems particularly appealing, as it could broaden Cr^III^ applications further to, for example, *in vivo* luminescent cell imaging in biomedicine.^28^

In an octahedral geometry, the energy of ^2^E excited state is primarily governed by d–d electronic repulsion, described by the Racah parameters *B* and *C*.^19,20^ This d–d electronic repulsion is influenced by the nephelauxetic effect, which arises from spatial extension of the d-orbitals facilitated by the ligand.^22^ This can reduce the Racah parameters compared to the free metal ion. A practical approach to modulating the nephelauxetic effect is by altering the covalency of metal–ligand bonds, as the Racah parameter *B* inversely correlates with it. In photoactive Cr^III^ complexes, this concept was first explored by us *via* introducing anionic amido units in the axial positions of the ligand scaffold in [Cr(L^py^)_2_]^+^ ([L^py^]^−^ = 3,6-di-*tert*-butyl-1,8-di(pyridin-2-yl)carbazol-9-ide, Fig. 1a).^24^ The presence of stronger covalent Cr–N_amido_ bonds reduced B to 550 cm^−1^, compared to the 700–800 cm^−1^ range observed for polypyridine Cr^III^ complexes.^3,12^ This, in turn, lowered the energy of the doublet excited states, resulting in emission at 1067 nm (∼1.2 eV) at 77 K in a glass matrix. Subsequent studies by other investigators reported a series of compounds, incorporating anionic amido and cyclometalated moieties, with the *B* values ranging from 550 to 670 cm^−1^, and spin-flip state emission observed between 910–980 nm (1.36–1.27 eV) in solution at room temperature.^25,29–31^

Introducing new anionic ligand scaffolds presents significant challenges, one of which relates to the empirical energy gap law or Marcus inverted-region behavior.^32^ This principle predicts that lowering the energy of a nested doublet excited state relative to the quartet ground state increases its deactivation rate. Indeed, studies on amido-based complexes [Cr(bpi^R^)_2_]^+^ ([bpi^R^]^−^ = 1,3-bis((2-R-pyridin-2-yl)imino)isoindolin-2-ide or 1,3-bis((4-R-pyridin-2-yl)-imino)isoindolin-2-ide, R = H, Me, OMe, NMe_2_), with electronically diverse substituents have confirmed that Cr^III^ excited-state dynamics follow this principle.^31^ The introduction of the electron-donating substituents at the *ortho*- or *para*-positions of the pyridine rings raises the energy of the photoactive doublet excited state and slows the radiative deactivation rates. Notably, in these complexes, the lowest excited state is a mixed ^2^MC/LMCT (MC = metal-centered, LMCT = ligand-to-metal charge transfer) state. In this specific scenario significant changes in the doublet excited-state energy can be rationalized solely by modifying substituents on the ligand backbone, without drastically changing the metal–ligand bonding situation.^22^

Another key challenge in optimizing photophysical properties is that reducing the Racah parameter *B* often comes at the expense of weakening the ligand field (10 Dq), rendering it difficult to fine-tune excited-state behavior. For instance, in the complexes [Cr(L^py^)_2_]^+^,^24^ Cr(*o*-CH_2_NMe_2_-Ph)_3_ (*o*-CH_2_NMe_2_-Ph = 4-(*tert*-butyl)-2-((dimethylamino)methyl)benzen-1-ide) and Cr(*o*-CH_2_P(Ph)_2_-Ph)_3_ (*o*-CH_2_P(Ph)_2_-Ph = 2-((diphenylphosphaneyl)methyl)-benzen-1-ide),^29^ the introduction of anionic π-donor ligands drastically destabilizes the t_2g_ orbitals, leading to a reduction in 10 Dq compared to some polypyridine-based Cr^III^ complexes.^3,12^ This not only results in shortening of the excited-state lifetime, but also prevents room-temperature emission in solution. To overcome this limitation, maximizing 10 Dq while keeping *B* values sufficiently low appears to be a reasonable strategy for optimizing ^2^MC state properties and minimizing non-radiative energy loss. A particularly well-suited example among known NIR-photoactive complexes is *fac*-Cr(ppy)_3_,^30^ which achieves a balance between these parameters. Its nearly ideal octahedral coordination and carbanionic ligand moieties yield a high 10 Dq of 26 320 cm^−1^ and a *B* parameter of 630 cm^−1^, resulting in a spin-flip doublet excited state with a ∼9 µs lifetime and 910 nm room-temperature emission in solution.

In this work, we aimed to explore the interplay between *B* and 10 Dq parameters using tridentate carbazolide ligand systems, structurally related to the previously reported [Cr(L^py^)_2_]^+^,^24^ to gain deeper insight into their impact on excited-state dynamics. Pyridine in the ligand scaffold was replaced with mesoionic carbene (MIC) or *N*-heterocyclic carbene (NHC) moieties in [Cr(L^MIC^)_2_]^+^ ([L^MIC^]^−^ = 3,6-di-*tert*-butyl-1,8-bis(4,5,6,7-tetrahydro-2*H*-[1,2,3]triazolo[1,5-*a*]pyridin-2-yl)carbazol-9-ide) and [Cr(L^NHC^)_2_]^+^ ([L^NHC^]^−^ = 3,6-di-*tert*-butyl-1,8-bis(imidazolin-2-yliden-1-yl)carbazolide) respectively, keeping the central carbazolide unit intact. The strong σ-donating properties of MIC and NHC binding motifs^33^ facilitate the energetic destabilization of the antibonding e_g_ metal orbitals, thereby increasing the ligand field splitting. As a result, these types of ligands are widely implemented in photoactive first row-transition metal complexes.^34–36^ However, to date, the photophysical properties of only two Cr^III^ carbene complexes—[Cr(ImPyIm)_2_]^3+^ (ImPyIm = 2,6-bis(imidazole-2-ylidene)pyridine) and [Cr(ImPy)_3_]^3+^ (ImPy = 2-imidazolylpyridine)—have been fully characterized.^37^ Interestingly, those two complexes exhibit drastically different behaviors, while [Cr(ImPy)_3_]^3+^ displays a long-lived (∼13 µs) and emissive (room temperature, solution) excited state, [Cr(ImPyIm)_2_]^3+^ manifests a dark state with a lifetime of less than 1 ns.^37^ This strong difference is speculated to arise from a lower-lying ^4^CT/MC manifold in [Cr(ImPyIm)_2_]^3+^, effectively deactivating ^2^MC *via* back-intersystem crossing.

Both ligand systems [L^NHC^]^−^ and [L^MIC^]^−^ have been previously reported in the literature.^38^ In particular, the [L^MIC^]^−^ ligand was utilized by some of us in the [Mn(L^MIC^)_2_]^2+^ complex,^39^ as the high oxidation state of Mn^IV^ requires electron-rich ligands, enabling the isolation of the complex as redox-stable species.^35^ Isoelectronic to Cr^III^, Mn^IV^ complexes feature greater ligand field splitting and metal–ligand bond covalency due to the higher effective nuclear charge of the metal center. Notably, lower-energy t_2g_ orbitals render ^4^LMCT/^2^LLCT (LLCT = ligand-to-ligand charge transfer) states easier accessible, and in some cases the ^2^LMCT state becomes the lowest photoactive state.^40–43^ Among the known examples, [Mn(dgpy)_2_]^4+^ (dgpy = 2,6-diguanidylpyridine) and exceptionally photorobust [Mn(PhB(MeIm)_3_)_2_]^2+^ ([PhB(MeIm)_3_]^−^ = tris(3-methylimidazolin-2-ylidene)phenylborate) have demonstrated ^2^LMCT states suitable for photocatalysis.^40–43^ We believe that the increased ligand field splitting in [Cr(L^MIC^)_2_]^+^ and [Cr(L^NHC^)_2_]^+^ complexes could ultimately align their excited-state relaxation patterns more closely with those of Mn^IV^, and anticipate that charge-transfer states will emerge to play an important role in the future.

## Results and discussion

### Synthesis and characterization

**Fig. 2:**
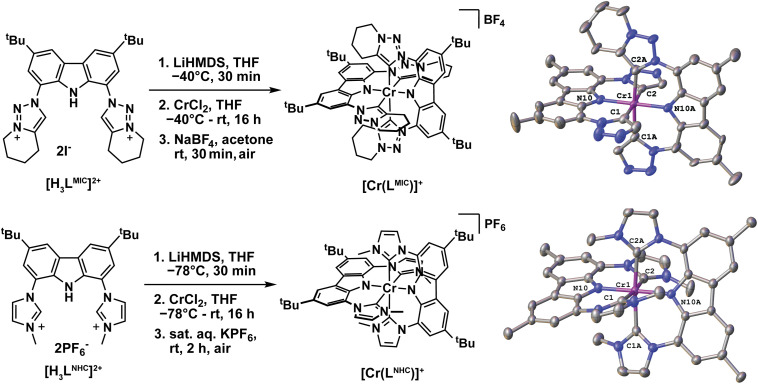
Synthesis of the Cr^III^ complexes [Cr(L^MIC^)_2_]^+^ and [Cr(L^NHC^)_2_]^+^ and their X-ray crystal structures with hydrogen atoms and counterions omitted for clarity. Additionally, in [Cr(L^MIC^)_2_]^+^ only one of the cyclohexyl residues is shown for clarity. Thermal ellipsoids are shown at the 50% probability level.

Complexes [Cr(L^MIC^)_2_]^+^ and [Cr(L^NHC^)_2_]^+^ were synthesized following a strategy similar to that used for the recently reported Mn^IV^ complex (Fig. 2).^39^ The pro-ligands [H_3_L^MIC^][I]_2_ or [H_3_L^NHC^][I]_2_ were deprotonated *in situ* in THF using lithium bis(trimethylsilyl)amide (LiHMDS), followed by addition of this solution to a suspension of chromium(ii) chloride (CrCl_2_) in THF. Subsequent oxidation of the complexes was achieved by aqueous work-up and aerobic salt metathesis using NaBF_4_ or KPF_6_ respectively. After purification by column chromatography and/or recrystallization, the target compounds were obtained in moderate yields of 24–50% (SI). Notably, the NHC complex ([Cr(L^NHC^)_2_]^+^ was also recently reported by Kunz, Heinze and co-worker and its excited state dynamics were thoroughly investigated.^44^

Single orange/red crystals of X-ray diffraction quality were obtained *via* vapor diffusion at room temperature from DCM/hexane ([Cr(L^MIC^)_2_]BF_4_) or acetone/hexane ([Cr(L^NHC^)_2_]BF_4_). The single crystal quality of the [BF_4_]^−^ salts was found to be the best, as other counterions lead to more complicated disorder and twinning effects. Both complexes crystallize in the monoclinic system with space group *P*2_1_/*c* with a positional disorder of the [BF_4_]^−^ anions over three ([Cr(L^MIC^)_2_]BF_4_) or two ([Cr(L^NHC^)_2_]BF_4_) positions and three disordered hexane ([Cr(L^MIC^)_2_]BF_4_) or 1.7 disordered acetone ([Cr(L^NHC^)_2_]BF_4_) molecules (Fig. S53 and S54). The Cr^III^ center is six-fold coordinated in a distorted octahedral coordination sphere by four imidazolylidene/triazolylidene units and the two amide donors. The C_carbene_–Cr–C_carbene_ angles are 176.38(11)°/173.00(12)° and 175.62(12)°/172.57(12)° for C1–Cr1–C2 and C1A–Cr1–C2A, while the N_amide_–Cr–N_amide_ angle N10–Cr1–N10A is found to be 179.90(13)°/178.85(11)° in [Cr(L^MIC^)_2_]BF_4_/[Cr(L^NHC^)_2_]BF_4_, respectively (Fig. 2, right). The values display that—despite the slightly higher steric bulk of the cyclohexyl ring in [Cr(L^MIC^)_2_]BF_4_ compared to [Cr(L^NHC^)_2_]BF_4_—the latter is substantially more distorted. This is also visible along the N_amido_–Cr–N_amido_ axis showing the two ligands to be almost perpendicular in [Cr(L^MIC^)_2_]BF_4_ (88.4(1)°), while the imidazolylidene substituents are strongly tilted in [Cr(L^NHC^)_2_]BF_4_ hence resulting in a smaller angle between the carbazole planes (61.89(1)°). We propose that his distortion is caused by unfavorable C–H_Imidazole_⋯C–H_carbazole_ repulsions, causing the imidazolylidene moieties to rotate more strongly out of plane compared to the triazolylidene. In the latter, the potential formation of favorable C–H_carbazole_⋯N_triazole_ interactions diminishes this rotation (Fig. 2, right). These unfavorable interactions are even more pronounced in [Cr(L^py^)_2_]^+^ with the six-membered pyridine donors.^24^

In accordance with enhanced covalency, the Cr–N_amido_ distances of 2.026(2)/2.002(2) Å (Cr1–N10) and 2.025(2)/2.004(2) Å (Cr1–N10A) are shortened compared to the Cr–C_carbene_ distances of 2.136(3)–2.144(3) Å/2.127(3)–2.160(3) Å in [Cr(L^MIC^)_2_]BF_4_/[Cr(L^NHC^)_2_]BF_4_. This observation is in line with axial compression distortion along the N_amido_–Cr–N_amido_ axis, previously reported in [Cr(L^py^)_2_]^+^.^24^ Notably, the metal–donor distances in [Cr(L^MIC^)_2_]BF_4_ are substantially larger, compared to the isoelectronic Mn^IV^ triazolylidene complex [Mn(L^MIC^)_2_]^2+^ previously reported by some of us (M–N_average_ 1.938(3); M–C_average_ 2.081(3) Å).^39^ Additionally, the metal–carbene distances in [Cr(L^MIC^)_2_]BF_4_ are longer compared to previous examples of heteroleptic and homoleptic Cr^III^ NHC complexes in the literature, *e.g.*, by Gibson and Steed (2.087(6)–2.120(6) Å)^45^ or by Scattergood *et al.* (2.093(4)–2.106(4) Å).^37^ We rationalize this bond elongation by the steric pressure of the cyclohexyl groups in the homoleptic [Cr(L^MIC^)_2_]BF_4_ complex. Further information on the structural parameters and data of complexes [Cr(L^MIC^)_2_]BF_4_ and [Cr(L^NHC^)_2_]BF_4_ can be found in the SI (Tables S2 and S3).

Evans NMR spectroscopy of the complexes revealed strong paramagnetism (Fig. S1–S8 displaying a magnetic moment of 3.91 µ_B_ for [Cr(L^MIC^)_2_]BF_4_ (Fig. S3) and 3.85 µ_B_ for [Cr(L^NHC^)_2_]BF_4_ (Fig. S7)), consistent with the presence of three unpaired electrons (expected spin-only value = 3.87 µ_B_) and a high-spin *d*^3^ configured Cr^III^ center. Additionally, [Cr(L^MIC^)_2_]BF_4_ and [Cr(L^NHC^)_2_]X (X = [PF_6_]^−^ or [BF_4_]^−^) were characterized by IR spectroscopy, high-resolution mass-spectrometry and elemental analysis (see SI for further information).

### Electrochemistry

**Fig. 3:**
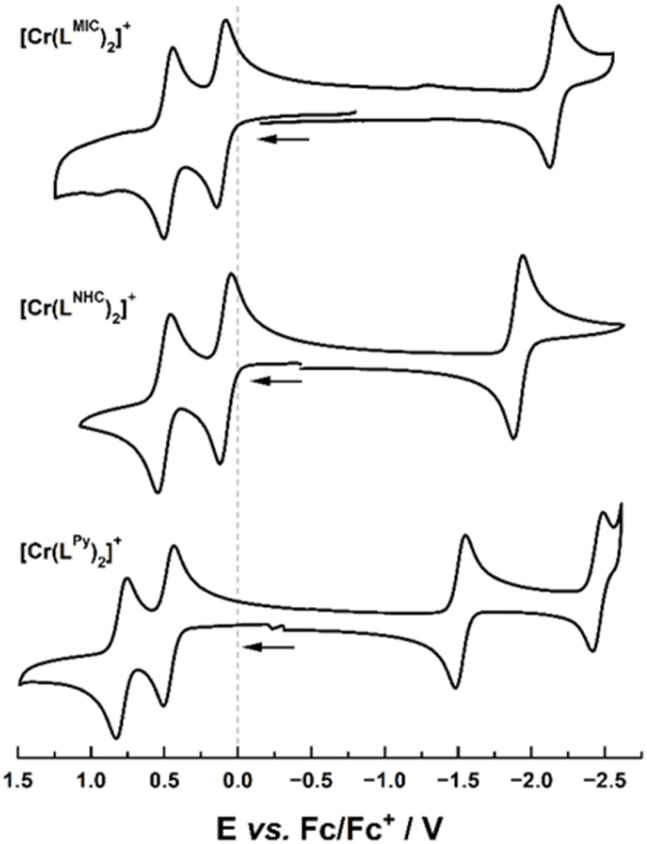
Cyclic voltammograms of 0.1 mM solutions of [Cr(L^MIC^)_2_]^+^ (top), [Cr(L^NHC^)_2_]^+^ (middle) and [Cr(L^Py^)_2_]^+^ (bottom) in 0.2 M NBu_4_PF_6_ in MeCN at 298 K. Scan rate: 100 mV s^−1^.

Cyclic voltammetry studies in acetonitrile at room temperature revealed the presence of three reversible redox events for both complexes (Fig. 3 and Table 1). Two reversible oxidations are observed at 0.11 V and 0.48 V *vs.* Fc/[Fc]^+^ for [Cr(L^MIC^)_2_]^+^, and at 0.08 V and 0.50 V ^44^*vs.* Fc/[Fc]^+^ for [Cr(L^NHC^)_2_]^+^. Given the proximity of the two redox processes and our previous investigations on the manganese analogue of [Mn(L^MIC^)_2_]^2+^,^39^ we propose that both oxidations are ligand-centered. Compared to [Cr(L^py^)_2_]^+^ (0.46 V and 0.78 V *vs.* Fc/[Fc]^+^, Fig. 3) the oxidations in [Cr(L^MIC^)_2_]^+^ and [Cr(L^NHC^)_2_]^+^ are anodically shifted, which is in agreement with the stronger σ-donor and weaker π-acceptor properties of NHC and MIC moieties relative to neutral *N*-donor ligands such as pyridines. Additionally, reversible reduction processes were recorded at −2.15 V and −1.91 V ^44^*vs.* Fc/[Fc]^+^ for [Cr(L^MIC^)_2_]^+^ and [Cr(L^NHC^)_2_]^+^, respectively, while [Cr(L^py^)_2_]^+^ shows a reduction event at −1.54 V *vs.* Fc/[Fc]^+^. The strong anodic shift of these values aligns with the σ-donor capacity of the ligand series and tentatively suggests—in combination with the pronounced shift compared to the previously reported Mn^IV^ complex [Mn(L^MIC^)_2_]^2+^—a metal-centered reduction process.^39^ However, spectroelectro-EPR spectroscopic measurements (Fig. S55 and S56) and DFT calculations (*vide infra*) rather indicate a ligand-centered process instead of the expected metal-centered reduction. Notably, all attempts to isolate any reduced or oxidized materials failed and EPR measurements after reduction further indicate instability of resulting complex (Fig. S55 and S56). For the oxidation, a fast colour change of the solutions is observed after addition of the oxidants, however the solutions quickly convert back to their original colour, indicating photo-instability of oxidizes species. This observation is in line with the ligand centered redox-process in the corresponding Mn^IV^ complex [Mn(L^MIC^)_2_]^2+^ which also rapidly decomposed at room temperature.^39^

**Table 1:** Electrochemical properties of the Cr^III^ complexes. *E*_red_, *E*_ox,1,_*E*_ox,2_ – half wave potentials for reversible reduction or oxidation processes, assignments of the loci of electron transfer are given in the parentheses

Complex	*E*_1/2_/V *vs.* Fc/[Fc]^+^		
	*E*_red_	*E*_ox,1_	*E*_ox,2_
[Cr(L^MIC^)_2_]^+^	−2.15 (L^0^/L^−^)	0.11 (L^+^/L^0^)	0.48
[Cr(L^NHC^)_2_]^+^	−1.91 (L^0^/L^−^)	0.08 (L^+^/L^0^)	0.50
[Cr(L^py^)_2_]^+^	−1.54 (Cr^III^/Cr^II^)^35^	0.46 (Cr^IV^/Cr^III^)^35^	0.78 (L^+^/L^0^)

To gain further information on the electronic structure of the native, mono-oxidized and mono-reduced complexes [Cr(L^MIC^)_2_]^+^, [Cr(L^MIC^)_2_]^2+^ and [Cr(L^MIC^)_2_]^0^ as well as its NHC and pyridine congeners [Cr(L^NHC^)_2_]^+^ and [Cr(L^Py^)_2_]^+^, computational investigations were performed at the density functional theory (DFT) level of theory. The DFT calculations support ligand-based oxidation as well as ligand reduction of [Cr(L^MIC^)_2_]^+^ (Tables S6–S7 and Fig. S57–S58).

### Ground- and excited-state properties: modelling and spectroscopy

**Fig. 4:**
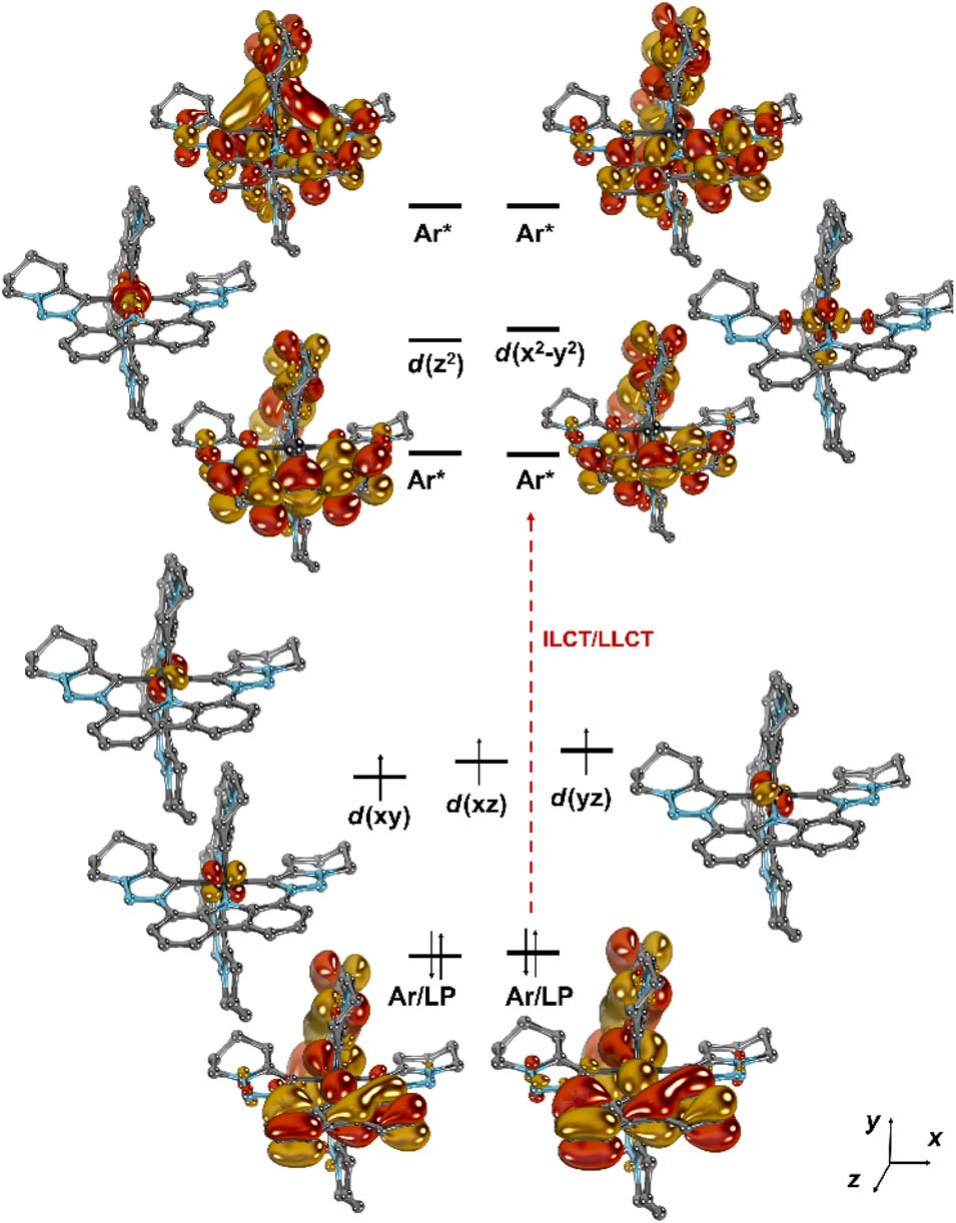
Frontier orbitals in [Cr(L^MIC^)_2_]^+^ according to CASSCF(7,11); the *t*Bu groups have been truncated by H-atoms to save computation time.

Comparative *ab initio* NEVPT2/CASSCF calculations were performed using the solid-state structural parameters of complexes [Cr(L^MIC^)_2_]^+^ (Fig. 4 and S62–S64), [Cr(L^NHC^)_2_]^+^ (Fig. S66), and [Cr(L^py^)_2_]^+^ (Fig. S67). Active spaces of saCASSCF(7,11) were chosen for [Cr(L^MIC^)_2_]^+^ (Fig. S63) and [Cr(L^py^)_2_]^+^ (Fig. S67), comprising the five 3d orbitals in combination with six ligand-based orbitals, thereof two formally occupied (carbazolide π-donor functionality) as well as four unoccupied π*-orbitals delocalized mainly across the carbazolides. For [Cr(L^NHC^)_2_]^+^, saCASSCF(7,10) with one π*-orbital less in the active space was required, as the considerably π-acidity of the NHCs led to otherwise difficult-to-converge wavefunctions (Fig. S66). Despite that the carbene complexes show near-ideal octahedral coordination geometries with orthogonal/coplanar ligand-π-systems (*vide supra*) and that the pyridine-congener is significantly distorted (*e.g.,* dihedral angle between the two pyridine ligands ∠C–N^py^–N^py^–C = 34°), the electronic structures of all complexes are similar. Fig. 4 depicts the computed molecular orbital diagram for [Cr(L^MIC^)_2_]^+^. The computations confirm that an idealized octahedral ligand field with a 3 + 2d-orbital splitting pattern is appropriate to understand the electronic structure, and that the covalency in the bonds with the donor ligands is moderate.

**Fig. 5:**
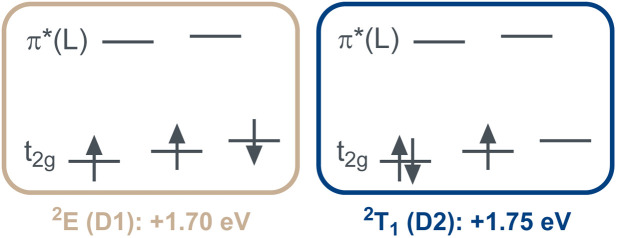
Electronic nature of the two energetically lowest doublet excited states D1 and D2 in [Cr(L^MIC^)_2_]^+^.

Notably, the two energetically lowest π*-orbitals of the carbazolido ligands are lower in energy than the d(*x*^2^–*y*^2^) and d(*z*^2^) orbitals. Hence, four low-intensity (starting at 525 nm, 2.36 eV, Q1–Q4; Table S10) and four high-intensity (starting at 460 nm, 2.71 eV, Q5–Q8) quartet mixed intra-ligand charge transfer (^4^ILCT) and ligand-to-ligand charge transfer (^4^LLCT) bands with predominant ^4^ILCT character are predicted. The weak d–d (MC) transitions (Q9–Q15) are predicted to occur in the energy range of 405–295 nm (^4^T_2_: 3.03 eV) and hence cannot be experimentally observed due to superposition by the intense charge transfer bands in the same spectral region. Corresponding vertical, that is still referring to the structural parameters of the quartet ground state, metal-centered doublet excited states are found at an energy range of 730 nm to 480 nm. The two energetically lowest D1 (1.70 eV, 730 nm) and D2 (1.75 eV, 710 nm) excited states represent the ^2^E and ^2^T_1_ spin-flip states (Fig. 5). Indeed, in the experimental UV-vis absorption spectrum of [Cr(L^MIC^)_2_]^+^ in acetonitrile, the most prominent band at 432 nm with a molar absorptivity (*ε*) of 19 300 M^−1^ cm^−1^ is assigned to ^4^LLCT/ILCT (*vide supra*). Two weaker intensity bands at 500 and 543 nm with *ε* of 2500 M^−1^ cm^−1^ and 1800 M^−1^ cm^−1^ similarly exhibit substantial charge transfer character.

**Fig. 6:**
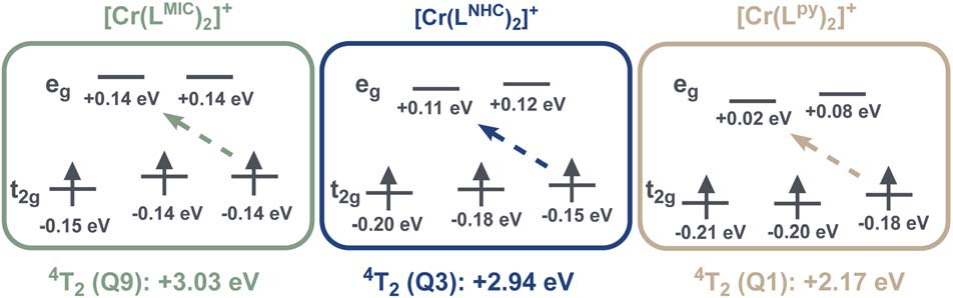
Comparison of 3d-orbital energies in complexes [Cr(L^py^)_2_]^+^, [Cr(L^NHC^)_2_]^+^ and [Cr(L^MIC^)_2_]^+^ as well as the corresponding energies for the transitions to the ^4^T_2_ excited states (10 Dq, respectively). See Fig. S70 for further details.

Upon moving to [Cr(L^NHC^)_2_]^+^, the energies of the ligand as well as the metal-centered states decrease in energy (Fig. 6). The lowest quartet state Q1 excited state appears at 690 nm (1.80 eV), and is anticipated to be of CT (charge-transfer) character akin to [Cr(L^MIC^)_2_]^+^. The ^4^T_2_ quartet state is predicted at 420 nm (Q3, 2.94 eV), and the ^2^T_1_ and ^2^E doublet states at 860 nm (D1, 1.44 eV) and 685 nm (D2, 1.80 eV), respectively.

**Fig. 7:**
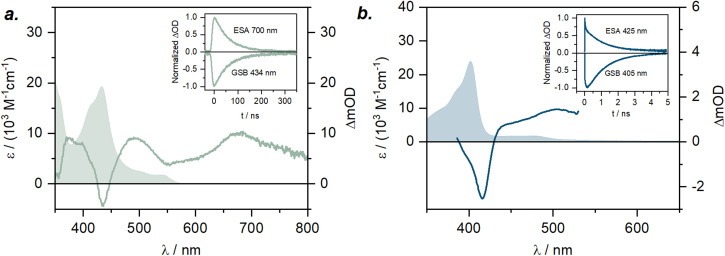
a) UV-vis absorption spectrum of [Cr(L^MIC^)_2_]^+^ in acetonitrile (filled area); UV-vis transient absorption (TA) spectrum (solid line) of [Cr(L^MIC^)_2_]^+^ in deaerated acetonitrile at 293 K, recorded following excitation at 532 nm with nanosecond pulses (∼70 mJ per pulse, ∼10 ns pulse duration), time-integrated over 200 ns; inset: decay of the excited-state absorption (ESA) signal at 700 nm and recovery of the ground state bleach (GSB) at 434 nm; (b) UV-vis absorption spectrum of [Cr(L^NHC^)_2_]^+^ in acetonitrile (filled area); TA spectrum (solid line) of [Cr(L^NHC^)_2_]^+^ in deaerated acetonitrile at 293 K, recorded following excitation at 355 nm with femtosecond pulses (∼0.2 mJ per pulse), at a delay time of 750 ps; inset: decay of ESA signal at 425 nm and recovery of GSB at 405 nm.

In agreement with the computational data, the ground state UV-vis electronic absorption spectrum of [Cr(L^NHC^)_2_]^+^ in acetonitrile (Fig. 7b) reveals an intense band at 402 nm with a molar absorptivity of 23 000 M^−1^ cm^−1^, which can be attributed to ^4^ILCT/LLCT transitions, while the bands at 450 and 477 nm (*ε* ∼2000 M^−1^ cm^−1^) have mainly ^4^LMCT character. In case of the literature-known [Cr(L^py^)_2_]^+^, the lowest-energy excited quartet state is not any more CT, but an MC state (Q1, ^4^T_2_, 2.17 eV). The Q3 state at 2.25 eV is associated with the first ILCT/LLCT transition, and the ^2^T_1_ and ^2^E states are found at 685 nm (1.80 eV) and 680 nm (1.82 eV), respectively. As the ^4^MC states are predicted at 24 400 cm^−1^ (409 nm, 3.03 eV) in [Cr(L^MIC^)_2_]^+^ and at 23 700 cm^−1^ (421 nm, 2.94 eV) in [Cr(L^NHC^)_2_]^+^, and according to the Tanabe–Sugano formalism (Fig. 1c), these energies correspond to the ligand field strength (10 Dq). Comparing these to the pyridine analogue [Cr(L^py^)_2_]^+^, which has a 10 Dq value of 17 500 cm^−1^ (2.17 eV), a consistent trend emerges: the ligand field splitting increases progressively going from pyridine to NHC to MIC complex, correlating with the enhanced σ-donating properties of the equatorial ligands. A larger magnitude of σ-donation leads to destabilization of the antibonding e_g_ orbitals and subsequent increase in the 10 Dq. In the orbital picture (*cf.*Fig. 6 and S65), complemented by *ab initio* ligand field theory (AILFT; Table S14),^46^ we find that the energy level of the vacant e_g_ orbital set is most elevated for the MIC-ligand, followed by the NHC and then pyridine. This observation is in line with the anticipated σ-donor strengths of these ligands MIC > NHC > py,^33,47,48^ namely in respect to their stereoelectronic properties, yet also their behavior in Co^III^ and Pd^II/IV^ complexes.^6,49^

A similar trend, yet weaker, is found for the populated t_2g_ orbital set, with the energies decreasing in the order MIC > NHC > py. Indeed, NHCs are better π-acceptors than MICs,^33,47,48,50^ and hence are expected to also comparatively lower the energies of the t_2g_ orbitals. We believe that the position of pyridine, albeit commonly considered to be less π-acidic than NHCs, is due to the distortion of the ligand framework MIC < NHC < py (*vide supra*), that is steric reasons. Indeed, the computations also suggest increasing *D*_4h_-character in the order MIC < NHC < py (Fig. S65 and Table S14).

Hand in hand with the computational predictions, we investigated the excited-state dynamics of the newly synthesized complexes using UV-vis transient absorption (TA) spectroscopy in acetonitrile. Following the excitation of [Cr(L^MIC^)_2_]^+^ at 532 nm with nanosecond pulses, the TA spectrum revealed a ground state bleach (GSB) at 434 nm, matching with the ^4^ILCT/LLCT band observed in the ground state absorption spectrum (Fig. 7a). Additionally, three intense excited state absorption (ESA) bands at 395, 490 and 670 nm are observed. These bands are associated with the electronic transitions originating from the ^2^MC state, which, based on NEVPT2/CASSCF calculations (SI, Table S10 and Fig. 4), represent the energetically lowest excited state. Given their intensity, those transitions are spin-allowed and occur within the doublet excited state manifold, leading to the population of the higher-lying ^2^MC or ^2^CT excited states.

Kinetic mono-exponential traces of ESA and GSB signals yield a ^2^MC excited state lifetime of 59 ns. Compared to the 4.4 ns lifetime observed in the parent complex [Cr(L^py^)_2_]^+^ (Fig. S42), this represents an increase of more than tenfold. This experimental observation aligns well with our computational results, which predict destabilization of the ^4^MC states and an increase in 10 Dq in the complex [Cr(L^MIC^)_2_]^+^ due to the strong σ-donation from the MICs (*cf.*Fig. 6). In addition, we anticipate minor changes in ^2^MC energies, as electron repulsion parameters are expected to be influenced to a relatively minor extent by the introduced ligand structural modification from pyridine to MIC. However, since the anionic carbazolide unit, primarily contributing to the Racah parameter *B*, remains unchanged, the destabilization of ^4^MC is projected to be significantly more pronounced than any variation in ^2^MC energy (see Table 2 and further discussion). This further leads to a larger energy gap between the lowest ^4^MC state and the key photoactive ^2^MC state, reducing the efficiency of back-intersystem crossing and ultimately contributing to a slower deactivation rate.

**Table 2:** Key photophysical properties of the Cr^III^ complexes. For further information see Table S1 and Fig. S43–S44

Complex	**[Cr(L** ^**MIC**^ **)**_**2**_ **]** ^**+**^	**[Cr(L** ^**NHC**^ **)**_**2**_ **]** ^**+**^	**[Cr(L** ^**py**^ **)**_**2**_ **]** ^**+**^
*λ*_abs_/nm (*ε*/M^−1^ cm^−1^)	432 (19 300); 500 (2500); 543 (1800)	402 (23 900); 450 (1700); 477 (1700)	408 (22 300); 490 (5300); 585 (600); 695 (70)
10 Dq^b^/cm^−1^	24 400	23 700	17 500
*B*/cm^−1^	600^a^	n/a	550 ^24^
*τ*(^2^MC)/ns	59	1.1	4.4
*E*(^2^MC)/eV	1.35 dark	n/a dark	1.16 ^24^ emissive (77 K)

a Estimated using *E*(^2^*E*) = 9 *B* + 3 *C* − 50(*B*^2^/10 Dq), assuming *C* = 3.2 *B*.^51^

b Calculated energies based on NEVPT2/CASSCF.

**Fig. 8:**
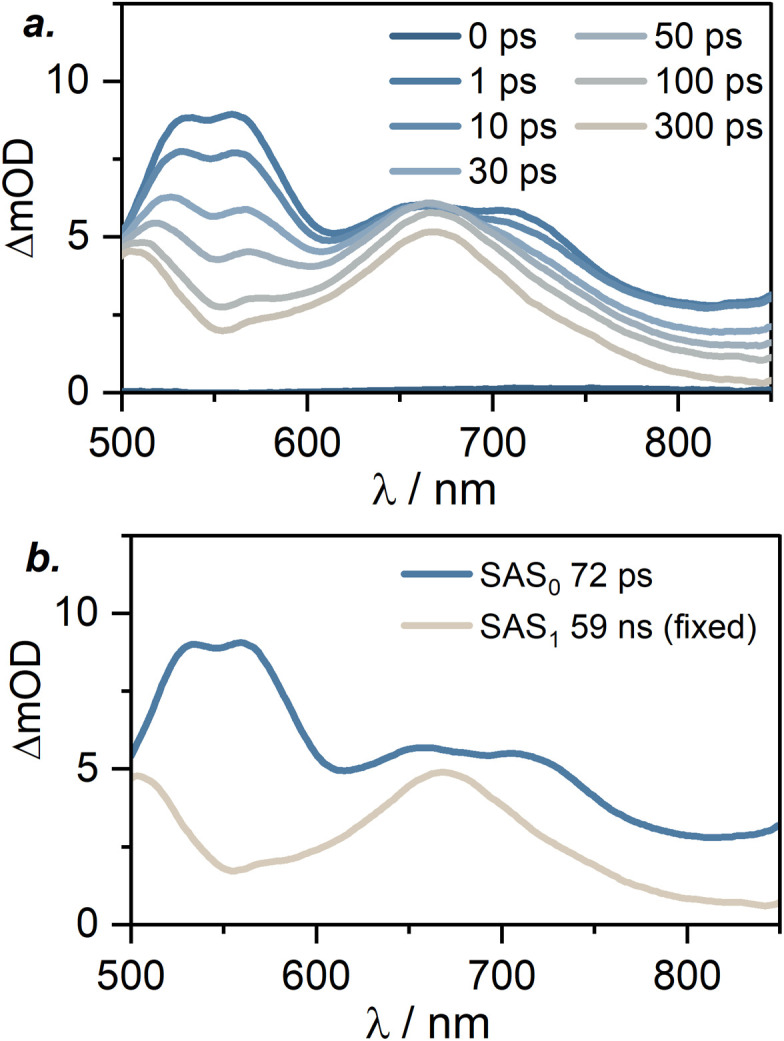
(a) UV-vis transient absorption spectra of [Cr(L^MIC^)_2_]^+^ in acetonitrile at 293 K, recorded at different delay times (shown in the graphs legend) following excitation at 430 nm with femtosecond pulses; (b) result of the global fit analysis. A sequential excited state population model was used for the fitting. Obtained species-associated spectra (SAS) and corresponding lifetimes are indicated in the inset.

To gain further insight into excited-state dynamics on faster timescales, we analyzed the TA spectra of [Cr(L^MIC^)_2_]^+^ following the femtosecond-pulse excitation at 430 nm, with delay times up to 300 ps, using a global fit with a sequential excited-state population model (Fig. 8 and S24–S25). Within 1 ps after the excitation, a characteristic intense ESA double band at 550–560 nm appears, along with ESA bands at 650 and 720 nm formed. Based on spectroelectro chemical data, we attribute these spectral features to the population of ^4^LLCT/ILCT states. Specifically, bands at 550 and 645 nm appear in the UV-Vis differential absorption spectrum of [Cr(L^MIC^)_2_]^+^ upon ligand oxidation (Fig. S26c), while bands at 580 and 720 nm emerge upon ligand reduction (Fig. S26b), supporting our assignment. Subsequently, with a lifetime of 72 ps (obtained from the global fit, see Fig. 7 and S24–S25) the 550 and 720 nm bands decay, giving rise to new ESA bands at 500 and 670 nm. As previously discussed (Fig. 8 and Table 2), these new bands are attributed to the long-lived ^2^MC state (59 ns), which does not decay within the experiment time window considered here. Moreover, the absence of spectral features associated with ligand oxidation or/and reduction further confirms our previous ^2^MC state assignment. In well-investigated Cr(acac)_3_ (acac = acetylacetonate) and [Cr(btmp)_2_]^3+^ (btmp = 2,6-bis(4-phenyl-1,2,3-triazol-1-yl-methyl)pyridine) complexes, intersystem crossing to the doublet states is known to occur on the sub-picosecond timescale and it proceeds more rapidly than internal conversion within the quartet manifold.^27,52^ In our measurements, the spectral signatures of the ^4^CT state persist at delay times beyond 70 ps. We therefore speculate that the 70 ps time constant obtained from the global fit likely reflects a combination of intersystem crossing, internal conversion and vibrational cooling. Consistent with this interpretation, internal conversion and vibrational cooling have previously been reported to occur on the timescales up to 300 ps in polypyridine Cr^III^ complexes.^12^

We anticipated that [Cr(L^NHC^)_2_]^+^ would exhibit photophysical properties intermediate between the parent [Cr(L^py^)_2_]^+^ and the novel [Cr(L^MIC^)_2_]^+^, as the σ-donation from NHC units is known to be weaker than that of MIC, bringing the 10 Dq value between the two. In the TA spectrum following excitation of [Cr(L^NHC^)_2_]^+^ at 355 nm with femtosecond pulses (pulse duration of ∼250 fs) at a delay time of 750 ps, a GSB at 405 nm and ESA bands at 425, 480 nm are observed (Fig. 7b). Similar to [Cr(L^MIC^)_2_]^+^, the GSB corresponds to the ^4^ILCT/LLCT band in the ground state absorption, while ESA features are attributed to the electronic transitions originating from ^2^MC state, predicated by DFT. The ^2^MC state in [Cr(L^NHC^)_2_]^+^ undergoes deactivation to the ground state with a lifetime of 1.1 ns. Unexpectedly, its faster excited-state relaxation compared to the parent complex [Cr(L^py^)_2_]^+^ suggests additional factors needed to be considered when rationalizing excited state dynamics. Aiming to do that, we examined the doublet and quartet excited state energies accessible upon the excitation at 355 nm (3.5 eV) (Table S13). Computational simulations predict low-lying ^4^LMCT states (1.8 and 1.9 eV) in close proximity to the ^2^MC states (1.8–1.85 eV), potentially contributing to a rapid deactivation of the latter (Fig. S65–S66 and Table S11). An analogical scenario was proposed for the homoleptic NHC complex [Cr(ImPyIm)_2_]^3+^, where excited-state decay occurred within the picosecond regime due to the low-lying charge-transfer states, populated through back-intersystem crossing.^37^

When discussing the excited-state properties of the parent complex [Cr(L^py^)_2_]^+^ and the novel complexes [Cr(L^MIC^)_2_]^+^ and [Cr(L^NHC^)_2_]^+^, we must address the key limitation regarding carbazolide ligands, namely unusually short excited state lifetimes relative to the other known NIR photoactive Cr^III^ species (^2^MC lifetimes typically in the microsecond range) and the absence of emission in solution at room temperature. There are several plausible explanations for that excited state behavior, which are applicable for all complexes discussed herein. The most significant factor is the electronic nature of the carbazolide unit. Low-energy charge-transfer transitions from π-orbitals localized on the carbazolide moiety to the metal's d-orbitals or ligand π* orbitals (mainly localized on pyridine or carbene units) become feasible, resulting in a high density of quartet and doublet charge-transfer and metal-centered states. This complicates predictions regarding the effects of structural modifications on excited-state dynamics, as is also reflected in our experimental and computational analysis (Table S1 and Fig. S43–S44). Additionally, the greater covalency of the axial Cr–N_amido_ bonds shortens their length compared to meridional Cr–C_carbene_ or Cr–N_pyridine_, inducing axial compression, which is observed already in the ground state (see XRD data, Fig. S53–S54 and Table S3). This, in turn, can potentially enhance Jahn–Teller distortion in the ^4^MC and ^4^CT excited states and facilitate faster non-radiative deactivation.^53^

### Rehm–Weller analysis

The absence of emission at room temperature in solution and technical limitations in detecting luminescence at 77 K in a glass matrix hindered the experimental determination of the ^2^MC excited state energies for both [Cr(L^MIC^)_2_]^+^ and [Cr(L^NHC^)_2_]^+^ complexes. Consequently, two alternative approaches were considered for estimating the doublet excited state energy. The first involves detecting spin-forbidden transitions *via* UV-vis ground state absorption spectroscopy, which however was not feasible in our case due to technical limitations. The second approach relies on studying a series of photoinduced electron transfer (PET) reactions, enabling estimation of the excited-state redox potential (*E*^0^(*D*^+^*/*D*^2+^)) and zero-point energy (*E*_00_) using eqn (1).^54^ This methodology has previously been applied to estimate the excited-state reduction potential of the photoactive ^3^MC state in Co(iii) polypyridine complex.^55^ The excited state lifetime of 59 ns for [Cr(L^MIC^)_2_]^+^ in solution allows for diffusion-based excited-state redox reactivity, and consequently renders it suitable for this type of analysis.

1 *E*_0_(*D*^+^*/*D*^2+^) = *E*_0_(*D*^+^/*D*^2+^) – *E*_00_/*e*

2 Δ*G*_ET_ = [*E*^0^(*D*^+^*/*D*^2+^) – *E*^0^(*A*/*A*^˙−^)] × *e*

3
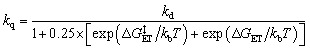

4 Δ*G*^‡^_ET_ = [(Δ*G*_ET_/2)^2^ + Δ*G*^‡^_ET_(0)^2^]^1/2^ + Δ*G*_ET_/2

**Fig. 9:**
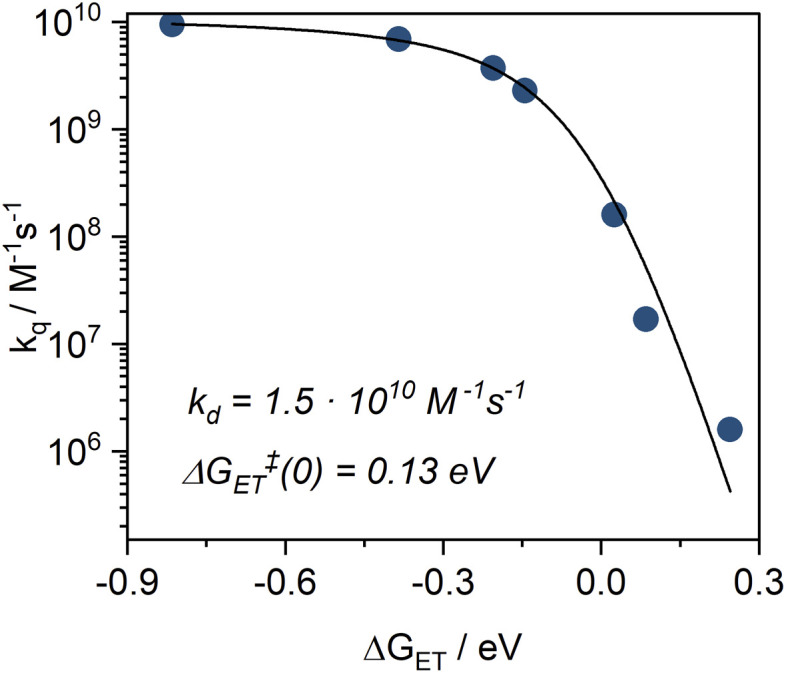
Rehm–Weller plot, showing a dependency between bimolecular electron transfer rate constants (*k*_q_) for [Cr(L^MIC^)_2_]^+^ with selected electron acceptors and free energy (Δ*G*_ET_). Best fit for *E*^0^(*D*^+^*/*D*^2+^) = −1.24 V *vs.* Fc/[Fc]^+^; *k*_d_ = (1.49 ± 0.02)·10^10^ M^−1^ s^−1^; Δ*G*_ET_^‡^(0) = 0.13 eV. Advanced data fitting results and discussion are presented in Fig. S34.

**Fig. 10:**
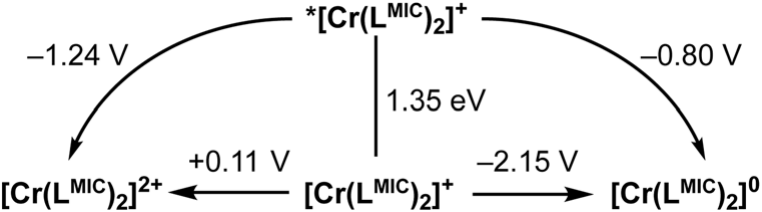
Latimer diagram of [Cr(L^MIC^)_2_]^+^. Ground state redox potentials obtained *via* cyclic voltammetry and given *vs.* Fc/[Fc]^+^. The excited state oxidation potential was determined through a series of bimolecular photoinduced electron transfer reactions involving selected electron acceptors (see Fig. 9 and Table 3). Successively, zero-point energy (*E*_00_) and excited state reduction potentials were calculated using eqn (1) and (2).

Based on the NEVPT2/CASSCF calculations, the doublet excited state energy is estimated at 1.70 eV, and the ground state oxidation potential (*E*^0^(*D*^+^/*D*^2+^)) of the complex was determined (*via* cyclic voltammetry) to be +0.05 V (*vs.* Fc/[Fc]^+^). Applying eqn (1), the excited state oxidation potential can be estimated to be higher than −1.65 V *vs.* Fc/[Fc]^+^. With this threshold in mind, we screened a series of electron acceptors for the oxidative quenching, including nitrobenzene and benzoquinone derivatives, with reduction potentials (*E*^0^(*A*/*A*˙^−^)) ranging from −1.48 V to −0.42 V *vs.* Fc/[Fc]^+^. We then performed PET experiments between the complex and selected quenchers (Table 3) in acetonitrile, using TA spectroscopy to monitor the reaction kinetics. The excited state lifetime of the ^2^MC state was analyzed as a function of quencher concentration using Stern–Volmer plots (Fig. S27–S33). The obtained bimolecular quenching rate constants (*k*_q_) were then evaluated as a function of the reaction driving force (Δ*G*_ET_, eqn (2)) within the framework of the Rehm–Weller formalism (Fig. 9). Full Rehm–Weller plot analysis using eqn (3) and (4) with varied *E*^0^(*D*^+^*/*D*^2+^) was performed, allowing the best fit for *E*^0^(*D*^+^*/*D*^2+^) = −1.24 V. Further, a diffusion rate constant of *k*_d_ = (1.49 ± 0.02)·10^10^ M^−1^ s^−1^ and the self-exchange activation free energy Δ*G*_ET_^‡^(0) = 0.131 ± 0.002 eV were obtained from the fit. Those values are in line with the observations for quenching studies between [Cr(dqp)_2_]^3+^ and a series of electron donors, with *k*_d_ = 1.7 × 10^10^ M^−1^ s^−1^ and Δ*G*_ET_^‡^(0) = 0.14 eV, as well as with other related studies with Cr^III^ complexes.^15,56^ Finally, using eqn (1), an *E*_00_ value of 1.35 eV was calculated, allowing us to construct a complete Latimer diagram for [Cr(L^MIC^)_2_]^+^ (Fig. 10).

**Table 3:** Bimolecular oxidative excited-states quenching of [Cr(L^MIC^)_2_]^+^ with a series of electron acceptors. Reduction potentials and bimolecular quenching rate constants for quenchers in acetonitrile at 293 K

Quencher	*E*(*A*/*A*^˙−^)/V *vs.* Fc/[Fc]^+^	*k*_q_/M^−1^ s^−1^	Δ*G*_ET_/eV
Tetrachloro-1,4-benzoquinone	−0.42	9.5 × 10^9^	−0.82
1,4-Naphthoquinone	−0.85	6.9 × 10^9^	−0.39
1,4-Benzoquinone	−1.03	3.7 × 10^9^	−0.21
1,4-Dinitrobenzene	−1.09	2.3 × 10^9^	−0.15
4-Nitrobenzaldehyde	−1.26	1.6 × 10^8^	0.02
4-Nitrobenzophenone	−1.32	1.7 × 10^7^	0.08
Nitrobenzene	−1.48	1.6 × 10^6^^a^	0.24

a Estimated upper limit, approximation for the quenching rate constant was made based on experiments indicating less than 10% quenching in the concentration range below 1 M.

Turning attention back to the NHC complex [Cr(L^NHC^)_2_]^+^, the excited state lifetime of 1.1 ns is too short for efficient quenching studies and the application of the same *E*_00_ estimation method used for [Cr(L^MIC^)_2_]^+^. According to the computational simulation, the doublet excited state energy is calculated at 1.44 eV, combining it with *E*^0^(*D*^+^/*D*^2+^) = 0.08 V *vs.* Fc/[Fc]^+^ in eqn (1), we estimate *E*^0^(*D*^+^*/*D*^2+^) to be above −1.36 V *vs.* Fc/[Fc]^+^. To probe the feasibility of the PET reaction, we tested methyl viologen (*E* = −1.00 V *vs.* Fc/[Fc]^+^) as an electron acceptor (Fig. S39). Less than 5% reduction in the excited state lifetime was observed for 100 mM concentration of the quencher, allowing the bimolecular quenching rate constant for this reaction to be estimated around 6.5 × 10^8^ M^−1^ s^−1^.

When comparing the experimentally determined doublet excited state energy (*E*_00_) of 1.35 eV for [Cr(L^MIC^)_2_]^+^ complex and 1.16 eV for the parent [Cr(L^py^)_2_]^+^ complex (Table 2),^24^ we observe a minor destabilization of the ^2^MC excited state by 0.19 eV. This can be rationalized by the decreased covalency of the Cr–C_carbene_ bond compared to the Cr–N_pyridine_ bond. Indeed, we calculated the electron repulsion parameter *B*, which inversely correlates with metal-bond covalence, using the Tanabe–Sugano formalism (see equation in Fig. 1c), and the value for [Cr(L^MIC^)_2_]^+^ was determined to be 600 cm^−1^ compared to 550 cm^−1^ for [Cr(L^py^)_2_]^+^.^24^

From these observations and previous results, we can now conclude how structural modifications in our new complex affect their excited state properties. Confirming our initial hypothesis, replacing the equatorial ligand moieties from pyridines to strong σ-donating MICs leads to a significant increase in the ligand field strength while maintaining minor changes in the Racah *B* parameter, keeping it sufficiently low (see Table 2). Furthermore, the increased ^4^MC–^2^MC energy gap, from 1.01 eV in [Cr(L^py^)_2_]^+^ to 1.68 eV in [Cr(L^MIC^)_2_]^+^ complex, supports the observed reduction in the non-radiative deactivation rate of the ^2^MC excited state in the new complex.

### Photocatalytic activity of [Cr(L^MIC^)_2_]^+^

**Fig. 11:**
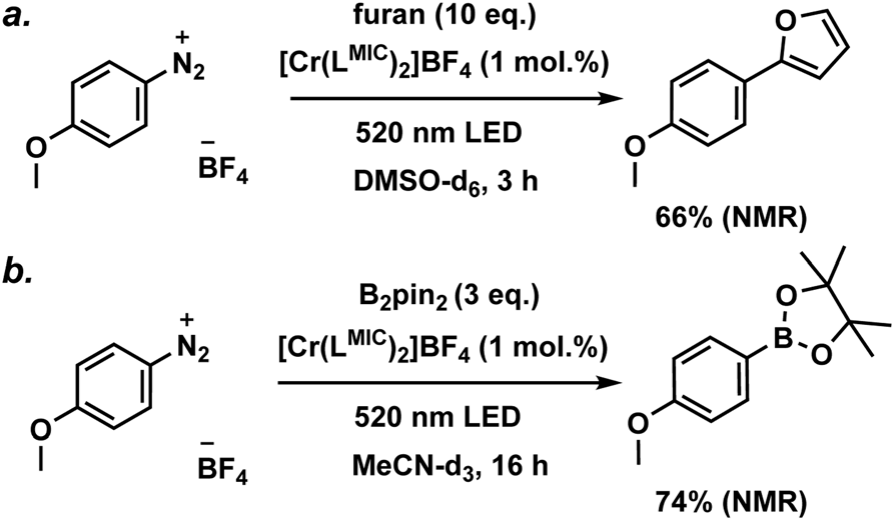
(a) Photocatalytic C–H arylation of furan with 4-methoxyphenyl diazonium tetrafluoroborate; (b) photocatalytic borylation of 4-methoxyphenyl diazonium tetrafluoroborate with bis(pinacolato)diboron. Control experiments are shown in the SI (Fig. S46, S47, S50 and S51).

Based on these insights, we investigated the [Cr(L^MIC^)_2_]^+^ complex, which exhibits the longest excited state lifetime (59 ns) in the carbazolide series and an excited state oxidation potential of −1.24 V (*vs.* Fc/[Fc]^+^), for photoinduced electron transfer catalysis. Aryl diazonium salts are known to form diazo-radicals upon single electron reduction, which can further undergo C–N bond dissociation, resulting in the generation of aryl radicals and the evolution of nitrogen. The produced aryl radical can be used for a vast scope of transformations, including C–H arylations, borylations, and phosphorylations.^57–59^ In our study, we focused on a *para*-methoxy-substituted phenyldiazonium salt with a reduction potential of −1.07 V (*vs.* Fc/[Fc]^+^). Using our novel Cr^III^ complex as a photocatalyst, we successfully demonstrated model C–H arylation and borylation reactions (Fig. 11) involving the mentioned substrate. Both photocatalytic transformations are anticipated to proceed *via* well-known, literature-reported mechanisms (Fig. S48 and S52).^36,57–59^

The photocatalytic performance of [Cr(L^MIC^)_2_]^+^ was first evaluated for the C–H arylation of furan with a *p*-methoxyphenyl diazonium salt (Fig. S45–S48). Under an argon atmosphere, a catalyst loading of 1 mol%, 10 eq. of furan, and 0.11 mM of the diazonium substrate were irradiated using 520 nm LED for 3 hours. A substrate conversion of >95% and yield of 66% (NMR, relative to an internal standard, Fig. S45) were obtained. The photocatalytical borylation of *p*-methoxyphenyl diazonium salt with bis(pinacolato)diboron was tested as a second model transformation. Substrate conversion of >99% and 74% product yield were achieved after 16 hours of irradiation with a 520 nm LED of the reaction mixture (see Fig. S49–S51, catalyst loading of 1 mol%, 3.0 eq. of bis(pinacolato)diboron and 0.11 mM of substrate).

As indicated by the NMR yields, selected model transformations proceed efficiently under our photocatalytic conditions. For comparison, this class of reactions can be also performed using Fe^III^, Cu^I^, Ru^II^, Os^II^ and Ir^III^ transition metal complexes as photocatalysts, as well as metal-free systems such as eosin Y, under visible-light irradiation, typically affording moderate to high yields.^36,57–59^ It should be noted that the literature-reported systems display appreciable thermal reactivity, which accounts for the yields of up to 20% observed in the control experiments, both in related studies and this work (Fig. S46, S47, S50 and S51).^36,57–59^

## Conclusions

A significant milestone in the development of Cr^III^ and Mn^IV^ complexes was achieved when the nephelauxetic effect was strategically incorporated into ligand design, enabling red-shifting of spin-flip transitions into the NIR region.^22,24,25,29,31^ Building on this concept, a deeper understanding of how ligands influence the properties of these low-energy excited states is essential for their future applications in photochemistry and biomedicine.

In this context, we demonstrated a feasible method for estimating the energy of dark ^2^MC excited states using photoinduced electron transfer and Rehm–Weller analysis. This approach has proven to be a valuable tool for probing the photophysical behavior of our new [Cr(L^MIC^)_2_]^+^ complex and Cr^III^ systems in general.

Ultimately, we established a versatile strategy for tuning the ratio between ligand field strength and the Racah parameter *B*, by using pincer-type ligands based on covalent carbazolide core units and modifying the donor groups from pyridines, as in our previously reported [Cr(L^py^)_2_]^+^,^24^ to stronger σ-donors such as MICs in the novel [Cr(L^MIC^)_2_]^+^. This enhanced 10 Dq from 17 500 to 24 400 cm^−1^ while only modestly increasing *B* from 550 to 600 cm^−1^. This strategy achieves a precise balance between the key parameters: a low interelectronic repulsion (reflecting metal–ligand bond covalency) favorable for spin-flip transitions in the near-infrared, combined with sufficiently high ligand field strength to extend excited-state lifetimes. Together, these results provide a clear framework for designing NIR-photoactive Cr^III^ complexes with tunable excited-state properties.

## Author contributions

Synthesis was carried out by PY, BW and AS. PY conducted spectroscopic investigations and analysis, photocatalysis experiments. Electrochemical investigations were carried out by PY and FRN. EPR spectroscopic investigations were carried out by DL. Theoretical investigations were performed by DM. Crystal structure solution and analysis was performed by FT and SH. The idea was conceived by SH and OSW. The manuscript was written by PY, BW, DM, OSW and SH and proof read by all authors.

## Conflicts of interest

There are no conflicts to declare.

## Supplementary Material

SC-OLF-D5SC09069E-s001

SC-OLF-D5SC09069E-s002

## Data Availability

CCDC 2304955 ([Cr(L^MIC^)_2_]BF_4_) and 2343870 ([Cr(L^NHC^)_2_]BF_4_) contain the supplementary crystallographic data for this paper.^61*a*,*b*^ Supplementary information: ^1^H, Evans, UV-Vis, IR and elemental analysis data for all complexes. In addition, the SI contains supporting spectra on the photophysical measurements, EPR spectra, computational details and additional crystallographic information.^60^ See DOI: https://doi.org/10.1039/d5sc09069e.
