# Supplementary material for: Interplay between ligand field strength and the nephelauxetic effect in chromium(iii) complexes with anionic amido ligands
Source: Chem Sci. 2026 Feb 19;17(17):8760–71. doi: 10.1039/d5sc09069e (PMC12984063; doi:10.1039/d5sc09069e)
Supplement: SC-017-D5SC09069E-s001 [file SC-017-D5SC09069E-s001.pdf]

Supplementary Information (SI) for Chemical Science.
This journal is © The Royal Society of Chemistry 2026

Interplay between Ligand Field Strength and the Nephelauxetic Effect in Chromium(III) Complexes with Anionic Amido Ligands

- Supporting Information -

Polina Yaltseva,‡ Benjamin Wittwer,‡ Daniel Leitner, Florian Neururer, Frank Tambornino, Alina Schmidt, Dominik Munz,* Oliver S. Wenger* and Stephan Hohloch*

Table of Contents

1. Experimental Procedures 3

2. NMR Spectroscopy 6

3. IR Spectroscopy 10

4. Mass spectrometry 11

5. Cyclic Voltammetry 13

6. Photophysical Properties of $[Cr(L^{MIC})_2]^+$ 18

UV-vis transient absorption 18

Alternative Rehm-Weller fitting analysis 25

7. Photophysical Properties of $[Cr(L^{NHC})_2]^+$ 26

Ground state UV-vis absorption 26

UV-vis transient absorption 26

8. Photophysical Properties of $[Cr(L^{py})_2]^+$ 29

Ground state UV-vis absorption 29

UV-Vis transient absorption 29

9. Dependence of the non-radiative deactivation rate constant on the experimental and theoretical/calculated energy gaps 31

10. Photoredox catalysis with $[Cr(L^{MIC})_2]^+$ 32

11. Crystallographic Information 38

12. EPR Spectroscopy 41

13. Computational Details 42

Chromium MIC Complex $[Cr(L^{MIC})_2]^+$ 43

XYZ Coordinates 62

14. References 79

15. Author Contributions 80

1. Experimental Procedures

Methods and experimental procedures

All chemicals for synthesis were purchased from commercial suppliers in reagent grade and used without any further purification. 3,6-di-*tert*-butyl-1,8-bis(3-methylimidazolin-2-yliden-1-yl)carbazolide bis(hexafluorophosphate) (H_3L^{NHC}),[1] 3,6-di-*tert*-butyl-1,8-bis(4,5,6,7-tetrahydro-2H-[1,2,3]triazolo[1,5-a]pyridin-8-ium)carbazolide bisiodide (H_3L^{MIC}),[2,3] 3,6-di-*tert*-butyl-1,8-di(pyridine-2-yl)-carbazole (H_3L^{py}) and $[Cr(L^{py})_2]PF_6$ were synthesized according to literature procedures.[4] Solvents for synthesis and spectroscopy were purchased in extra dry (>99.5% purity) and HPLC grades, solvents for synthesis were additionally degassed via freeze-pump-thaw cycles.

NMR spectra were recorded on Bruker Avance III instruments with 250 or 400 MHz proton frequency. Deuterated solvents were purchased from Eurisotop (Cambridge Isotope Laboratories). Chemical shifts were reported in δ values in ppm and were referenced to the solvent residue peak.[5]

Cyclic voltammetry was conducted under Ar atmosphere in an electrochemical cell containing a glassy carbon disk as the working electrode, a silver wire as the counter electrode, and a saturated calomel electrode (SCE) as the reference electrode. In case of the ferrocene/ferrocenium referenced CVs, they were measured using a three-electrode setup using a glassy carbon working electrode, a platinum counter electrode and a silver-wire pseudo reference electrode. Ferrocene was used an internal standard.

Steady-state absorption spectroscopy was measured on a Varian Cary 5000 UV-Vis-NIR instrument. UV-vis transient absorption measurements on the nanosecond timescale were carried out on an LP920-KS instrument from Edinburgh Instruments. A frequency-tripled pulsed Nd:YAG laser (Quantel Q-smart 450, ca. 10 ns pulse width) with a beam expander (BE02-355 from Thorlabs) in the beam path was used for excitation at 355 nm (pulse energy of ~100 mJ) and a frequency-doubled Nd:YAG laser (Quantel Brilliant b, ca. 10 ns pulse width) with a beam expander (GBE02-A from Thorlabs) in the beam path was used for excitation at 532 nm (pulse energy of ~70 mJ). An iCCD camera from Andor was used to detect transient absorption spectra, and single-wavelength kinetics were recorded with a photomultiplier tube (Hamamatsu). UV-vis transient absorption measurements on the picosecond timescale were carried out with a TRASS instrument from Hamamatsu and a mode-locked picosecond Nd:YVO_4/YAG laser from Ekspla (PL2251B-20-SH/TH/FH with PRETRIG option, ca. 30 ps pulse width) as excitation light source. The frequency-tripled (355 nm) output of this laser powered an Ekspla PG402-264 OPA with a resulting energy output of 6 mJ at 430 nm. UV-vis transient absorption measurements on the femtosecond timescale were carried out with a HARPIA-TA instrument (Light Conversion). Excitation light is generated by a PHAROS laser (Light Conversion, Yb:KGW laser, source wavelength of 1030 nm, pulse width of ca. 190 fs, pulse energy ~0.2 mJ), and the actual pump light wavelength was generated by an OPA ORPHEUS (Light Conversion, used ~ 90% of fundamental pulse). The probe light was generated by a sapphire (5 mm thickness; ~10% of the fundamental pulse were used to generate a white light super-continuum).

Elemental analysis was carried out by Sylvie Mittelheisser on a Vario Micro Cube instrument (University of Basel, Department of Chemistry) and by Roland Egger on a Vario Unicube Instrument (University of Innsbruck, Department of General, Inorganic and Theoretical Chemistry). Electrospray ionization high resolution mass spectra (ESI-HRMS) were recorded by Dr. Michael Pfeffer (University of Basel, Department of Chemistry) on a Bruker maxis 4G ESI-Q-TOF instrument and by Dr. Thomas Müller on aThermo Fisher Scientific, Q Exactive hybrid quadrupole-Orbitrap mass spectrometer instrument (University of Innsbruck).

All photochemical transformations were carried out on an NMR scale. The typical experimental setup included: an NMR tube placed in a beaker with cold water and the light source - 520 nm Kessil LED lamp.

EPR measurements were performed on a Brucker Magnettech ESR5000 X-band spectrometer with a temperature control unit in 3 mm o.d. fused silica tubes. Spectro electrochemistry was performed using a BioLogic potentiostat with a three-electrode

array (working electrode: platinum, counter electrode: platinum, reference electrode: silver) with NBu_4BArF_{24} as supporting electrolyte. Analysis was performed using the EasySpin package for MatLab. Values were obtained by least-squares fitting to the experimental spectra using the garlic and pepper functions.[6]

X-ray diffraction crystallography was performed at the University of Innsbruck (Bruker D8 Quest) All crystals were kept at low temperatures throughout data collection. Data collection, refinement and reduction was performed using the ApexIV. All structures were solved with SHELXT[7] and refined using the OLEX 2 software package[8] using SHELXL[9] least square refinements. Strongly disordered solvent molecules have been removed using the SQUEEZE operation.[10] All nonhydrogen atoms were refined anisotropically, and hydrogen atoms were included at the geometrically calculated positions and refined using a riding model.

Synthesis of the Cr^{III} complexes

[Cr(L^{MIC})$_2$]BF_4: The pro-ligand azolium salt [H_3L^{MIC}][I]$_2$ (400 mg, 0.514 mmol, 2.0 eq) was dissolved in THF (5.0 mL) and stirred for one minute before being cooled to –40°C. To this, LiHMDS (301 mg, 1.80 mmol, 7.0 eq) was added as a solution in THF (5.0 mL, cooled to – 40 °C) and the reaction mixture turned first dark red in colour, followed by a colour change to vibrant green and strongly fluorescent. The deprotonation reaction is stirred for 30 min while warming to room temperature. During this time, $CrCl_2$ (31.6 mg, 0.257 mmol, 1.0 eq) was suspended in THF (5.0 mL) and cooled to –40 °C as well. The deprotonated ligand was then slowly added at –40 °C to the chromium chloride suspension, resulting in a dark red/ brown reaction mixture, which was stirred overnight at room temperature. The next day, the reddish-brown reaction mixture was evaporated to dryness and taken out of the glovebox. The residue was dissolved in acetone and $NaBF_4$ (329 mg, 3.00 mmol, 10 eq) was added as a solid. The mixture was stirred for 30 min in an open flask and then slowly diluted with water to form a brown-orange precipitate/suspension. The solid was filtered off, and re-dissolved in acetone. This procedure was repeated two more times, to ensure complete anion exchange to tetrafluoroborate. The orange precipitate is dried in air and purified my flash column chromatography using dichloromethane/methanol (99:1). It is recommended to use a long (30 cm) column to ensure good separation from the remaining impurities. Finally, the orange material is recrystallized by slow (vapour) diffusion of pentane into dichloromethane at room temperature to give the title compound as large, transparent orange blocks in a yield of 50% (151 mg, 0.127 mmol). Anal. calcd. for [Cr(L^{MIC})$_2$]BF_4 (%): C, 65.13; H, 6.49; N, 16.62. Found (%): C, 65.43; H, 6.75; N, 16.10. HRMS (ESI, positive mode): calcd. for [Cr(L^{MIC})$_2$]$^+$ (m/z): 1092.5779. Found (m/z): 1092.5726.

[Cr(L^{NHC})$_2$]PF_6: The pro-ligand [H_3L^{NHC}][I]$_2$ (84.0 mg, 115 μmol, 2.0 eq) was charged into a 25 mL Schlenk flask, which was evacuated and refilled with nitrogen (3 cycles). Dry and deaerated THF (10 mL) was added to the flask and the solution was cooled down to -78°C, followed by a dropwise addition of LiHMDS (1 M in THF, 559 μL, 559 μmol, 8 eq). After completing the addition, the mixture was stirred at -78°C for 30 minutes, followed by the dropwise addition of a solution containing $CrCl_2$ (7.20 mg, 59.0 μmol, 1.02 eq) in dry and deaerated THF (5.0 mL). The mixture was subsequently allowed to gradually warm up to room temperature and was then stirred for 16 hours at this temperature. The resulting dark orange suspension was filtered through celite, and the celite layer was further washed with dichloromethane. The filtrate was concentrated *in vacuo*, and the resulting solid was suspended in 2 mL of THF. The bright orange precipitate was then filtered and washed with an additional 2 mL of cold THF. The crude product was dissolved in 1 mL of acetone, and saturated aqueous KPF_6 was added. (*Note: For the preparation of the [BF_4]$^–$ salt, $NaBF_4$ is used instead of KPF_6*). The mixture was stirred at room temperature for 30 minutes, followed by filtration of the precipitate. The precipitate was then washed with 5 mL of diethyl ether and dried *in vacuo*. Finally, the product (15.0 mg, 13.9 μmol, 24%) was obtained as dark orange needle-like crystals after recrystallization via solvent layering (dichloromethane – diethyl ether).

Anal. calcd. for [Cr(L^{NHC})$_2$]PF_6·(H_2O) (%): C, 61.59; H, 6.09; N, 12.82. Found (%): C, 61.48; H, 5.86; N, 12.75. HRMS (ESI, positive mode): calcd. for [Cr(L^{NHC})$_2$]$^+$ (m/z): 928.4720. Found (m/z): 928.4724.

[Cr(L^{NHC})$_2$]BF_4: Anal. calcd. for [Cr(L^{NHC})$_2$]BF_4·(CH_2Cl_2) (%): C, 62.19; H, 6.04; N, 12.72. Found (%): C, 61.89; H, 5.74; N, 12.42.

HRMS (ESI, positive mode): calcd. for [Cr(L^{NHC})$_2$]$^+$ (m/z): 928.4720. Found (m/z): 928.4694.

2. NMR Spectroscopy

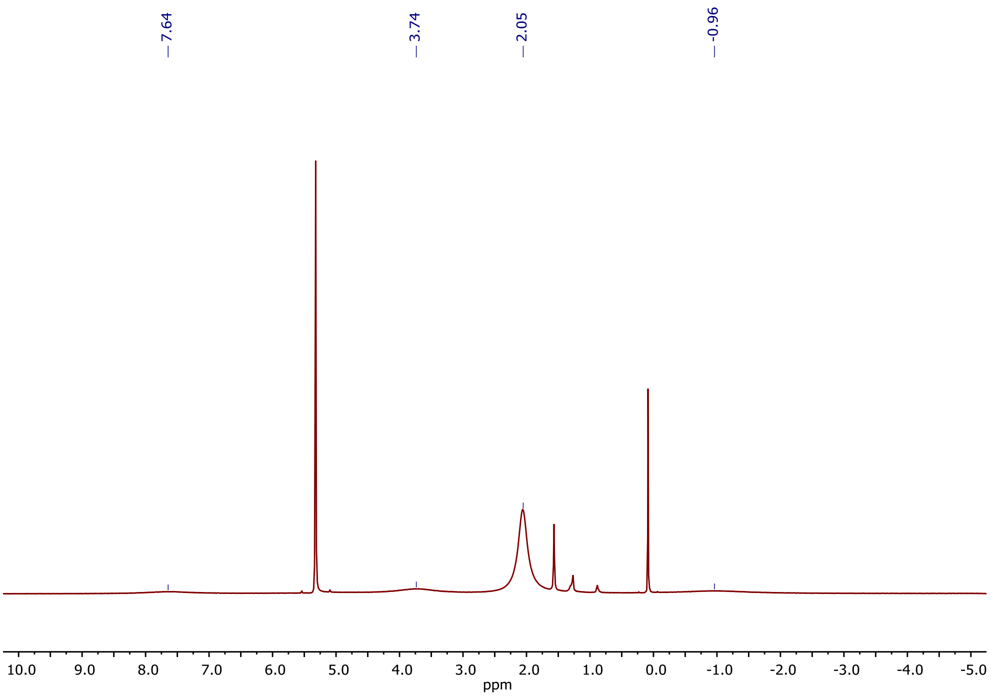

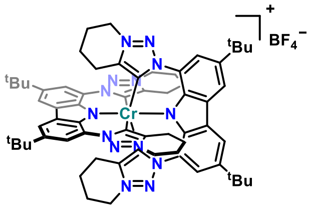

Figure S1: ^{1}H NMR of ***[Cr(L^{MIC})$_2$][BF$_4$]*** *in CD_2Cl_2 at 298 K from +10 – –5 ppm.*

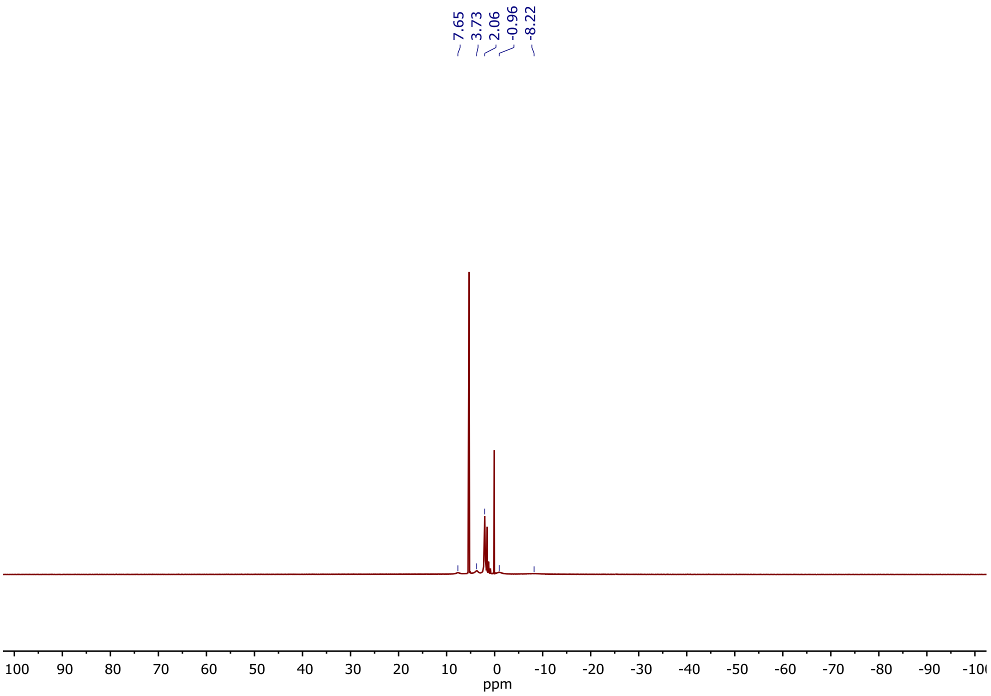

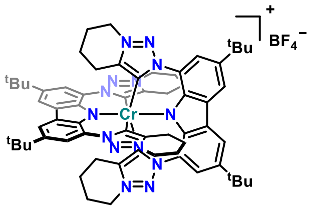

Figure S2: ^{1}H NMR of ***[Cr(L^{MIC})$_2$][BF$_4$]*** *in CD_2Cl_2 at 298 K from +100 – –100 ppm.*

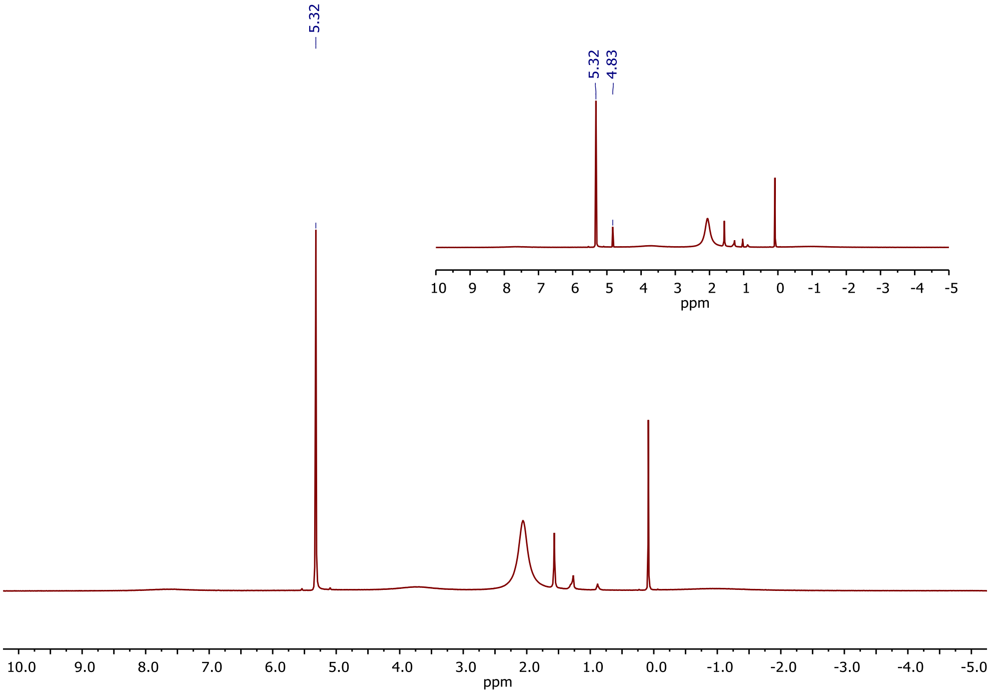

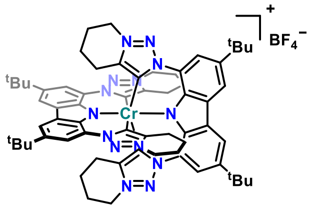

*Figure S3: ^{1}H NMR of **[Cr(L^{MIC})$_2$][BF$_4$]** in CD_2Cl_2 at 298 K from +10 – –5 ppm. Inlay shows the NMR from the Evans Method measurement showing the peak difference between the NMR solvent and the internal standard (NMR solvent in the capillary)*

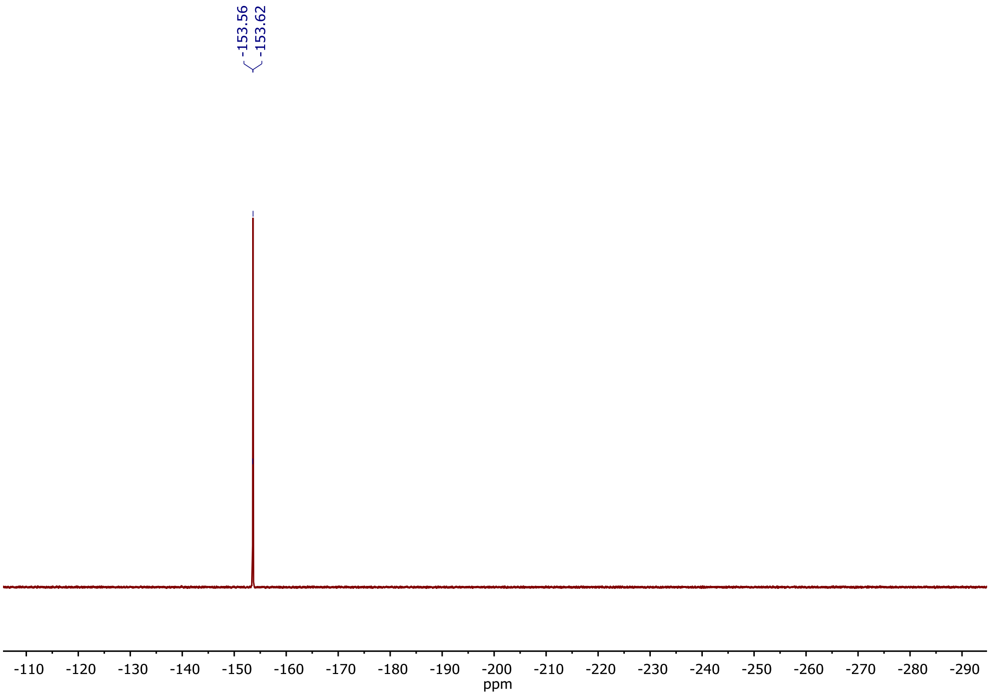

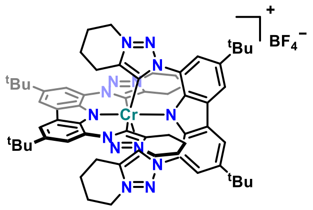

*Figure S4: ^{19}F NMR of **[Cr(L^{MIC})$_2$][BF$_4$]** in CD_2Cl_2 at 298 K from –110 – –290 ppm.*

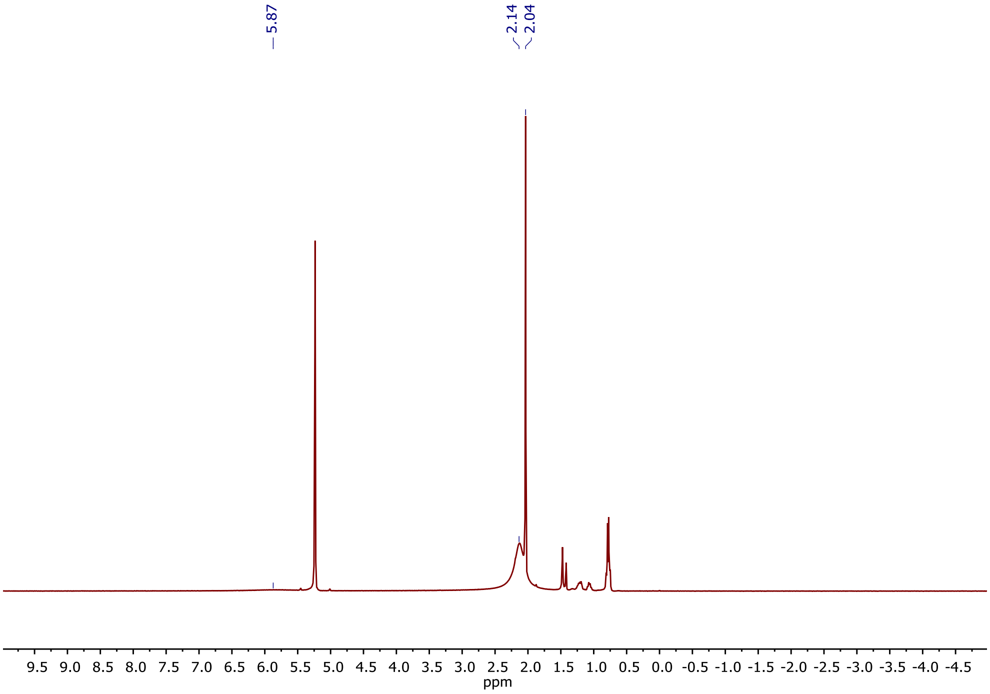

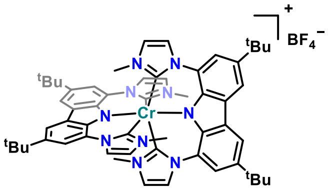

Figure S5: ¹H NMR of ***[Cr(L^{NHC})$_2$][BF$_4$]*** *in CD_2Cl_2 at 298 K from +10 – –5 ppm.*

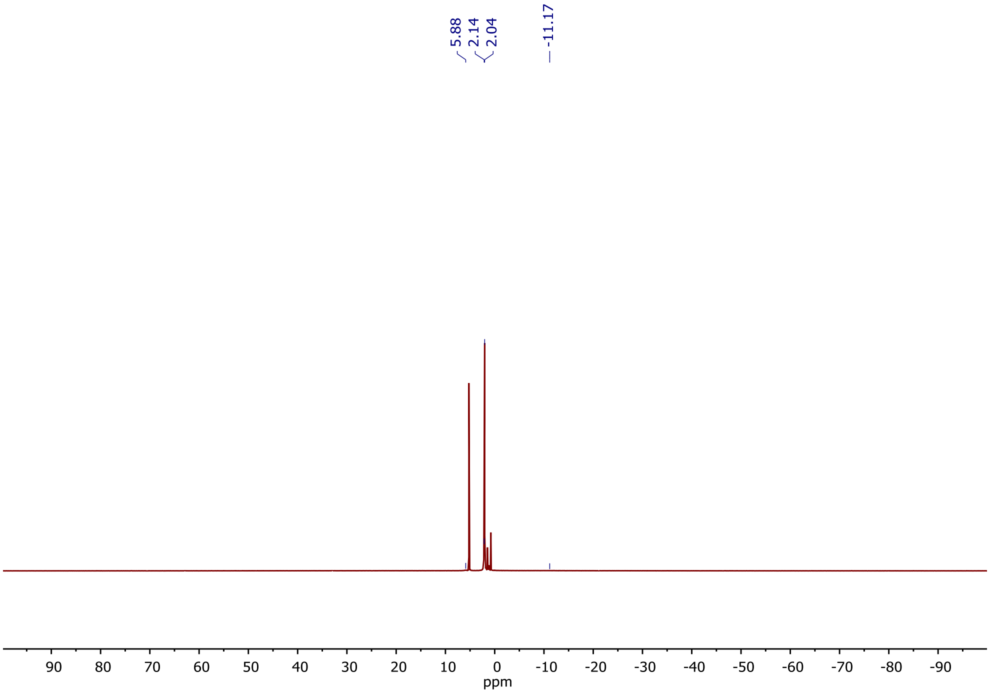

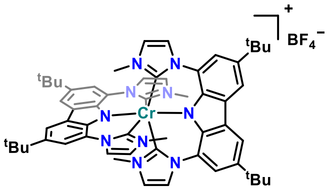

Figure S6: ¹H NMR of ***[Cr(L^{NHC})$_2$][BF$_4$]*** *in CD_2Cl_2 at 298 K from +100 – –100 ppm.*

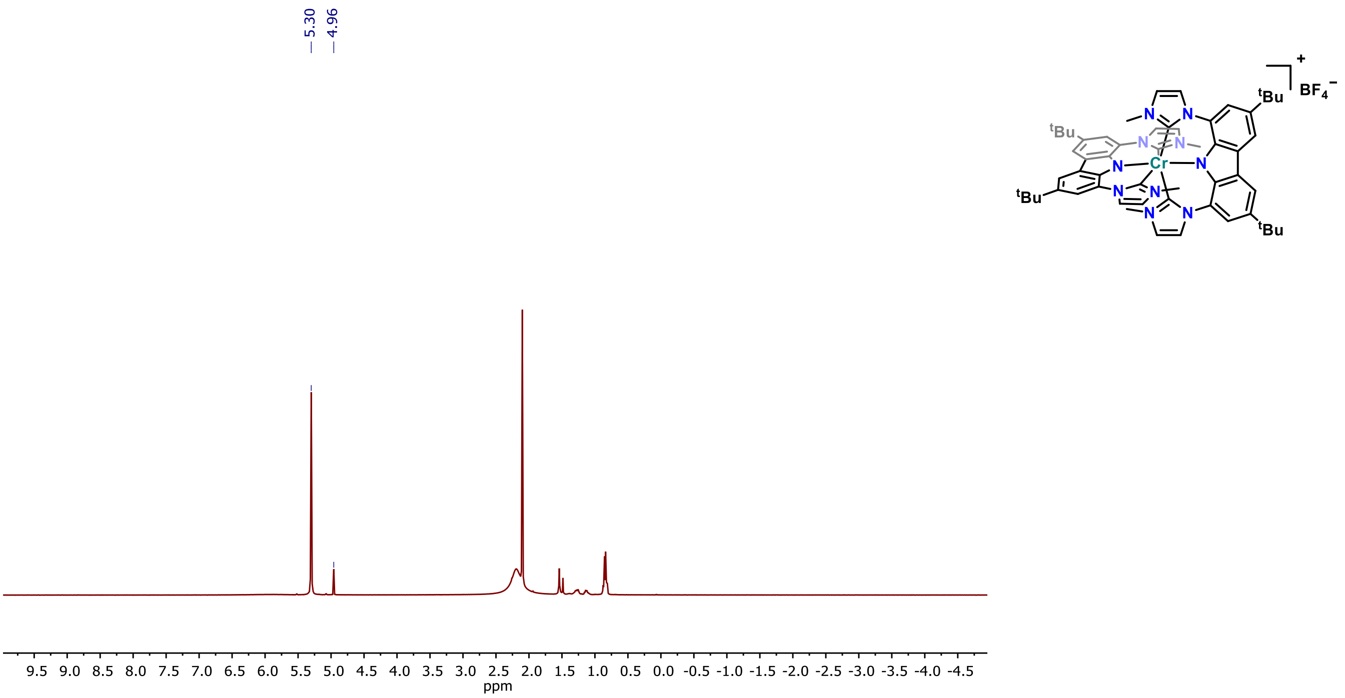

*Figure S7: ^{1}H NMR of **[Cr(L^{NHC})$_2$][BF$_4$]** in CD_2Cl_2 at 298 K from +10 – –5 ppm. The Evans Method measurement showing the peak difference between the NMR solvent and the internal standard (NMR solvent in the capillary)*

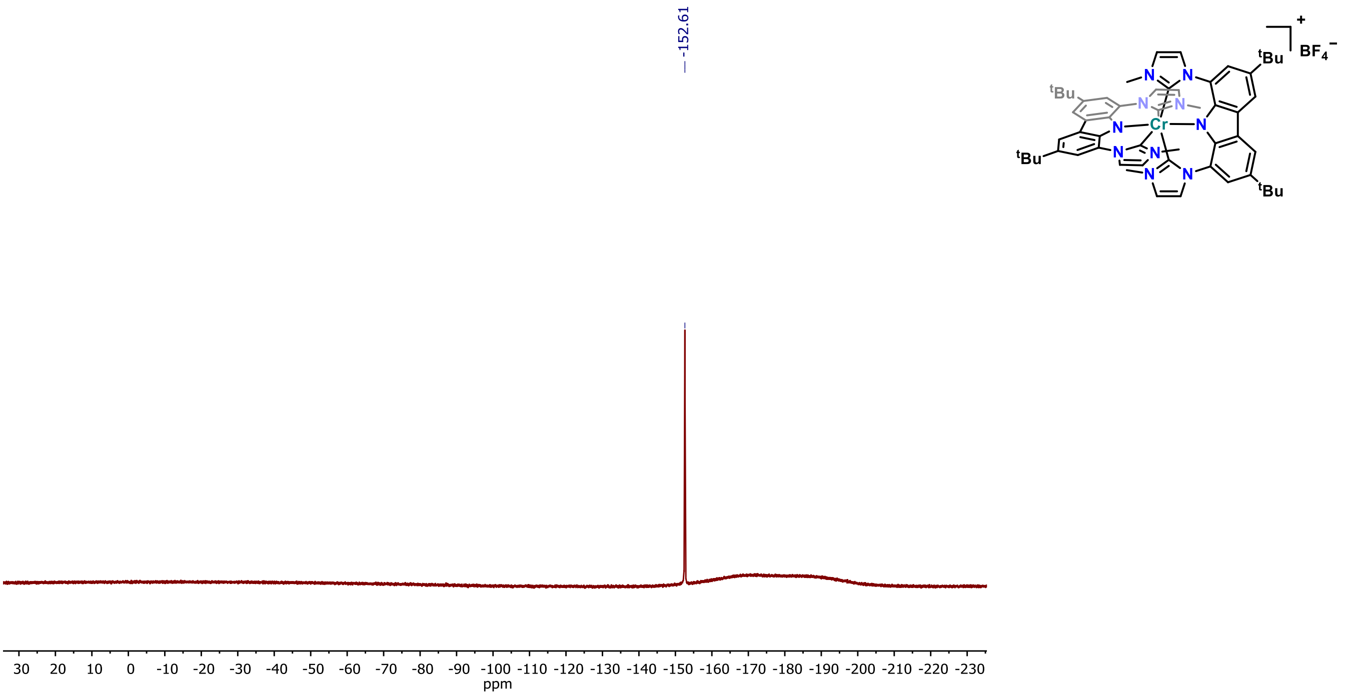

*Figure S8: ^{19}F NMR of **[Cr(L^{NHC})$_2$][BF$_4$]** in CD_2Cl_2 at 298 K from +30 – –230 ppm.*

3. IR Spectroscopy

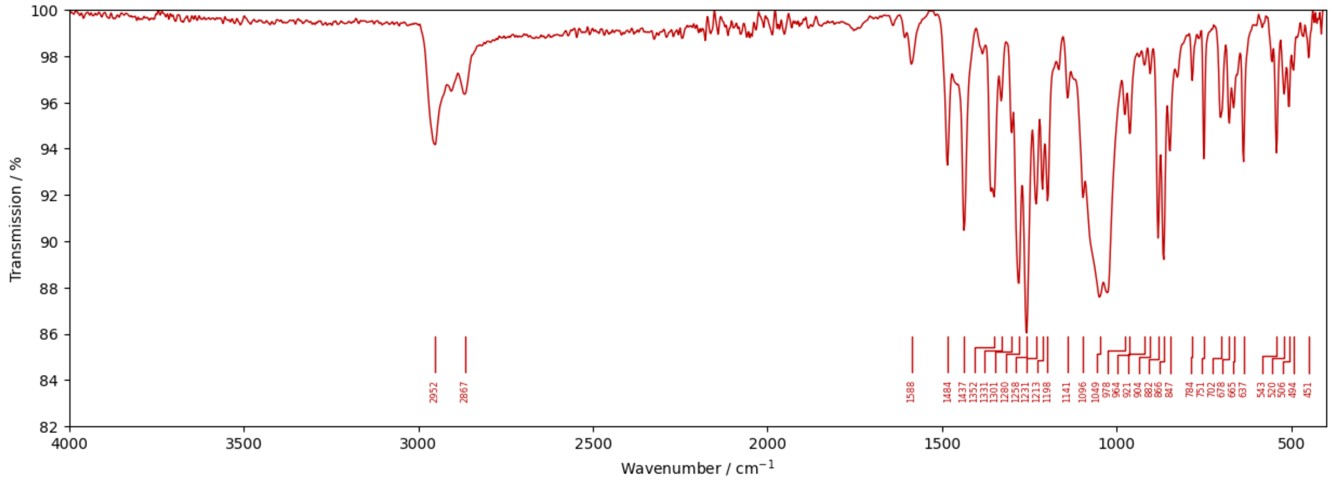

Figure S9: FT-IR (ATR) of ***[Cr(L^{MIC})$_2$][BF$_4$]*** *at 298 K.*

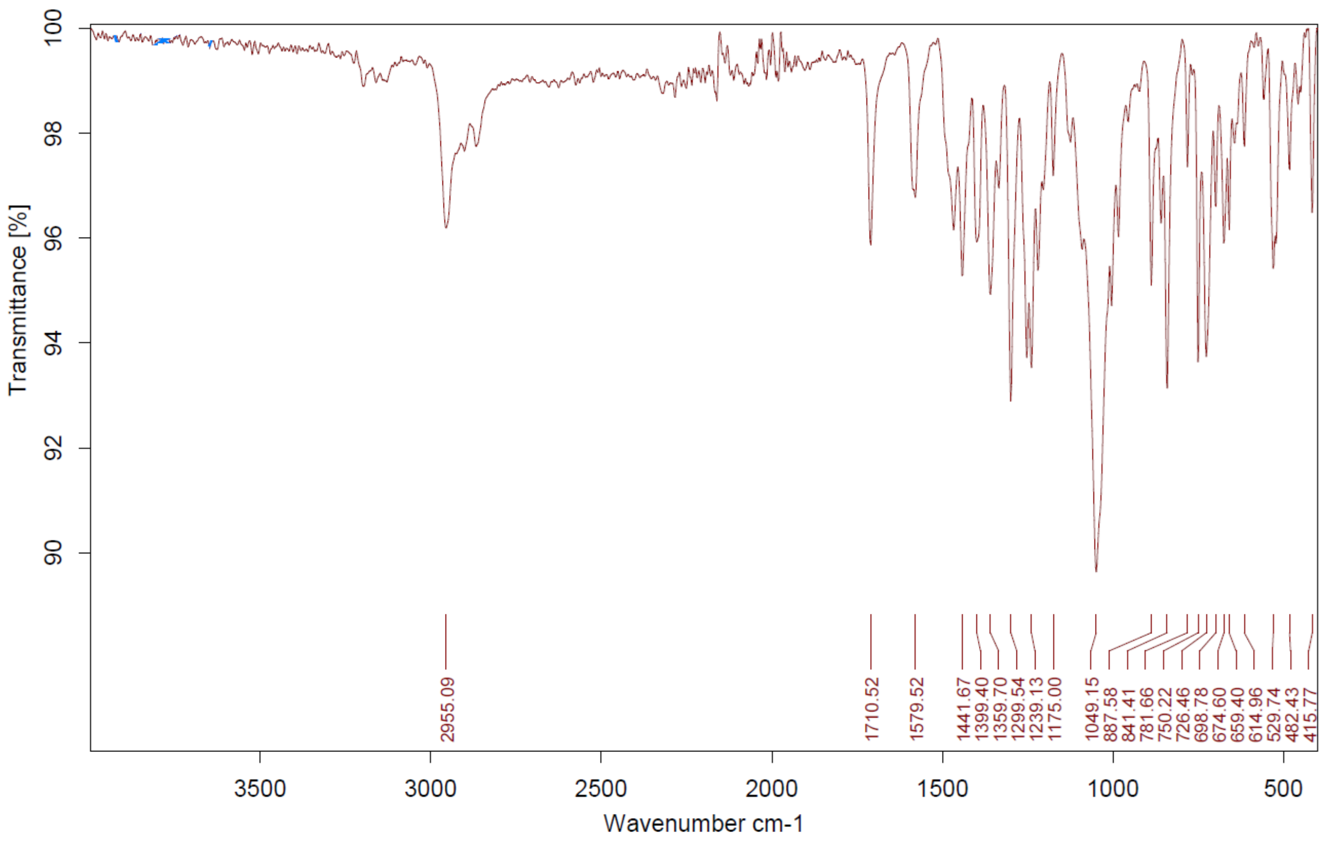

Figure S10: FT-IR (ATR) of ***[Cr(L^{NHC})$_2$][BF$_4$]*** *at 298 K.*

4. Mass spectrometry

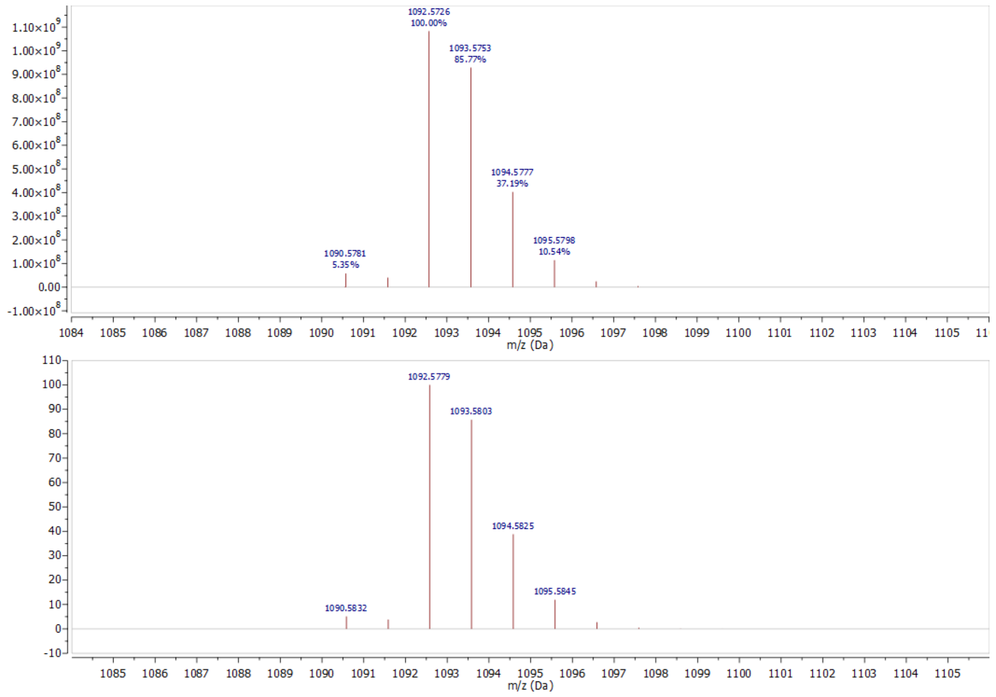

Figure S11: Experimental (top) and simulated (bottom) HR-ESI(+)-MS spectra of ***[Cr(L^{MIC})$_2$]$^+$***.

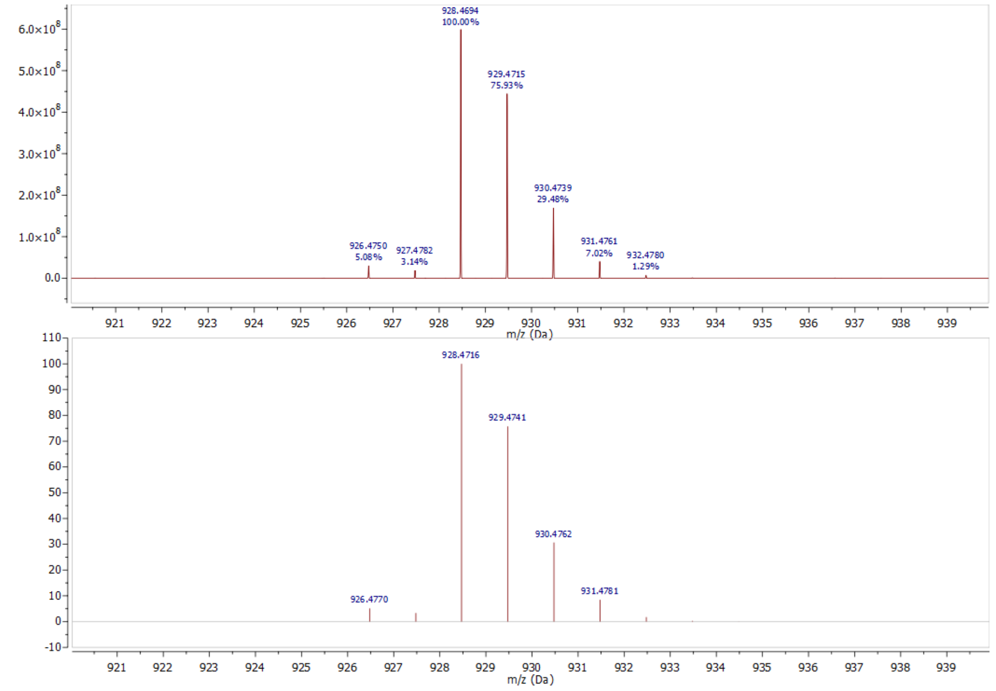

Figure S12: Experimental (top) and simulated (bottom) HR-ESI(+)-MS spectra of ***[Cr(L^{NHC})$_2$]$^+$****, from* ***[Cr(L^{NHC})$_2$]BF$_4$*** *sample.*

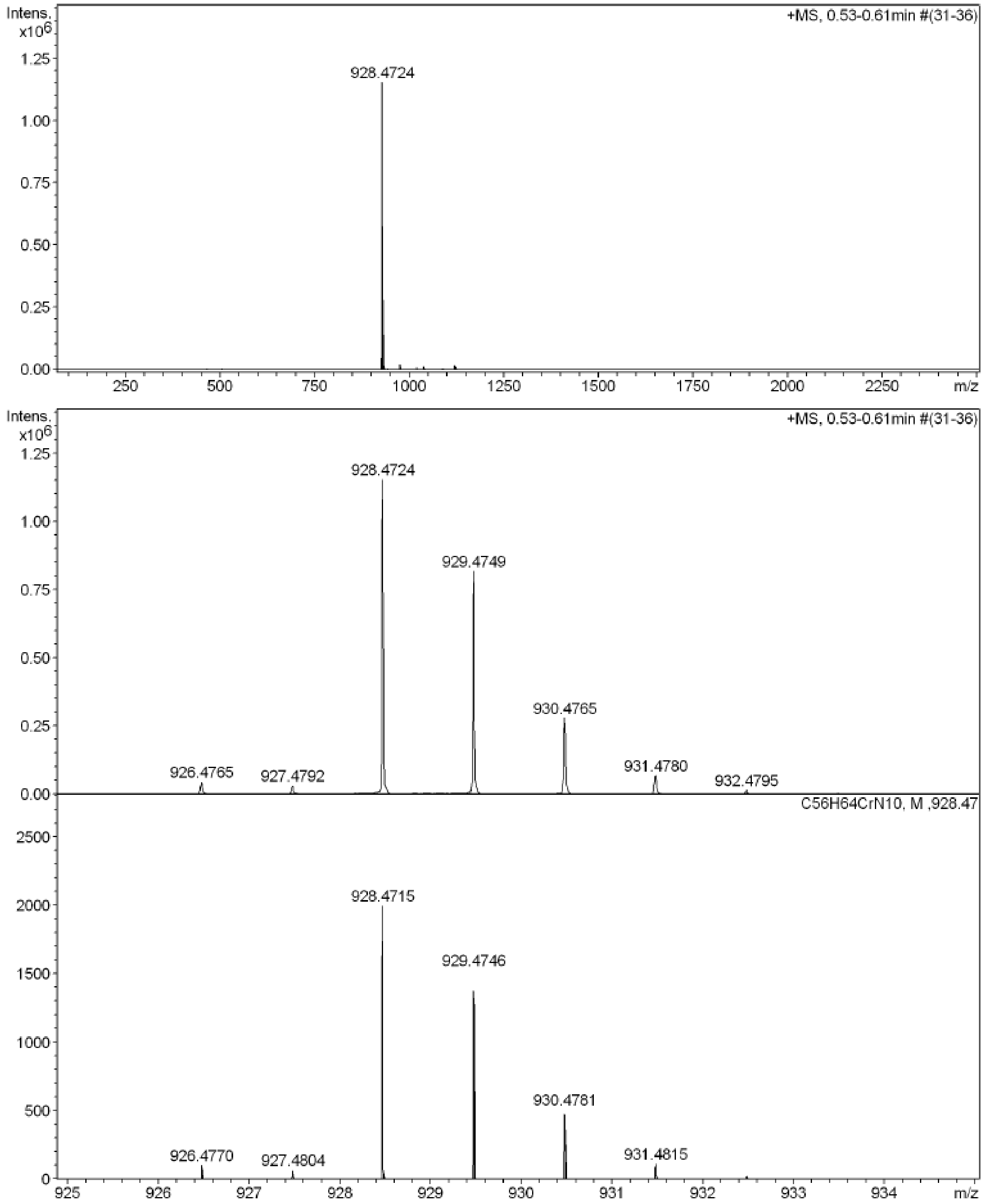

Figure S13: Experimental (top) and simulated (bottom) HR-ESI(+)-MS spectra of ***[Cr(L^{NHC})$_2$]$^+$****,from* ***[Cr(L^{NHC})$_2$]PF$_6$*** *sample.*

5. Cyclic Voltammetry

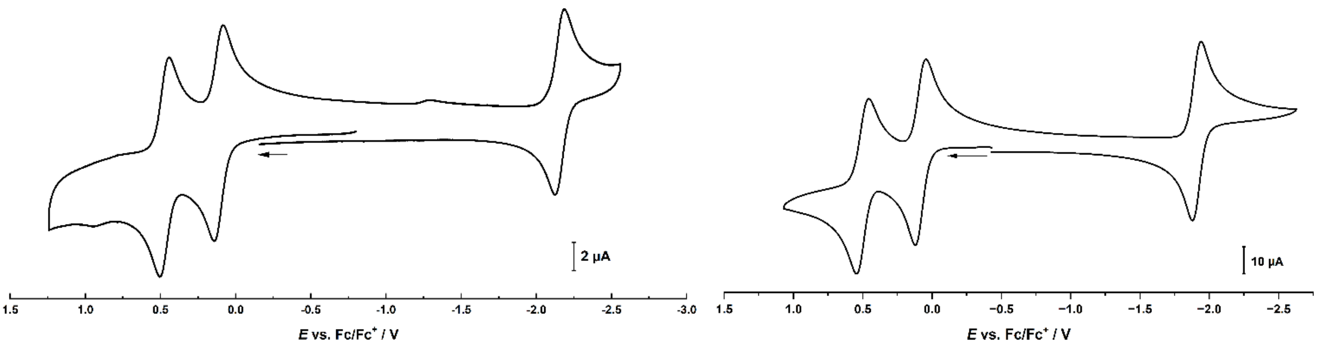

*Figure S14: Cyclic voltammogram of 1 mM **[Cr(L^{MIC})$_2$]BF$_4$** (left) and **[Cr(L^{NHC})$_2$]PF$_6$** (right) in dry and deaerated acetonitrile with 0.2 M NBu$_4$PF$_6$ as the supporting electrolyte measured against ferrocene as an internal standard. A glassy carbon disk was used as the working electrode, platinum wire was used as a counter electrode and a silver wire was used as a pseudo-reference electrode.*

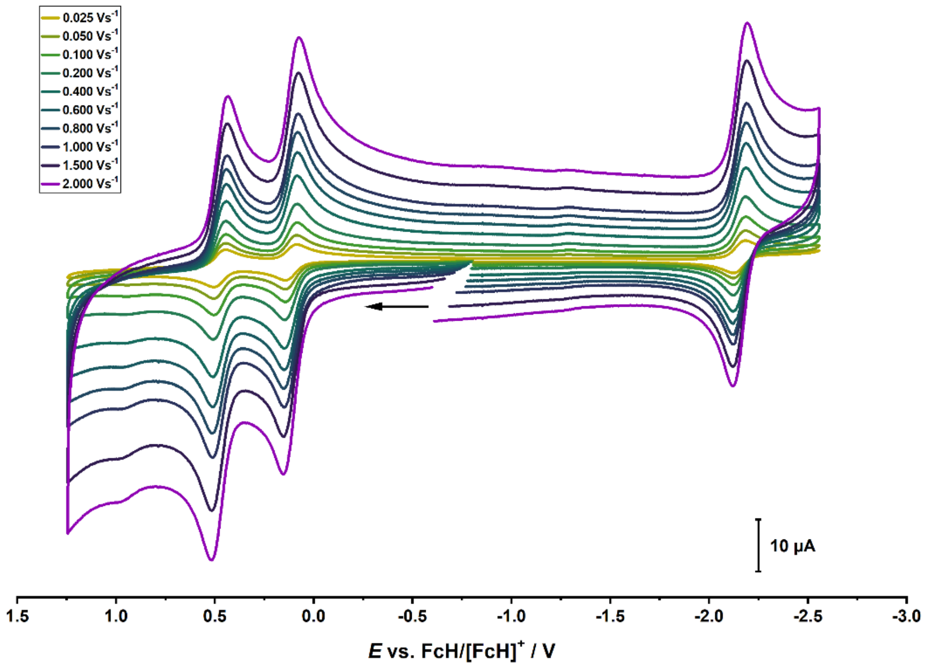

*Figure S15: Cyclic voltammogram of 1 mM **[Cr(L^{MIC})$_2$]BF$_4$** recorded at various scan rates between 25 – 2000 mV s^{-1} in 0.2 M NBu$_4$PF$_6$ in MeCN at 298 K.*

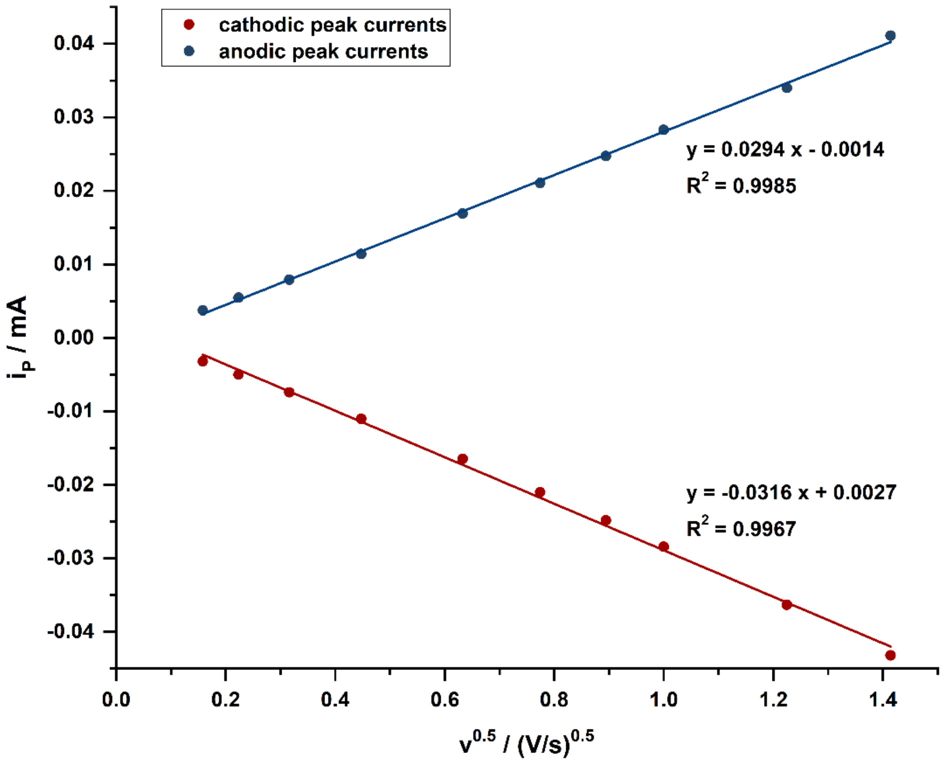

*Figure S16: Randles-Sevcik plot of the first oxidation process for **[Cr(L^{MIC})$_2$]BF$_4$**.*

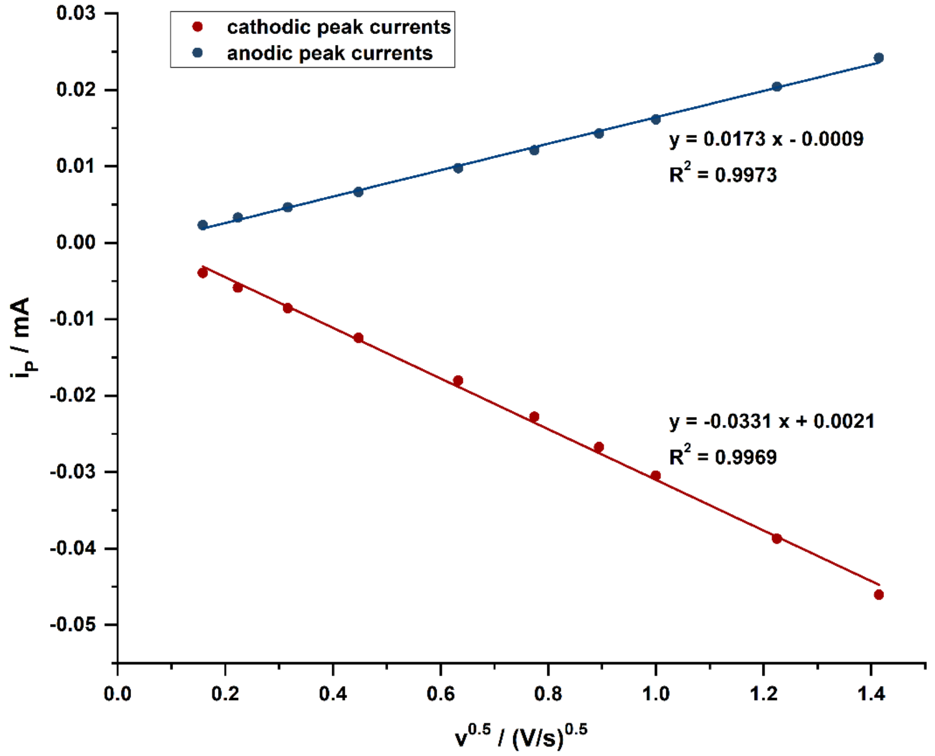

*Figure S17: Randles-Sevcik plot of the second oxidation process for **[Cr(L^{MIC})$_2$]BF$_4$**.*

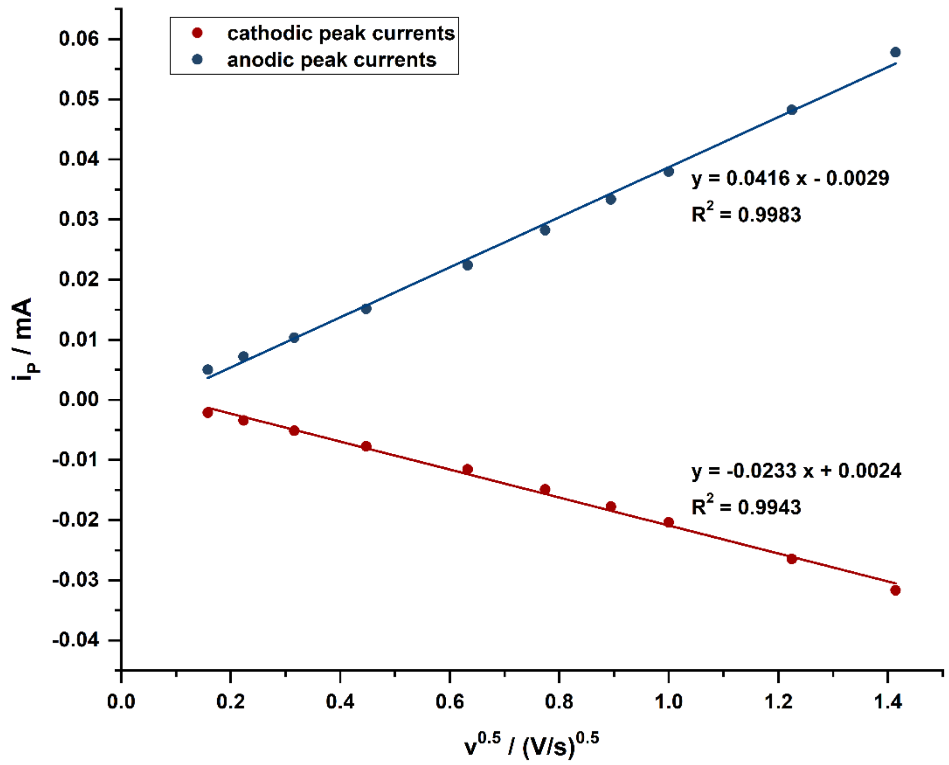

Figure S18: Randles-Sevcik plot of the first reduction process for ***[Cr(L^{MIC})$_2$]BF$_4$****.*

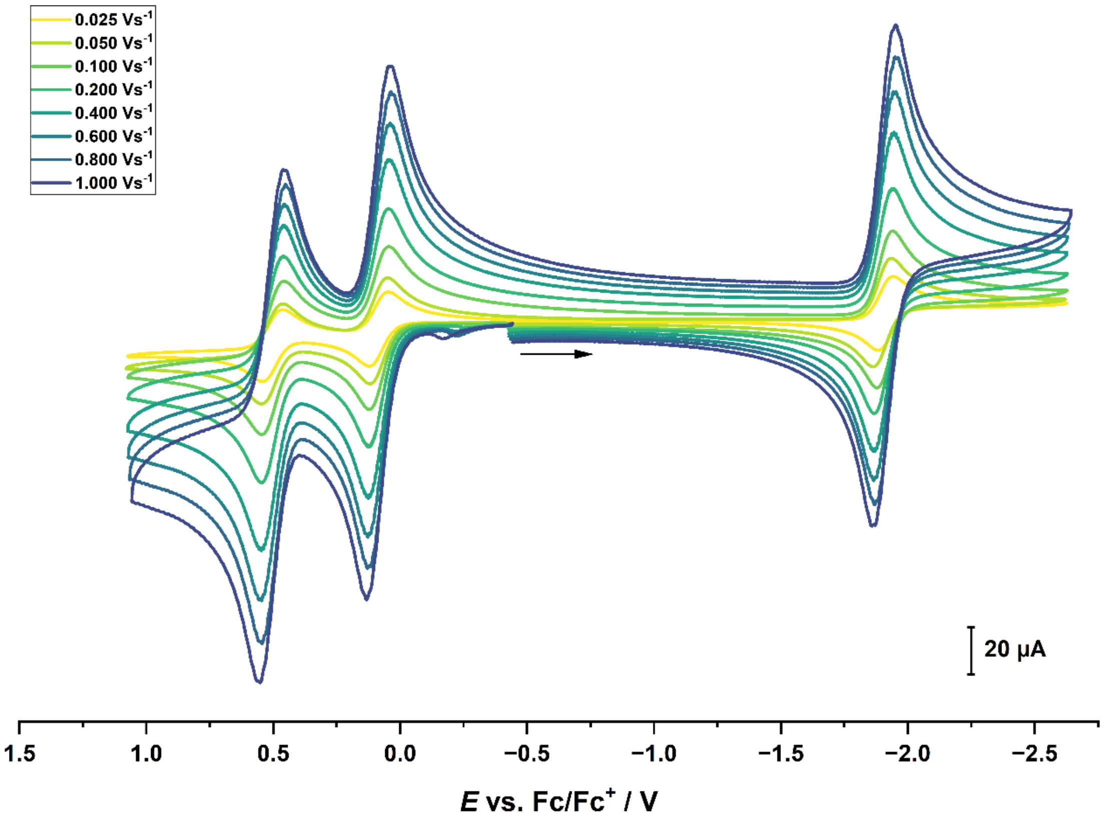

Figure S19: Cyclic voltammogram of 1 mM ***[Cr(L^{NHC})$_2$]BF$_4$*** *recorded at various scan rates between 25 – 2000 mV s^{-1} in 0.2 M NBu$_4$PF$_6$ in MeCN at 298 K.*

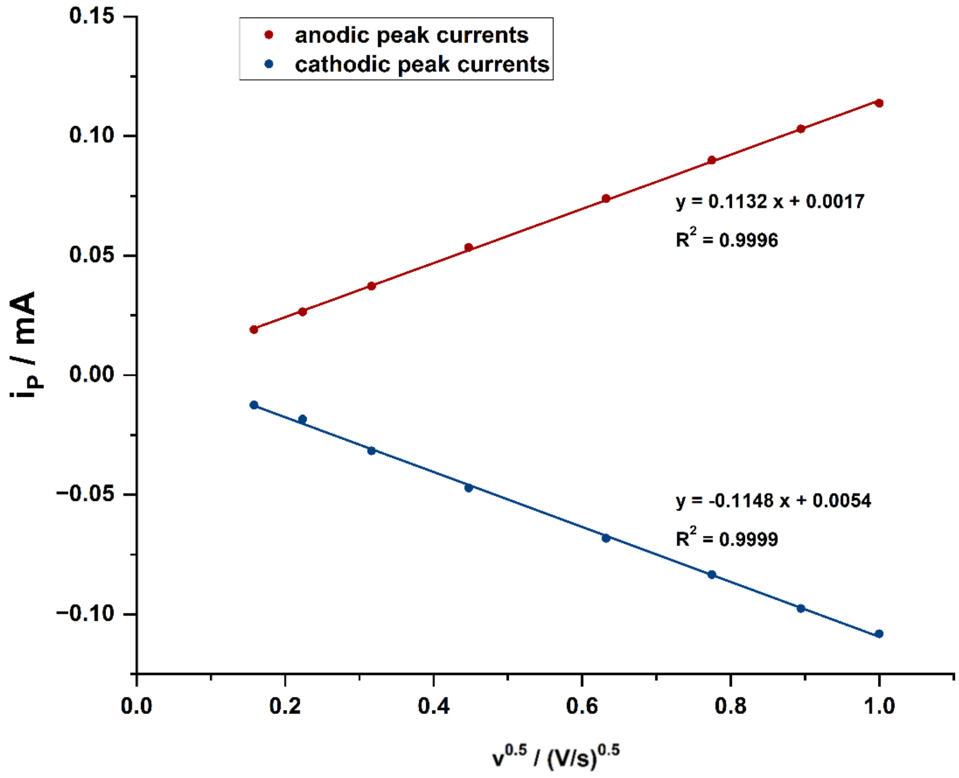

Figure S20: Randles-Sevcik plot of the first oxidation process for ***[Cr(L^{NHC})$_2$]BF$_4$****.*

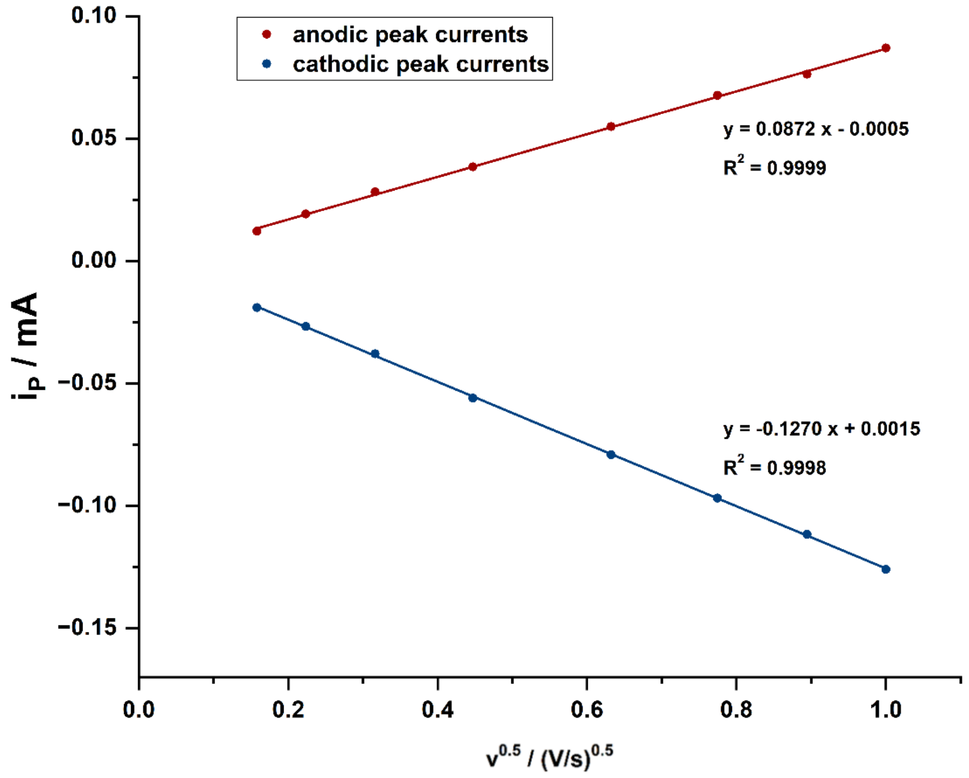

Figure S21: Randles-Sevcik plot of the first reduction process for ***[Cr(L^{NHC})$_2$]BF$_4$****.*

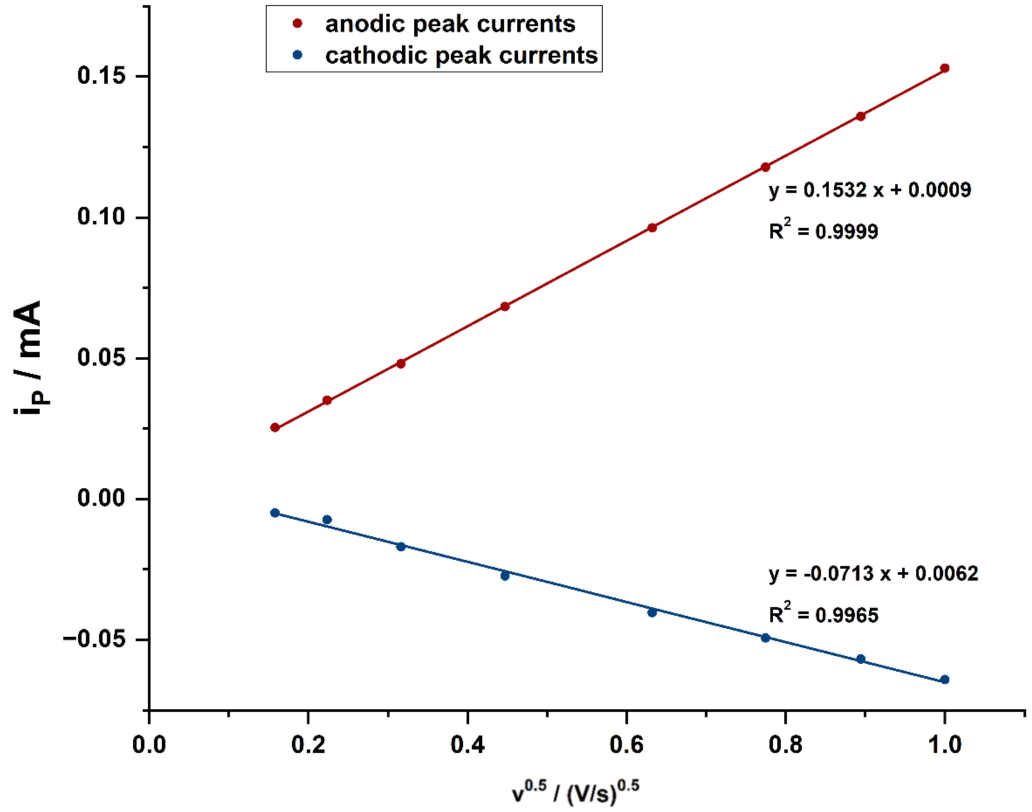

Figure S22: Randles-Sevcik plot of the second oxidation process for ***[Cr(L^{NHC})$_2$]BF$_4$****.*

6. Photophysical Properties of $[Cr(L^{MIC})_2]^+$

UV-vis transient absorption

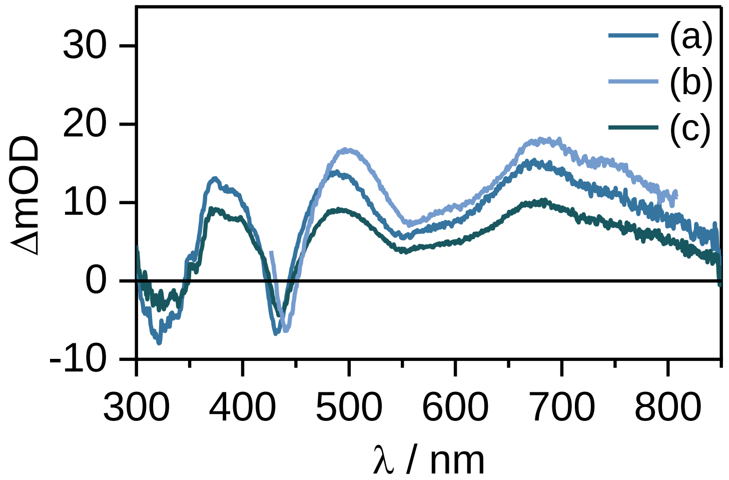

Figure S23: Transient UV-vis absorption spectra of 100 µM ***$[Cr(L^{MIC})_2]BF_4$*** *in deaerated acetonitrile at 293 K, recorded following excitation at:* ***a.*** *355 nm (~100 mJ per pulse) and* ***c.*** *532 nm (~ 70 mJ per pulse) with nanosecond pulses (pulse duration ~10 ns), time-integrated over 200 ns;* ***b.*** *430 nm (6 mJ per pulse) with picosecond pulses (pulse duration ~30 ps), time-integrated over 20 ns.*

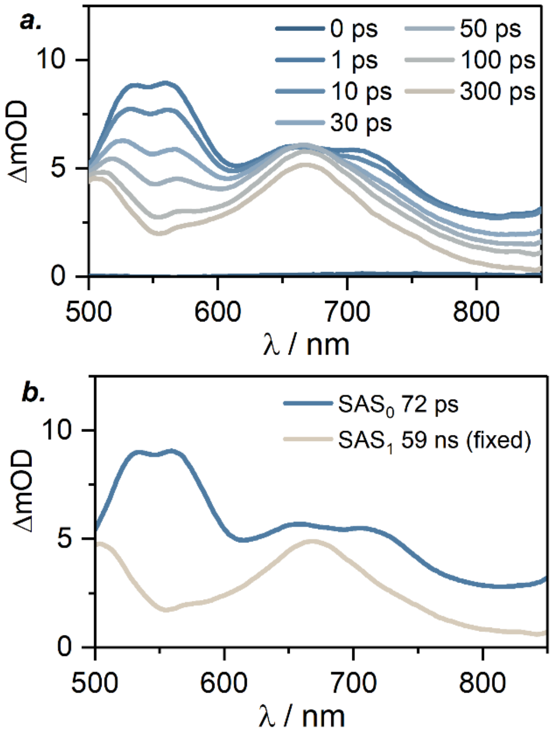

Figure S24: ***a.*** *UV-vis transient absorption spectra of* ***$[Cr(L^{MIC})_2]^+$*** *in acetonitrile at 293 K, recorded at different delay times (shown in the inset) following excitation at 430 nm with femtosecond pulses;* ***b.*** *result of the global fit analysis. For the fitting a sequential excited state population model was used. Obtained species-associated spectra SAS0-1 and corresponding excited state lifetimes are indicated in the inset.*

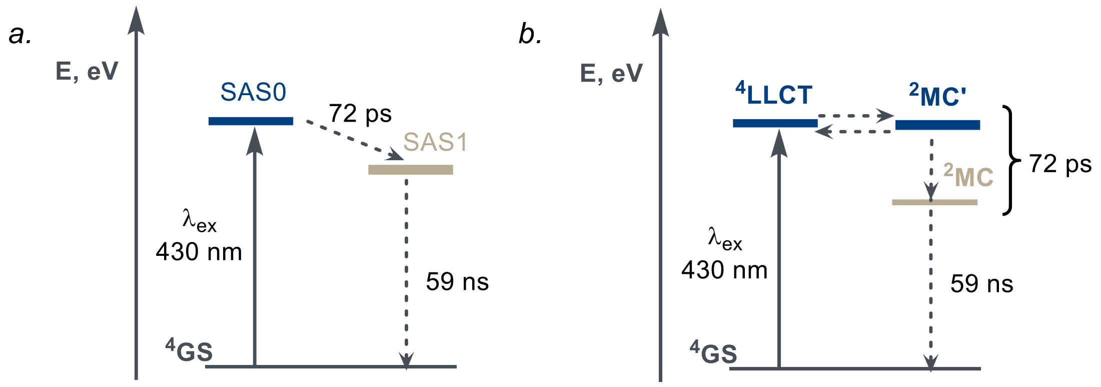

Figure S25: ***a.*** *Scheme of the global fit analysis sequential excited state population model for* ***[Cr(L^{MIC})$_2$]$^+$****, SAS0 was assumed to be equilibrated ^{4}CT and higher-lying ^{2}MC state, SAS1 – lowest ^{2}MC state.* ***b.*** *Schematic excited state relaxation model proposed based on the global fit model in a. with excited state character assignment supported by spectro-electrochemistry data in Figure S24 and calculations (Table S15-16).*

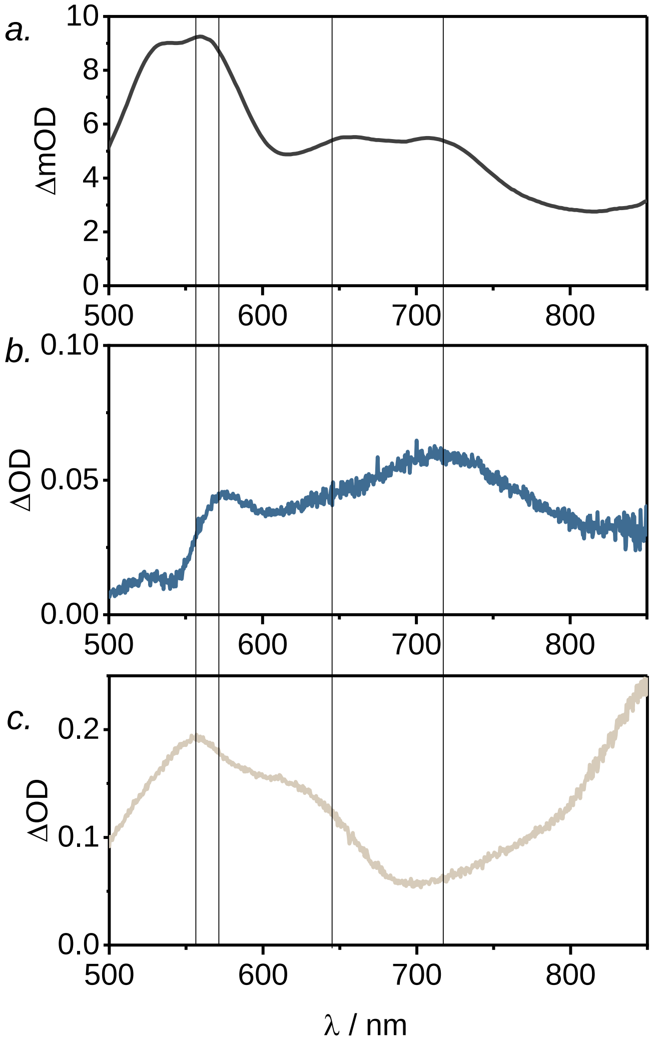

Figure S26: ***a.*** *UV-vis transient absorption spectra of* ***[Cr(L^{MIC})$_2$]$^+$*** *in acetonitrile at 293 K, recorded at different delay time of 1 ps following excitation at 430 nm with femtosecond pulses;* ***b.*** *UV-vis differential (ground-state) absorption spectrum of [Cr(L^{MIC})$_2$]$^+$ in deaerated CH_3CN at room temperature with −1.9 V vs SCE potential applied, corresponding to a ligand-based L^0/L$^-$ reduction process;* ***c.*** *UV-vis differential (ground-state) absorption spectrum of [Cr(L^{MIC})$_2$]$^+$ in deaerated CH_3CN at room temperature with 0.5 V vs SCE potential applied, corresponding to a ligand-based L^0/L$^+$ oxidation process.*

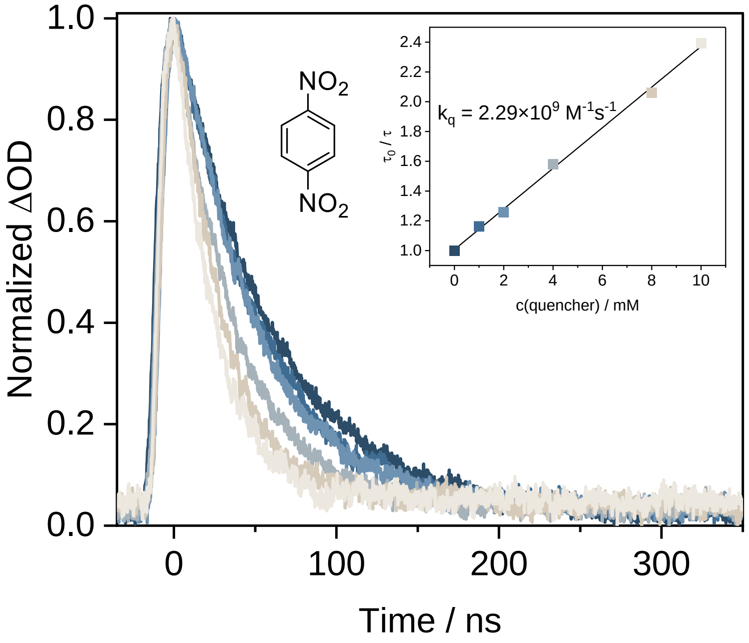

Figure S27: Main plot: Decay of ***[Cr(L^{MIC})$_2$]$^+$*** *(30 µM) excited state absorption band at 700 nm in the presence of increasing concentrations of 1,4-dinitrobenzene after excitation at 532 nm with nanosecond pulsed-laser (~70 mJ per pulse). Inset: Stern-Volmer plot (color-coding corresponds to the kinetic plots).*

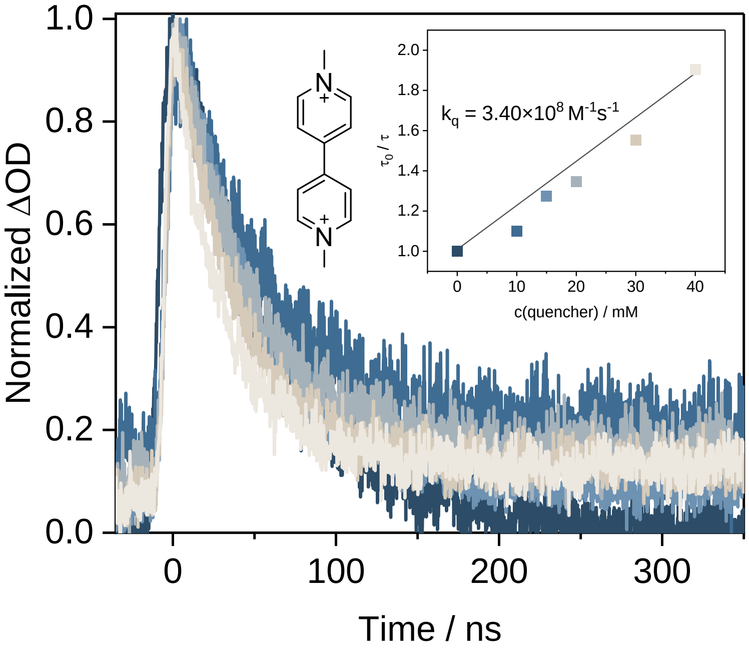

Figure S28: Main plot: Decay of ***[Cr(L^{MIC})$_2$]$^+$*** *(30 µM) excited state absorption band at 700 nm in the presence of increasing concentrations of methyl viologen hexafluorophosphate after excitation at 532 nm with nanosecond pulsed-laser (~70 mJ per pulse). Inset: Stern-Volmer plot (color-coding corresponds to the kinetic plots).*

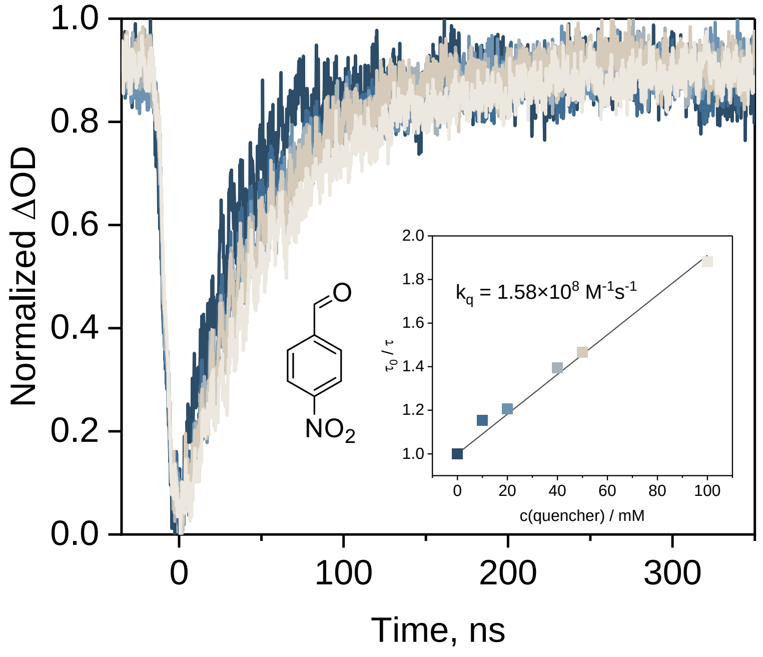

*Figure S29: Main plot: Recovery of **[Cr(L^{MIC})$_2$]$^+$** (30 µM) ground state bleach at 434 nm in the presence of increasing concentrations of 4-nitrobenzaldehyde after excitation at 532 nm with nanosecond pulsed-laser (~70 mJ per pulse). Inset: Stern-Volmer plot (color-coding corresponds to the kinetic plots).*

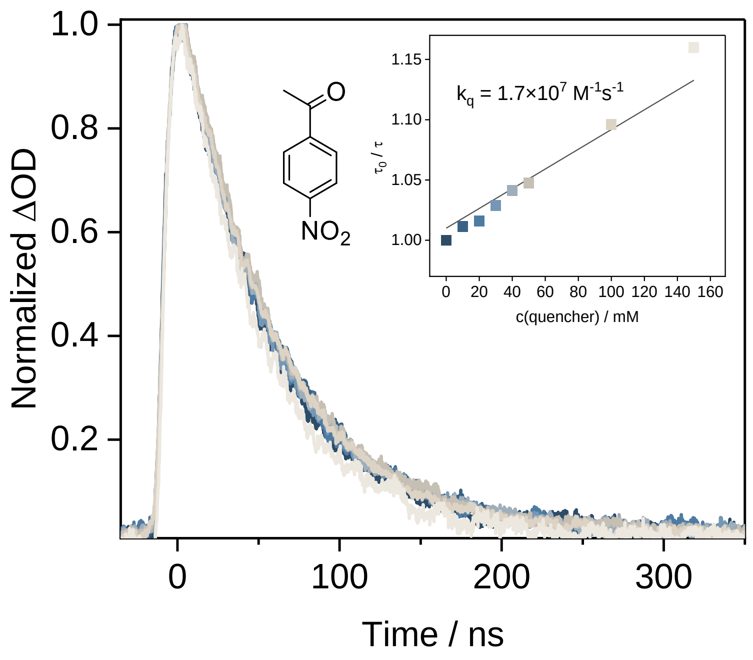

*Figure S30: Main plot: Decay of **[Cr(L^{MIC})$_2$]$^+$** (30 µM) excited state absorption band at 700 nm in the presence of increasing concentrations of 4-nitroacetophenone after excitation at 532 nm with nanosecond pulsed-laser (~70 mJ per pulse). Inset: Stern-Volmer plot (color-coding corresponds to the kinetic plots).*

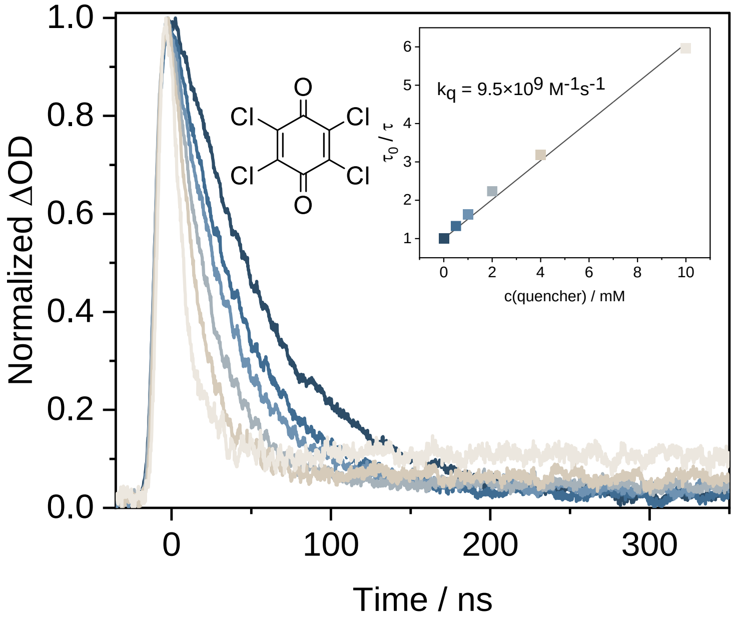

Figure S31: Main plot: Decay of ***[Cr(L^{MIC})$_2$]$^+$*** *(30 µM) excited state absorption band at 700 nm in the presence of increasing concentrations of tetrachloro-1,4-benzoquinone after excitation at 532 nm with nanosecond pulsed-laser (~70 mJ per pulse). Inset: Stern-Volmer plot (color-coding corresponds to the kinetic plots). The residual ΔOD signal after 50 ns at quencher concentrations exceeding 4 mM is attributed to photodegradation of tetrachloro-1,4-benzoquinone.*

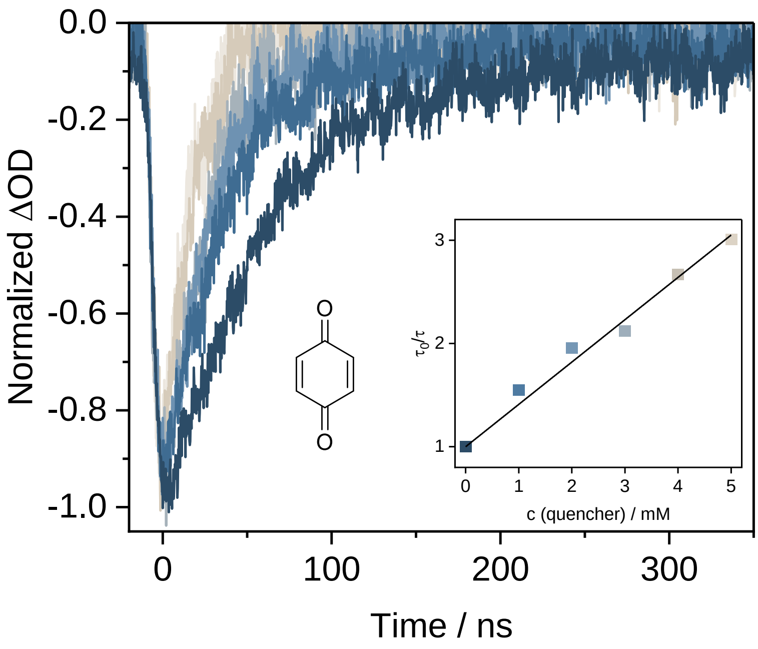

Figure S32: Main plot: Recovery of ***[Cr(L^{MIC})$_2$]$^+$*** *(30 µM) ground state bleach at 434 nm in the presence of increasing concentrations of 1,4-benzoquinone after excitation at 532 nm with nanosecond pulsed-laser (~70 mJ per pulse). Inset: Stern-Volmer plot (color-coding corresponds to the kinetic plots).*

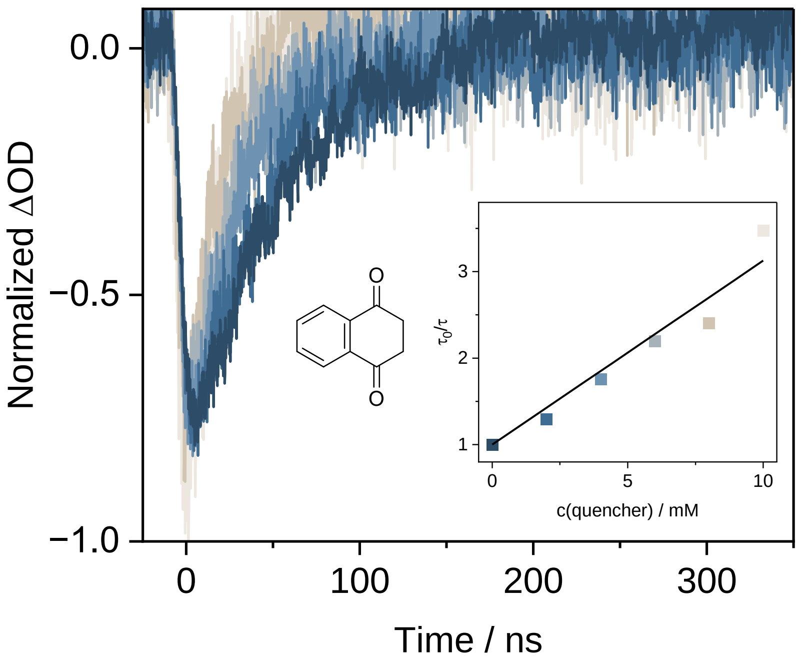

Figure S33: Main plot: Recovery of ***[Cr(L^{MIC})$_2$]$^+$*** *(30 µM) ground state bleach at 434 nm in the presence of increasing concentrations of 1,4-naphthoquinone after excitation at 532 nm with nanosecond pulsed-laser (~70 mJ per pulse). Inset: Stern-Volmer plot (color-coding corresponds to the kinetic plots).*

Alternative Rehm-Weller fitting analysis

It is important to note that in the Rehm-Weller equation (Eq. S3) the pre-exponential factor, which we fixed as constant of 0.25 in Eq.3 for fitting simplicity (Figure 10), is in fact a function of the dissociation rate constant of the donor-acceptor encounter complex back to the initial reactants (k_{d-1}). The value of 0.25 in Eq.3 was originally assumed by Rehm and Weller for an onward reaction with $k_d = 2\cdot10^{10}$ $M^{-1}s^{-1}$.[11] Clearly, in our case that value would change together with k_d and k_{d-1}. To account for this, advanced data fitting included treating the pre-exponential factor as a function of k_d in Eq. S3, compared to Eq. 3 in the main paper. Parameter *a* in Eq. S3 includes the dissociation rate constant of the donor-acceptor encounter complex back to the initial reactants (k_{d-1}) and a constant related to the dielectric relaxation time of the solvent (k^0). See Figure 10 for the fit results comparison. According to the fitting results (Figure S34), we obtained a lower k_q value of $1.17\cdot10^{10}$ $M^{-1}s^{-1}$, compared to the $1.49\cdot10^{10}$ $M^{-1}s^{-1}$ from the simplified fit. We also observed a higher $\Delta G_{ET}^{\ddagger}(0)$ of 0.16 eV, as opposed to 0.13 eV. However, ultimately, that approach gave a similar $E^0(D^{+*}/D^{2+})$ value of –0.84 V.

$$E_0(D^{+*} / D^{2+}) = E_0(D^{+} / D^{2+}) - E_{00} / e \quad (\mathbf{S1})$$

$$\Delta G_{ET} = [E^0(D^{+*} / D^{2+}) - E^0(A / A^{\bullet-})] \times e \quad (\mathbf{S2})$$

$$k_q = \frac{k_d}{1 + a \cdot [\exp(\Delta G^{\ddagger}_{ET}/k_bT) + \exp((\Delta G_{ET}/k_bT)]} \quad \text{where: } a = k_{d-1}/k^0 \quad (\mathbf{S3})$$

$$\Delta G^{\ddagger}_{ET} = \left[(\Delta G_{ET}/2)^2 + \Delta G^{\ddagger}_{ET}(0)^2\right]^{1/2} + \Delta G_{ET}/2 \quad (\mathbf{S4})$$

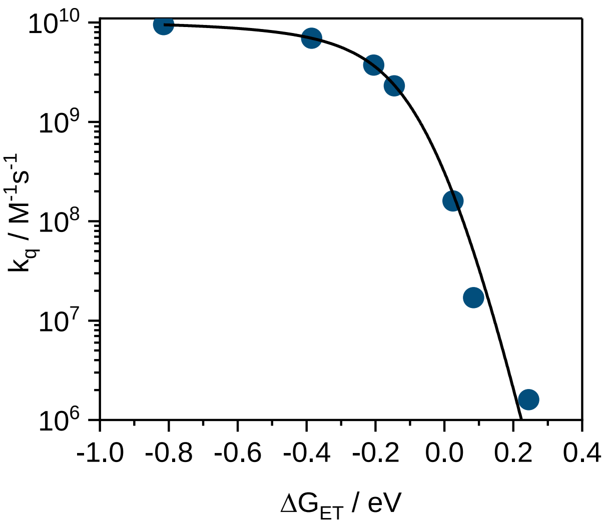

Figure S34: Rehm-Weller plot, showing a dependency between the bimolecular electron transfer rate constants (k_q) for ***[Cr(L^{MIC})$_2$]$^+$*** *with selected electron acceptors and free energy (ΔG_{ET}). Best fit for $E^0(D^{+*}/D^{2+})$ = −0.84 V; $k_d = (1.17 \pm 0.03)\cdot10^{10}$ $M^{-1}s^{-1}$; $\Delta G_{ET}^{\ddagger}(0) = 0.15 \pm 0.01$ eV; $a = (1.6 \pm 0.3)\cdot10^{11}$ $M^{-1}s^{-1}$.*

7. Photophysical Properties of $[Cr(L^{NHC})_2]^+$

Ground state UV-vis absorption

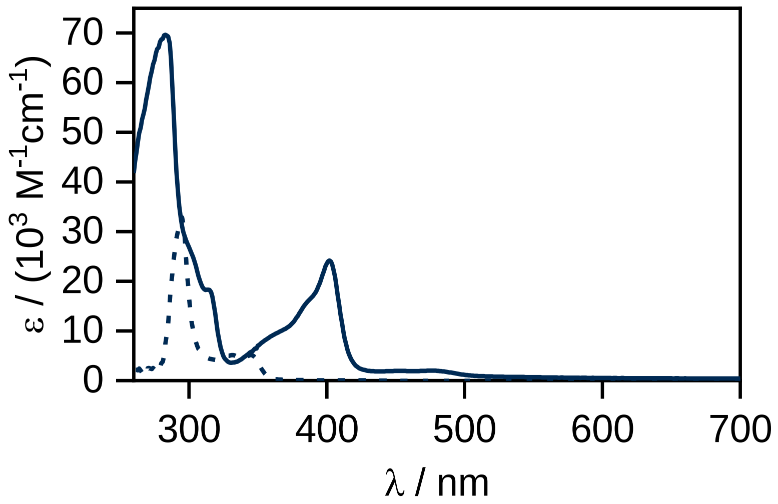

Figure S35: UV-vis absorption of ***[Cr(L^{NHC})$_2$]$^+$*** *(solid line) and* ***[H$_3$(L^{NHC})$_2$]$^{2+}$*** *pro-ligand (dashed line) in acetonitrile at 293 K.*

UV-vis transient absorption

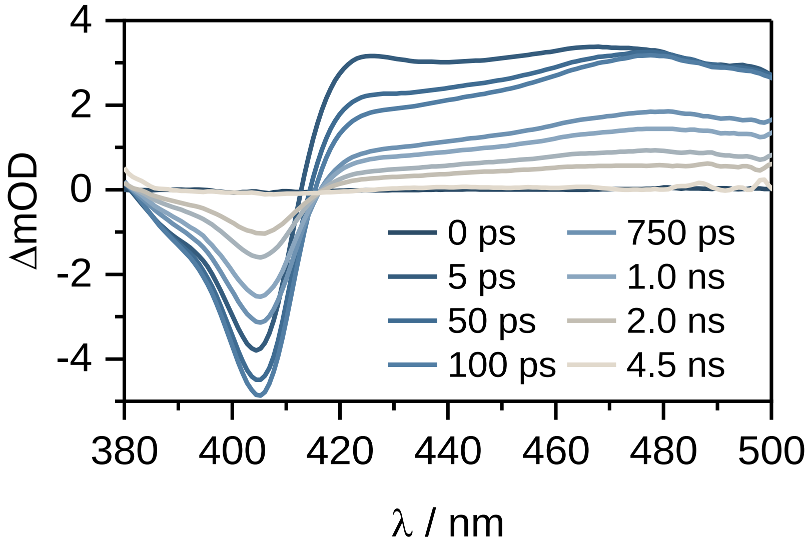

Figure S36: UV-Vis transient absorption spectra of ***[Cr(L^{NHC})$_2$]$^+$*** *in acetonitrile at 293 K, recorded at different delay times (shown in the inset) following excitation at 355 nm with femtosecond pulses.*

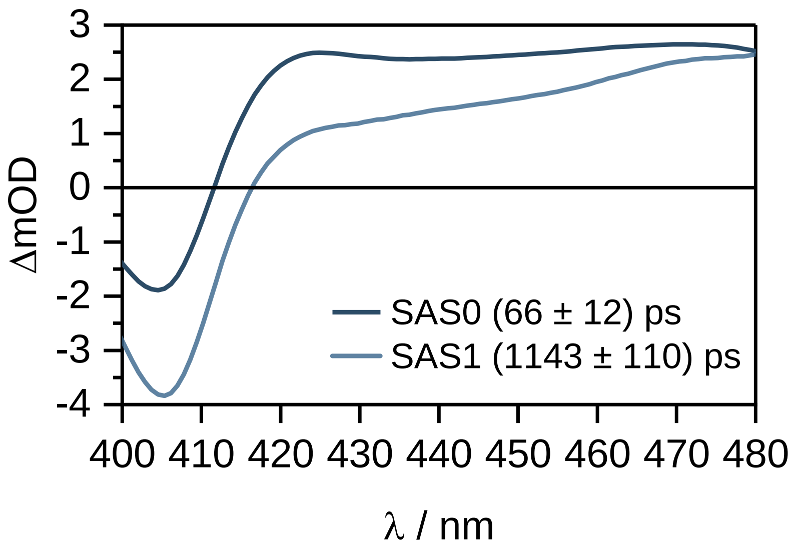

Figure S37: Result of the global fit analysis of the UV-Vis transient absorption spectra for ***[Cr(L^{NHC})$_2$]$^+$*** *(see Figure S36). For the fitting a sequential excited state population model was used. Obtained SAS and corresponding lifetimes are indicated in the inset.*

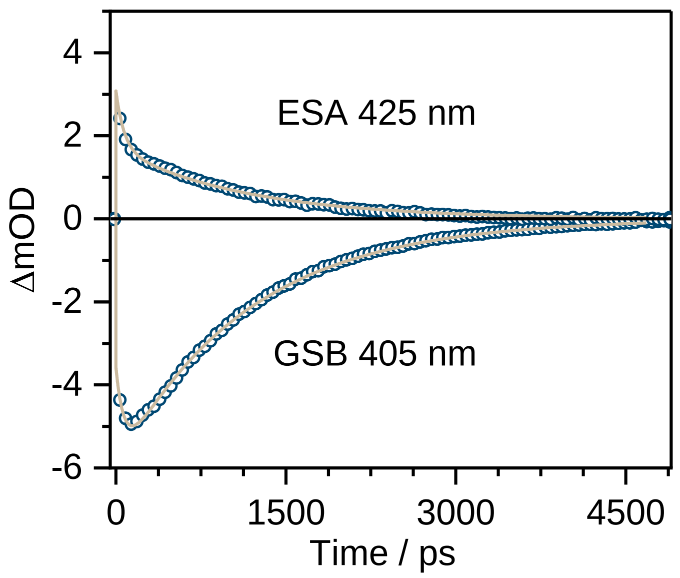

Figure S38: Decay of the ESA signal at 425 nm and the GSB at 405 nm for ***[Cr(L^{NHC})$_2$]$^+$*** *in acetonitrile at 293 K following excitation at 355 nm with femtosecond pulses. The experimental data are depicted by circles, while the fits are represented by lines.*

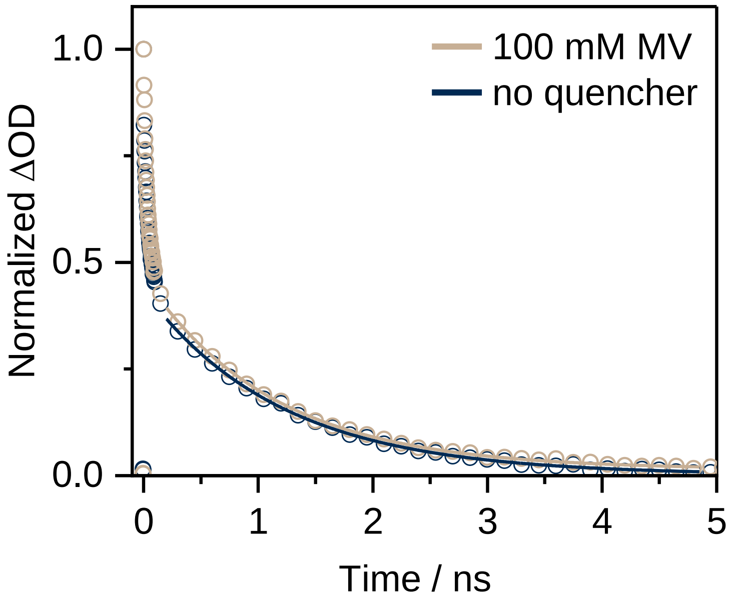

Figure S39: Decay of the ESA signal at 425 nm of ***[Cr(L^{NHC})$_2$]$^+$*** *in acetonitrile at 293 K in the absence of the quencher (blue) and presence of 100 mM of methyl viologen (brown) after the excitation at 355 nm with femtosecond pulses. The experimental data are depicted by circles, while the fits are represented by lines.*

8. Photophysical Properties of $[Cr(L^{py})_2]^+$

Ground state UV-vis absorption

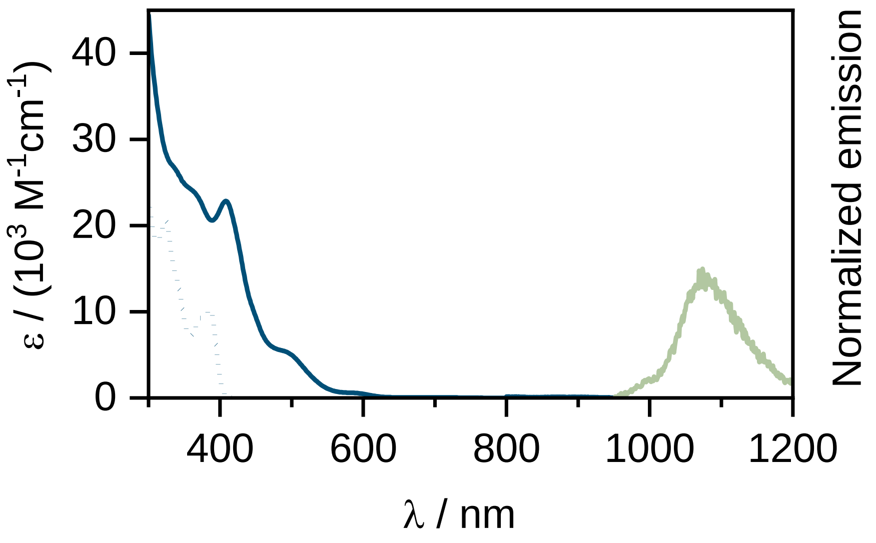

Figure S40: UV-vis absorption of ***[Cr(L^py)_2]^+*** *(blue solid line) and* ***HL^py*** *pro-ligand (dashed line) in acetonitrile; emission of* ***[Cr(L^py)_2]^+*** *(green solid line) in 2-methyl tetrahydrofuran at 77 K.*

UV-Vis transient absorption

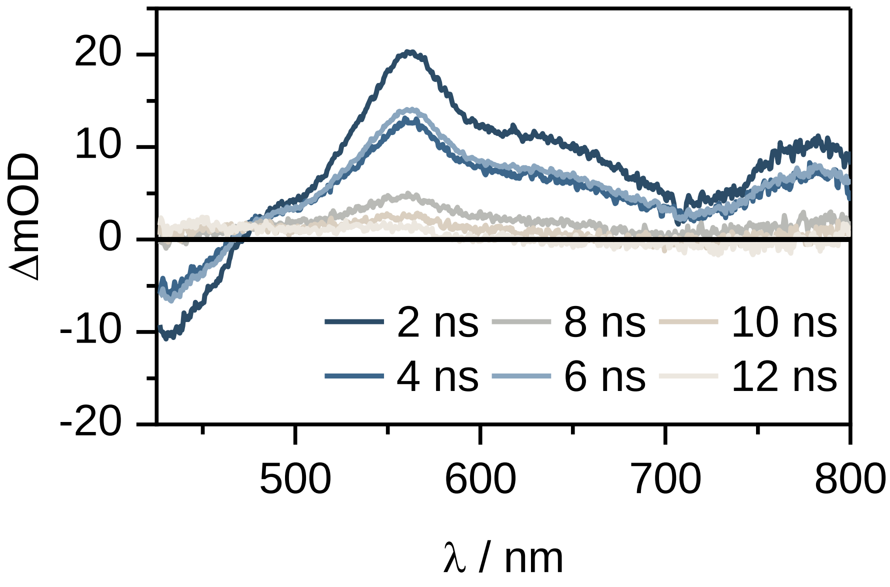

Figure S41: UV-Vis transient absorption spectra of ***[Cr(L^py)_2]^+*** *in deaerated acetonitrile at 293 K, recorded at different delay times (shown in the inset) following excitation at 355 nm with picosecond pulses.*

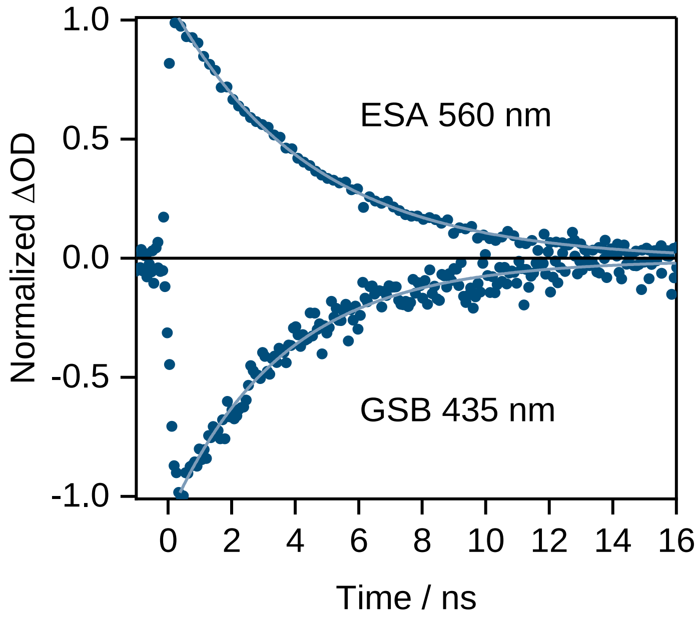

Figure S42: Decay of the ESA at 560 nm and recovery of the GSB at 435 nm after excitation of ***[Cr(L^{py})$_2$]$^+$*** *with picosecond pulses at 355 nm. A monoexponential fitting for both data sets provides a time constant of (4.4 ± 0.1) ns. The experimental data are depicted by circles, while the fits are represented by lines.*

9. Dependence of the non-radiative deactivation rate constant on the experimental and theoretical/calculated energy gaps

Table S1. Dependence of the non-radiative deactivation rate constant (k_{nr}) on the experimental and theoretical/calculated energy gaps (ΔE) between key excited states (^{4}MC-^{2}MC and ^{4}CT-^{2}MC) in [Cr(L^{MIC})$_2$]$^+$, [Cr(L^{NHC})$_2$]$^+$, [Cr(L^{py})$_2$]$^+$ complexes.

Complex	$\ln k_{nr}$	ΔE_{exp}(^{4}MC-^{2}MC) / eV	ΔE_{theor}(^{4}MC-^{2}MC) / eV	ΔE_{exp}(^{4}CT-^{2}MC) / eV	ΔE_{theor}(^{4}CT-^{2}MC) / eV
**[Cr(L^{MIC})$_2$]$^+$**	16.64	1.68	1.33	1.01	0.66
**[Cr(L^{NHC})$_2$]$^+$**	20.62	n/a	1.50	n/a	0.36
**[Cr(L^{py})$_2$]$^+$**	19.25	1.01	0.37	1.09	0.45

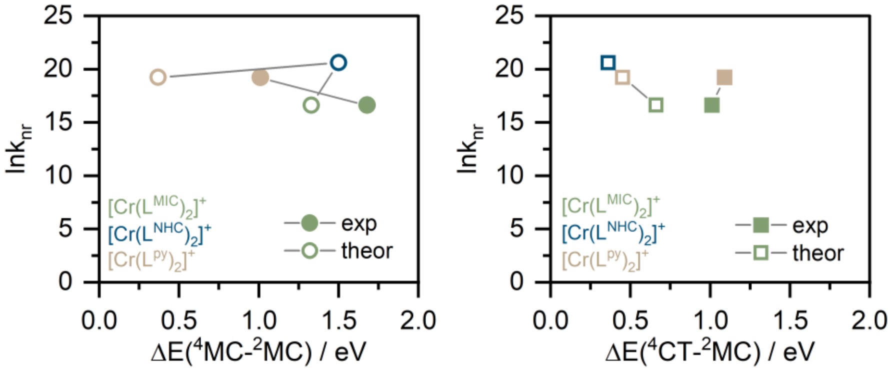

Figure S43. Data of Table S1 illustrated as plot of logarithmic non-radiative deactivation rate constant against energy gap between ^{4}MC-^{2}MC excited states (left) and ^{4}CT-^{2}MC excited states (right) in [Cr(L^{MIC})$_2$]$^+$, [Cr(L^{NHC})$_2$]$^+$, [Cr(L^{py})$_2$]$^+$ complexes, see legend for color coding.

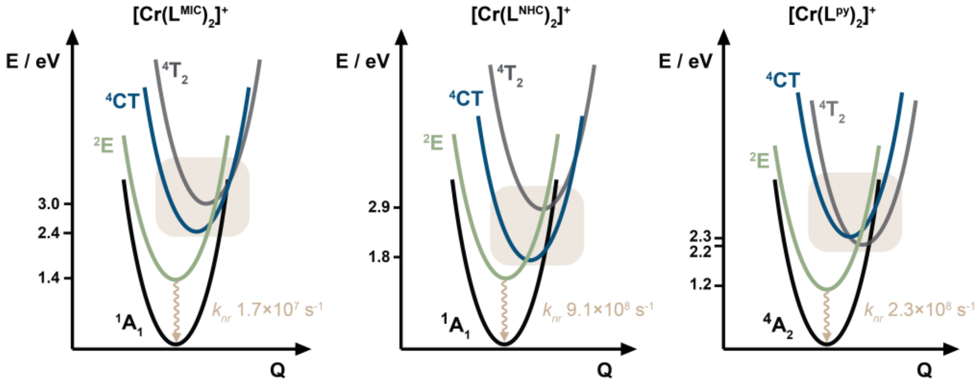

Figure S44. Proposed excited-state ordering in [Cr(L^{MIC})$_2$]$^+$, [Cr(L^{NHC})$_2$]$^+$, [Cr(L^{py})$_2$]$^+$ complexes depicted with simplified potential well diagram based on the combination of experimental and computational data (for latter see Computational Details).

10. Photoredox catalysis with $[Cr(L^{MIC})_2]^+$

Photocatalytic C-H arylation of furan with 4-methoxyphenyl diazonium tetrafluoroborate

The procedure was adapted from a previously published method.[12] 1,3,5-Trimethoxybenzene was used as internal standard. In an NMR tube 4-methoxyphenyl diazonium tetrafluoroborate (15 mg, 67.6 μmol, 1 eq.), **$[Cr(L^{MIC})_2]BF_4$** (1 mol.%), 1,3,5-trimethoxybenzene (11.4 mg, 67.6 μmol, 1 eq.) and furan (41.9 μL, 676 μmol, 10 eq.) were dissolved in 0.6 mL of dry DMSO-d_6, then the solution was deaerated. The sample was continuously irradiated with a 520 nm Kessil LED lamp for 3 hours, ^{1}H NMR spectra were measured every 30 min to monitor the reaction progress. A yield of 66% and conversion of >95% were determined relative to the internal standard.

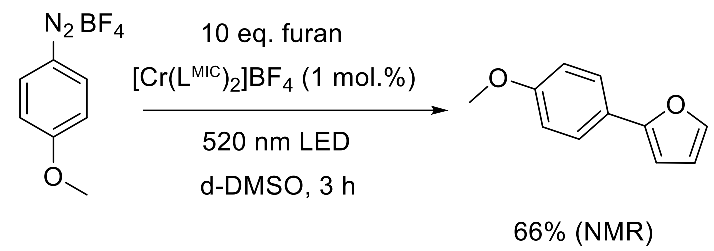

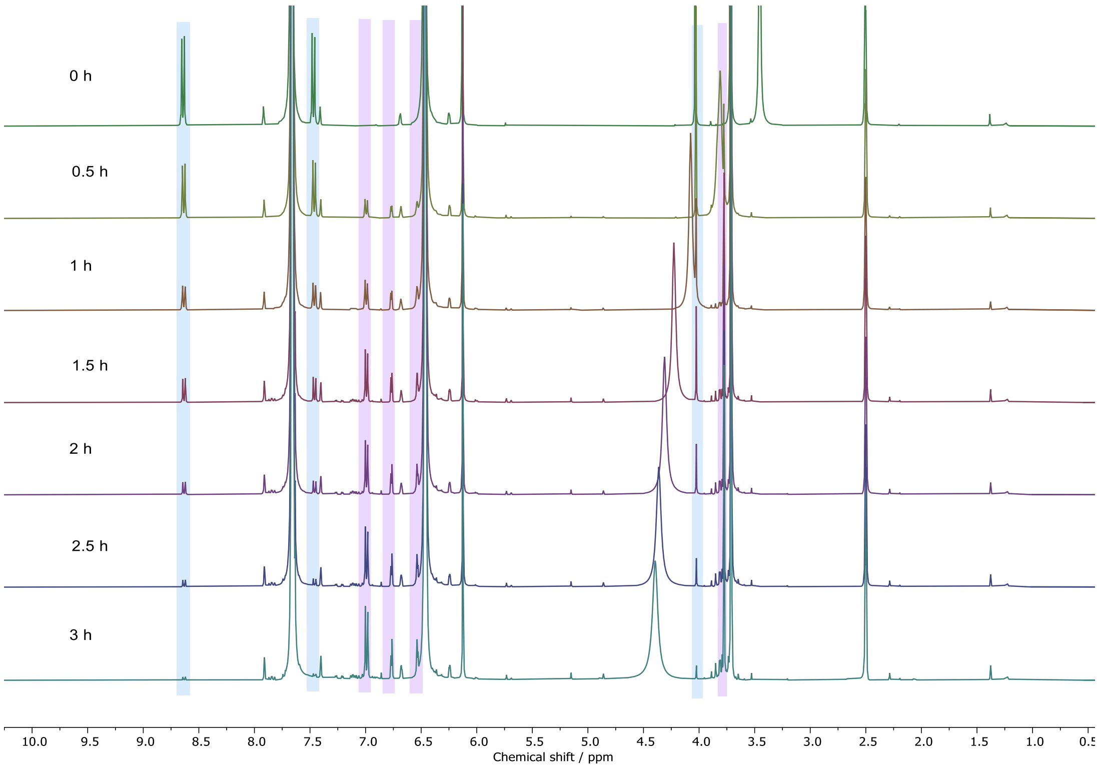

Figure S45: The C-H arylation of furan with 4-methoxyphenyl diazonium tetrafluoroborate was monitored via ^{1}H-NMR spectroscopy over the indicated irradiation periods. Signals corresponding to the starting material and product are highlighted in blue and purple, respectively.

A control experiment was performed to assess the photocatalytic activity of **[Cr(L^{MIC})$_2$]BF$_4$**. The same experiment as described above was repeated in the absence of the photocatalyst. The experiment revealed a product yield of 18% and a starting material conversion of 28%.

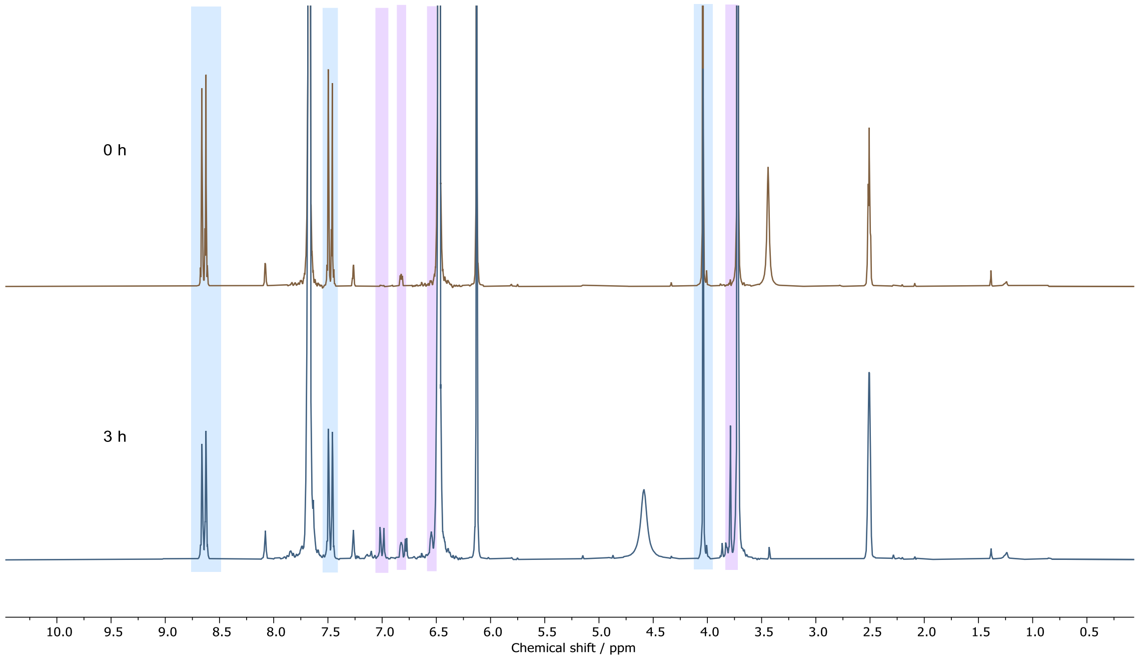

Figure S46: Control experiment in the absence of the photocatalyst. ^{1}H-NMR spectra measured after the indicated irradiation periods. Signals corresponding to the starting material and photoproduct are highlighted in blue and purple, respectively.

A control experiment was performed to assess the photocatalytic activity of **[Cr(L^{MIC})$_2$]BF$_4$**. The same experiment as described above was repeated in the presence of the photocatalyst and in the absence of light. The experiment revealed a product yield of 4.7% and a starting material conversion of 11%.

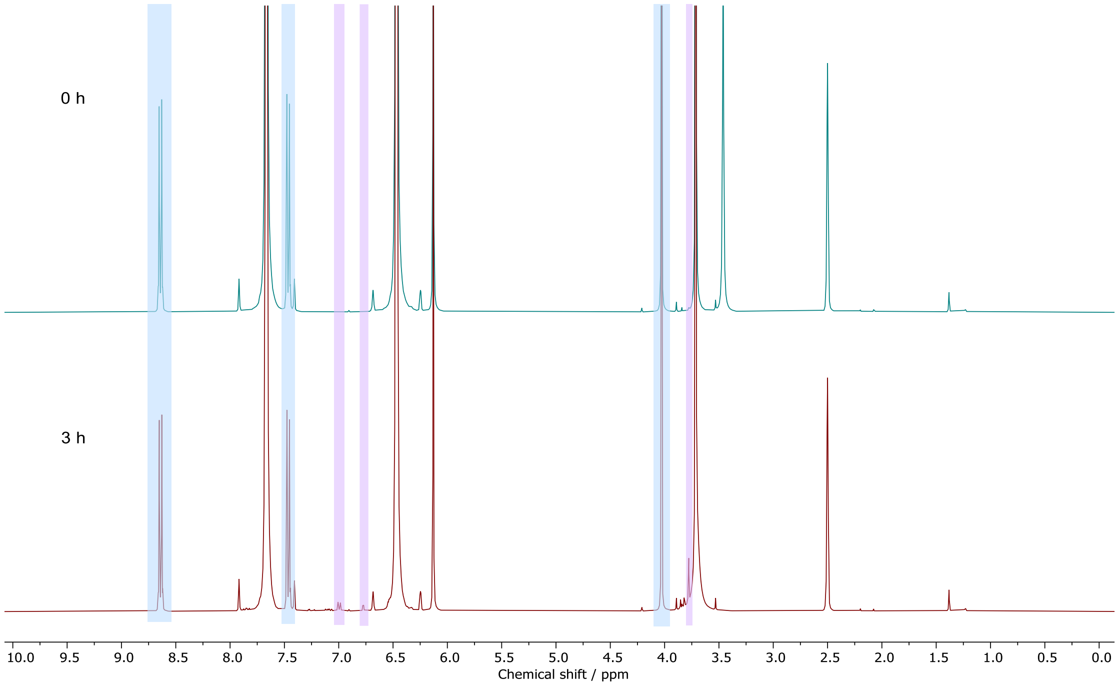

Figure S47: Control experiment in the presence of photocatalyst and in the absence of light. ^{1}H-NMR spectra measured after the indicated irradiation periods. Signals corresponding to the starting material and photoproduct are highlighted in blue and purple, respectively.

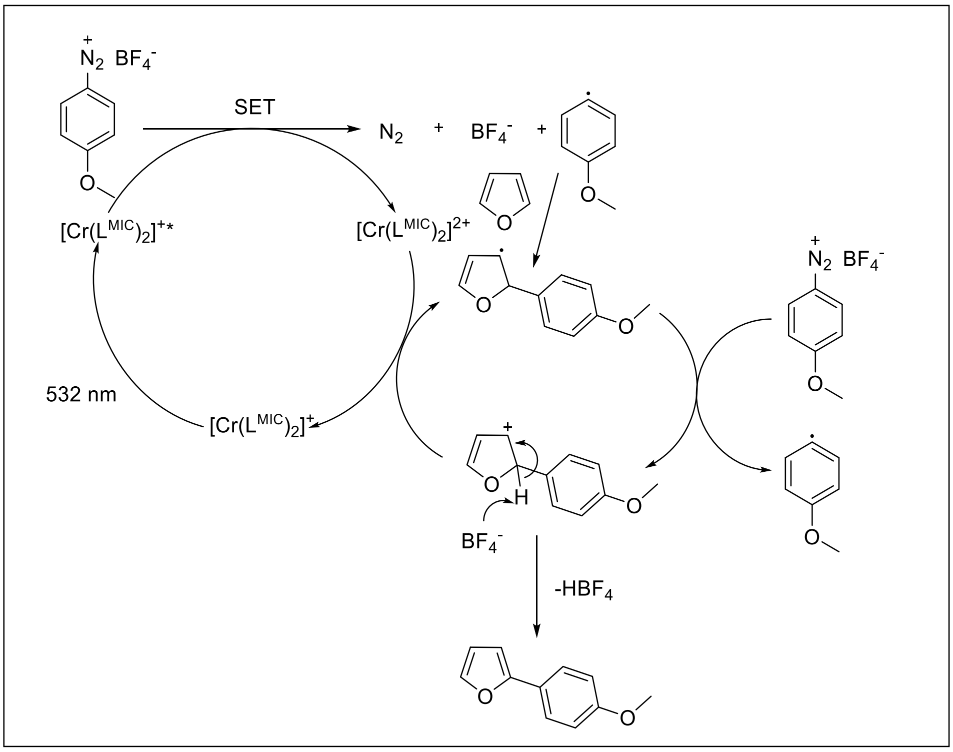

Figure S48: Mechanism of the photocatalytic C-H arylation of furan with 4-methoxyphenyl diazonium tetrafluoroborate, as reported in the literature.[11]

Photocatalytic borylation of 4-methoxyphenyl diazonium tetrafluoroborate with bis(pinacolato)diboron

The procedure was adapted from a previously published method.[12,13] 1,3,5-Trimethoxybenzene was used as internal standard. In an NMR tube 4-methoxyphenyl diazonium tetrafluoroborate (15 mg, 67.6 µmol, 1 eq.), **[Cr(L^{MIC})$_2$]BF$_4$** (1 mol.%), 1,3,5-trimethoxybenzene (11.4 mg, 67.6 µmol, 1 eq.) and bis(pinacolato)diboron (52.0 mg, 203 µmol, 3 eq.) were dissolved in 0.6 mL of dry MeCN-d^3, then the solution was deaerated. The sample was continuously irradiated with a 520 nm Kessil LED lamp for 16 hours, ^{1}H NMR spectra were measured every 2 hours to monitor the reaction progress. A yield of 74% and conversion of >99% were determined relative to the internal standard.

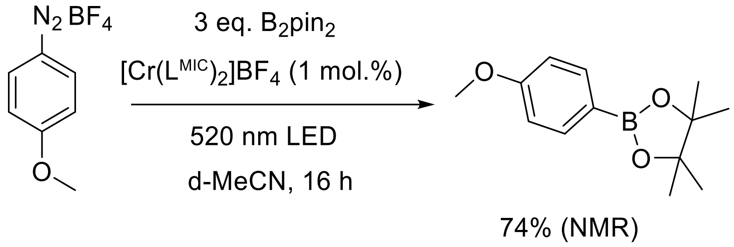

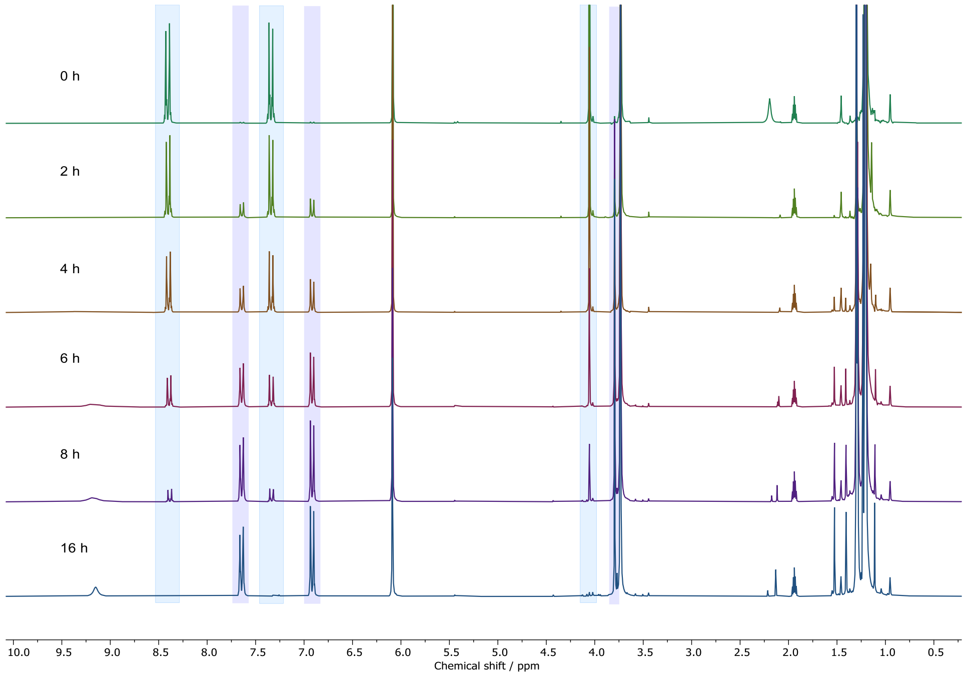

Figure S49: Borylation of 4-methoxyphenyl diazonium tetrafluoroborate with bis(pinacolato)diboron was monitored via ^{1}H-NMR spectroscopy over indicated irradiation periods. Signals corresponding to the starting material and photoproduct are highlighted in blue and purple, respectively.

A control experiment was performed to assess the photocatalytic activity of **[Cr(L^{MIC})$_2$]BF$_4$.** The same experiment as described above was repeated in the absence of the photocatalyst. The control experiment revealed a product yield of 10% and a starting material conversion of 22%.

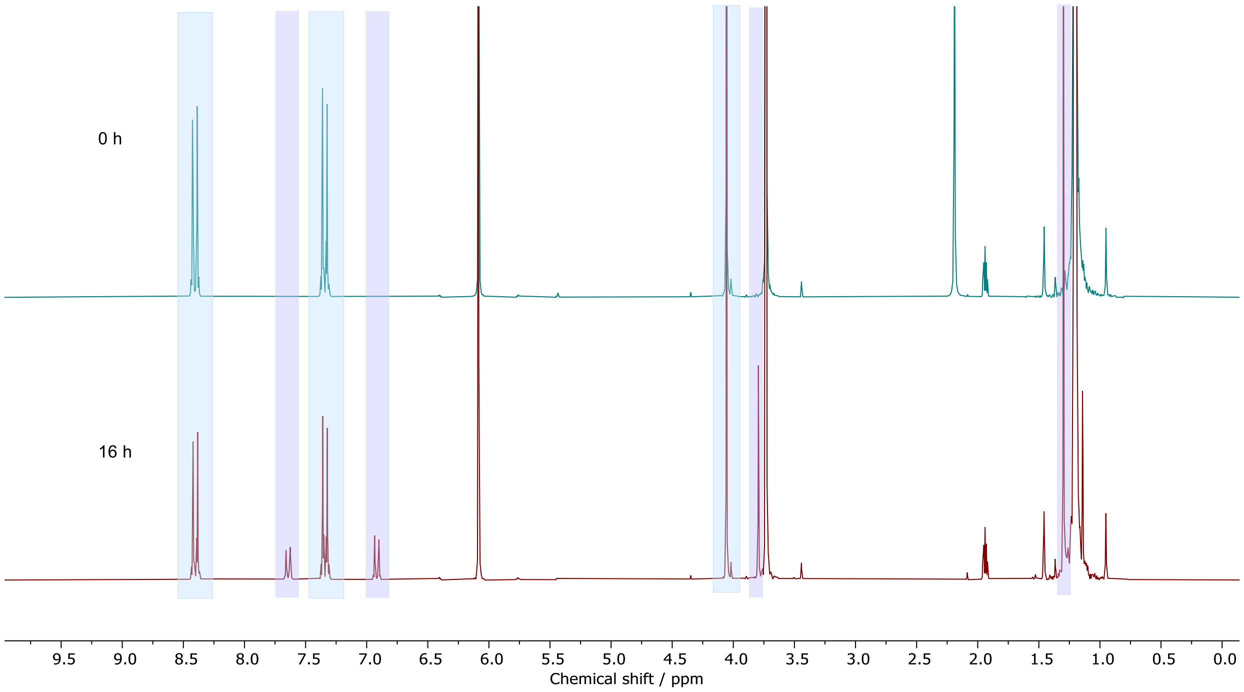

Figure S50: Control experiment in the absence of the photocatalyst. ^{1}H-NMR spectra measured after the irradiation periods. Signals corresponding to the starting material and photoproduct are highlighted in blue and purple, respectively.

A control experiment was performed to assess the photocatalytic activity of **$[Cr(L^{MIC})_2]BF_4$.** The same experiment as described above was repeated in the presence of photocatalyst and in the absence of light. The control experiment revealed that, in this case, trace signals of the product (<0.1 mM) were already detectable in the spectrum immediately after sample preparation (see 0 h panel in Figure S51), further after 16 h a product yield of 3.2% and a starting material conversion of 8.8% were achieved.

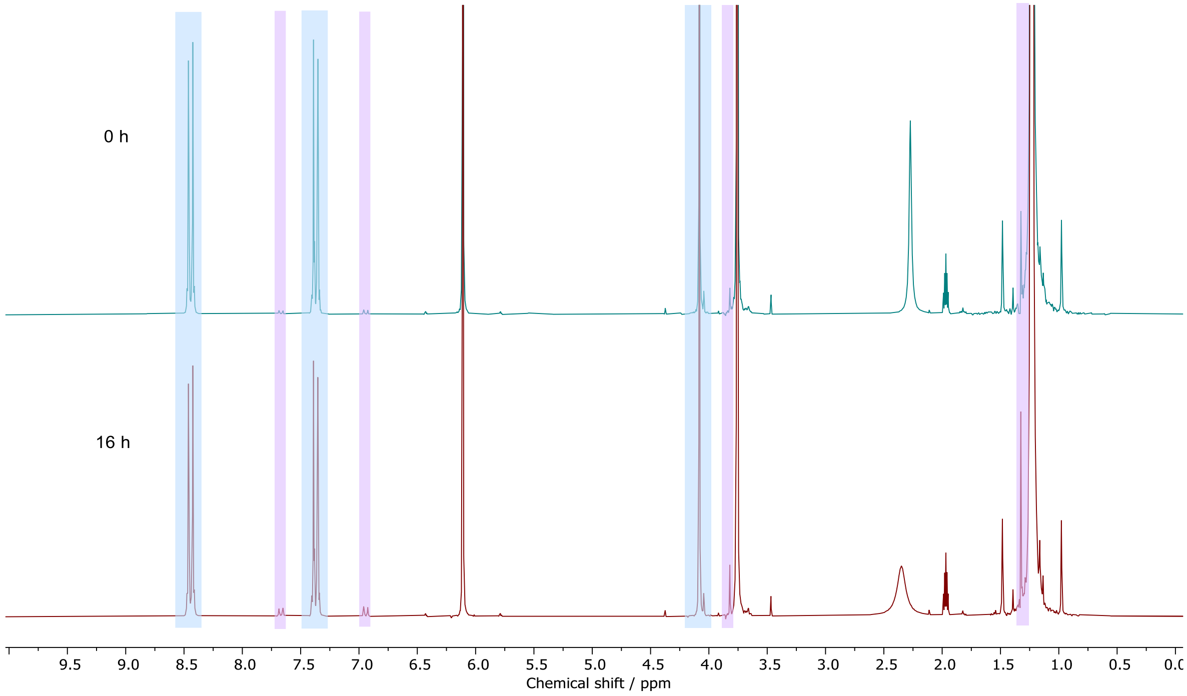

Figure S51: Control experiment in the presence of photocatalyst and in the absence of light. ^{1}H-NMR spectra measured after the irradiation periods. Signals corresponding to the starting material and photoproduct are highlighted in blue and purple, respectively.

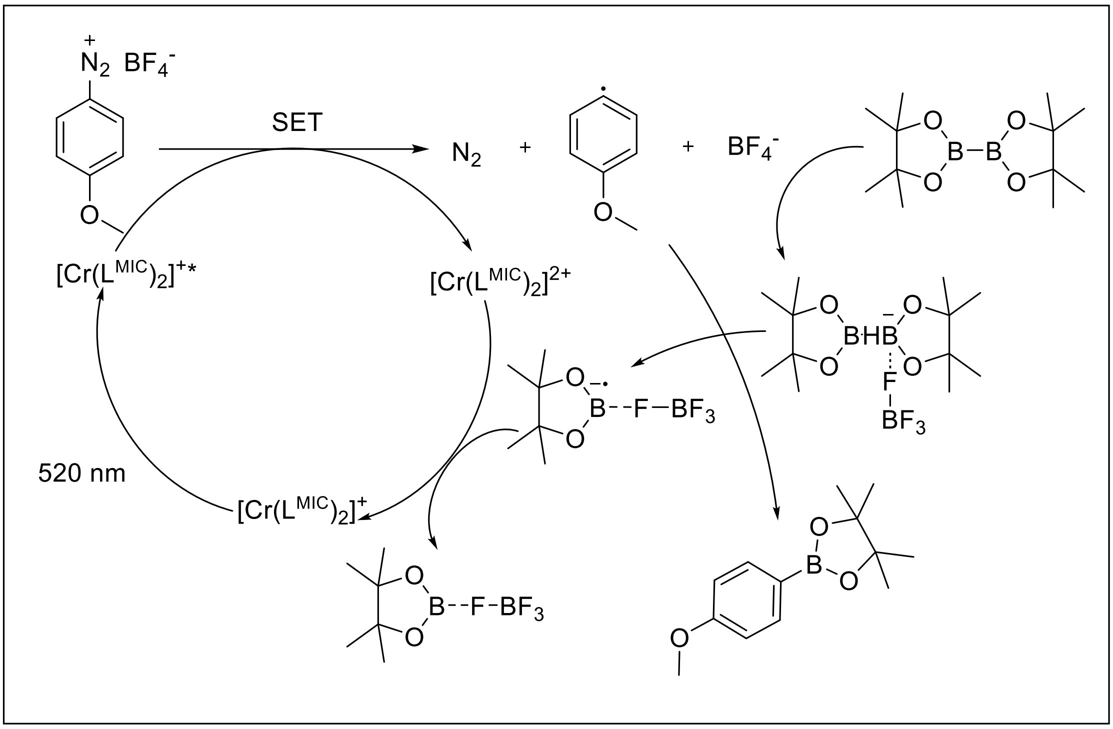

Figure S52: Mechanism of photocatalytic 4-methoxyphenyl diazonium tetrafluoroborate borylation with bis(pinacolato)diboron, reported in the literature.[13,14]

11. Crystallographic Information

*Table S2: Crystallographic data for complexes **[Cr(L^MIC)₂]⁺** and **[Cr(L^NHC)₂]⁺**.*

	**[Cr(L^{MIC})$_2$][BF_4]**	**[Cr(L^{NHC})$_2$][BF_4]**
Chemical formula	$C_{64}H_{76}N_{14}Cr_1\ B_1F_4$ 3(C_6H_{14})	$C_{56}H_{64}N_{10}Cr_1\ B_1F_4$ 1.7($C_3H_6O_1$)
M_r	1438.71	1114.71
Crystal system	Monoclinic	Monoclinic
Space group	$P2_1/c$	$P2_1/c$
a (Å)	13.9525(9)	10.6942(9)
b (Å)	32.012(3)	23.5231(19)
c (Å)	17.1974(14)	23.714(2)
α (°)	90	90
β (°)	99.868(2)	93.368(3)
γ (°)	90	90
V (Å^3)	7566.1(10)	5955.3(9)
Z	4	4
Densitiy (g cm^{-3})	1.263	1.243
F(000)	3092	2358
Radiation Type	MoKα	MoKα
μ (mm^{-1})	0.214	0.255
Crystal size (mm)	0.34x0.32x0.30	0.20x0.19x0.02
Meas. Refl.	173087	100688
Indep. Refl.	13290	10567
Obsvd. [$I > 2\sigma(I)$]	10488	6857
R_{int}	0.0845	0.1086
R_1 [$I > 2\sigma(I)$]	0.0732	0.0600
$wR_2(F^2)$	0.2138	0.1761
GooF	1.043	1.039
$\Delta\rho_{max}$ (e Å^{-3})	1.183	0.299
$\Delta\rho_{min}$ (e Å^{-3})	-0.436	-0.504
CCDC	2304955	2343870

*Table S3: Selected bond lengths and angles for complex **[Cr(L^{MIC})$_2$]$^+$** and **[Cr(L^{NHC})$_2$]$^+$***

	**[Cr(L^{MIC})$_2$][BF_4]**	**[Cr(L^{NHC})$_2$][BF_4]**
Cr1-N10	2.026(2)	2.002(2)
Cr1-N10A	2.025(2)	2.004(2)
Cr1-C1	2.136(3)	2.127(3)
Cr1-C2	2.137(3)	2.159(3)
Cr1-C1A	2.144(3)	2.160(3)
Cr1-C2A	2.136(3)	2.141(3)
C1-Cr1-C2	176.38(11)	173.00(12)
C1A-Cr1-C2A	175.62(12)	172.57(12)
N10-Cr1-N10A	179.90(13)	178.85(11)

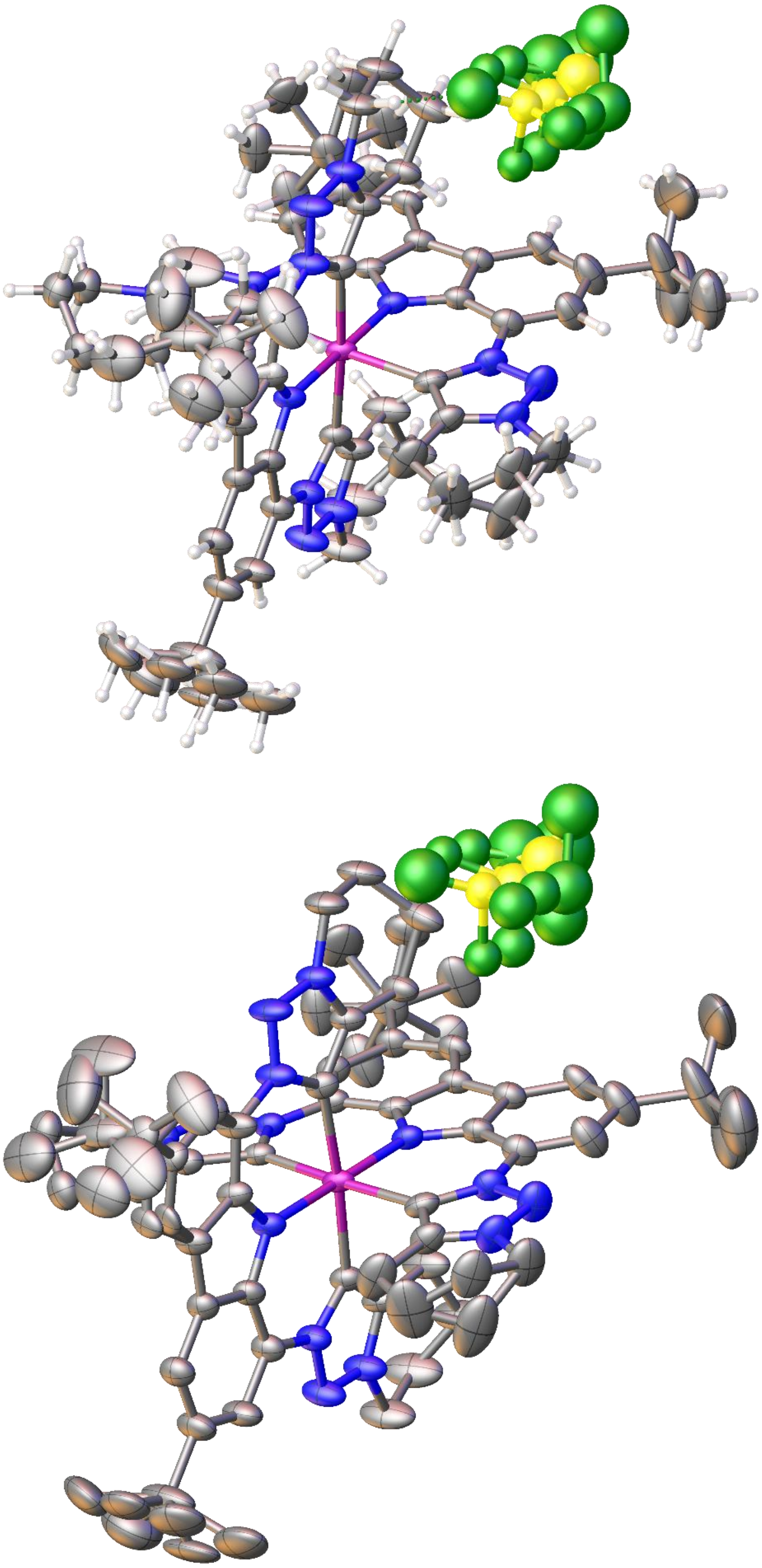

*Figure S53: Molecular structure of complex **[Cr(L^{MIC})$_2$][BF$_4$]** displayed with (top) and without (bottom) hydrogen atoms and full molecular disorders, including the positional disorder of the tetrafluoroborate counter ions.*

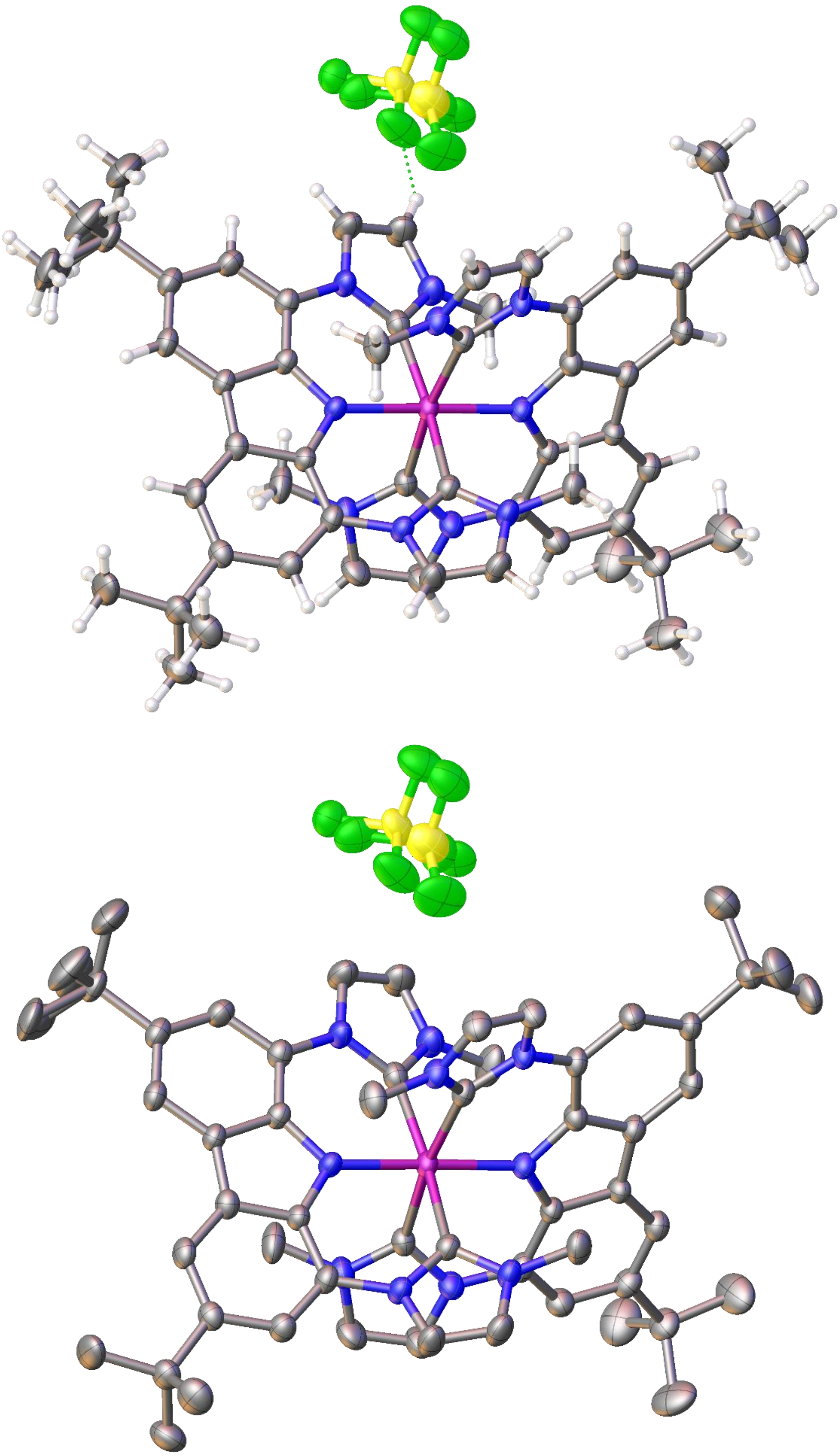

Figure S54: Molecular structure of complex ***$[Cr(L^{NHC})_2][BF_4]$*** *displayed with (top) and without (bottom) hydrogen atoms and full molecular disorders, including the positional disorder of the tetrafluoroborate counter ions.*

12. EPR Spectroscopy

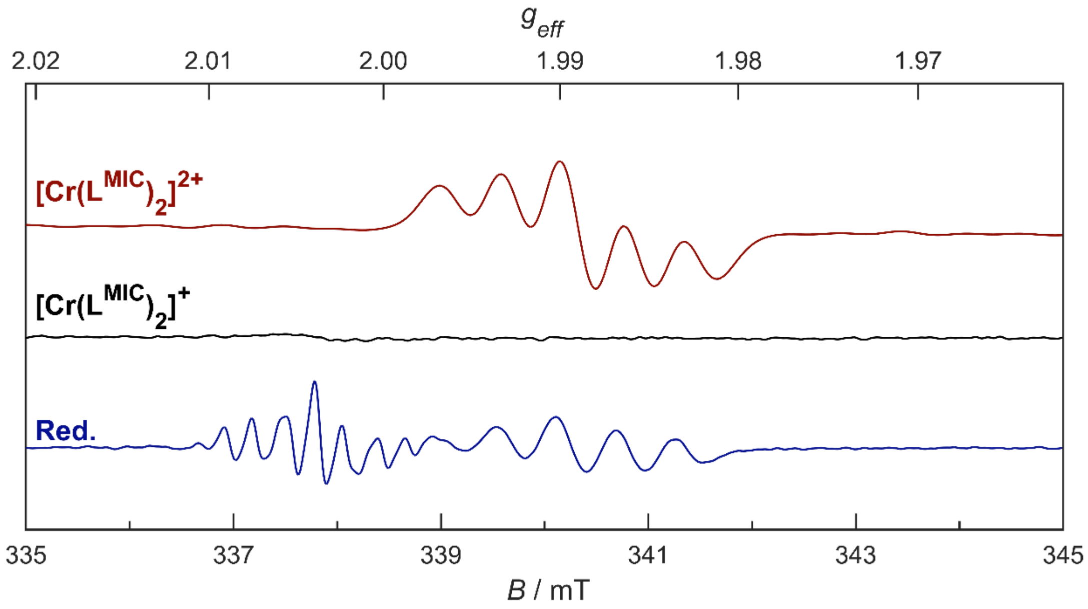

Figure S55: Solution (238 K) EPR spectra of the in-situ oxidation and reduction of $[Cr(L^{MIC)}{}_2]^+$. The oxidation shows one organic species, while the reduction shows (at least) two different signals, we attribute one to the chromium-bound ligand and the second to a free ligand radical (g = 2.003). We hence assume that the complex undergoes further chemical transformation upon reduction, including ligand dissociation.

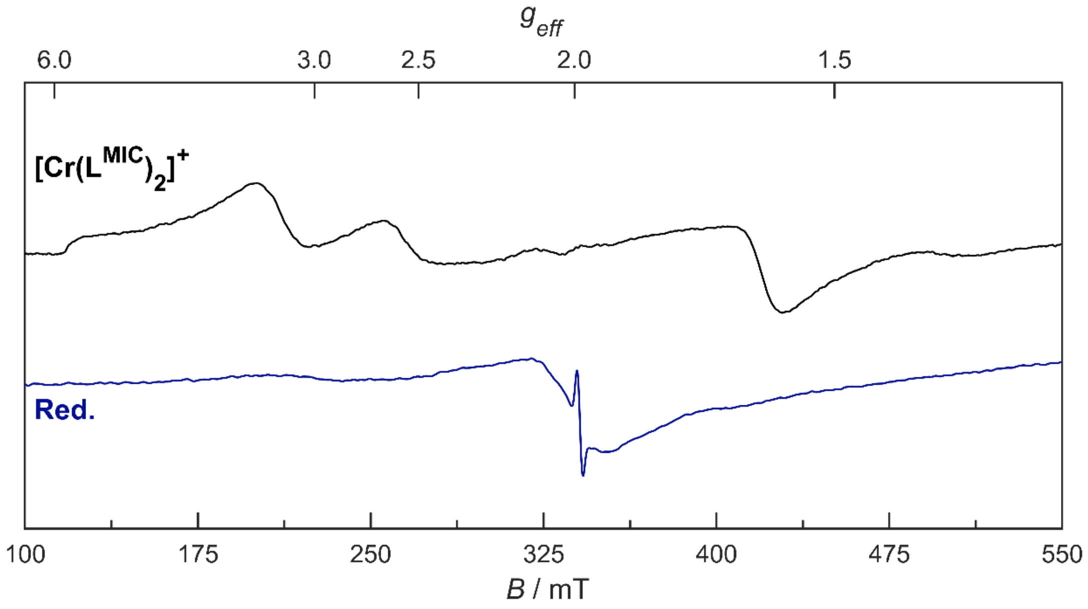

Figure S56: Frozen solution (98 K) EPR spectra of the in-situ reduction of $[Cr(L^{MIC)}{}_2]^+$. The chromium signal disappears and is mostly replaced *by the organic signature (see Fig. S 50).*

13. Computational Details

The calculations were performed with ORCA 5.0.4.[15,16] All calculated structures were verified as true minima by the absence of imaginary eigenvalues in the harmonic vibrational frequency analysis. Tighter than default convergence criteria were chosen for both the optimization of the structural parameters (*tightopt*) and the scf (*tightscf*). The geometry optimizations for $[Cr]^{+}$ were performed at the r^2SCAN-3c,[17] ZORA-PBE0-D4/ZORA-def2-SVP (Cr: ZORA-def2-TZVP),[18–21] ZORA-ωB97X-V/ZORA-def2-SVP (Cr: def2-TZVP)[22] and ZORA-cam-B3LYP/ZORA-def2-SVP (Cr: ZORA-def2-TZVP)[23] level of theories. The geometry optimizations for [Cr] and $[Cr]^{2+}$ were performed at the r^2SCAN-3c and ZORA-PBE0-D4/ZORA-def2-SVP (Cr: ZORA-def2-TZVP) level of theories only. Where applicable, the zeroth-order regular approximation (ZORA) in conjunction with the related basis sets was used to include scalar relativistic effects, the RIJCOSX approximation[24–26] and the related auxiliary basis set SARC/J[26,27] (*autoaux*, respectively, for AuxC)[28] were used to speed up the calculations. The r^2SCAN-3c method was found to afford the best fit with the structural parameters found in the solid state (Table S4). Various low-spin (including broken-symmetry) states were computationally evaluated, yet did either not converge or were found to be higher in energy (Table S3). Population analyses were conducted with single-point calculations at the triple-ζ level of theory (ZORA-def2-TZVPP), Intrinsic Bond Orbitals (IBOs)[29] were calculated at the triple-ζ level of theory as well. The absorption spectra were then modelled by time-dependent DFT (TD-DFT) as single point calculations using the r^2SCAN-3c structural parameters (S = 3/2) using the ZORA-def2-TZVPP basis set (ZORA-TPSSh[30], ZORA-cam-B3LYP, ZORA-ωB97X-D, Fig. S50). Solvation effects in acetonitrile were explored with the Conductor-like Polarizable Continuum Model (CPCM) module,[31] yet were found to be small (Tables S9, 11).

Furthermore, CASSCF calculations were performed for **$[Cr(L^{MIC})_2]^{+}$**, **$[Mn(L^{MIC})_2]^{2+}$** as well as the NHC- and pyridine congeners **$[Cr(L^{NHC})_2]^{+}$** and **$[Cr(L^{Py})_2]^{+}$**. For these calculations, model systems were used with all ^{t}Bu groups truncated with hydrogen atoms (*optimizehydrogens*). Also here, ZORA in combination with the ZORA-def2-TZVPP model system was used. The energies of states were refined using n-electron valence state perturbation theory (NEVPT2)[32] to account for dynamic correlation without using the frozencore approximation (*nofrozencore*). Various active spaces were explored, namely CASSCF(7,10), CASSCF(7,11), and CASSCF(7,13), which included all five *d*-orbitals, two doubly occupied carbazolido-based orbitals and their antibonding combinations, as well as one (two, four, respectively) ligand-based unoccupied orbitals. We consider CASSCF(7,11) for **$[Cr(L^{MIC})_2]^{+}$**, **$[Cr(L^{Py})_2]^{+}$** and **$[Mn(L^{MIC})_2]^{2+}$** and CASSCF(7,10) for **$[Cr(L^{NHC})_2]^{+}$** most reliable / comparable, the arguably more appropriate CASSCF(7,13) active space proved difficult to converge (and also computationally very demanding) for some of the compounds. State averaging was applied as given below. The Ab-Initio Ligand Field Theory (AILFT) analysis[33] was conducted at the NEVPT2 level of theory as well with CASSCF(3,5) and 40 doublet roots as well as 10 quartet roots. Molecular orbitals and optimized geometries were visualized with Chemcraft,[34] Avogadro,[35] and IBOView.[29]

Chromium MIC Complex $[Cr(L^{MIC})_2]^+$

Table S4: Energies of DFT-computed structures.

	***E* in [Eh]**	***G* in [Eh]**	***E*(SP) in [Eh]**
**$[Cr(L^{MIC})_2]^+$_r²SCAN3c_truncated**	-3665.890266	-3665.098647	n.a.
**$[Cr(L^{MIC})_2]^+$_r²SCAN3c**	-4294.721925	-4293.504046	n.a.
**$[Cr(L^{MIC})_2]^+$_doublet_r²SCAN3c**	scf not converging		
**$[Cr(L^{MIC})_2]^+$_camB3LYP**	-4304.131526	-4302.880943	n.a.
**$[Cr(L^{MIC})_2]^+$_PBE0**	-4301.879608	-4300.635713	-4305.226442
**$[Cr(L^{MIC})_2]^+$_doublet_PBE0**	-4301.843347	-4300.598725	-4305.190469
**$[Cr(L^{MIC})_2]^+$_wB97XV**	-4304.759627	n.a.	n.a.
**$[Cr(L^{MIC})_2]^+$_PBE**	-4301.635943	-4300.439543	n.a.
**$[Cr(L^{MIC})_2]$_sUKS_r²SCAN3c**	scf not converging		
**$[Cr(L^{MIC})_2]$_sUKS_PBE0**	-4301.964783	-4300.683215	-4305.272879
**$[Cr(L^{MIC})_2]$_t_r²SCAN3c**	-4294.846135	-4293.631774	n.a.
**$[Cr(L^{MIC})_2]$_t_PBE0**	-4301.994473	-4300.754611	-4305.341752
**$[Cr(L^{MIC})_2]$_q_r²SCAN3c**	-4294.836153	-4293.624062	n.a.
**$[Cr(L^{MIC})_2]$_q_PBE0**	-4301.984829	-4300.747906	-4305.333074
**$[Cr(L^{MIC})_2]^{2+}$_sUKS_r²SCAN3c**	scf not converging		
**$[Cr(L^{MIC})_2]^{2+}$_sUKS_PBE0**	-4301.555676	-4300.310070	-4304.904535
**$[Cr(L^{MIC})_2]^{2+}$_t_r²SCAN3c**	-4294.443915	-4293.224996	n.a.
**$[Cr(L^{MIC})_2]^{2+}$_t_PBE0**	-4301.586180	-4300.342281	-4304.934678
**$[Cr(L^{MIC})_2]^{2+}$_q_r²SCAN3c**	-4294.433199	-4293.215958	n.a.
**$[Cr(L^{MIC})_2]^{2+}$_q_PBE0**	-4301.577567	-4300.335327	-4304.925938

*Table S5: Benchmark of computed structural parameters with values found in the solid state of **$[Cr(L^{MIC})_2]^+$**. Note that the degeneracy of the four MIC ligands is lifted in the solid state through interaction with the counteranion BF_4^-. Bond lengths are given in [Å], angles in [°].*

	**SC-XRD**	**r²SCAN-3c**	**PBE-D4/def2-SVP**	**PBE0-D4/def2-SVP**	**ωB97X-V/def2-SVP**	**cam-B3LYP-D4/def2-SVP**
**Cr-C2**	2.144					
**Cr-C1 (3×)**	2.136	2.138	2.115	2.12	2.142	2.134
**Cr-N2**	2.025	2.019	2.004	2.005	2.017	2.012
**N-Cr-C (between pincers)**	91.9	91.9	91.2	91.3	91.5	91.5

Table S6: Löwdin's atomic spin densities on Cr in [a.u.].

	**r²SCAN-3c**	**PBE0**
**$[Cr(L^{MIC})_2]$**.	2.57	2.72
**$[Cr(L^{MIC})_2]^+$**	2.83	2.87
**$[Cr(L^{MIC})_2]^{2+}$**	2.47	2.66

Table S7: Number of electrons in d-orbitals according to localized IBOs (cutoff: 0.7). All redox states (their ground states, respectively) are predicted to feature a d^3 electron configuration.

	**r^2SCAN-3c**	**PBE0**
**$[Cr(L^{MIC})_2]$**.	d^3	d^3
**$[Cr(L^{MIC})_2]^+$**	d^3	d^3
**$[Cr(L^{MIC})_2]^{2+}$**	d^3	d^3

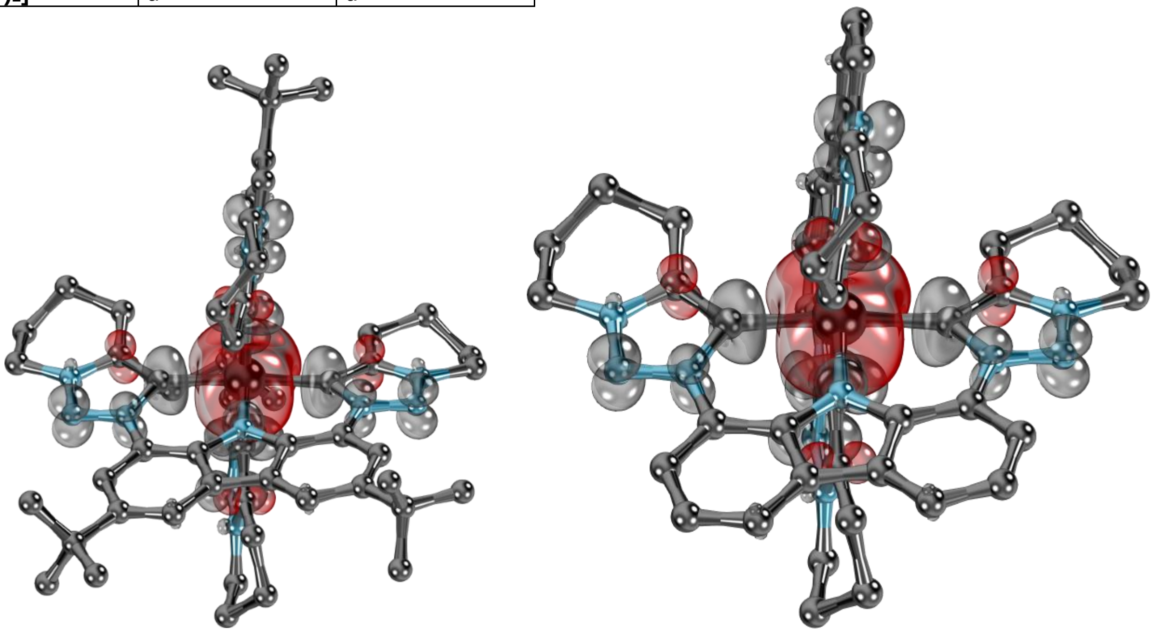

*Figure S57: Spin density in **$[Cr(L^{MIC})_2]$** as obtained at the r^2SCAN-3c level of theory (S = 3/2). Orbitals obtained with ZORA-PBE0-D4/def2-TZVPP//ZORA-PBE0-D4/def2-SVP are consistent. The spin density plot indicates anti-ferromagnetic coupling with a delocalized, ligand-centered radical. ^{t}Bu in right structure truncated for clarity.*

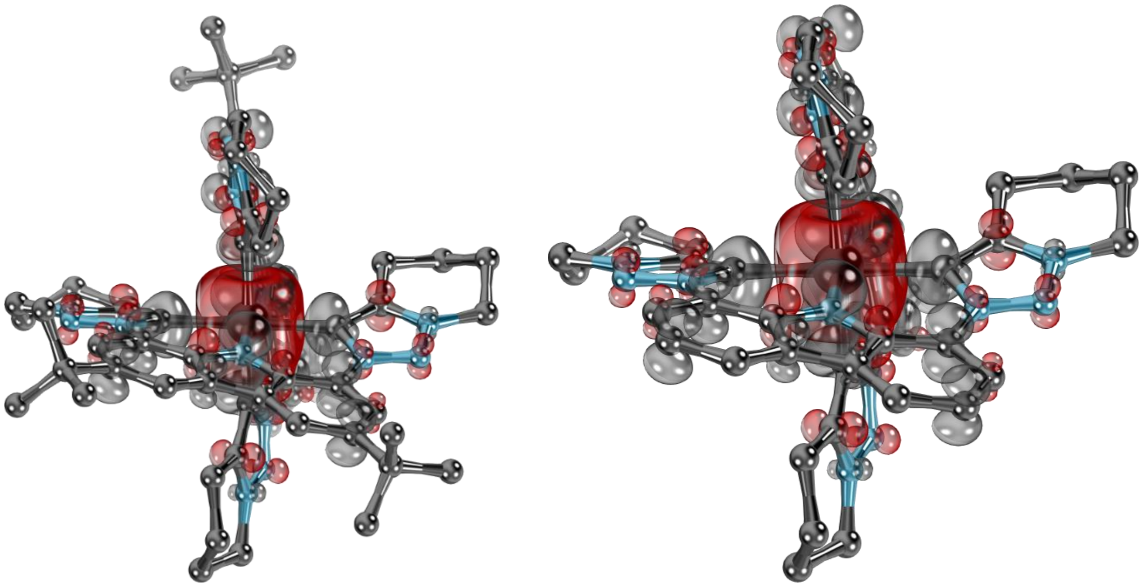

*Figure S58: Spin density in **$[Cr(L^{MIC})_2]^{2+}$** as obtained at the r^2SCAN-3c level of theory (S = 3/2). Orbitals obtained with ZORA-PBE0-D4/def2-TZVPP//ZORA-PBE0-D4/def2-SVP are consistent. The spin density plot indicates anti-ferromagnetic coupling with a delocalized, ligand-centered radical. ^{t}Bu in right structure truncated for clarity.*

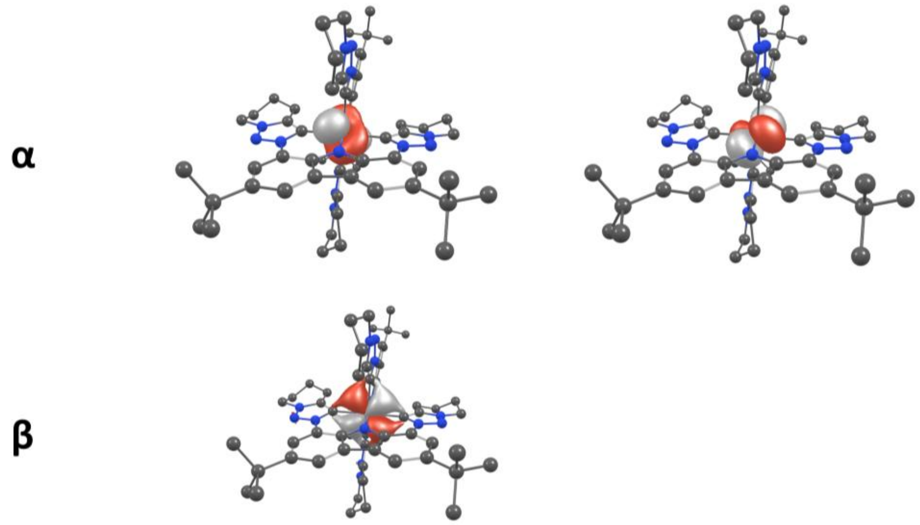

*Figure S59: Metal-centered IBOs for the spin-flip (broken-symmetry, respectively) doublet excited state of **[Cr(L^{MIC})$_2$]$^+$** as obtained at the ZORA-PBE0/def2-TZVPP//ZORA-PBE0-D4/def2-SVP level of theory. The computations with r^{2}SCAN-3c did not converge.*

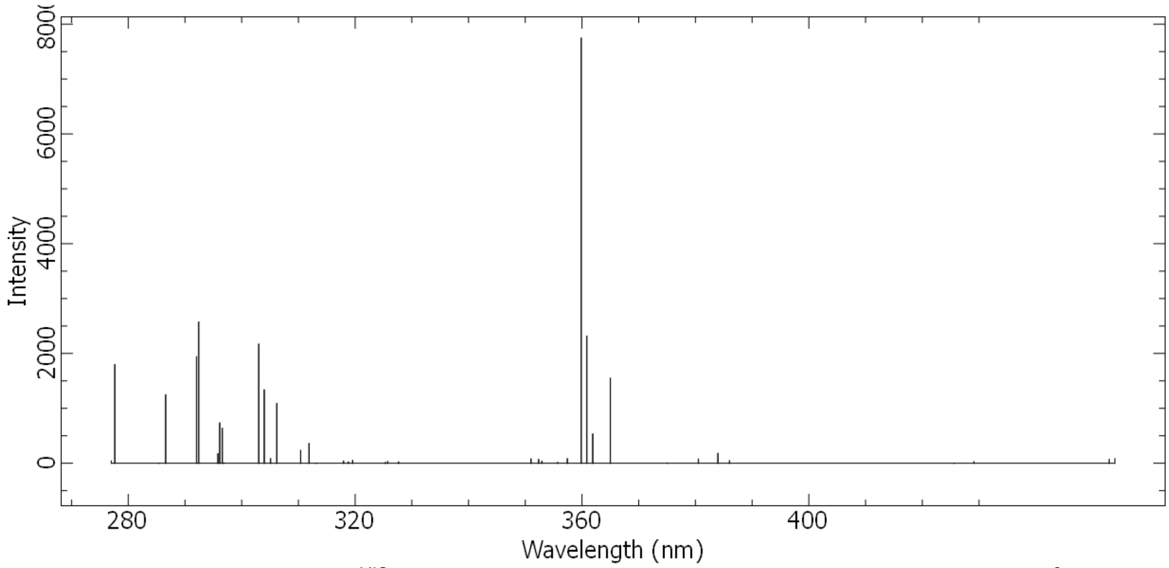

Figure S60: Absorption spectrum of [Cr(L^{MIC})$_2$]$^+$ (S = 3/2) as obtained by ZORA-cam-B3LYP/def2-TZVPP//r^{2}SCAN-3c.

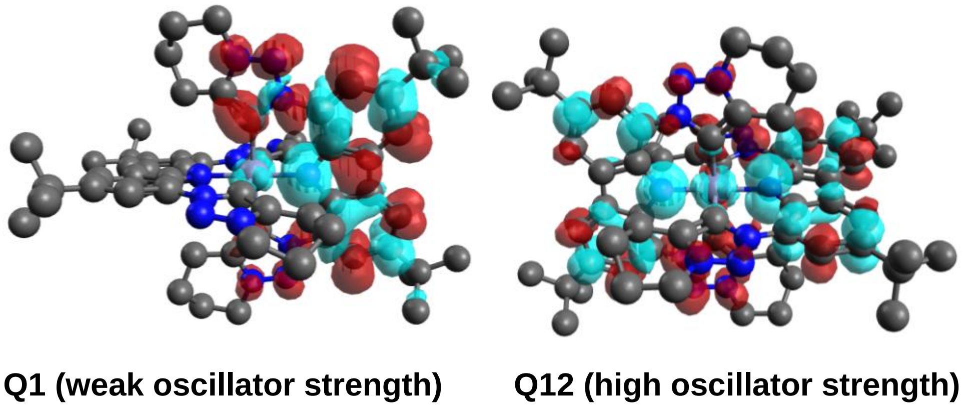

*Figure S61: Transition difference densities for pertinent quartet excited state of **[Cr(L^{MIC})$_2$]$^+$** (S = 3/2; cam-B3LYP).*

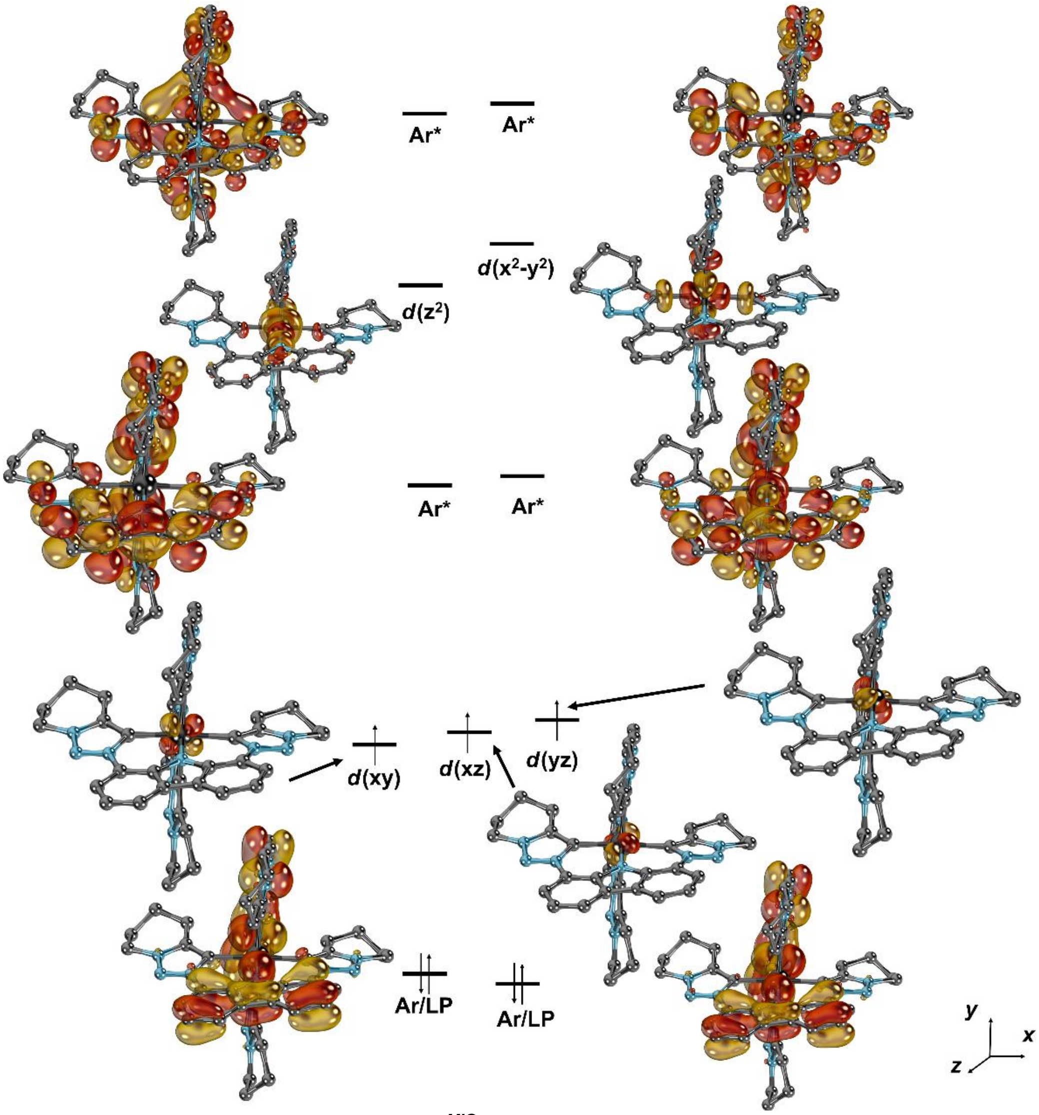

*Figure S62: Molecular Orbital diagram (c = 0.98) of **[Cr(L^{MIC})$_2$]$^+$** (S = 3/2) as obtained by state-averaged CASSCF(7,11) in order of decreasing occupation (and not energy) of natural orbitals. Isodensity values are adjusted for clarity.*

*Table S8: Configurations of pertinent roots (NEVPT2) for Molecular Orbital diagram of **[Cr(L^{MIC})$_2$]$^+$** (S = 3/2) as obtained by CASSCF(7,11) in order of decreasing occupation (and not energy) of natural orbitals. Root 1 (LLCT) features low oscillator strength (fosc = 0.02), roots 5 (ILCT; fosc = 0.07) and 7 (LLCT; fosc = 0.14) feature moderate oscillator strength, roots 6 (ILCT; fosc = 0.47) and 8 (LLCTfosc = 0.22) feature high oscillator strengths.*

ROOT 0: *E* = 0 eV
0.98379 [0]: 22111000000
0.00418 [1127]: 12112000000
0.00301 [112]: 21121000000

ROOT 1 (LLCT): *E* = 2.36 eV
0.62282 [124]: 21111000010
0.16427 [1133]: 12111000001
0.13760 [84]: 21211000000
0.01993 [1128]: 12111100000
0.01534 [121]: 21111010000
0.00775 [11]: 22101000010
0.00556 [6]: 22110000001
0.00408 [125]: 21111000001
0.00287 [1132]: 12111000010

ROOT 5 (ILCT/LLCT): *E* = 2.71 eV
0.48324 [120]: 21111100000
0.27936 [1129]: 12111010000
0.08311 [1132]: 12111000010
0.05315 [125]: 21111000001
0.03627 [1130]: 12111001000
0.01066 [7]: 22101100000
0.00892 [5]: 22110000010
0.00864 [2]: 22110010000
0.00530 [121]: 21111010000
0.00309 [12]: 22101000001

ROOT 6 (ILCT/LLCT): *E* = 2.76 eV
0.45782 [1128]: 12111100000
0.31975 [121]: 21111010000
0.07223 [124]: 21111000010
0.04294 [122]: 21111001000
0.04234 [1133]: 12111000001
0.01770 [1]: 22110100000
0.00725 [120]: 21111100000
0.00545 [8]: 22101010000
0.00463 [11]: 22101000010
0.00434 [6]: 22110000001

ROOT 7 (ILCT/LLCT): *E* = 2.87 eV
0.54593 [1132]: 12111000010
0.20963 [125]: 21111000001
0.06544 [120]: 21111100000
0.04758 [1129]: 12111010000
0.03743 [5]: 22110000010
0.03324 [1092]: 12211000000
0.01305 [1130]: 12111001000
0.01076 [12]: 22101000001
0.00653 [124]: 21111000010
0.00269 [3903]: 02112000010
0.00255 [2]: 22110010000

ROOT 8 (ILCT/LLCT): *E* = 2.91 eV
0.55798 [124]: 21111000010
0.22907 [1133]: 12111000001
0.05005 [1128]: 12111100000
0.04426 [121]: 21111010000
0.02531 [84]: 21211000000
0.02479 [11]: 22101000010
0.02112 [6]: 22110000001
0.01362 [122]: 21111001000
0.00502 [125]: 21111000001
0.00403 [1132]: 12111000010
0.00317 [1614]: 11112000010

ROOT 9 (*d*-*d* transition, $^4T_2$): *E* = 3.03 eV
0.68379 [3]: 22110001000
0.19626 [10]: 22101000100
0.10067 [2]: 22110010000
0.00251 [1130]: 12111001000

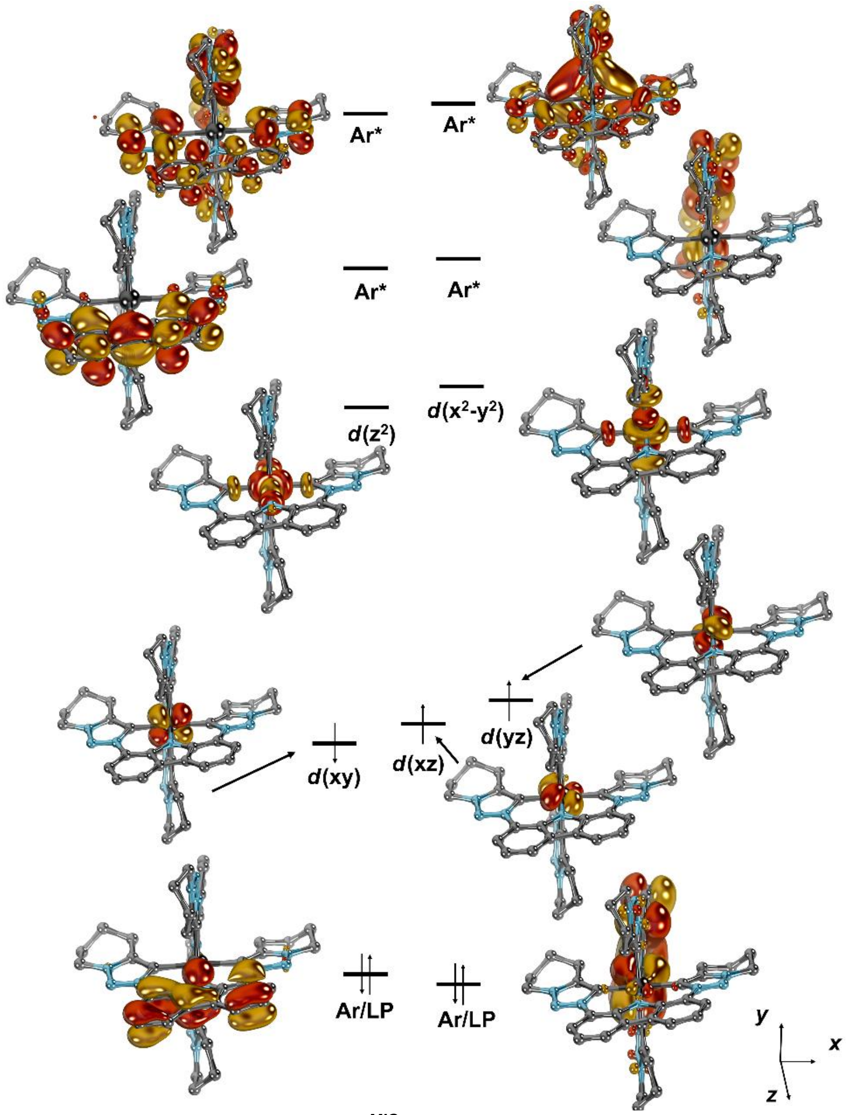

*Figure S63: Molecular Orbital diagram (c = 0.94) of **[Cr(L^{MIC})$_2$]$^+$** (S = 1/2) as obtained by state-averaged (40 roots) CASSCF(7,11) in order of decreasing occupation (and not energy) of natural orbitals. Isodensity values are adjusted for clarity.*

*Table S9: Configurations of pertinent roots (NEVPT2) for Molecular Orbital diagram of **[Cr(L^{MIC})$_2$]$^+$** (S = 1/2) as obtained by CASSCF(7,11) in order of decreasing occupation (and not energy) of natural orbitals.*

```
ROOT   0 (Spin-Flip State, 2E):  E = 1.70 eV (vs. Q0)
      0.94202 [     9]: 22111000000
      0.01744 [     3]: 22200010000
      0.00359 [     8]: 22120000000
      0.00330 [    52]: 22011100000
      0.00312 [    46]: 22020010000
      0.00310 [     2]: 22200100000
      0.00290 [   727]: 20111002000
      0.00265 [  4463]: 02111000200
      0.00261 [    79]: 22002100000

ROOT   2 (d–d transition, 2T1/2T2):  E= 1.75 eV (vs. Q0)
      0.59177 [     0]: 22210000000
      0.30435 [    51]: 22012000000
      0.03554 [     1]: 22201000000
      0.01780 [    44]: 22021000000
      0.01376 [    10]: 22110100000
      0.00642 [  1501]: 12111000001
      0.00330 [     9]: 22111000000
      0.00299 [    18]: 22101010000
      0.00273 [   205]: 21111000001

ROOT   3 (d–d transition, 2T1/2T2):  E= 1.80 eV (vs. Q0)
      0.50965 [     1]: 22201000000
      0.39076 [    44]: 22021000000
      0.03059 [     0]: 22210000000
      0.02415 [    51]: 22012000000
      0.01135 [    17]: 22101100000
      0.00510 [    11]: 22110010000
      0.00352 [   205]: 21111000001

ROOT   4 (d–d transition, 2T1/2T2):  E= 1.81 eV (vs. Q0)
      0.29364 [    16]: 22102000000
      0.22533 [     9]: 22111000000
      0.01217 [    45]: 22020100000
      0.00947 [    79]: 22002100000
      0.00507 [    46]: 22020010000

ROOT   5 (Spin-Flip State, 2E):  E = 1.92 eV (vs. Q0)
      0.73787 [     9]: 22111000000
      0.11253 [    16]: 22102000000
      0.11114 [     8]: 22120000000
      0.00651 [    52]: 22011100000
      0.00436 [    46]: 22020010000
      0.00370 [    80]: 22002010000
      0.00324 [    53]: 22011010000

ROOT   6 (ILCT/LLCT):  E= 2.58 eV (vs. Q0)
      0.05035 [  1498]: 12111001000
      0.04888 [    13]: 22110000100
      0.02047 [   203]: 21111000100
      0.00874 [    20]: 22101000100
      0.00520 [  4445]: 02112000100
      0.00361 [  1651]: 12011000101
      0.00295 [  3146]: 10111002100
      0.00294 [    12]: 22110001000
```

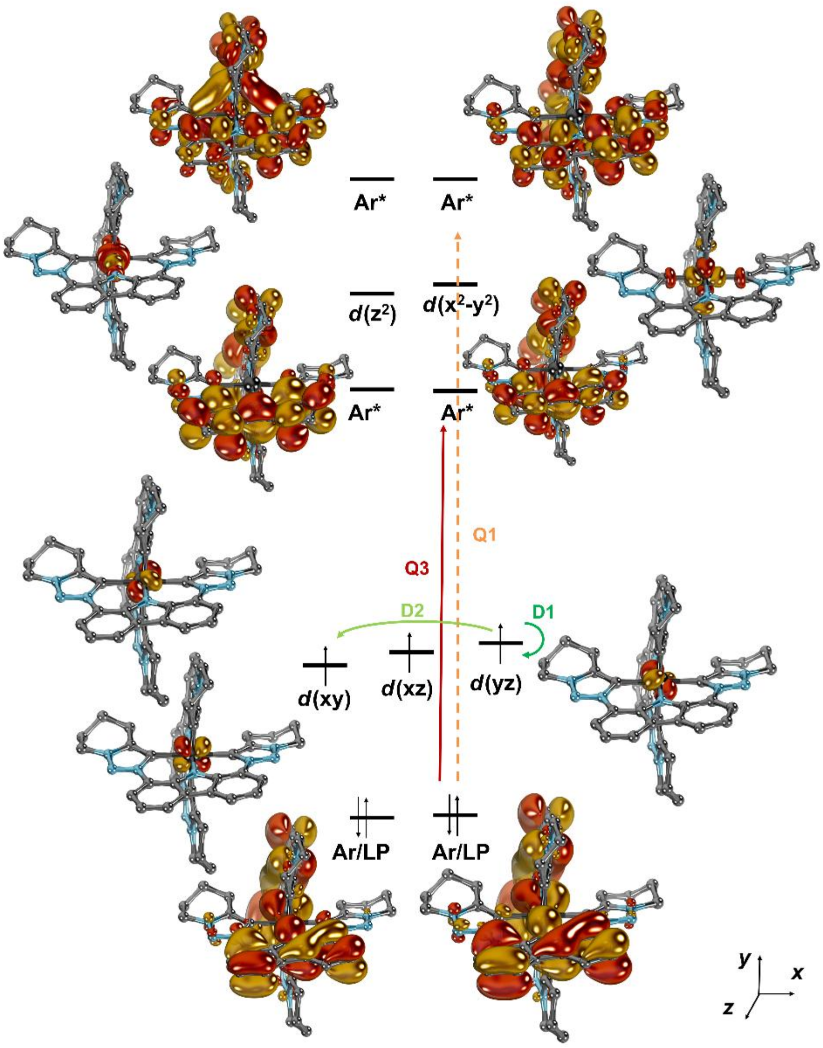

*Figure S64: Idealized electronic nature of pertinent transitions (excited states, respectively) of **[Cr(L^{MIC})$_2$]$^+$**. The D0 excited state is a spin-flip state; order of orbitals does not follow their energies, yet their occupations.*

*Table S10: Energies of vertical excited states by NEVPT2//CASSCF(7,11) of **$[Cr(L^{MIC})_2]^+$** (structural parameters of S = 3/2; separately state averaging for either quartets of doublets). Energies are given in [eV].*

Quartet	Gasphase	CPCM(MeCN)	Doublet	Gasphase	CPCM(MeCN)
**Q0**	0.00	0.00	**D1**	1.70	1.73
**Q1**	2.36	2.50	**D2**	1.75	1.75
**Q2**	2.44	2.57	**D3**	1.80	1.82
**Q3**	2.66	2.68	**D4**	1.81	1.82
**Q4**	2.66	2.69	**D5**	1.92	1.92
**Q5**	2.71	2.73	**D6**	2.58	2.61
**Q6**	2.76	2.79	**D7**	2.59	2.62
**Q7**	2.87	2.96	**D8**	2.71	2.73
**Q8**	2.91	3.01	**D9**	2.79	2.75
**Q9**	3.03	3.04	**D10**	2.91	2.92
**Q10**	3.12	3.13	**D11**	2.92	3.21

Comparison of Ligands (NHC, py) and Metal (Mn)

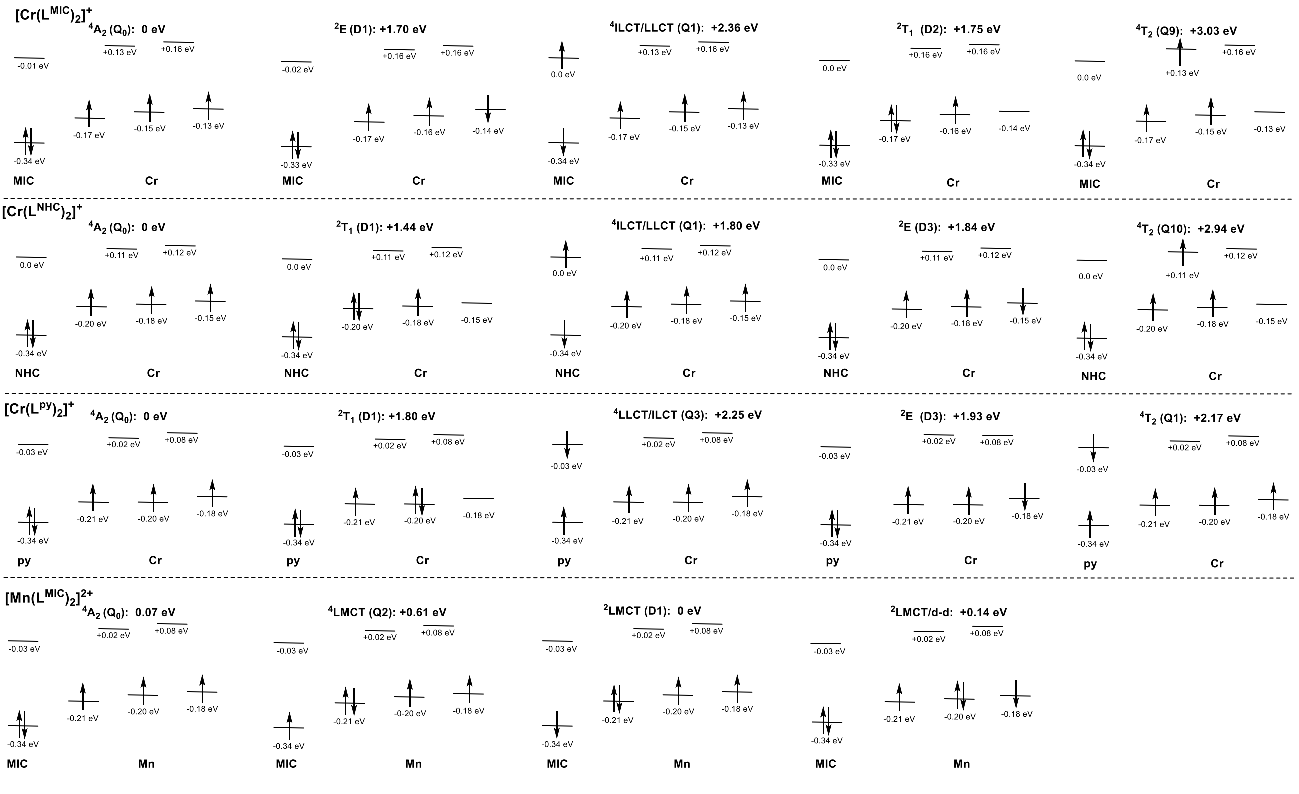

Figure S65: Idealized nature of pertinent transitions and their energies based on NEVPT2/CASSCF calculations (for all-state averaged calculations, in case of MIC-Cr additionally based on separate state-averaging for the quartet- and singlet states. Orbital energies are idealized, yet the order corresponds to their computed energies. Note that the orbital energies obtained by AILFT (vide infra) differ considerably, and further note that the states particularly for the Mn-complex are heavily multiconfigurational (vide infra).

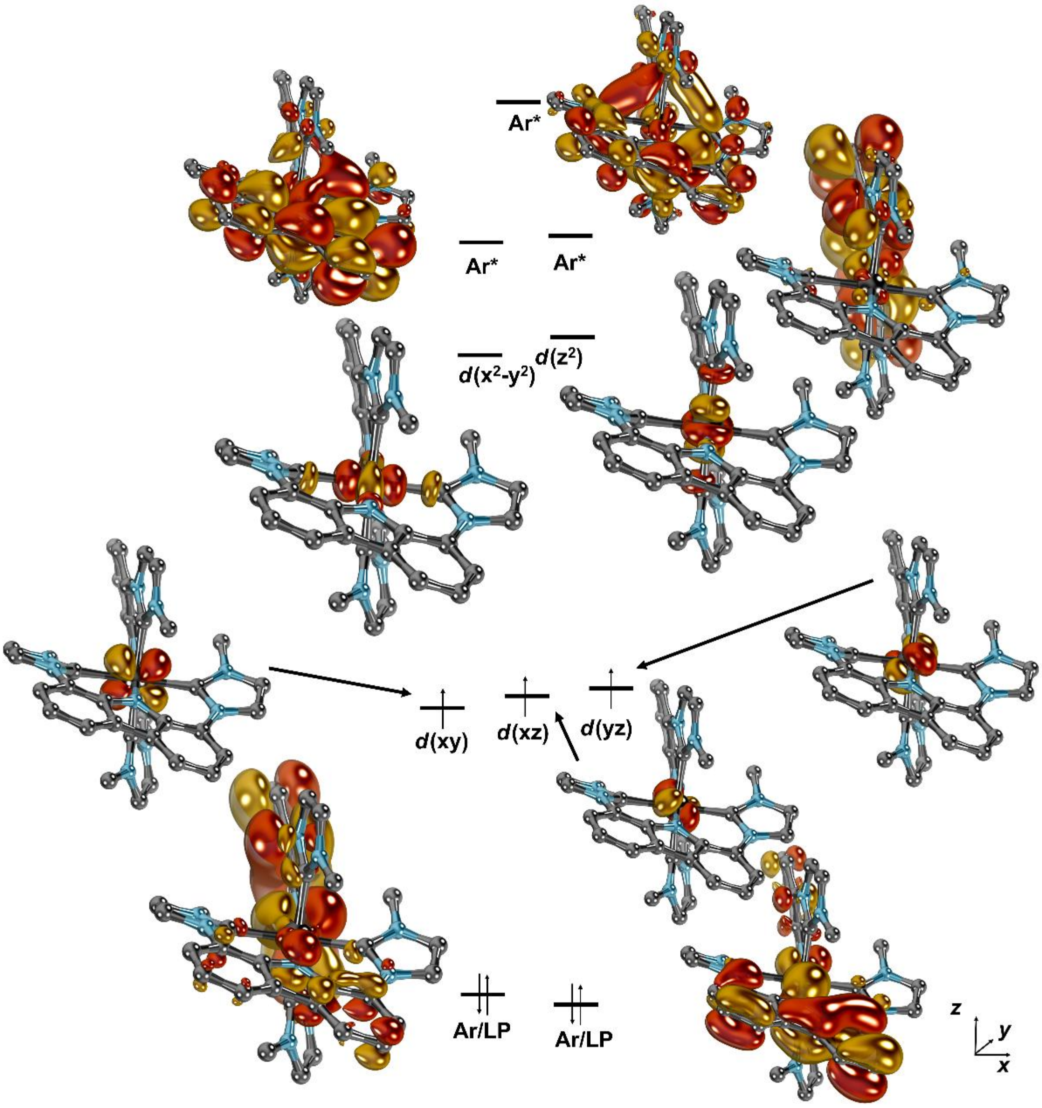

*Figure S66: Molecular Orbital diagram of **[Cr(L^{NHC})$_2$]$^+$** (S = 3/2, 20 roots; S = ½, 40 roots) as obtained by state-averaged CASSCF(7,10) in order of decreasing occupation (and not energy) of natural orbitals. Isodensity values are adjusted for clarity.*

*Table S11: Configurations of pertinent roots (NEVPT2) for Molecular Orbital diagram of **[Cr(L^{NHC})$_2$]$^+$** (S = 3/2, 20 roots; S = ½, 40 roots) as obtained by CASSCF(7,10) in order of decreasing occupation (and not energy) of natural orbitals.*

DOUBLETS:

ROOT 0 (*d-d* transition, **$^2T_1$/$^2T_2$**) E= 1.442 eV (vs. Q0)
0.75464 [0]: 2221000000
0.15248 [41]: 2201200000
0.01187 [10]: 2211001000
0.01115 [921]: 1211100001
0.01002 [1]: 2220100000
0.00975 [9]: 2211010000
0.00518 [920]: 1211100010
0.00503 [883]: 1221100000
0.00270 [13]: 2211000001

ROOT 1: (*d-d* transition, **$^2T_1$/$^2T_2$**) E= 1.803 eV (vs. Q0)
0.61112 [7]: 2212000000
0.28321 [14]: 2210200000
0.03649 [8]: 2211100000
0.01178 [37]: 2202001000
0.00899 [36]: 2202010000
0.00369 [40]: 2202000001
0.00362 [66]: 2200200001
0.00334 [42]: 2201110000
0.00300 [62]: 2200210000

ROOT 2: (*d-d* transition, **$^2T_1$/$^2T_2$**) E= 1.828 eV (vs. Q0)
0.61912 [1]: 2220100000
0.28818 [35]: 2202100000
0.01076 [8]: 2211100000
0.00839 [0]: 2221000000
0.00760 [16]: 2210101000
0.00723 [15]: 2210110000
0.00698 [19]: 2210100001
0.00497 [151]: 2111100001
0.00369 [41]: 2201200000
0.00336 [18]: 2210100010

ROOT 3: (Spin-Flip State, **^2E**) E= 1.844 eV (vs. Q0)
0.91963 [8]: 2211100000
0.01319 [2]: 2220010000
0.01180 [3]: 2220001000
0.00699 [1]: 2220100000
0.00455 [46]: 2201100001
0.00288 [12]: 2211000010
0.00280 [35]: 2202100000
0.00263 [37]: 2202001000

ROOT 4: (*d-d* transition, **$^2T_1$/$^2T_2$**) E= 2.017 eV (vs. Q0)
0.61112 [7]: 2212000000
0.28321 [14]: 2210200000
0.03649 [8]: 2211100000
0.01178 [37]: 2202001000
0.00899 [36]: 2202010000
0.00369 [40]: 2202000001
0.00362 [66]: 2200200001
0.00334 [42]: 2201110000
0.00300 [62]: 2200210000

ROOT 10: (*d-d* transition) 3.001 eV (vs. Q0)
0.27471 [9]: 2211010000
0.16637 [113]: 2121100000
0.15282 [883]: 1221100000
0.08748 [151]: 2111100001
0.08538 [921]: 1211100001
0.04942 [15]: 2210110000

QUARTETS:

ROOT 0: (**$^4A_2$**) E= 0 eV
0.95330 [0]: 2211100000
0.01143 [25]: 2201100001
0.00568 [24]: 2201100010
0.00366 [4]: 2211000010
0.00345 [657]: 1211200000
0.00271 [23]: 2201100100
0.00259 [3]: 2211000100

ROOT 1: (LMCT) E= 1.803 eV
0.50260 [630]: 1221100000
0.23517 [662]: 1211100001
0.06759 [661]: 1211100010
0.05508 [56]: 2121100000
0.02251 [88]: 2111100001
0.02148 [660]: 1211100100
0.02058 [87]: 2111100010
0.00774 [762]: 1201100002
0.00688 [86]: 2111100100
0.00614 [761]: 1201100011
0.00510 [658]: 1211110000
0.00350 [659]: 1211101000
0.00338 [5]: 2211000001
0.00285 [651]: 1212100000
0.00258 [868]: 1122100000
0.00252 [759]: 1201100101

ROOT 2: (LMCT) E= 1.907 eV
0.41277 [56]: 2121100000
0.26089 [88]: 2111100001
0.05713 [662]: 1211100001
0.05107 [87]: 2111100010
0.04858 [630]: 1221100000
0.03435 [661]: 1211100010
0.02809 [86]: 2111100100
0.02402 [660]: 1211100100
0.00760 [188]: 2101100002
0.00703 [657]: 1211200000
0.00557 [187]: 2101100011
0.00529 [84]: 2111110000
0.00476 [77]: 2112100000
0.00404 [874]: 1121200000
0.00393 [4]: 2211000010
0.00319 [85]: 2111101000
0.00315 [3]: 2211000100

ROOT 3: (*d-d* transition, **$^4T_2$**) E= 2.935 eV
0.49448 [1]: 2211010000
0.24409 [2]: 2211001000
0.13834 [6]: 2210110000
0.07783 [7]: 2210101000
0.00517 [29]: 2201010001
0.00325 [5]: 2211000001
0.00303 [32]: 2201001001
0.00277 [28]: 2201010010

ROOT 4: (LLCT/ILCT) E= 2.938 eV
0.67384 [86]: 2111100100
0.11430 [660]: 1211100100
0.06988 [56]: 2121100000
0.02648 [3]: 2211000100
0.01961 [83]: 2111200000

0.02856 [10]: 2211001000	0.01578 [8]: 2210100100
0.02152 [150]: 2111100010	0.01333 [88]: 2111100001
0.01610 [919]: 1211100100	0.01044 [185]: 2101100101
0.01526 [16]: 2210101000	0.00502 [184]: 2101100110
0.00969 [1]: 2220100000	0.00480 [657]: 1211200000
0.00762 [19]: 2210100001	0.00431 [661]: 1211100010
0.00681 [920]: 1211100010	0.00419 [662]: 1211100001
0.00548 [67]: 2200120000	0.00331 [87]: 2111100010
0.00289 [2442]: 0221200000	0.00286 [99]: 2111000110
0.00272 [1027]: 1201100002	
0.00261 [257]: 2101100002	

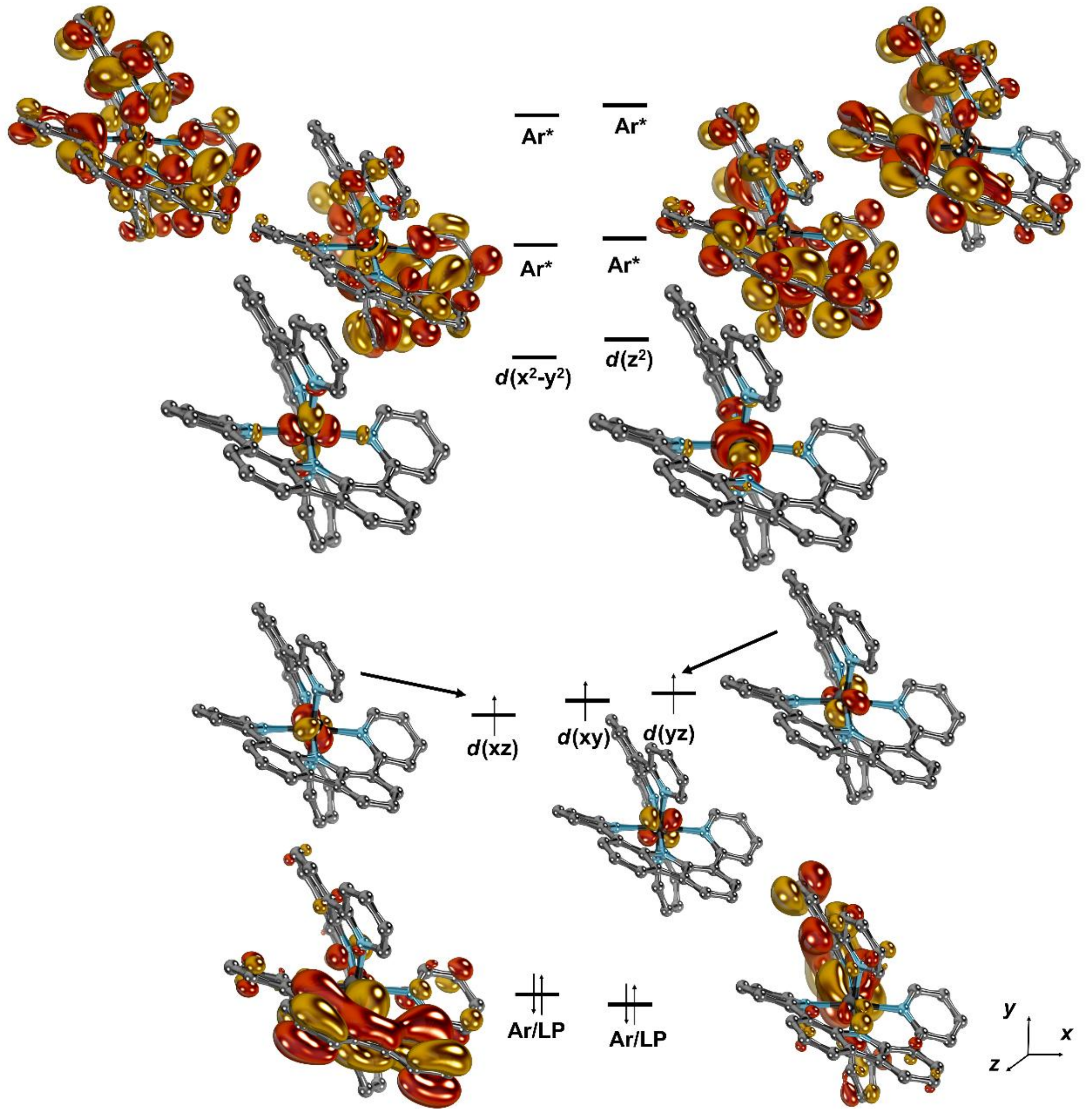

Figure S67: Molecular Orbital diagram of ***[Cr(L^{Py})$_2$]$^+$*** *(S = 3/2, 20 roots; S = ½, 40 roots) as obtained by state-averaged CASSCF(7,11) in order of decreasing occupation (and not energy) of natural orbitals. Isodensity values are adjusted for clarity.*

Table S12: Configurations of pertinent roots (NEVPT2) for Molecular Orbital diagram of ***[Cr(L^{Py})$_2$]$^+$*** *(S = 3/2, 20 roots; S = ½, 40 roots) as obtained by CASSCF(7,11) in order of decreasing occupation (and not energy) of natural orbitals.*

DOUBLETS:

ROOT 0: (*d-d* transition, **$^2T_1$/$^2T_2$**) E= 1.803 eV (vs. Q0)
0.54620 [8]: 22120000000
0.23308 [9]: 22111000000
0.12315 [16]: 22102000000
0.01744 [11]: 22110010000
0.01237 [0]: 22210000000
0.00929 [45]: 22020100000
0.00700 [51]: 22012000000
0.00670 [44]: 22021000000
0.00661 [1498]: 12111001000
0.00491 [1]: 22201000000
0.00269 [29]: 22100020000

ROOT 1: (*d-d* transition, **$^2T_1$/$^2T_2$**) E= 1.824 eV (vs. Q0)
0.66238 [0]: 22210000000
0.24090 [51]: 22012000000
0.02336 [3]: 22200010000
0.00995 [17]: 22101100000
0.00992 [1]: 22201000000
0.00940 [9]: 22111000000
0.00763 [8]: 22120000000
0.00344 [4]: 22200001000
0.00335 [81]: 22002001000
0.00334 [44]: 22021000000
0.00323 [1499]: 12111000100
0.00278 [80]: 22002010000

ROOT 2: (Spin Flip, ^{2}E) E= 1.938 eV (vs. Q0)
0.63519 [9]: 22111000000
0.18782 [8]: 22120000000
0.06844 [16]: 22102000000
0.02388 [45]: 22020100000
0.01905 [1]: 22201000000
0.01712 [44]: 22021000000
0.00612 [11]: 22110010000
0.00402 [19]: 22101001000
0.00401 [1498]: 12111001000
0.00360 [2]: 22200100000
0.00308 [79]: 22002100000
0.00274 [18]: 22101010000

ROOT 3: (Spin-Flip, **^{2}E**) E= 2.089 eV (vs. Q0)
0.91394 [9]: 22111000000
0.01896 [2]: 22200100000
0.01078 [79]: 22002100000
0.01043 [1]: 22201000000
0.00951 [44]: 22021000000
0.00926 [19]: 22101001000
0.00536 [59]: 22010110000
0.00464 [18]: 22101010000
0.00264 [8]: 22120000000

ROOT 4: (*d-d* transition; **$^2T_1$/$^2T_2$**) E= 2.098 eV (vs. Q0)
0.48915 [44]: 22021000000
0.37243 [1]: 22201000000
0.07322 [9]: 22111000000
0.01493 [10]: 22110100000
0.01073 [53]: 22011010000
0.00873 [54]: 22011001000
0.00492 [51]: 22012000000
0.00304 [0]: 22210000000

QUARTETS:

ROOT 0: (**$^4A_2$**) E= 0 eV
0.97182 [0]: 22111000000
0.01137 [9]: 22101001000
0.00355 [1127]: 12112000000
0.00302 [31]: 22011000100

ROOT 1: (*d-d* transition, **$^4T_2$**) E= 2.167 eV
0.93162 [7]: 22101100000
0.02051 [1]: 22110100000
0.01957 [28]: 22011100000
0.00702 [11]: 22101000010
0.00331 [1155]: 12102100000
0.00312 [51]: 22001100100

ROOT 2: (*d-d* transition, **$^4T_2$**) E= 2.226 eV
0.73365 [1]: 22110100000
0.18382 [29]: 22011010000
0.02325 [13]: 22100110000
0.02300 [7]: 22101100000
0.00400 [5]: 22110000010
0.00372 [14]: 22100101000
0.00349 [8]: 22101010000

ROOT 3: (LLCT/ILCT) E = 2.253 eV
0.19784 [1133]: 12111000001
0.19009 [1130]: 12111001000
0.13683 [1132]: 12111000010
0.13163 [1120]: 12121000000
0.09854 [124]: 21111000010
0.08321 [1131]: 12111000100
0.02335 [122]: 21111001000
0.01967 [1092]: 12211000000
0.01706 [123]: 21111000100
0.01266 [1129]: 12111010000
0.01107 [6]: 22110000001
0.00908 [1128]: 12111100000
0.00611 [120]: 21111100000
0.00566 [112]: 21121000000
0.00465 [1172]: 12101002000
0.00440 [4]: 22110000100
0.00438 [84]: 21211000000
0.00361 [3]: 22110001000
0.00336 [1175]: 12101001001
0.00284 [32]: 22011000010

ROOT 4: (LLCT/ILCT) E= 2.299 eV
0.40286 [122]: 21111001000
0.19572 [123]: 21111000100
0.08957 [112]: 21121000000
0.08824 [124]: 21111000010
0.07166 [1131]: 12111000100
0.02714 [125]: 21111000001
0.02421 [84]: 21211000000
0.01461 [1132]: 12111000010
0.01459 [4]: 22110000100
0.00787 [121]: 21111010000
0.00670 [164]: 21101002000
0.00417 [3]: 22110001000
0.00405 [119]: 21112000000
0.00402 [30]: 22011001000
0.00373 [1130]: 12111001000
0.00363 [5]: 22110000010
0.00327 [1092]: 12211000000
0.00264 [165]: 21101001100

ROOT 11: (*d-d* transition) E= 3.305 eV<br>0.37471 [17]: 22101100000<br>0.10986 [203]: 21111000100<br>0.09355 [1498]: 12111001000<br>0.04662 [202]: 21111001000<br>0.04040 [1501]: 12111000001<br>0.03733 [58]: 22010200000<br>0.03702 [51]: 22012000000<br>0.03612 [1499]: 12111000100<br>0.02925 [204]: 21111000010<br>0.02306 [80]: 22002010000<br>0.01903 [1488]: 12121000000<br>0.01741 [12]: 22110001000<br>0.01486 [0]: 22210000000<br>0.01295 [157]: 21211000000<br>0.01274 [192]: 21121000000<br>0.01038 [10]: 22110100000<br>0.00985 [11]: 22110010000<br>0.00823 [3]: 22200010000<br>0.00507 [52]: 22011100000<br>0.00375 [55]: 22011000100<br>0.00358 [1500]: 12111000010<br>0.00348 [15]: 22110000001<br>0.00312 [2]: 22200100000<br>0.00298 [1453]: 12211000000<br>0.00283 [14]: 22110000010	

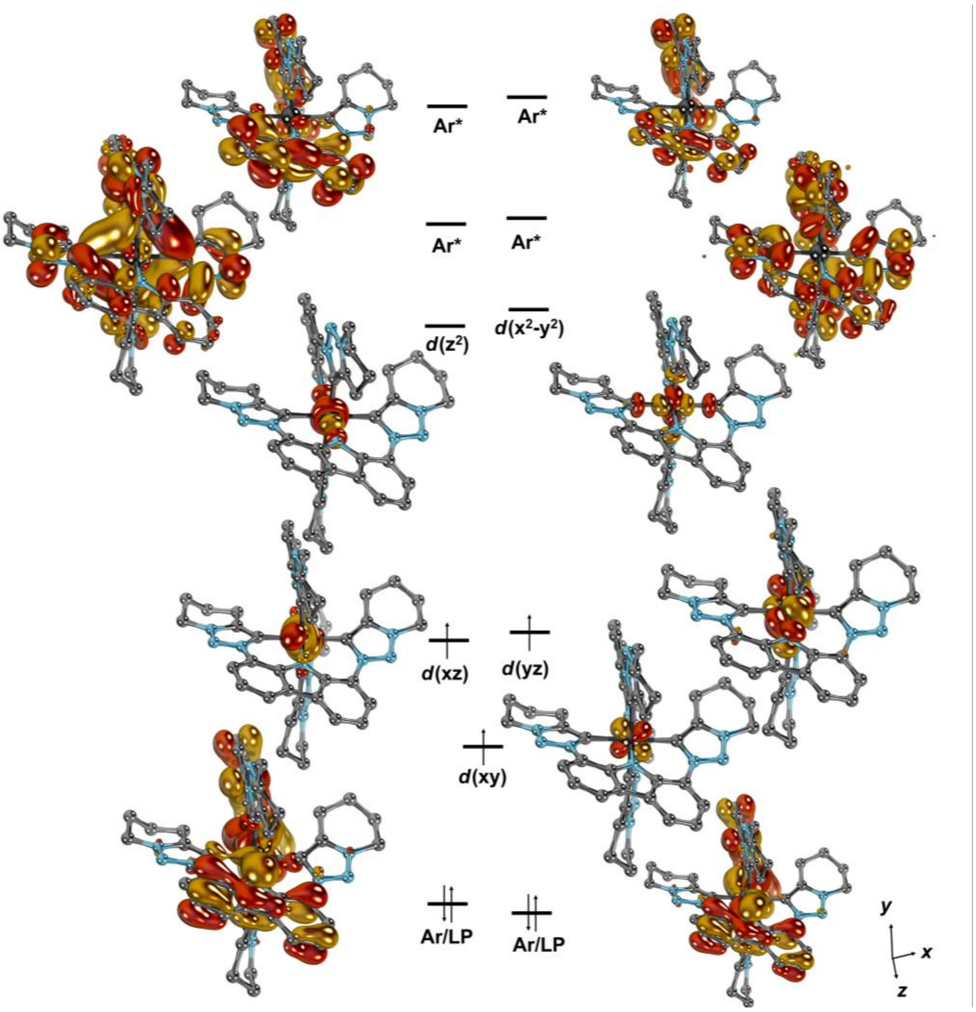

*Figure S68: Molecular Orbital diagram of **[Mn(L^{MIC})$_2$]$^{2+}$** (S = 3/2, 40 roots; S = ½, 80 roots) as obtained by state-averaged CASSCF(7,11) in order of decreasing occupation (and not energy) of natural orbitals. Isodensity values are adjusted for clarity.*

*Table S13: Configurations of pertinent roots (NEVPT2) for Molecular Orbital diagram of **[Mn(L^{MIC})$_2$]$^{2+}$** (S = 3/2, 80 roots; S = ½, 40 roots) as obtained by CASSCF(7,11) in order of decreasing occupation (and not energy) of natural orbitals. Note that the non-truncated complex features experimentally in the solid-state an S = 3/2 ground state,[3] which is calculated to be essentially isoergic (ΔE = +0.07 eV).*

DOUBLETS:

ROOT 0: (MLCT) E= 0 eV
0.33455 [1453]: 12211000000
0.27704 [0]: 22210000000
0.12101 [1488]: 12121000000
0.09446 [8]: 22120000000
0.05676 [4309]: 02212000000
0.04087 [1866]: 11221000000
0.02052 [4414]: 02122000000
0.00435 [1496]: 12111100000
0.00431 [1634]: 12011200000
0.00410 [5184]: 01222000000
0.00337 [58]: 22010200000
0.00261 [1490]: 12120010000
0.00254 [1498]: 12111001000
0.00252 [156]: 21220000000
0.00250 [199]: 21112000000

ROOT 1: (LMCT/*d-d* transition) E= 0.138 eV (*vs*. ROOT0, doublet)
0.35154 [1488]: 12121000000
0.24648 [8]: 22120000000
0.12409 [1453]: 12211000000
0.09243 [0]: 22210000000
0.06365 [4414]: 02122000000
0.04487 [199]: 21112000000
0.02234 [4309]: 02212000000
0.01532 [16]: 22102000000
0.00378 [1455]: 12210010000
0.00293 [1535]: 12101020000
0.00262 [51]: 22012000000

ROOT 2: (LLCT/ILCT) E= 0.164 eV (*vs*. ROOT0, doublet)
0.59447 [157]: 21211000000
0.19813 [1]: 22201000000
0.06575 [1873]: 11212000000
0.06039 [570]: 20221000000
0.02817 [9]: 22111000000
0.00674 [338]: 21011200000
0.00480 [200]: 21111100000
0.00422 [2748]: 10222000000
0.00371 [192]: 21121000000
0.00343 [1460]: 12202000000

ROOT 3: (LLCT/ILCT) E= 0.339 eV (*vs*. ROOT0, doublet)
0.67572 [192]: 21121000000
0.15280 [9]: 22111000000
0.08474 [1978]: 11122000000
0.02857 [44]: 22021000000
0.00972 [157]: 21211000000
0.00528 [239]: 21101020000
0.00439 [570]: 20221000000
0.00431 [1495]: 12112000000
0.00403 [233]: 21101200000
0.00397 [305]: 21021100000
0.00258 [1]: 22201000000

ROOT 4: (LLCT/ILCT) E= 0.531 eV (*vs*. ROOT0, doublet)
0.63887 [1495]: 12112000000
0.23932 [9]: 22111000000
0.03952 [1978]: 11122000000
0.03306 [192]: 21121000000
0.00605 [1508]: 12110020000
0.00592 [1873]: 11212000000
0.00454 [1628]: 12012100000
0.00448 [157]: 21211000000
0.00399 [1502]: 12110200000

QUARTETS:

ROOT 1: (**^{4}A$_2$**) E= 0.069 eV (*vs*. ROOT0, doublet)
0.20448 [0]: 22111000000
0.15115 [1127]: 12112000000
0.07660 [1613]: 11112000100
0.06639 [112]: 21121000000
0.05075 [553]: 20111010100
0.04258 [1850]: 11022001000
0.04081 [3913]: 02111010100
0.02996 [123]: 21111000100
0.02944 [1612]: 11112001000
0.01827 [1586]: 11121000100
0.01616 [1623]: 11111011000
0.01456 [30]: 22011001000
0.01258 [1155]: 12102100000
0.01195 [1142]: 12110010100
0.01155 [1162]: 12101110000
0.01094 [84]: 21211000000
0.01044 [226]: 21021001000
0.01001 [1121]: 12120100000
0.00749 [122]: 21111001000
0.00710 [161]: 21101010100
0.00681 [1129]: 12111010000
0.00654 [1582]: 11122000000
0.00630 [1473]: 11220001000
0.00569 [541]: 20112001000
0.00546 [1255]: 12012001000
0.00521 [2675]: 10112010100
0.00490 [1253]: 12012100000
0.00484 [133]: 21110011000
0.00451 [1260]: 12011110000
0.00444 [1479]: 11211010000
0.00438 [252]: 21011110000
0.00432 [1131]: 12111000100
0.00389 [7]: 22101100000
0.00365 [3901]: 02112001000
0.00351 [1134]: 12110200000
0.00313 [124]: 21111000010
0.00291 [1588]: 11121000001
0.00258 [9]: 22101001000
0.00256 [4704]: 01121010100

ROOT 2: (**^{4}A$_2$ – mixes with LLCT/ILCT**) E= 0.169 eV (*vs*. ROOT0, doublet)
0.30202 [0]: 22111000000
0.25073 [112]: 21121000000
0.13489 [1128]: 12111100000
0.08292 [1127]: 12112000000
0.07013 [1]: 22110100000
0.05270 [1582]: 11122000000
0.03711 [84]: 21211000000
0.01996 [3899]: 02112100000
0.00980 [1583]: 11121100000
0.00796 [1477]: 11212000000
0.00265 [159]: 21101020000

ROOT 3: (ILCT/LLCT) E= 0.605 eV (*vs*. ROOT0, doublet)
0.55052 [1092]: 12211000000
0.34241 [1120]: 12121000000
0.03466 [1470]: 11221000000
0.01598 [1127]: 12112000000
0.00851 [112]: 21121000000
0.00764 [119]: 21112000000
0.00759 [1259]: 12011200000
0.00616 [1128]: 12111100000
0.00328 [1585]: 11121001000
0.00311 [1122]: 12120010000
0.00304 [1161]: 12101200000

	ROOT 4: (ILCT/LLCT) E= 0.690 eV (*vs*. ROOT0, doublet)<br>0.44917 [1127]: 12112000000<br>0.28075 [112]: 21121000000<br>0.14177 [119]: 21112000000<br>0.04419 [0]: 22111000000<br>0.03263 [84]: 21211000000<br>0.01419 [1092]: 12211000000<br>0.00373 [1140]: 12110020000<br><br>ROOT 5: (ILCT/LLCT) E= 0.719 eV (*vs*. ROOT0, doublet)<br>0.52764 [1120]: 12121000000<br>0.35870 [1092]: 12211000000<br>0.07345 [119]: 21112000000<br>0.00438 [1094]: 12210010000<br>0.00402 [1167]: 12101020000<br>0.00395 [1470]: 11221000000<br>0.00350 [1232]: 12021100000<br>0.00264 [1259]: 12011200000

Comparison of Ligands (NHC, py) and Metal (Mn): AILFT

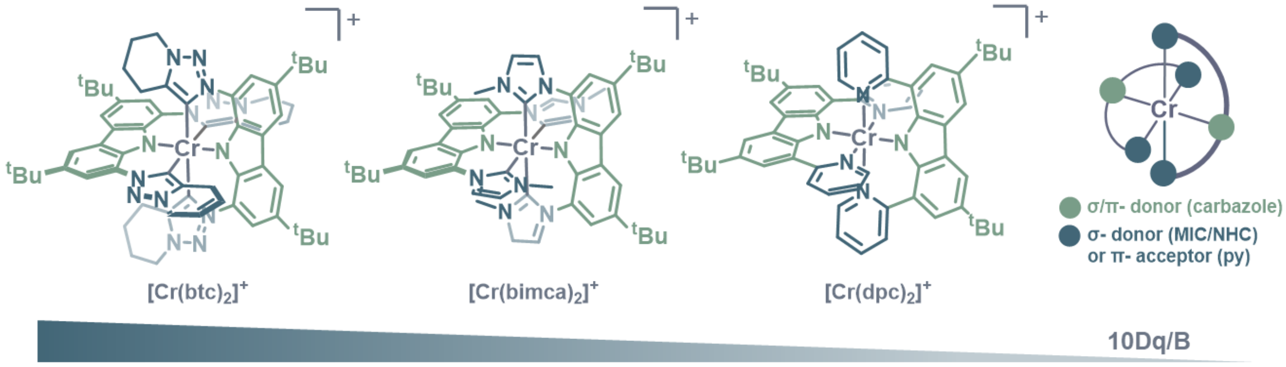

Table S14: Ab-Initio Ligand Field (AILFT) comparison of MIC, NHC and py ligands as well as comparison with MIC-Mn complex as obtained at the NEVPT2//CASSCF(3,5) level of theory. Energies are given in [cm^{-1}].

**[Mn(L^{MIC})$_2$]$^{2+}$**	***d*(xz)**	***d*(yz)**	***d*(xy)**	***d*(x^2-y^2)** **C-C-C-C**	***d*(z^2)** **N-Mn-N**	**10 Dq** **(1st transition)**
	0.0	281.0	2633.0	32798.4	35795.2	35155.4
**[Cr(L^{MIC})$_2$]$^+$**	***d*(xy)**	***d*(yz)**	***d*(xz)**	***d*(z^2)** **C-Cr-C**	***d*(x^2-y^2)** **N-C-N-C**	**10 Dq** **(1st transition)**
	0.0	537.5	1317.6	23608.8	24154.8	25102.6
**[Cr(L^{NHC})$_2$]$^+$**	***d*(xy)**	***d*(yz)**	***d*(xz)**	***d*(x^2-y^2)** **C-C-C-C**	***d*(z^2)** **N-Cr-N**	**10 Dq** **(1st transition)**
	0.0	1644.8	3479.4	23604.5	25586.4	24558.2
**[Cr(L^{Py})$_2$]$^+$**	***d*(xy)**	***d*(yz)**	***d*(xz)**	***d*(z^2)** **N-Cr-N**	***d*(x^2-y^2)** **py-py-py-py**	**10 Dq** **(1st transition)**
	0.0	634.0	3217.5	15589.0	23413.9	17866.3

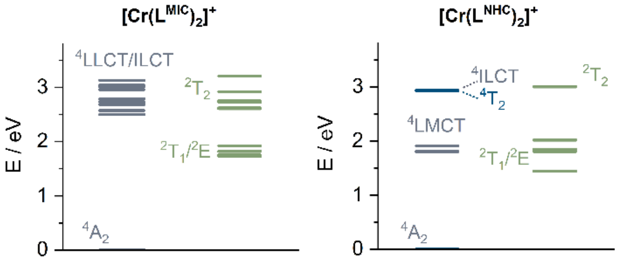

Figure S69: NEVPT2/CASSCF calculated quartet and doublet state energies with their character assignment for $[Cr(L^{MIC})_2]^+$ and $[Cr(L^{NHC})_2]^+$.

XYZ Coordinates

Truncated Systems used for CASSCF

107

$[Cr(L^{MIC})_2]^+$_r2SCAN3c_truncated.xyz

24	0.000000000	0.000000000	0.000000000
7	-0.092425272	-0.172861905	-4.329088293
7	-0.076112104	2.016066112	0.065631802
7	-0.239796928	1.289058681	-2.863888658
7	0.068852075	-2.017359992	-0.077715820
7	-0.299859625	1.115777376	-4.186416425
7	2.941299325	-1.113975125	0.138295535
7	0.149367902	1.111035910	2.940568903
7	-2.849684097	-1.290491715	-0.363729842
7	-4.317929514	0.173736559	-0.294417039
7	4.314154560	0.426788906	-0.078356191
6	0.106574634	-0.818518945	-3.144654293
7	0.104140514	-0.449753346	4.307325079
6	-0.234175107	4.237072774	-0.574304425
7	4.253838870	-0.874522562	0.084994734
7	0.218857063	0.856901095	4.249231683
6	-0.403298471	5.286584110	-1.474040488
1	-0.412490503	6.312229719	-1.113416949
6	-0.221453162	2.878477507	-0.994657847
6	-0.524427421	3.677724193	-3.251612432
1	-0.622348505	3.455586069	-4.307632193
6	-0.563825216	5.017487520	-2.830958221
7	-4.165715897	-1.113714068	-0.501771694
6	-0.346643117	2.619341695	-2.366849441
6	0.844736507	-4.198554382	0.028815777
6	-0.005914680	0.142746497	-2.133621386
6	0.018210239	2.813072943	1.182650341
6	-0.569188799	-4.240904030	-0.209976363
6	-2.132022586	-0.147029432	-0.081086799
6	-0.986327394	-2.881611867	-0.249923689
6	-0.061405830	4.197544346	0.849253300
6	-3.145984248	0.815456876	-0.021546193
6	1.803898570	-5.186222063	0.207735925
1	1.514872133	-6.233994918	0.175775977
6	1.178175108	-2.813140932	0.077538726
6	2.137909308	0.000000000	0.007727236
6	0.000000000	0.000000000	2.136149179
6	3.475274675	-3.476290492	0.427251894
1	4.510859721	-3.187196917	0.561768341
6	0.393273922	-2.285935541	-3.139887827
1	-0.495715167	-2.826193690	-2.789223755
1	1.183382320	-2.497328939	-2.415063306
6	-0.045433998	-1.029676141	3.082459569
6	2.525449634	-2.471883696	0.234555877
6	3.087301916	1.019166986	-0.130555953
6	-0.045743900	-0.744838346	-5.681873101
1	0.911594897	-0.455295582	-6.133403502
1	-0.851492613	-0.287846416	-6.263753191
6	0.162232583	2.474535367	2.532311976
6	-0.167237008	-2.259022063	-5.592808424
1	0.054969414	-2.679784335	-6.578500629
1	-1.199448647	-2.538701404	-5.344370884
6	3.137841089	-4.835189397	0.431008273
6	0.032463972	5.188654322	1.817270798
1	-0.029037049	6.235149268	1.528475034
6	0.788794877	-2.795867820	-4.529455894
1	1.814250820	-2.481601978	-4.767852397
1	0.783706125	-3.889956788	-4.524558896
6	-2.351637184	-2.622603411	-0.435216212
6	-5.668545223	0.751219751	-0.336917672
1	-6.185656397	0.460791109	0.586393814
1	-6.195913340	0.299066274	-1.181959302
6	-3.155588945	2.280313373	0.277144483
1	-2.745421319	2.826289859	-0.582126840
1	-2.484314561	2.483591818	1.115631083
6	2.983829446	2.492493203	-0.365950038
1	2.247304916	2.921944016	0.314991144
1	2.594009285	2.657226133	-1.379947847
6	-1.463327395	-5.292319016	-0.396270059
1	-1.106309198	-6.318765986	-0.364902580
6	0.207675052	4.842151682	3.159064189
6	-4.566719336	2.793364848	0.581201451
1	-4.876222423	2.476343174	1.586599098
1	-4.557248374	3.887472606	0.581192227
6	0.263046152	3.483358305	3.491432263
1	0.375843478	3.196829974	4.530110272
6	-5.563923627	2.265063161	-0.448047188
1	-5.241456528	2.545343375	-1.459416087
1	-6.560475627	2.690707999	-0.294258450
6	5.626509477	1.079015683	-0.170194170
1	6.292575549	0.402956645	-0.713703489
1	6.015760211	1.202050298	0.848624872
6	0.093355092	-1.109441707	5.620158511
1	0.864882502	-0.637582722	6.235614737
6	5.462733965	2.428110745	-0.854048537
1	6.411464998	2.969151270	-0.781722686
1	5.250585913	2.281848832	-1.921363025
6	4.328434721	3.207603763	-0.192695893
1	4.255331740	4.214594972	-0.614370005
1	4.547505475	3.326745509	0.877168047
6	-2.810257321	-5.023844600	-0.627151782
6	-3.229991832	-3.683138104	-0.631729941
1	-4.280789160	-3.462331758	-0.777442812
6	-0.595375113	-3.133754881	4.331648357
6	-0.229023699	-2.509494356	2.981337146
1	0.696543759	-2.964514821	2.605844658
1	-0.999553459	-2.726452659	2.237344073
1	-0.882534149	-0.914701120	6.082687954
1	-1.640845233	-2.905582772	4.580836462
1	-0.515739030	-4.222422632	4.256673851
6	0.317693286	-2.603096097	5.435166262
1	1.367431500	-2.795694844	5.177559747
1	0.120830389	-3.100497244	6.390065700
1	-0.703661658	5.811225377	-3.554806065
1	0.289151239	5.597179353	3.931580956
1	3.901871435	-5.587544230	0.585037779
1	-3.529072172	-5.819071414	-0.783370424

107

$[Mn(L^{MIC})_2]^{2+}$_r2SCAN3c_truncated.xyz

25	0.000000000	0.000000000	0.000000000
7	0.029464360	0.022286650	-1.938212250
7	2.868009713	0.300195034	-1.098857644
7	0.000000000	0.000000000	1.939091684
7	-0.429461302	-2.818459467	1.143365752
7	-2.830018636	-0.218837776	-1.185709937
7	0.430460903	2.825067858	1.129799151
7	4.260275944	0.018500940	0.398927223
7	-4.239635909	0.017970638	0.308490884
7	0.060750810	4.240785773	-0.328811638
7	-0.523785455	-4.124954250	0.926737289
7	-0.287258951	-4.224357823	-0.359617849
7	0.458438083	4.138237889	0.922352191
7	-4.151295196	-0.220483266	-0.978132232
7	4.181610334	0.303115896	-0.884378623
6	2.093027675	0.000000000	-0.007430677
6	0.026672521	2.078735522	0.052454400
6	-0.120255128	-2.068771009	0.036981979
6	1.109848213	0.292663464	-2.762217459
6	0.344370630	1.057022404	2.776125958
6	-2.074635417	0.018441990	-0.066495478
6	1.289154364	3.165460343	3.396892700
1	1.610051984	4.169530016	3.098536023
6	0.752852424	0.309093532	-4.128915272
6	-1.037952200	-0.152727766	-2.806901428
6	-3.266442072	-0.625683943	-3.558979005
1	-4.316386543	-0.808215468	-3.307278752
6	1.670336513	0.710389976	-5.096869680
1	1.367165058	0.735413720	-6.151513670
6	-3.047676355	0.177129415	0.923091530
6	-0.022947412	-3.034345347	-0.959244616
6	1.409393642	2.763571436	4.732678127
6	-2.381058346	-0.369554551	-2.526237334
6	3.313625990	1.011521281	-3.390015990
1	4.337320986	1.249925666	-3.083799075
6	-0.235576152	3.045954092	-0.905707166
6	0.716399073	2.350025639	2.439665874
6	0.875874842	1.522946394	5.104741804
1	0.906454597	1.200884447	6.152937741
6	-0.254210767	-1.053749061	2.796607977
6	2.429441501	0.556830921	-2.425344542
6	3.051605384	-0.188514545	0.986343177
6	-0.580322423	-2.368817348	2.482665386
6	-0.986521935	-2.835430895	4.839202513
6	0.346206961	0.679638468	4.128708483
6	-1.535556733	-0.278753326	-5.195148966
1	-1.202942854	-0.206197440	-6.238013738
6	-0.132147454	-0.687756163	4.149571676
6	-0.985772909	-3.233887251	3.487793119
1	-1.273677625	-4.254975559	3.218312992
6	-0.495825742	-1.569062392	5.165379326
1	-0.423702036	-1.259083960	6.215221614
6	2.939387915	1.128106644	-4.731375252
6	-2.867654484	-0.604915977	-4.901399726
6	-0.638400735	-0.055721074	-4.160306141
6	5.592403434	-0.032907037	1.019474450
1	6.178590841	0.800272846	0.598205315
1	6.070912924	-0.978201336	0.702123265
6	-0.468771765	-5.393186474	-2.479472682
1	-1.490037528	-5.048334261	-2.734243687

```
1	-0.346249934	-6.382922575	-2.952514235
6	-0.786605908	3.017268757	-2.289010099
1	-0.024221284	2.531161284	-2.930798283
1	-1.650647327	2.332658975	-2.316999607
6	3.003415699	-0.544856490	2.432458895
1	2.259958863	-1.343357009	2.593107808
1	2.637139105	0.329285777	3.000839055
6	4.372331254	-0.948899962	2.974144548
1	4.325636998	-0.994036348	4.075444609
1	4.636738389	-1.969620443	2.633617016
6	5.441500992	0.034830926	2.518762159
1	5.171679799	1.062854721	2.830646441
1	6.417785337	-0.187627261	2.982843592
6	-0.333281659	-5.556421906	-0.982030001
1	-1.174205213	-6.100660404	-0.521996145
1	0.601274284	-6.083589638	-0.714109589
6	-5.582098800	0.106664864	0.911581379
1	-6.210269591	-0.648395011	0.411536380
1	-5.990488613	1.105673589	0.670317038
6	-3.005828219	0.459348504	2.395592556
1	-2.267846666	1.254094448	2.597335848
1	-2.625424321	-0.442106220	2.911048987
6	0.292826006	-3.008127271	-2.423528973
1	1.143749351	-2.328685170	-2.601720465
1	-0.564820400	-2.559476886	-2.958747695
6	-5.455361660	-0.099284819	2.399656839
1	-5.212422427	-1.157445545	2.619205655
1	-6.433761955	0.100801970	2.869803355
6	-4.378687585	0.820593933	2.955989490
1	-4.345216814	0.763866899	4.057249998
1	-4.629815935	1.871286492	2.709801472
6	0.566436933	-4.401544033	-2.991101455
1	0.553266800	-4.351965606	-4.092920426
1	1.581412917	-4.742400213	-2.706809825
6	0.007911639	5.571859476	-0.951030827
1	-0.739359027	6.145259818	-0.369156526
1	0.983776162	6.056771575	-0.785639563
6	-0.341919087	5.452427199	-2.369595459
1	-0.790219664	6.410237281	-2.691965156
1	0.620916237	5.431386692	-2.928483001
6	-1.134067770	4.352058900	-2.826221217
1	-1.133557178	4.318312434	-3.929362533
1	-2.200444983	4.542761589	-2.570222930
1	1.868127404	3.428063664	5.471351714
1	-1.314419290	-3.533351989	5.615890282
1	3.655785252	1.490125170	-5.475343067
1	-3.591844578	-0.808291010	-5.696175127
83
```

[Cr(L^{NHC})$_2$]$^+$ _r2SCAN3c_truncated.xyz

```
24	0.000000000	0.000000000	0.000000000
7	0.000000000	0.000000000	2.002286901
7	0.008843982	0.038965427	-2.003956850
7	-2.851249469	-0.551845245	-1.235855337
7	-0.256408966	-2.907661969	1.210600102
7	-0.591682590	3.037007530	-0.824228789
7	0.111171407	2.911399095	1.197530608
7	-3.101278137	0.061687025	0.798414161
7	2.778223589	0.902008173	-1.170168297
7	0.254311773	-3.085567869	-0.855732063
7	3.058194941	0.382647583	0.880914256
6	-0.105761922	-1.089848629	2.857337974
6	0.755652101	0.096684803	-4.197837194
6	-0.201619528	2.114176510	0.115046280
6	-0.589615982	-0.394166678	-4.196391794
6	-2.303443391	-0.762531523	-2.547379437
6	0.062898829	-0.708051065	4.223822775
6	1.071824479	0.339740960	-2.844094810
6	0.242191844	1.076877216	2.838975872
6	-0.273858771	-2.438999400	2.573287108
6	0.096817859	-2.153074919	0.123632211
6	0.783526092	3.305980667	3.506189336
1	0.979367890	4.344178547	3.263675003
6	-1.007050496	-0.380860636	-2.852472990
6	2.354156564	0.777958870	-2.535491420
6	0.317125456	0.701309019	4.196948602
6	-1.431195129	-0.860219557	-5.217780939
1	-1.077598756	-0.866470004	-6.245740321
6	0.646774899	1.621349584	5.200678244
1	0.706711250	1.293820513	6.235285552
6	2.132507051	0.320951275	-0.109500094
6	0.924080888	2.919538065	4.858554026
6	-0.013540084	-1.646179837	5.258398676
1	0.112600096	-1.325271220	6.289201372
6	1.684819079	0.290459922	-5.225748126
1	1.405318426	0.082358568	-6.255080113
6	-2.130133172	-0.172219052	-0.126510192
6	-3.116766269	-1.249377140	-3.561675597
1	-4.116339892	-1.598691954	-3.331587657
6	-2.691568299	-1.320552706	-4.910596319
6	0.411016302	2.428070227	2.510651261
6	2.950515517	0.741276263	-4.916627199
6	-0.237978602	-2.977689925	4.961512796
6	3.266981684	0.965642562	-3.563239652
1	4.282499703	1.264113184	-3.332531671
6	-4.217194894	-0.551822233	-0.980409121
1	-4.964469591	-0.777578568	-1.719894324
6	-4.357537112	-0.163665456	0.292315423
1	-5.249364790	-0.025162052	0.883951525
6	-0.353166679	-3.353023785	3.612154504
1	-0.475958131	-4.407386659	3.401101744
6	4.197975809	1.013276122	0.474469234
1	5.037615833	1.165523686	1.136066656
6	4.041278364	1.344719620	-0.800182255
1	4.709509129	1.861460686	-1.466680025
6	-0.353529744	-4.247465798	0.876260756
1	-0.635579421	-5.019172893	1.569737243
6	-0.024422025	-4.349118112	-0.414456763
1	0.035538847	-5.214450111	-1.056540548
6	-0.533869431	4.321072627	-0.340740089
1	-0.814429290	5.174414975	-0.938764100
6	-0.093322697	4.255050007	0.918884472
1	0.046054031	5.040332249	1.640416222
6	-1.095145362	2.763890417	-2.166160621
1	-0.286457351	2.488135106	-2.846774979
1	-1.578240123	3.671453638	-2.534664881
1	-1.831928869	1.961393190	-2.140156541
6	-2.927677887	0.552870456	2.162095612
1	-2.508130160	-0.219555528	2.810892533
1	-3.907839369	0.847081699	2.542068731
1	-2.269799029	1.422619204	2.172739381
6	0.764713644	-2.877651278	-2.205172470
1	-0.045761453	-2.696300929	-2.915671128
1	1.308058369	-3.777574517	-2.503388561
1	1.452125357	-2.033831109	-2.216905033
6	3.002811197	-0.290637479	2.170668549
1	2.698450823	0.393405330	2.968029492
1	3.998036743	-0.683843254	2.393531729
1	2.300345282	-1.120377823	2.121519043
1	3.702491771	0.900174335	-5.680280334
1	-3.360437538	-1.710727601	-5.668146416
1	-0.304491248	-3.728838660	5.739455025
1	1.217221399	3.655598579	5.597770453
79
```

[Cr(L^{Py})$_2$]$^+$_r2SCAN3c_truncated.xyz

```
24	0.000000000	0.000000000	0.000000000
7	0.000000000	0.000000000	1.962526678
7	2.121013865	0.094510486	-0.207941071
7	-0.160514812	-0.145487300	-1.957420150
7	0.216329633	2.121548044	0.043350604
7	-0.032008348	-2.105315401	0.130911644
7	-2.114168796	0.069534504	0.038812824
6	0.200279726	1.086420207	2.798798327
6	-1.740900825	-2.974262590	3.332031522
1	-2.144536806	-3.946588695	3.074978989
6	0.874059113	-0.108141988	-2.879416176
6	-1.962682340	-2.532171055	4.641273586
6	-0.771657862	-0.518198567	4.076989255
6	-0.670510922	-2.852357281	1.069898719
6	2.801488784	0.466700240	-1.329772617
6	-0.600186447	-0.968587858	2.739751655
6	-1.042943773	-2.244773782	2.361526705
6	0.671065024	2.368310313	2.452147067
6	0.803104992	2.825636292	1.055007329
6	-1.448147316	-1.291995740	5.009468162
1	-1.583968739	-0.923602628	6.023754845
6	-0.187198915	0.804074992	4.121470800
6	0.576180566	2.958517013	4.855386942
6	-1.232518919	-0.723090080	-2.612907300
6	2.212370905	0.238077456	-2.663527248
6	0.846562936	3.265106764	3.510290840
1	1.168265330	4.278479239	3.298716311
6	0.119669011	2.714528515	-1.174940896
1	-0.411055162	2.146811568	-1.930054460
6	1.456877064	4.030266401	0.776817838
1	2.013689810	4.530076513	1.558166562
6	0.020105904	1.719314510	5.150231668
1	-0.259756355	1.467650399	6.169995771
6	0.493550763	-2.751979575	-0.942398790
1	1.062836789	-2.146457026	-1.636271903
6	0.337417006	-4.092028094	-1.179785573
1	0.783603998	-4.528459172	-2.066660492
6	2.793670872	0.086823627	0.975007298
1	2.228151441	-0.262861109	1.829624073
6	3.062111212	0.171163129	-3.791371671
```

1	4.117696528	0.383559834	-3.673428185
6	-0.924911676	-0.997519451	-3.966883347
6	2.639731920	-0.238200466	-5.058524309
6	-2.945121727	-0.640887908	-0.766953733
6	0.444439980	-0.582158452	-4.145186540
6	-2.493117359	-1.075058537	-2.092779090
6	4.105115619	0.476628535	1.118830386
1	4.578215187	0.426918125	2.092876172
6	-0.874628123	-4.221951957	0.849043558
1	-1.385491600	-4.814380421	1.594576582
6	4.106808422	0.956441702	-1.210681466
1	4.611761957	1.340083743	-2.086960675
6	1.313070854	-0.638446540	-5.221379983
1	0.967970272	-1.008436121	-6.183738176
6	-2.640492171	0.698916001	1.116728141
1	-1.966787791	1.329116971	1.684375940
6	-1.846046111	-1.606983567	-4.811018351
1	-1.587716585	-1.795180981	-5.850359003
6	0.671277973	3.936341760	-1.470682327
1	0.555747172	4.349876581	-2.466391167
6	-0.393069503	-4.841471026	-0.283032025
1	-0.563258045	-5.902090289	-0.440989940
6	4.761939300	0.962567923	-0.003077945
1	5.778046996	1.340063816	0.063761965
6	-3.365344067	-1.738140635	-2.973246571
1	-4.338174877	-2.053287176	-2.616663098
6	1.410791382	4.583390463	-0.487459134
1	1.926957835	5.515096044	-0.698070840
6	-3.079517444	-2.012085415	-4.308545035
6	-4.270704813	-0.856776263	-0.365618558
1	-4.927983223	-1.463832943	-0.970665476
6	-3.948500646	0.563946720	1.501844025
1	-4.302269081	1.092906876	2.379989638
6	-4.762340302	-0.284347098	0.782146407
1	-5.788812141	-0.466294159	1.086229127
1	0.743226691	3.701387397	5.626146377
1	-2.515762895	-3.145877010	5.341876094
1	-3.806732673	-2.515138056	-4.934370917
1	3.344704219	-0.283171977	-5.880184304

Non-truncated Systems used in DFT

155

[Cr(L^{MIC})$_2$]$^+$_camB3LYP.xyz

Cr	3.68797615124547	12.49707214494685	12.00045020964063
N	3.35455227230508	11.40107245282449	7.83638637949063
N	5.69943632239704	12.46029766695154	12.00630749291406
N	4.86493136948479	11.80413247270911	9.17831557368000
N	1.67522354776978	12.53423958046204	11.99561168888056
N	4.64430771313951	11.50963857448974	7.91638915028785
N	2.44768672591939	9.76931656977123	12.93150357138250
N	4.87630737748131	13.13587119259586	14.83288569704790
N	2.55520352395722	15.24104313535482	10.98095706994937
N	4.08492668808784	16.55791996838647	10.56913634466553
N	3.90850320622329	8.33109009709285	13.13699723928896
C	2.73311293408357	11.61273124241932	9.02836557367958
N	3.37806218012072	13.60185857582125	16.16785917341211
C	7.89357918445794	12.26445774916382	11.30885517385783
N	2.62918375513618	8.50787852656918	13.25331537959313
N	4.66211050537175	13.43813932678503	16.09356798340390
C	8.91476621805835	12.07614788927668	10.37827967009267
H	9.94781086873189	12.12758939611117	10.70951076150381
C	6.52576872064297	12.21910331894848	10.94202285574448
C	7.24804381555170	11.76167026084252	8.68654730992145
H	6.97188348187199	11.55059000091652	7.65935783457719
C	8.60642554555346	11.83025385569057	9.04463102488950
N	2.79393155634129	16.44896733906381	10.52110871745347
C	6.22120562849422	11.94091098307611	9.60136173021445
C	-0.53260018044288	11.92912353761242	12.31770867654329
C	3.73778656510843	11.90324933333113	9.95332213976591
C	6.52618983235897	12.65298346991545	13.08226025740088
C	-0.50751293584695	13.25663052697584	11.75945535961948
C	3.67153664068808	14.52694352708782	11.33642505055583
C	0.86621315805957	13.56630004601837	11.60014174652985
C	7.89606544613627	12.53201032778355	12.72447886569768
C	4.68974088309034	15.44174107276415	11.05947219253043
C	9.67017368014634	11.62816003668390	7.96074619692944
C	-1.56292083088515	11.08824254826121	12.72167994845934
H	-2.59303226936153	11.42786757631862	12.63354680440257
C	0.83085844008723	11.54336495998004	12.42022287526901
C	3.59143473258655	10.45079873534782	12.60005003682159
C	3.74927961063397	13.08605423526868	14.05120154787991
C	0.06501095169669	9.43174045080685	13.30819612071330
H	0.32605562040766	8.44791263043318	13.67617147010452
C	1.24127969670684	11.51474416136293	9.11671873932492
H	0.82295447637643	12.52348738235781	9.21744067365535
H	0.96627542066669	10.98264260766006	10.02904179702826
C	2.75405801253853	13.41852436775872	14.97249387607928
C	1.10760574074534	10.25881878109140	12.90165859894888
C	4.56318082652215	9.45845944388967	12.74728034314423
C	2.73719152746445	11.04308823426787	6.55993677598187
H	2.90503119356510	9.97177062415641	6.39680978210698
H	3.26557929731917	11.59169431423062	5.77563127046377
C	6.22707753984355	12.93547683504080	14.41979300103506
C	1.25165942324485	11.36178019613876	6.60342454103801
H	0.77582054417807	10.92300317487246	5.71995570640212
H	1.10195738006831	12.44828607232377	6.54102653654142
C	-1.27908470809945	9.82335460020794	13.23734713417406
C	8.91157315425832	12.65164731707268	13.66709986342617
H	9.94686510516355	12.53889140063957	13.35147395294985
C	0.63664391423915	10.82552415662737	7.89283160291400
H	0.80288473894888	9.74026758148497	7.95089710725780
H	-0.44789152486804	10.97489402934127	7.89344919472350
C	1.19230809539475	14.83340858432343	11.09468501493486
C	4.71908726979218	17.81217488758816	10.16438138281182
H	4.56008585721602	18.53980495010011	10.96927145223801
H	4.19481018686345	18.17159896286051	9.27488617311930
C	6.18189274794785	15.40023651007593	11.17882868675216
H	6.58422585087318	14.76032304715158	10.38434446031226
H	6.45812709070981	14.92221283936602	12.11993434628323
C	6.04022115703170	9.41282449123328	12.50107110486677
H	6.51333134925683	10.28735526276897	12.94641112633696
H	6.21524082903643	9.49146079554315	11.42054389646478
C	-1.51338008489449	14.15244194112829	11.39999935264957
H	-2.55154526717571	13.86464655198085	11.53706524825445
C	8.60544985266041	12.90230737303536	15.00446344118481
C	6.80600333019500	16.79265218031303	11.08085433140621
H	6.64743491142496	17.34332700609607	12.01898612280380
H	7.88918598750858	16.69459002153128	10.95592867089365
C	7.25472383624311	13.04968787392361	15.35049281886255
H	6.97564183361756	13.26408347005999	16.37394477500317
C	6.20224792221828	17.58272617146275	9.92349180779616
H	6.34708305315578	17.03593568197493	8.98186308847269
H	6.69019175961434	18.55611833786440	9.80548840420443
C	4.49109270959673	7.02346001314325	13.43289482645279
H	3.77439402090041	6.26672093400191	13.10362342954709
H	4.59832436286203	6.93951769591045	14.52123844453383
C	9.72822703566493	12.99031447145269	16.04494858402339
C	2.77189429688699	13.98501979145377	17.44234102479382
H	3.27100828623029	13.41097489567997	18.22762903694715
C	5.84052983143674	6.90526317350117	12.74414056021679
H	6.33180287198699	5.98993666192213	13.09111523545961
H	5.69681257563651	6.80066599281648	11.65987973865051
C	6.68733383507430	8.13797677293522	13.04557822819674
H	7.68926118123845	8.03185569622053	12.61712641227082
H	6.82159956547186	8.22507381942998	14.13303557897787
C	-1.18383793870674	15.39535694660347	10.87036897212299
C	0.17998100686193	15.71116135724094	10.73528208658190
H	0.47196295565322	16.67987070677083	10.34497847599195
C	-2.42116394266190	8.91007483900280	13.69802243466679
C	0.69005893006864	14.29859068455311	16.09987027108902
C	1.26832102638846	13.57959272469072	14.88056874642766
H	0.80761095744357	12.58922647684766	14.78458608378026
H	1.01753309439089	14.11941242188893	13.96620150638424
C	10.44863431689851	11.63228764154637	16.11738008878337
H	10.89283115567852	11.35717416063703	15.15449245943201
H	9.75184986455032	10.83586990054805	16.40423205390022
H	11.25498570004430	11.66271182904149	16.86015231326818
C	10.73205988425282	14.08043672985507	15.63481360138157
H	10.24648635959485	15.06199936119586	15.58505865919452
H	11.17969691512619	13.87824427024717	14.65595568189178
H	11.54841622229935	14.14280697430863	16.36439271842776
C	-2.23242847314030	16.42425886157610	10.43602921090660
C	-2.04430505792414	17.72338195842570	11.23765169685470
H	-2.16254404515570	17.54011437670304	12.31201807971134
H	-2.78901566514032	18.47011013056372	10.93704398481884
H	-1.05336194425752	18.16279594012012	11.07954707099074
C	-3.65946355180612	15.91906781708840	10.67133071565408
H	-3.86931309764677	15.00659517733662	10.10138642766517
H	-4.37960636753199	16.67981862475769	10.35035137083245
H	-3.84880638581977	15.71409140399518	11.73130780521904
C	9.19802334564584	13.33007529492764	17.44198398217459
H	8.50608289004867	12.56613479215025	17.81463382156583
H	8.68360907916144	14.29791954499868	17.45883193044216
H	10.03291284005530	13.38855440428649	18.14914460839504
H	2.98835743546333	15.04707776946498	17.60853726540144
H	0.90642782786610	15.37493511180055	16.04114295525994
H	-0.40019532713069	14.19893602441208	16.09371730727329
C	-1.91224763622255	7.55208969268860	14.19237657060933
H	-1.38126084766655	7.00470342090206	13.40485851691159
H	-2.75903928762899	6.93325815147180	14.50923387486595
H	-1.24085131703274	7.65517110069339	15.05265993894114
C	-3.38603628246357	8.66424930644751	12.52579287277037
H	-3.82266664058877	9.59703873454150	12.15325870868461
H	-4.21185267399778	8.01432683575142	12.83923733135459
H	-2.87038480406097	8.17812615743046	11.68933905990052

C	-3.17883863585329	9.59257652885145	14.84982446619573
H	-2.51434147027470	9.76979364938634	15.70364997628389
H	-4.00979077569575	8.96316471590743	15.19079590643393
H	-3.59647212136574	10.55824210783860	14.54488566803383
C	-2.06313568187084	16.71778480326803	8.93564796317668
H	-1.06904742621607	17.11860813360614	8.70894851429302
H	-2.80299209866857	17.45533417819951	8.60229258693181
H	-2.20086101771807	15.80662070413067	8.34160360564923
C	1.27332307792622	13.73598410173248	17.39261055461977
H	1.07232086834071	12.65780720435863	17.45517618408497
H	0.81471344510202	14.19765389530852	18.27354110415103
C	9.49851870161117	10.23861961450273	7.32340498502559
H	9.61185807653803	9.44731388274580	8.07366133018805
H	10.25310909025928	10.07782491253506	6.54403916846903
H	8.51349808346171	10.12131361400407	6.85867472867027
C	9.50418210790072	12.71175288490556	6.88156572539380
H	8.52057005760467	12.66039629852531	6.40186674784164
H	10.26233988582098	12.59479427652828	6.09787183166092
H	9.61420443927587	13.71299600453863	7.31449358163664
C	11.08951707639627	11.72464948664808	8.52855457455738
H	11.28589299349787	12.70782634257785	8.97169485188637
H	11.82063999397766	11.57332793840367	7.72664145382282
H	11.27598827159145	10.96006749064664	9.29151603274859

155

[Cr(L^MIC)₂]⁺_d_PBE0.xyz

Cr	3.68878834243712	12.49238709911110	12.00385778182834
N	3.32798694842098	11.47062295822204	7.84836053523041
N	5.67982855140526	12.45410010275927	12.00650300078664
N	4.84780406397732	11.87587979474778	9.17538819436650
N	1.69468403365146	12.53011962909466	12.00269236989853
N	4.61707408569819	11.60406900791141	7.90839424059955
N	2.46917355087488	9.78952206877540	12.96518025054079
N	4.85715331101997	13.07473729593430	14.83227420194467
N	2.56906025737701	15.20207771803562	10.93662203713065
N	4.10682360370441	16.51641829894738	10.55987625578707
N	3.93318421600640	8.35333840657444	13.13920871312251
C	2.71796621624274	11.64259426171754	9.05194524534828
N	3.35510702057326	13.58191590101785	16.14463951527557
C	7.87721320555576	12.27656492225925	11.30689457151696
N	2.65739193079432	8.52946820310731	13.29490326196204
N	4.63487887308164	13.37491023225086	16.09392137114534
C	8.89769105948189	12.11557786878879	10.36863919303731
H	9.93335817175480	12.15145703238092	10.69802469651834
C	6.50633274422575	12.24633038381585	10.93670805913708
C	7.22564270443520	11.85968035807297	8.66558103798672
H	6.94607656210295	11.67907076696291	7.63152212168371
C	8.58491683488502	11.91471141641685	9.02520693111954
N	2.81814683722116	16.40466766774491	10.46369869265074
C	6.19750319614717	12.01038386857510	9.58876967231330
C	-0.51426956535134	11.93242144798633	12.34467170229672
C	3.73201357545546	11.93932846204056	9.97159259330780
C	6.50444667074192	12.60992427799923	13.08803495838420
C	-0.49221242070028	13.23914698943178	11.74484184913674
C	3.67237203972108	14.49370773554799	11.34242208547785
C	0.88445876206298	13.54691061858446	11.57675021762118
C	7.87813753948116	12.49708509268529	12.72824138379066
C	4.69900091844231	15.41345624581292	11.09250061221546
C	9.64544860300741	11.73931781670195	7.93741689575981
C	-1.54084882758309	11.10070439692595	12.78077215196448
H	-2.57392889741448	11.43429020013074	12.68976879049200
C	0.85347576095074	11.55095820217246	12.45419331623494
C	3.60169925348486	10.47006411763925	12.58936822334582
C	3.74291004862234	13.06565377522078	14.02878917740003
C	0.09741431624190	9.45901082439815	13.39887102844606
H	0.36266944234007	8.48145960818648	13.78609929544371
C	1.23678385699493	11.49169868530622	9.16223242195241
H	0.78428551902073	12.48396808097962	9.29395922887276
H	0.99819108017280	10.93234627132087	10.07164318908929
C	2.74316318349780	13.43076277518292	14.93970085739336
C	1.13822050361196	10.27668588130478	12.95942530286225
C	4.57859743854073	9.47315783878824	12.71641020842944
C	2.70098320084557	11.12447650507168	6.57904897498487
H	2.90101110119933	10.06206993593820	6.38696287139321
H	3.19850146022569	11.70709323847752	5.79735628446160
C	6.19880573701196	12.85785609187646	14.43153112526289
C	1.20906298885769	11.39214637211410	6.65264075011997
H	0.73400881463417	10.95657344661354	5.76605348374220
H	1.02109458291577	12.47493274261807	6.61558869886519
C	-1.24789364618171	9.84980270132007	13.33310235572056
C	8.89114935049315	12.58607697441222	13.67862892566792
H	9.92930668879394	12.48455799699670	13.36434701014600
C	0.63491717558189	10.80867295163380	7.93714571371533
H	0.83748016702937	9.72751336727715	7.96937935232492
H	-0.45472701173055	10.92156585409407	7.95969877376864
C	1.21371252136607	14.79773604617972	11.03358331687804
C	4.75013874875644	17.76015347489797	10.15518995936803
H	4.54597541400321	18.50892100097569	10.93192005303471
H	4.26700766420974	18.09394632788606	9.23151322170076
C	6.17858991132802	15.38976189936035	11.28984281166131
H	6.62956371019822	14.73533527973898	10.53149752623519
H	6.40896853549549	14.93045536825661	12.25551674606146
C	6.04319141574639	9.42222890837030	12.42910257724712
H	6.53141965519250	10.29757349585485	12.86231774510925
H	6.18757004388519	9.50434220552498	11.34242222617592
C	-1.49798602942279	14.12128273726684	11.34845577143408
H	-2.53855179858286	13.83986508257058	11.49101922017283
C	8.57768449962808	12.79613126519967	15.02475742591863
C	6.79384993969317	16.78319305286449	11.19428505936867
H	6.58512333087230	17.35370179194889	12.11184032590402
H	7.88365646360122	16.69235574912922	11.12632419013579
C	7.22604994719079	12.93868400061781	15.37079638067795
H	6.94347412958744	13.12899735589519	16.40018534306248
C	6.24231706438685	17.53817733148120	9.99195722201083
H	6.43726163010167	16.96929459337943	9.07139729445832
H	6.72777228791119	18.51354957907601	9.87219618477095
C	4.52062447572929	7.05172543197071	13.42696543948092
H	3.79046163389417	6.29294196398564	13.12949423401269
H	4.66327473717431	6.97687840595898	14.51335241964460
C	9.69480420656527	12.86107963140355	16.06858720446550
C	2.74280520270970	13.97850163257905	17.40621187994977
H	3.20376121028961	13.37921608987134	18.19763700440360
C	5.84369832842600	6.92114627535774	12.69618343656550
H	6.34204080775068	6.00576273435397	13.03559347405098
H	5.66329919439468	6.80604868435187	11.61751941195584
C	6.70310777858116	8.15066143274644	12.95777829146557
H	7.69061644939700	8.03853043663443	12.49635282729291
H	6.87603823694451	8.24661449847388	14.04011534906521
C	-1.16476126520534	15.34607329239985	10.77231641916008
C	0.19969630024947	15.66139990629314	10.63524466225165
H	0.49378255782535	16.61951051650041	10.21607199554679
C	-2.38074914611385	8.95334916984349	13.83791440063042
C	0.70368121175274	14.39066194738428	16.03367597243470
C	1.27328679354361	13.65949649295018	14.82109131994041
H	0.76845475784499	12.69121140165141	14.70184097736127
H	1.06815794067473	14.21838265317548	13.90368445760613
C	10.44231481738431	11.51822783884156	16.08299851691125
H	10.89369295610462	11.29328558434573	15.10944127508599
H	9.76198268089491	10.69523794913149	16.33518529989317
H	11.24817905436477	11.53439557796307	16.82795822935601
C	10.67071178411700	13.98930129696552	15.70297088796605
H	10.15954095277307	14.95985222068524	15.68745907305443
H	11.12555039849948	13.83452952416829	14.71754493903686
H	11.48476075674064	14.04588559458884	16.43700805413372
C	-2.21011642896598	16.35614535310176	10.29742472792863
C	-2.03381420873960	17.67485621802162	11.06579051940845
H	-2.16172562503756	17.51969614119226	12.14422774372653
H	-2.77829467650873	18.41167932921962	10.73805730090266
H	-1.04174294082899	18.11344698774263	10.90568314260008
C	-3.63576734070834	15.85229244369419	10.52916018756294
H	-3.83452710743252	14.92052588769224	9.98483104899571
H	-4.35552505684368	16.59961637971924	10.17447731464971
H	-3.83912996656273	15.68070590931706	11.59356939880288
C	9.15626655019610	13.12868933098103	17.47558506847381
H	8.47566806769307	12.33613335046111	17.81100456792513
H	8.62622375341863	14.08756952913141	17.53485351622710
H	9.98881304190419	13.16905261637823	18.18809688541606
H	3.00101396238768	15.03050378213607	17.58569117917150
H	0.96495534499973	15.45872120758417	15.98868777198345
H	-0.39040337322816	14.33685916980243	16.00627450087523
C	-1.86768911120441	7.60299007715945	14.34211928010443
H	-1.35825368488335	7.03898935687164	13.55062294733426
H	-2.71057338413411	6.99376681895315	14.68977078422185
H	-1.17563540888682	7.71611759002688	15.18598663202076
C	-3.37672578548584	8.69449574057579	12.69742254074080
H	-3.81979636295770	9.62364502963020	12.32063161790328
H	-4.19782897975457	8.05481268196884	13.04530250945131
H	-2.88669848887878	8.19050585722035	11.85513477034892
C	-3.10003443139393	9.66317118744911	14.99579176582375
H	-2.41015014775106	9.84978528513422	15.82819926835697
H	-3.92720545627890	9.04679961292583	15.37083122904049
H	-3.51776211238564	10.62778457567948	14.68378614597566
C	-2.02204916653816	16.60823502544347	8.79368308954533
H	-1.02559285388182	17.00663842709744	8.56841254923130
H	-2.76022200169023	17.33470744277508	8.43057993277458
H	-2.14945678443756	15.68016644991251	8.22249769462803
C	1.23859710011962	13.79374374371543	17.32882073090419
H	0.99010949669026	12.72377580890630	17.37931596599311
H	0.78488421803796	14.26745527243120	18.20699794874421
C	9.47370575555618	10.36295440997586	7.27624234205386
H	9.59085451549281	9.55772641037312	8.01208959390388
H	10.22615468787132	10.21754624205761	6.49051093160647
H	8.48607958586236	10.25287105877629	6.81291902134174
C	9.47282474641287	12.84170575733960	6.88111426567712
H	8.48653622698876	12.79786285301874	6.40400838547368
H	10.22837926411614	12.73937958145285	6.09169734033049
H	9.58396210358467	13.83581675632643	7.33176514420511

C	11.06332693640136	11.82890489766253	8.50390163102390
H	11.25632634027632	12.80342347240965	8.96980439398264
H	11.79416143989144	11.70041541188680	7.69658170527878
H	11.25594413694805	11.04598301219861	9.24805615219907

155

[Cr(L^{MIC})$_2$]$^+$_PBE0.xyz

Cr	3.68713299155759	12.48950766703299	12.00058586283287
N	3.33818284686227	11.42358242841120	7.84252001638252
N	5.68993370631717	12.45340205454244	12.00425543972216
N	4.85340882629391	11.82578217014013	9.17667028269877
N	1.68281691699469	12.52765038956898	11.99299946310612
N	4.62859190330525	11.54094300270710	7.91229732720321
N	2.45330694426313	9.76908159570860	12.92867302227938
N	4.86682640385037	13.10944000089045	14.82950993286921
N	2.56707596883648	15.22288743495056	10.97081196551562
N	4.10298661859798	16.53666322528285	10.58125425033702
N	3.91612232889713	8.33421205361592	13.12665421831074
C	2.72118147659055	11.61942450364937	9.03900607162554
N	3.36586115350321	13.58840150213531	16.15435305114899
C	7.88513840704917	12.26431601937424	11.30571908615585
N	2.63600965926162	8.50648370225230	13.24913397912252
N	4.64992851351112	13.41381022941991	16.09039942210298
C	8.90501098755733	12.08514291720306	10.37014065422332
H	9.94086988634145	12.13030847600506	10.69791623656554
C	6.51269725418809	12.22378164475355	10.93844676612650
C	7.23216025343589	11.79180150360074	8.67466499903027
H	6.95291961722714	11.59182392819782	7.64411275533281
C	8.59174506121875	11.85489064134362	9.03175842657060
N	2.81227814139589	16.42897347112559	10.50689287940682
C	6.20359036120036	11.96090223384012	9.59436035972191
C	-0.52663236597785	11.92868861195633	12.31994917323554
C	3.73149517788290	11.91165403725813	9.96182758352521
C	6.51370786984603	12.63449604056447	13.08056312119286
C	-0.50048020599530	13.24862564012880	11.75105404631411
C	3.67580010066329	14.50909945480201	11.35118258727403
C	0.87813747412259	13.55597039514616	11.58981550425840
C	7.88788536755361	12.51743702222472	12.72099411585188
C	4.70057532072988	15.42571674065663	11.09111024067663
C	9.65164337278112	11.66114702301986	7.94637259780836
C	-1.55652690620119	11.09019558019389	12.73556933462455
H	-2.58847499450802	11.42870405271653	12.64953221164309
C	0.84069589680225	11.54176110915014	12.42333404262503
C	3.59382725904493	10.45426417464821	12.59147685847234
C	3.74471914257972	13.06745689092398	14.03972511097810
C	0.07640924279635	9.43386547624422	13.32659338056715
H	0.33805040615679	8.44975867783695	13.69933783500671
C	1.23609429521916	11.50136477028687	9.13830473290519
H	0.80631465593690	12.50492734166356	9.26190753696452
H	0.97722933279351	10.95269736081328	10.04849973770565
C	2.74543148877000	13.41059582396371	14.95686565069771
C	1.12088106540946	10.25760690325812	12.90862965227911
C	4.56959143124311	9.46133533661816	12.73524910938232
C	2.71579376227847	11.07321163364465	6.57222597337724
H	2.89090728642495	10.00336613164329	6.39776398882613
H	3.23505960693857	11.63174135019708	5.78714349450076
C	6.21061940654332	12.90678384512277	14.42103238439467
C	1.23042908716958	11.37930718536196	6.62788818433480
H	0.75297478390465	10.94638383761896	5.74129510684343
H	1.07106259578377	12.46611730539022	6.57727567884499
C	-1.26843550536640	9.82749728385099	13.26184488999618
C	8.90301364860176	12.63234505471009	13.66662507919500
H	9.94070018619952	12.52646808227953	13.35206348840628
C	0.62875948466610	10.82420916082225	7.91247066791201
H	0.80223781718278	9.73842816262236	7.95665244948044
H	-0.45771200352449	10.96564400662231	7.92305077767946
C	1.21016791041594	14.81867826481180	11.07358044680177
C	4.74393919002633	17.78178388562076	10.17738114557852
H	4.56549405762477	18.52104935926363	10.96943431234379
H	4.23965273161747	18.13145611059561	9.27108905738551
C	6.18436906909065	15.39001981936896	11.25254199312194
H	6.61072024067683	14.73650846716326	10.47909312174042
H	6.43486639587772	14.92272114133307	12.20911113967994
C	6.04271546616378	9.42071908599446	12.49311013491271
H	6.51133789269389	10.29958408795499	12.94137186197187
H	6.22039038213563	9.50488962009037	11.41150752563266
C	-1.50407905422175	14.14349076222218	11.37828555288347
H	-2.54529534948817	13.86164828217473	11.51542517289394
C	8.59245361417875	12.87306946475183	15.00778198436441
C	6.80733312418803	16.77980822482884	11.15248716172386
H	6.62674647692185	17.34249565574029	12.08072990334586
H	7.89425934961696	16.68188121376967	11.05501476238127
C	7.24051394571291	13.01472728321637	15.35432255051419
H	6.960304237921 79	13.22308162819386	16.38083878947326
C	6.22993461752389	17.55153114252279	9.97303038889309
H	6.39656556953591	16.99180027966661	9.04139729423511
H	6.71902046088455	18.52485747184433	9.85148752798062
C	4.49964449347777	7.03150410069398	13.41725855244894
H	3.78575198708781	6.27285050498322	13.08225120512582
H	4.60216500373619	6.94183440738425	14.50701123394367
C	9.71205933253717	12.96731844197525	16.04680554813430
C	2.75625092241798	13.97924352807337	17.41896178430017
H	3.24439268017570	13.40266068937830	18.21094425054416
C	5.84974364053357	6.91786831274032	12.73424025364423
H	6.34157677401549	6.00231337796967	13.08255363103770
H	5.70982438675027	6.81238092001456	11.64858555603968
C	6.69133833782751	8.15029290441776	13.03797663629295
H	7.69587506259683	8.04743924971740	12.61255750324397
H	6.82361458616836	8.23881526022519	14.12669334456513
C	-1.16813940250588	15.38135330205166	10.83310243187603
C	0.19718151894126	15.69448629391402	10.69966250600624
H	0.49264905535616	16.66072127476736	10.30068479600128
C	-2.40465538412040	8.92281483811946	13.74442971438143
C	0.68891625216942	14.31455267342695	16.06650509458913
C	1.26761744535119	13.59222200609694	14.85282148278571
H	0.79340261503255	12.60697202097509	14.74596015070441
H	1.03426346499434	14.13760030277926	13.93417262958358
C	10.46376162897750	11.62746712521736	16.09292733788162
H	10.91298137502197	11.37956507126705	15.12394984779289
H	9.78677492056027	10.80892124038008	16.36756582512345
H	11.27171300495645	11.66487876251011	16.83486318464059
C	10.68423963290758	14.08840466830749	15.65062325811378
H	10.17079791797437	15.05712795727858	15.61251152873876
H	11.13625348379072	13.91019062633887	14.66787737934437
H	11.50040655796213	14.16500283890504	16.38049203223280
C	-2.21113349332972	16.40555902481384	10.38390962919103
C	-2.02192735249068	17.71054001787972	11.17230499644196
H	-2.14285460436561	17.53857179743381	12.24900697963959
H	-2.76468989359035	18.45651548427810	10.86187190431859
H	-1.02880302908964	18.14663274354106	11.01200645643246
C	-3.63778359111458	15.90589903697125	10.61849470378995
H	-3.84718332894039	14.98612671774180	10.05803550011496
H	-4.35614661092972	16.66414159486291	10.28473758158471
H	-3.83244754126138	15.71469775468817	11.68120981065927
C	9.17652673873707	13.26844924788668	17.44812461713363
H	8.49924768741037	12.48252889781741	17.80516727165859
H	8.64380251343457	14.22698741511370	17.48519136693879
H	10.01089604824612	13.32869155155907	18.15709414394564
H	2.98341314385915	15.04058846306939	17.58503552697275
H	0.91514867516192	15.38996720751016	16.00913944975561
H	-0.40310433631804	14.22552649627605	16.05254136545459
C	-1.89529994066734	7.56223476304092	14.22420737021402
H	-1.38425285018741	7.01265618164915	13.42362405595106
H	-2.74001532221406	6.94770004884691	14.55776191475296
H	-1.20557222833200	7.65848471145248	15.07205620556549
C	-3.39656308946830	8.68815186017818	12.59531461954874
H	-3.83672713517848	9.62553284107553	12.23588646507374
H	-4.21990280016514	8.04269263303162	12.92691995743276
H	-2.90390902760413	8.20079297416739	11.74481685492284
C	-3.12746015962054	9.61072887182634	14.91317287848247
H	-2.44061173090172	9.77980261311776	15.75183237411848
H	-3.95729151728399	8.98876749996116	15.27289561962554
H	-3.54217592737969	10.58208700599566	14.61859933000509
C	-2.03285010013160	16.68152593223596	8.88310164596732
H	-1.03544903199756	17.07739584296676	8.65734782639397
H	-2.76911553406333	17.41841491790160	8.53747156199646
H	-2.17000527839531	15.76382037259162	8.29761959358610
C	1.25766016791381	13.74687407599325	17.36050981364138
H	1.04368006290672	12.67014511114157	17.42376485420531
H	0.79924921537562	14.21425779179073	18.23963781923554
C	9.47661420323392	10.27694397620908	7.30280728468409
H	9.59050059932196	9.48082682976630	8.04900857028784
H	10.22962361052790	10.11937931755997	6.51997040822802
H	8.48938723115655	10.16367416039518	6.83940463824300
C	9.48199677074970	12.75040260848424	6.87599644694670
H	8.49538753776597	12.70291684352924	6.39979951906918
H	10.23713768076919	12.63653577208553	6.08770524099119
H	9.59545879438734	13.74976661283580	7.31428323676607
C	11.06971042869995	11.75413408824102	8.51184306716205
H	11.26656927693246	12.73511621870958	8.96227243078716
H	11.80032023405079	11.61001437564791	7.70694877208995
H	11.25875839787797	10.98237844537381	9.26850799054293

155

[Cr(L^{MIC})$_2$]$^+$_PBE.xyz

Cr	3.68582302676768	12.47479919812851	12.00811568063571
N	3.30140756946004	11.43849410519622	7.82665665534188
N	5.68850077696204	12.43074714580619	12.00725702396761
N	4.84363305761294	11.85438618378455	9.16193388443520
N	1.68161792992311	12.52245037493308	11.99897488498801
N	4.60933199417989	11.57765404410518	7.87488886416801
N	2.44121866818124	9.75569104858571	12.94650248367036
N	4.86933418722080	13.04761723681254	14.85210741614259
N	2.58092735608085	15.21426592429313	10.95908246259175
N	4.14651248439750	16.54236884744865	10.61515406053757
N	3.91851874710854	8.29961622310970	13.12495389431381

C	2.69288513620252	11.61000877666202	9.04581056029443
N	3.34849685279994	13.54368843245108	16.18467183094905
C	7.90208300828985	12.26183204301114	11.29593311924326
N	2.62198256939866	8.47130435862684	13.27156286768127
N	4.64854977181248	13.34418614649945	16.13696403583090
C	8.92380503021251	12.10377563420210	10.34679812795195
H	9.96832585221334	12.14258511192809	10.67348638634193
C	6.51715726922027	12.22478443259436	10.92669813661146
C	7.23471381125933	11.84334742114711	8.63566949782514
H	6.94836900040256	11.66342964389530	7.59534721363111
C	8.60325072398158	11.90142281371871	8.99324794747140
N	2.84056740759047	16.43887603886030	10.48960903075653
C	6.20040510142724	11.99028777865684	9.57071311601554
C	-0.55019633330744	11.93252606734092	12.32896058984024
C	3.71642890391662	11.91551683026931	9.96888920403309
C	6.52300210895724	12.59354394040634	13.09224053654898
C	-0.51801973044594	13.24752412805997	11.73337541328400
C	3.68973786026658	14.49515696698293	11.38257709740879
C	0.87386208925488	13.55398592325844	11.57447877458435
C	7.90797692831801	12.48901184803373	12.72180470405416
C	4.72927849243323	15.42341661743926	11.15848108182882
C	9.66848091152580	11.72825974317636	7.89697054951432
C	-1.58764231456798	11.09788014688536	12.76178279260323
H	-2.62632114453057	11.43963963647312	12.67118724649442
C	0.82671049577382	11.53765889985686	12.44197138944241
C	3.59233351302565	10.44218466024770	12.58559151277082
C	3.73962465197164	13.03258588429799	14.04566742574375
C	0.05178240025359	9.42942587972929	13.37762489018826
H	0.31338748766378	8.44156527628330	13.76233414139447
C	1.20667447115650	11.45096736603709	9.16164634231733
H	0.74720397798932	12.44765743827778	9.30493976982781
H	0.97206362651826	10.88429224429386	10.07728145387041
C	2.73217622549619	13.39109135879032	14.96705349361927
C	1.10664781039366	10.24929007963994	12.94058418987596
C	4.57382500427650	9.43586035537769	12.71825440634324
C	2.66460260519064	11.08423470452439	6.55337125260267
H	2.86821141226546	10.01359530160332	6.36186388362774
H	3.16200299272378	11.66811269353604	5.76088850707652
C	6.21773978796754	12.84739516419015	14.44481341758379
C	1.16272117773394	11.34915853128003	6.63325936329120
H	0.68167131735869	10.90457267249838	5.74455594960600
H	0.96972913651591	12.43893275220683	6.59087645294880
C	-1.29932949188990	9.83300672509810	13.31354418158749
C	8.93010143840014	12.60555579658825	13.67183680567751
H	9.97583409868217	12.51855835085571	13.35108657452466
C	0.59252802859030	10.76606873452672	7.93095444406752
H	0.79450536224003	9.67674487070932	7.96269406213180
H	-0.50458226229641	10.88137024258510	7.96053708196324
C	1.21735731777725	14.81348686529293	11.03605210931537
C	4.80715962405391	17.79219068533241	10.22293729451731
H	4.59235155685780	18.54615028010156	11.00386794602911
H	4.33641988113835	18.13520745971793	9.28638094258376
C	6.21054775351517	15.39752344683572	11.38785062006863
H	6.67889884943641	14.73016700877525	10.63923612927447
H	6.42286786494438	14.93786983234756	12.36671097874128
C	6.04935248286724	9.38792775619140	12.45450239556888
H	6.53285333758860	10.26632016703363	12.90785712191898
H	6.21476517583772	9.48702848219616	11.36382014313928
C	-1.52102401618272	14.14160054429028	11.32790365959246
H	-2.57175002807954	13.86373984741489	11.46202829376482
C	8.61803396502679	12.83334577412263	15.02720030604815
C	6.84192581017907	16.79539873389926	11.29863235846554
H	6.62459956403080	17.37187622870569	12.22004645247581
H	7.93964964720805	16.69489100226685	11.24534123065850
C	7.25800912003120	12.95492769695161	15.38389057830396
H	6.97573183833073	13.15386592312904	16.41966175801818
C	6.31016169144905	17.56176836552586	10.08268482493454
H	6.51865845883400	16.99145038413547	9.15643471484784
H	6.80553916359586	18.54231046147400	9.97360440453318
C	4.50590195116959	6.98563960614403	13.40460135811314
H	3.77646899790210	6.22516474127153	13.07995970003851
H	4.62800683184548	6.89170014636301	14.50059871096088
C	9.74768325062609	12.95809136405995	16.06465095270926
C	2.73042128221322	13.94042774132893	17.45451286512816
H	3.19763039912037	13.33921393488519	18.25240992689217
C	5.85148367347428	6.86594920108908	12.69312369119142
H	6.34949161833328	5.94219024725265	13.03589269281532
H	5.68969561576839	6.75859499462355	11.60266634219247
C	6.70841644099782	8.10307442829952	12.98176186924100
H	7.71039024712275	7.99733371131062	12.53141136037179
H	6.86627299299840	8.18815860993470	14.07540449843699
C	-1.17371011814730	15.37507930730697	10.74993753350218
C	0.20133739447530	15.68817264788277	10.62566272118636
H	0.50616890036161	16.65232058582369	10.20832403342430
C	-2.44257937641401	8.94104481809574	13.82905369257139
C	0.67635868898060	14.34789213274311	16.07241187842694
C	1.25546665121087	13.61867066737024	14.85026048183249
H	0.74763586888245	12.64374232216385	14.72191633546103
H	1.04789763636989	14.18396540324661	13.92738600934712
C	10.57400815441847	11.65024502417228	16.07636120898514
H	11.02641262527262	11.44091154624773	15.09108010375497
H	9.94154556790117	10.78593312799325	16.34796487187666
H	11.39498322317665	11.71882662840775	16.81317653255402
C	10.66238236826237	14.14329055974791	15.67585691041922
H	10.09355814270252	15.09019807852841	15.65321750120322
H	11.11895940584187	14.00061602872046	14.68063644549493
H	11.48416601389987	14.25481875437879	16.40671031533596
C	-2.21736706161876	16.39201495237096	10.25661216587965
C	-2.03215214620691	17.72942298325828	11.01142176422485
H	-2.16533662659671	17.59073758386468	12.09937856378554
H	-2.77490564683993	18.47242594652782	10.66793481444744
H	-1.02917229288854	18.16082484725296	10.84683084467668
C	-3.65681741530250	15.89926145491646	10.49061932629151
H	-3.86394842925609	14.95650846742429	9.95267026564305
H	-4.37508661824631	16.65325037994597	10.12319503355371
H	-3.86650196560129	15.73862949839675	11.56354815303063
C	9.20985594825955	13.20508605124887	17.48590042468064
H	8.56398561963727	12.37782130991716	17.83160581118750
H	8.63376455497148	14.14564678639120	17.55210968389681
H	10.05294185632187	13.28417708738767	18.19467585520468
H	2.98817035083494	15.00102058568834	17.63649543161497
H	0.93210987608759	15.42545896108297	16.02840392571435
H	-0.42515982300060	14.28675244858836	16.04375969156460
C	-1.93856633404227	7.56942467632080	14.31376764861585
H	-1.44564615592500	7.00266743435643	13.50326545557053
H	-2.78993186846300	6.96379511271782	14.67140982070019
H	-1.22635511994818	7.66365319924808	15.15325272503345
C	-3.47058900888274	8.70872483416996	12.69745703774759
H	-3.90861253499479	9.65527346573593	12.33572320450549
H	-4.30155030764566	8.07523280659861	13.05752700189293
H	-3.00254169535472	8.20127570830841	11.83482764884826
C	-3.13414185349538	9.65509699344492	15.01518054591536
H	-2.42113399157258	9.82416287384196	15.84222569125885
H	-3.96932926163442	9.04318016635475	15.40232719792923
H	-3.54526514958499	10.63611952009240	14.71822772559420
C	-2.01893627584292	16.62398848550150	8.74004178636215
H	-1.01122171883662	17.01515636383154	8.51389657063054
H	-2.75529125085175	17.35556430808973	8.36010315790177
H	-2.15011332689364	15.68272039113174	8.17629107607829
C	1.21693767200415	13.75129231435529	17.37667366502328
H	0.96907377241282	12.67317420283955	17.43062337710146
H	0.75919613790549	14.22924635291754	18.26034782823816
C	9.49617585195907	10.34251762204099	7.23134083035057
H	9.61810110798326	9.53110993655268	7.97127866492224
H	10.25147841170079	10.19779848170418	6.43734753785260
H	8.50003370330216	10.23015490606128	6.76827666109860
C	9.48976037093448	12.83711180370031	6.83309838016539
H	8.49376512537290	12.79346654524637	6.35808961005604
H	10.24623193622899	12.73201805591810	6.03411233629562
H	9.60540936356758	13.83906677669634	7.28431259383630
C	11.09697536748366	11.82177828398381	8.46257600028529
H	11.29098846985458	12.80316316397771	8.93188899139735
H	11.83119356447776	11.69489898325665	7.64756644462975
H	11.29597694299141	11.03411378579054	9.21147935526057

155

[Cr(L^MIC)₂]²⁺_q_PBE0.xyz

Cr	3.68893442727023	12.49836977367914	12.01165815531672
N	3.30711849209558	11.49728018380855	7.84112296838856
N	5.69411862641963	12.46658033580517	11.99930816166815
N	4.81663305354278	11.96106368792594	9.16179986499652
N	1.68161363478246	12.53813462461504	12.01643363719919
N	4.58552624849211	11.69442887942020	7.89442558392594
N	2.47828369070601	9.83705034542064	13.07872066088176
N	4.86991173365085	12.99575074113222	14.84636907157685
N	2.58479214050131	15.17772273611323	10.88278695447202
N	4.10394230735378	16.53179237026258	10.57431701149324
N	3.91937208883135	8.37779446632146	13.25648353304917
C	2.69811467591606	11.62285757692935	9.05231925745247
N	3.39940996859732	13.55908233769340	16.17236094050800
C	7.87711371730669	12.32437016504294	11.27274849385081
N	2.65904189703020	8.58970261177469	13.45843562208696
N	4.66467716445903	13.29196879185103	16.11199367932573
C	8.88163993789912	12.21405664863777	10.32154491875618
H	9.92329825089732	12.23248254490941	10.63153911148592
C	6.50222626649817	12.31105568376699	10.91191222384371
C	7.18474557954997	12.02950806523217	8.61874885996218
H	6.89916530769133	11.88730293444391	7.58076628459829
C	8.54676965832764	12.08105858452896	8.96566561804989
N	2.82988702498753	16.37829389551726	10.40395339651792
C	6.16625172864284	12.12505144722087	9.55999142181470
C	-0.51881113905377	11.94937932359633	12.37408281958036
C	3.70235585045782	11.95463706050407	9.96677788499729
C	6.51698717494966	12.56469948367307	13.08572211113679
C	-0.49449098139555	13.22987080350947	11.70162080362040
C	3.68590523452405	14.51195999649132	11.36649812808912
C	0.88362226174033	13.53361979212663	11.53315736058492

C 7.88748371737378 12.46593161713116 12.71254312950543
C 4.69615436007220 15.45651139050790 11.16377066413865
C 9.59531901200605 11.96515684899333 7.86257150795932
C -1.53780691258058 11.13662620577885 12.83623316980169
H -2.57492571074351 11.44607375062737 12.71838927093583
C 0.84871166828652 11.57782273688210 12.51325984928369
C 3.61047304319944 10.47343655405861 12.62582895274828
C 3.75008861769444 13.05205665700392 14.05042927239262
C 0.11052608616812 9.52825516062557 13.55073614891499
H 0.37292320625698 8.56883528469885 13.98245290436985
C 1.22773799398524 11.39134348021776 9.17201681154459
H 0.72041841557408 12.35954868292632 9.28144955973078
H 1.02495717340479 10.83345185927014 10.09125581291580
C 2.77408068992113 13.45007045652781 14.96777474259495
C 1.15066246113157 10.32884336601668 13.07319225769785
C 4.56924645303207 9.46313408317101 12.75194376932233
C 2.68722166179474 11.12533622935483 6.57090916723295
H 2.96543240391114 10.08389375373217 6.36417686679317
H 3.13144632410432 11.75370656871968 5.79271692713962
C 6.20307782853380 12.75208915779176 14.43866981200684
C 1.18098541912780 11.28157155231850 6.66613599890686
H 0.72965473233565 10.80982347554615 5.78615786642636
H 0.91061766898392 12.34659539405437 6.62762663201854
C -1.23353842815569 9.90843981928316 13.45681319043637
C 8.89660418170545 12.49357654192808 13.65877459642988
H 9.93581653905539 12.40765278003085 13.34591912823031
C 0.66497726878949 10.65826094512806 7.95667445099646
H 0.94618906277564 9.59529193154257 7.99110196186978
H -0.42953300708688 10.69074473372205 7.98889940190326
C 1.23302538475379 14.75918577673579 10.94079580720267
C 4.73743347792217 17.79012075686422 10.18513760459977
H 4.44584726749911 18.54659203413999 10.92502385427400
H 4.31626745543757 18.08378023368523 9.21861751604035
C 6.15737071194360 15.48634082400785 11.47060787481422
H 6.68689915305029 14.84235408508889 10.75528789044919
H 6.32715221600448 15.04804819484995 12.45904898067297
C 6.01387416267587 9.35981079054826 12.38451387429187
H 6.54644584398018 10.24709609520990 12.73120393668268
H 6.08982133496414 9.36439657470641 11.28730727464297
C -1.48920005058779 14.08232775446775 11.24493890537731
H -2.53403925533856 13.81715605807443 11.38414800919098
C 8.57905327386161 12.62354590172340 15.02523726864275
C 6.72620644362545 16.90175039957678 11.40084264465211
H 6.42419613433260 17.47626803528135 12.28895715203399
H 7.82034804410017 16.85340707518777 11.42052145019078
C 7.23269607203342 12.76404598395182 15.38197853848959
H 6.95864147313888 12.90420952207866 16.42166506071720
C 6.24389796432189 17.61523735443881 10.14424916812302
H 6.53152698820291 17.04194082650108 9.25121825536044
H 6.70362116990439 18.60490854784863 10.04519069481381
C 4.49598447433138 7.07450042722158 13.57906813291142
H 3.73878847307336 6.31909462668466 13.34898124862632
H 4.68176840469115 7.05178888747648 14.66091975953440
C 9.69760770184859 12.61130597912865 16.06334629653140
C 2.81028781971672 13.98133592078309 17.44130218003821
H 3.22893557420093 13.34328555487553 18.22574987418325
C 5.78300695400434 6.88310258006843 12.79956196143696
H 6.28038583279577 5.97885374133784 13.16778810614690
H 5.55482700712829 6.70996190462813 11.73814243483754
C 6.67524535656039 8.10704612382446 12.95563333468308
H 7.63836587360605 7.94796180534792 12.45848063115367
H 6.89869355313255 8.26104699414746 14.02162786161579
C -1.14083878864752 15.28017966174536 10.60247889567110
C 0.22398400069879 15.59575306197279 10.47642366860524
H 0.51871445176152 16.53672117465104 10.02130449920130
C -2.36566327653212 9.02401829517964 13.97057950363475
C 0.80272356128963 14.53280220638018 16.06501233425961
C 1.31811494247210 13.75981948593939 14.85311521348268
H 0.75831081712024 12.82009814372053 14.75098161578808
H 1.14342296363258 14.32213953400241 13.93068871228864
C 10.43421726254419 11.26404627535019 15.97282414698350
H 10.88314487376886 11.10470742437178 14.98532260884346
H 9.75306885409017 10.42809454718417 16.17462286233772
H 11.24379092800904 11.22590177514076 16.71185896494301
C 10.67657667456437 13.75817646869071 15.76357670657829
H 10.17321465745841 14.73131588318507 15.81484491266595
H 11.13437410736994 13.66519146105468 14.77181473502296
H 11.48963496025546 13.75978744192085 16.49960687483827
C -2.18024074647072 16.25879322001855 10.06277344779050
C -2.03303472860723 17.60188168416234 10.79692519226970
H -2.19135026677796 17.48150627375019 11.87555694480278
H -2.77667911148018 18.31723521491100 10.42520895643628
H -1.04303083699542 18.04880028893089 10.64784378259218
C -3.60792055305457 15.74804739320681 10.26607055442182
H -3.78248686242036 14.79479109444475 9.75135108871206
H -4.31921565134112 16.47336090208480 9.85523673650586
H -3.85301206400028 15.62234380985077 11.32804971872519
C 9.16757081409091 12.78322085880280 17.48775816961761
H 8.48404486718615 11.97372281296795 17.77307484881171
H 8.64974465989699 13.74142315096493 17.62033553150261
H 10.00413607143816 12.76619182143900 18.19542661159525
H 3.14220943190133 15.01050141032543 17.62936068608697
H 1.13746945350902 15.57957004671317 16.01634123463115
H -0.29223814925089 14.55573743815257 16.04176266726879
C -1.84794814589224 7.74106934578653 14.62235765692674
H -1.29171414609663 7.11473028060223 13.91379949399196
H -2.69414982411814 7.14612425436621 14.98425526254181
H -1.20340015579497 7.94902210310861 15.48573293274952
C -3.27149377989797 8.64083766397846 12.78767395075393
H -3.71778448007562 9.51866901539705 12.30567375399952
H -4.09379714267019 8.00399878193011 13.13570284632671
H -2.71185297601523 8.08394504476930 12.02633028926571
C -3.18087434692871 9.80862142911403 15.01155127366623
H -2.55764100529145 10.09850616230721 15.86645480808655
H -4.00349943982860 9.18901770794152 15.38860589055312
H -3.62359761458328 10.71911137133610 14.59070208590545
C -1.94274354501758 16.46219152181941 8.55762856622307
H -0.94824624229077 16.87328089604636 8.34789130195499
H -2.68120138655425 17.16396075125854 8.15153178585122
H -2.04115284405892 15.51590802297193 8.01148374343758
C 1.29737608481496 13.89945123561104 17.35911598064107
H 0.97530016471068 12.84963066255489 17.41287756400796
H 0.87703148358768 14.40364167908777 18.23645506961075
C 9.42735774409594 10.61122759320302 7.15286759932584
H 9.56432417360972 9.77832003867559 7.85330704740843
H 10.17420041531279 10.50798010826437 6.35624784064207
H 8.43826158644770 10.50722371105343 6.69157172636556
C 9.38833598815565 13.10650029718210 6.85325625859565
H 8.39978651725601 13.06511779252273 6.38096348242379
H 10.13752547348044 13.04238523252030 6.05497798576649
H 9.49324751769066 14.08554758786514 7.33679375936131
C 11.01932849890986 12.05312303669638 8.41396683756044
H 11.20749744765000 13.01028795600003 8.91645127103203
H 11.73802058468703 11.97232802083610 7.59067502867760
H 11.23979183308050 11.23981315394080 9.11651553499097

155

[Cr(L^MIC)₂]_q_PBE0.xyz

Cr 3.68975256661759 12.49281375417544 12.02017872622235
N 3.28564175828804 11.51546985574849 7.86220767265030
N 5.69033110455633 12.46538434761050 12.00402452698369
N 4.82291370568771 11.98084463627594 9.16743487466074
N 1.68678765803874 12.53269008982939 12.02298916972494
N 4.57590170852123 11.74024189401724 7.88830082201343
N 2.45945829979518 9.83047403147703 13.08407266162677
N 4.89200354487999 12.99778031330634 14.85004410616057
N 2.57574723984454 15.15941208983079 10.86674540068579
N 4.12025871289224 16.50311763814640 10.56548978051337
N 3.91925055963404 8.37536499207420 13.24714180229014
C 2.70878610749079 11.59311675781165 9.09625177334994
N 3.40174005208794 13.57662262143494 16.16051460264583
C 7.87706873367379 12.32263158064266 11.27556501144442
N 2.64548075390139 8.57612730648243 13.46236837786417
N 4.67784467986616 13.29353917787605 16.12155394363657
C 8.88924015809999 12.21844947219411 10.30975783116500
H 9.92920785338057 12.23621331641037 10.62676719014686
C 6.50130711076720 12.31348911580276 10.91618497714371
C 7.18631080622735 12.06050670217761 8.61385818290031
H 6.89095351493604 11.93244305671125 7.57674369760355
C 8.55036871537425 12.10212153647244 8.96402433205922
N 2.83401640590586 16.35804403494101 10.36575744069011
C 6.16523865296836 12.14713263100546 9.56164602199418
C -0.52162593511350 11.95433443358800 12.37355819951089
C 3.71947052570103 11.93222702800894 9.99082462175159
C 6.52114593835922 12.55956653974135 13.08267294620137
C -0.49439658753122 13.22044762830747 11.70291983737508
C 3.66813763014592 14.49913415388259 11.38464402614314
C 0.88453203202589 13.52354748208339 11.53415717906571
C 7.89180268077958 12.45619371389099 12.70277952645698
C 4.68356449286049 15.43173772195769 11.19594718939763
C 9.59410830342045 11.99581508653738 7.84998416218070
C -1.55266264032022 11.13285020697446 12.83754532616311
H -2.58720014142099 11.45246375207155 12.71529952645823
C 0.84646971423915 11.57869443218924 12.51695859832049
C 3.58974184344423 10.47385725059786 12.63058711143370
C 3.77996147739310 13.06879499875759 14.04262890662165
C 0.09203121475370 9.52610521654016 13.55156138735402
H 0.35924188345359 8.56684733152936 13.97962908880634
C 1.25354066970732 11.30477155232492 9.25731650299980
H 0.70979674053459 12.24439637587982 9.42692123023919
H 1.10232622851521 10.70919643146944 10.16290976321389
C 2.79999288049818 13.48202420379651 14.94146681589781
C 1.13824317502436 10.32764107495738 13.08054899181549
C 4.55332812941765 9.47299835455702 12.74531424146359
C 2.64626816005943 11.17039016501802 6.60926290185870
H 2.93744953354326 10.14340278336529 6.34383819938307
H 3.04237029435003 11.83737944422976 5.83514680159554

C	6.21822859523448	12.74300642556817	14.44032356101784
C	1.13789757133251	11.28132856987940	6.75264550307346
H	0.66832444823028	10.83661517615027	5.86691972604248
H	0.84307155261170	12.34060407314928	6.77585591143756
C	-1.25358138927889	9.91268554891132	13.44961213386911
C	8.91264998839877	12.47982598589361	13.65792114976488
H	9.94890176598004	12.38596822932373	13.33446209704865
C	0.67915993725039	10.59578796775014	8.03417933437236
H	1.00048943706680	9.54281713458841	8.01873923143758
H	-0.41510835423026	10.58868348397185	8.09885255934976
C	1.23128492782800	14.74211486660639	10.92518907349431
C	4.76816789746386	17.73810719533617	10.17382680557412
H	4.46264606520190	18.52654530421620	10.87724767518772
H	4.39243765603998	18.01492715472398	9.18224058396206
C	6.13089243648996	15.45758608472995	11.56005027264850
H	6.69112477930578	14.79308064750485	10.88767300539237
H	6.25883180012447	15.03811903279882	12.56282250930865
C	5.99450039783536	9.38829052932416	12.36395287646991
H	6.51860816566313	10.28793019809401	12.69181790227781
H	6.06573507911569	9.38295767924835	11.26624395339634
C	-1.49800542769402	14.08135075571680	11.23612399783290
H	-2.54057289262379	13.80794904996006	11.37951497489981
C	8.60095410884063	12.61108336104622	15.01359831282505
C	6.70644668997454	16.86914897082112	11.48353829368961
H	6.36511188801460	17.46598223754172	12.34346839349577
H	7.79942143827672	16.81971829741651	11.55037822125297
C	7.25342558654720	12.75394917214332	15.38105882269906
H	6.97533766712112	12.89551838533521	16.41909438891891
C	6.27609542974078	17.55582835727712	10.19266250558631
H	6.58859976379875	16.94920855262136	9.33016209748456
H	6.74931976141003	18.53896926744653	10.08179533446941
C	4.50956287725770	7.09376036107133	13.57867923941092
H	3.76773815122274	6.32094255700408	13.35105981059596
H	4.70102845650134	7.06701684973000	14.66143762340351
C	9.72420742912985	12.59535717708038	16.05454642726796
C	2.79880918996832	13.99671515002991	17.41014333985427
H	3.18578100930640	13.34784751260044	18.20360031370900
C	5.80209908862134	6.91290146510830	12.80245225905263
H	6.31352116489272	6.01677329717735	13.17433086191530
H	5.57406569209841	6.73673118361797	11.74101555249293
C	6.67552990937637	8.15283140219602	12.94643086485334
H	7.64333136437927	8.00521981096661	12.45332395719268
H	6.89183343869597	8.32157155176411	14.01230266839357
C	-1.14846764431620	15.26479560957847	10.59043492031145
C	0.21823963956431	15.57927367417253	10.45352229452545
H	0.52139811047001	16.51302834719930	9.98912613570617
C	-2.39053723172777	9.02163799677046	13.95745020348705
C	0.82651697537470	14.59014219547294	15.99848113593824
C	1.35527607573684	13.82200972034183	14.79027888624586
H	0.77416733722484	12.89960851148140	14.65170681891908
H	1.21700128786225	14.39741613501788	13.86963950916681
C	10.45294788421319	11.24444577592539	15.98363352128496
H	10.88726028707846	11.07247083441662	14.99182072960403
H	9.75933355048200	10.41965073706850	16.19027008565380
H	11.26691125080198	11.20091507247395	16.72025973435958
C	10.71667944340694	13.72933976791493	15.75770263796128
H	10.21668339394933	14.70483897798732	15.80395218296946
H	11.16037271871968	13.63026523676889	14.76026269804399
H	11.53561643683712	13.72895695592625	16.48984590963815
C	-2.18538746907933	16.24933374753920	10.04546326245978
C	-2.03767162331935	17.59777911188466	10.76687030028500
H	-2.19867265981794	17.47917572767908	11.84565419589102
H	-2.77126307218028	18.32289971744706	10.38883749767600
H	-1.03832552789743	18.02558016368709	10.62526496711259
C	-3.61630721218577	15.75000607318652	10.25425036367048
H	-3.78987477958640	14.79146391505223	9.74921103534763
H	-4.32842512424139	16.47678178961813	9.84336149005933
H	-3.85012948951394	15.62317108363264	11.31861895316367
C	9.19857358840969	12.78208027379608	17.47941028107964
H	8.50480117134051	11.98153057732693	17.76460546803002
H	8.68006454009362	13.74169812022605	17.59753085768327
H	10.03505565302010	12.76604294295261	18.18956184364306
H	3.13279620850235	15.02203634828239	17.62485602995941
H	1.18312079033203	15.63135452773883	15.97084943058010
H	-0.26781096717531	14.63255434216771	15.95230720696631
C	-1.87518173242252	7.73501109556918	14.60520811948324
H	-1.30850264014752	7.12083043194746	13.89445164591499
H	-2.72118027249449	7.13380496161499	14.96185927239716
H	-1.22861973764374	7.94454654206420	15.46653066366546
C	-3.29637201713892	8.63482148378142	12.77783046418902
H	-3.73078665368348	9.51757616289461	12.29413245842284
H	-4.12311274821409	7.99572550069299	13.11679041613404
H	-2.72781666821810	8.08514675241208	12.01741520017177
C	-3.21369737816708	9.78949510850087	15.00237404242748
H	-2.58828714882469	10.07479441036616	15.85760426998483
H	-4.04161811522171	9.17073851828121	15.37450595095340
H	-3.64340566718398	10.70736362630543	14.58430163687958
C	-1.95991295619334	16.44735534956646	8.53908014197609
H	-0.95804857366231	16.83885948896095	8.32785115149772
H	-2.69174905853466	17.15541129968190	8.12712127705008
H	-2.06488539486009	15.49563208938513	8.00316180414747
C	1.28503033815316	13.93598126784419	17.29657025605560
H	0.95258499885276	12.88797523339835	17.32004866943555
H	0.84868326219496	14.43065670924550	18.17271916927065
C	9.42214965080987	10.65704573978225	7.11599390072377
H	9.55994544380612	9.81590988078501	7.80690757731791
H	10.15844459158678	10.55988781432366	6.30642361424962
H	8.42336808825682	10.56399746507543	6.67400361955394
C	9.39873025020494	13.15139694943601	6.85663745473820
H	8.40004639712703	13.13124913803817	6.40512868523510
H	10.13682273844827	13.09358078796129	6.04518940054483
H	9.51687241380747	14.11945106434885	7.35956197017619
C	11.02250627839446	12.06521215430692	8.39253925880160
H	11.21232850781110	13.01224181873402	8.91317187155290
H	11.74003009996437	11.99018019375785	7.56565420516953
H	11.23172297101611	11.24389962204880	9.08918411164436

155

[Cr(L^{MIC})$_2$]$^{2+}$_q_r2SCAN3c.xyz

Cr	3.68866077465337	12.47575245311484	12.00060705122430
N	3.35678692808055	11.48019315188422	7.80539784097464
N	5.70919294975480	12.44447085330560	11.99822152834891
N	4.85925329425821	12.00601795167993	9.13656218704398
N	1.66618311519360	12.52109811364400	11.99154638804541
N	4.64044778131770	11.73902525877460	7.84672521369909
N	2.43644144274241	9.82771644786478	13.10484655381841
N	4.88031519532403	12.90563317846446	14.86145028498417
N	2.56800807240986	15.14600559318835	10.81026679158484
N	4.09269591271978	16.52664531631286	10.53915270178072
N	3.85538302055603	8.31806552904299	13.21307088756367
C	2.75245259685497	11.56175002390510	9.02610760028244
N	3.40514374123099	13.50596142146804	16.19156216007463
C	7.90169712030839	12.34429882766235	11.27932210027890
N	2.59331923632054	8.55202719246018	13.46939764636325
N	4.67590903715235	13.19151324503741	16.14991606504569
C	8.91415965249330	12.29348279377177	10.33463675561879
H	9.94990866668705	12.30966576575091	10.65491039380067
C	6.53012073077028	12.33567189471831	10.90549388571849
C	7.23344577712970	12.16717040354814	8.60891552400283
H	6.96825404273036	12.06762799638114	7.56425896599101
C	8.58935344166283	12.22693556044015	8.97284311378074
N	2.81166180919729	16.36358408088365	10.32008929342162
C	6.20744769102242	12.19802529847907	9.54606362093109
C	-0.54084716161694	11.96269875011916	12.38568986120372
C	3.74029077751463	11.93201303649793	9.94289202327817
C	6.53272048947217	12.50510778680813	13.09633841362038
C	-0.51303219536151	13.21445634637160	11.66695018777527
C	3.66896031617725	14.50272216845754	11.34252068720089
C	0.86476868468924	13.50896963049249	11.47801714898453
C	7.90419262799956	12.42842544395439	12.72082229875204
C	4.67193649741869	15.45936725984578	11.16235657443622
C	9.64947296284803	12.18941689373264	7.87351746737943
C	-1.56263161718473	11.18937573468813	12.89986960160475
H	-2.59413589231784	11.50807244997675	12.78232818181479
C	0.82344808328992	11.58151685445711	12.52997100185889
C	3.57590032831384	10.43541010777732	12.61129931693898
C	3.76306360440084	13.02236087881514	14.05633789563412
C	0.07295753009406	9.59002779236826	13.66523642447074
H	0.32086577585239	8.65025929430767	14.13811670535969
C	1.29954447757517	11.23613882188140	9.16157952098028
H	0.72838079738046	12.16606655146430	9.28120571903362
H	1.14679692815690	10.65946853446747	10.07862501094228
C	2.79504180261913	13.44168793137432	14.97237852854865
C	1.11462096702019	10.34893432396344	13.12575631473151
C	4.51006799780094	9.39827443968103	12.69544355101860
C	2.74139345224070	11.08773634432396	6.52481845677910
H	3.07302215202211	10.06622944545655	6.30186756451251
H	3.13395628133794	11.75338708241562	5.75082668080495
C	6.21704653280305	12.65262631144139	14.45219986276560
C	1.22666377013407	11.15631878537489	6.65150455718835
H	0.78919134452858	10.67530063033169	5.77155796860027
H	0.89629772146908	12.20316391321021	6.64691012519449
C	-1.26298613689728	9.99358581639159	13.58084365071635
C	8.91120410211286	12.43039692518442	13.66603561884922
H	9.94849636209421	12.36570609563466	13.35129973172079
C	0.77533152403350	10.47184819821773	7.94079499871349
H	1.14265399917165	9.43692082802939	7.95633735229862
H	-0.31643622091450	10.42373956863620	7.98741595232985
C	1.21235529785395	14.71893481526072	10.85702081487440
C	4.73154083537453	17.80010402384730	10.16085487390345
H	4.39645328290100	18.56016082124147	10.87717307765079
H	4.36074245390506	18.07463197750036	9.16919917842361
C	6.11828127602307	15.51305423492255	11.53837576376121
H	6.69624355728889	14.88817966532933	10.84511550174946
H	6.24772869791432	15.07336711357284	12.53170804170889
C	5.93747093977477	9.26564503888777	12.26684455414840
H	6.50328919424187	10.14118913379259	12.58759214214505

H	5.96659675067743	9.26287351173809	11.16771346628377
C	-1.50840037273821	14.05062397825553	11.18777608219277
H	-2.54988227000515	13.79190982325371	11.34277418151068
C	8.58924594021117	12.50980892906752	15.03429837682559
C	6.65970413609187	16.94636236736588	11.50090827876618
H	6.28035126904339	17.51610414361101	12.35979836884656
H	7.74959917118347	16.92179012511874	11.59006330812286
C	7.24503753094753	12.63263996981307	15.39805922871843
H	6.97851490863401	12.73732163451127	16.44017122429915
C	6.24339437703900	17.63716476031314	10.20357838381336
H	6.58343934950035	17.05038679387162	9.34017317185696
H	6.69581969857568	18.62971429953013	10.11826051341087
C	4.41890471447836	6.98921852773034	13.50823450347965
H	3.64031795862655	6.24993463091109	13.30183338892781
H	4.65257646225550	6.95803440495784	14.57977182708527
C	9.70932531549733	12.47572538122518	16.07182114560942
C	2.81021842092343	13.93421557926267	17.47021974703818
H	3.17684591786275	13.25908449875722	18.24868569435134
C	5.67233607604640	6.78548308272843	12.67102932692207
H	6.16517978334075	5.86907071255044	13.00940142112000
H	5.39898478438717	6.63728261381783	11.61823159079400
C	6.59524749959913	7.99347643845336	12.81437220523049
H	7.53778553709626	7.82105843872594	12.28660116366549
H	6.84632722359423	8.13576756722292	13.87413503076814
C	-1.15912481747957	15.22240563006218	10.50254680683659
C	0.20302860510421	15.53851654620825	10.36481359288512
H	0.48619307391639	16.46490521069207	9.88194236694398
C	-2.39839875874167	9.16705856086850	14.17978252350053
C	0.86802215544222	14.62177130450033	16.05426413684121
C	1.35727965405791	13.82807583677382	14.83784347884651
H	0.74728182364840	12.92233064801010	14.72517763549045
H	1.22744740875628	14.40496952741019	13.91734280768393
C	10.48229477053231	11.14929345286735	15.92422365993260
H	10.93706978296724	11.04676479364364	14.93414075149139
H	9.82090194045418	10.29170196198297	16.08742015151902
H	11.28789816193491	11.10477849098473	16.66436173154962
C	10.66659544057354	13.65863607870506	15.82137077006367
H	10.13779748650295	14.61383596900829	15.90518370109923
H	11.13197282053073	13.61087013970830	14.83213272726528
H	11.47032351235304	13.64662547982683	16.56467026014308
C	-2.19931731703736	16.18076107855626	9.92582160342756
C	-2.04704848994443	17.55995504606357	10.59872641096380
H	-2.19466887467346	17.48405561013914	11.68107319422588
H	-2.79630636479592	18.25136966985196	10.19949083050705
H	-1.06112528460532	17.99904389453356	10.41783067780699
C	-3.63246330207454	15.68705369255823	10.15399085982574
H	-3.81284300207791	14.71620832705428	9.67924455088999
H	-4.33415104719341	16.40095299308711	9.71309610805712
H	-3.87452379464962	15.61015265776849	11.21967619942067
C	9.18351953864621	12.57348418871837	17.50793994963920
H	8.51374957963509	11.74267844993286	17.75602616667839
H	8.65758647975794	13.51756270838070	17.68811436728372
H	10.02623296655603	12.53256455488955	18.20415050383872
H	3.18496521077067	14.94275014180934	17.68375337514349
H	1.27757031836129	15.64053892536938	16.03062955963030
H	-0.22099035899606	14.71431221778355	16.00959515333257
C	-1.89653120779808	7.90696434954618	14.89316287215449
H	-1.38226308564124	7.22494606361904	14.20714500407919
H	-2.75004094471296	7.36503687920377	15.31078737347512
H	-1.22293924428997	8.14697901761382	15.72336631404007
C	-3.35829229987141	8.73814684180581	13.05111653138960
H	-3.80186506484060	9.59716764550288	12.53841829178261
H	-4.17770916942355	8.14395585910408	13.46859284090123
H	-2.83734013202812	8.12721145615846	12.30655845094357
C	-3.16330698912704	10.03290946973594	15.20132770001943
H	-2.50113074816462	10.36342995547563	16.00876060573222
H	-3.97656624207880	9.44921808053756	15.64464495430262
H	-3.60815811252035	10.91966302597715	14.73933092437334
C	-1.96812369914841	16.32096185431561	8.40778172027167
H	-0.98137942717322	16.73558388417245	8.17903345719462
H	-2.71773920324912	16.99419617641756	7.97936517131981
H	-2.05587594044900	15.35033168501113	7.90804380392964
C	1.29369009111108	13.92824861600299	17.34745579256671
H	0.91975530836111	12.89624001179644	17.36035815950701
H	0.87892135585222	14.43341662684186	18.22488568543889
C	9.51405766154248	10.87145870903441	7.08416707189848
H	9.64941825690769	10.00661950399545	7.74212866057644
H	10.27920681974146	10.82812880593952	6.30223370529103
H	8.53778710533288	10.78179392807410	6.59781843815868
C	9.43294686951564	13.38281336099051	6.92127313140530
H	8.45244016207749	13.34949481308790	6.43628453298474
H	10.19297087809230	13.36938405307182	6.13339891951184
H	9.51731135227208	14.33268408478942	7.45994706804918
C	11.07238272237058	12.27237277853516	8.43764464418136
H	11.23814197272124	13.20141540890276	8.99414667706058
H	11.78992992286936	12.25381559906163	7.61229297147717
H	11.30468129852086	11.42330630284648	9.08965982479380

155

[Cr(L^{MIC})$_2$]_q_r2SCAN3c.xyz

Cr	3.67808074288023	12.49162745940842	12.00714101388243
N	3.36514505988863	11.35313054639976	7.83218149467213
N	5.69988432289137	12.42912870092021	12.01223321637767
N	4.86674247308446	11.46979528610819	9.27530934116882
N	1.65917820876966	12.54057874869013	11.99881115975321
N	4.66142820410418	11.13103757691768	7.98919081326100
N	2.40176830521961	9.67268033613241	12.57263742549357
N	4.91350710997732	13.41719656727391	14.75156977941559
N	2.54011434427649	15.34243378016446	11.30996460101288
N	4.07028558282273	16.58196727949029	10.62409449092070
N	3.89558523107425	8.29366943681367	13.03702085883600
C	2.75630117761237	11.80830645288192	8.96932706995542
N	3.42017109253273	13.57903449634579	16.19668353536096
C	7.89526303857042	12.09496303858182	11.35561983901855
N	2.58781392249486	8.36883158643227	12.84261937664278
N	4.72598285358332	13.72962019991321	16.04562454750753
C	8.91459727942248	11.66445807522103	10.49886628324758
H	9.94782324339605	11.77284641082215	10.81068394261341
C	6.52094722120014	11.97567814703608	11.00814288591368
C	7.22898892578709	11.01714246693693	8.90753709357726
H	6.95527920673847	10.61427511864295	7.94118176056587
C	8.58528215087331	11.10230575853254	9.26993139303993
N	2.76702959655029	16.58931230678738	10.85795200350530
C	6.20117651986357	11.46392928211797	9.74175728126473
C	-0.56159417403363	11.89801464253154	12.14199814247490
C	3.73865361138047	11.90030840907462	9.95476335308599
C	6.53968274153668	12.85765770243797	13.01238905160829
C	-0.52389387016094	13.31003842881195	11.92470004445472
C	3.66003749294555	14.53037406802130	11.37624271712412
C	0.85598786702287	13.64352287300456	11.82886861149510
C	7.91057040670685	12.69126025401516	12.65413624899422
C	4.66176235336067	15.38475458983185	10.91634845168103
C	9.63846405949022	10.58064375584582	8.28791072627694
C	-1.60883241117097	10.98768426287531	12.28924144115960
H	-2.63458217823367	11.34403600432795	12.24410344678115
C	0.80211190962009	11.48255067751089	12.18897229154771
C	3.55327004795932	10.44195246464033	12.58033530150288
C	3.76492139780330	13.06864739667152	14.06090190986437
C	0.00875272288022	9.22514736192778	12.55019204090891
H	0.25359926355731	8.18751705850741	12.72276232359512
C	1.30434958233076	12.16740975062346	8.93062851585172
H	1.21074756948217	13.26256277167908	8.94982327711200
H	0.80846215981932	11.80646301528776	9.83245438877974
C	2.78790378616957	13.19012478029144	15.04920231078586
C	1.07247120457683	10.12696239205336	12.41435811580966
C	4.52525569919683	9.50118734434424	12.91588465607533
C	2.74357787754691	11.04886310371679	6.54721516038549
H	2.56130991754693	9.96590376082244	6.49255751609698
H	3.45735614931472	11.31306107531419	5.76013609672019
C	6.24645216951069	13.37423112703522	14.28030480732064
C	1.43381271885852	11.81629250505259	6.43191330950764
H	0.90343969379676	11.46947053515568	5.53839446252043
H	1.64185406380660	12.88619906537920	6.29669572213222
C	-1.32977507552183	9.63463331101188	12.48582167014439
C	8.94132897567479	13.08105388786741	13.50944010889065
H	9.97327993822365	12.93971196400853	13.19912427728294
C	0.59895640923365	11.60298932815555	7.69293927692453
H	0.42137687800689	10.52709726669919	7.83020534766278
H	-0.38294958529456	12.07657102785627	7.59236838797876
C	1.19703927296781	14.97726235070346	11.55949450398458
C	4.71470436781975	17.80113952037298	10.14445355120433
H	4.88676790080490	18.46812882100555	11.00127308579003
H	4.01964059160827	18.29842123532500	9.46023812812637
C	6.12077743944971	15.19805040478287	10.64603512782135
H	6.23794596900137	14.53970257543884	9.77359576522433
H	6.58797865759763	14.67466715816032	11.48159114356896
C	5.99694375042456	9.59333743029538	13.16307980788141
H	6.21052585271707	10.49787862332352	13.73786622219743
H	6.52507324015846	9.71099658368607	12.20760857694304
C	-1.52757519332066	14.27877901318784	11.80834440972299
H	-2.56569408909036	13.97612171077254	11.89317278688184
C	8.63866383287930	13.64792561406263	14.74831520608248
C	6.84284051966536	16.52722574721976	10.40059172138218
H	7.00742051144591	17.03980027486532	11.35880812263664
H	7.83125358803994	16.32459450110283	9.97573355905780
C	7.29377907832050	13.77756703607808	15.11909900524546
H	7.03205159525385	14.18243279625429	16.08568550999286
C	6.03429109208247	17.43692877999286	9.47818380757591
H	5.83935394402290	16.92677074918202	8.52533093022498
H	6.57792559568977	18.36045901270749	9.25131155383726
C	4.48377487290202	7.00989418857812	13.41043334421685
H	4.02995944434771	6.23459212361271	12.78396382277195
H	4.21844632675662	6.79941845585472	14.45621266323179
C	9.77627062178372	14.08978896399415	15.67557325820860
C	2.81672538779846	13.86683074542209	17.49476871952554
H	3.51705708351729	13.53597897265706	18.26843866894799
C	5.99606454855633	7.07983415321040	13.24643777929905

H	6.43577842636837	6.18530951995826	13.70062135370454
H	6.25718645913920	7.06910506564825	12.17981368190298
C	6.52934931809478	8.35661992431980	13.89378182408930
H	7.62382544300159	8.37158239882209	13.87474902045694
H	6.22543411801948	8.38263069044508	14.94992900519425
C	-1.17723508728082	15.60665313566529	11.58924528074193
C	0.18475094239122	15.93429282509932	11.46124571571782
H	0.47588924715791	16.95708840906657	11.26067907926505
C	-2.48424781834687	8.63756339849553	12.63398042054025
C	0.64473209098439	13.47108908688815	16.33567736947942
C	1.31711672695691	12.92013174767266	15.07363722673576
H	1.15577785677908	11.83533009015351	15.00236849306259
H	0.84681755482813	13.35222842095135	14.18906784096077
C	10.64083746951206	12.86789383956319	16.04087008107755
H	11.08378737062715	12.40785671311524	15.15226420101536
H	10.03487292233559	12.10749079056951	16.54535067937866
H	11.45667875053133	13.16227641471525	16.71232206081602
C	10.64947741235761	15.13395106763045	14.95438804488380
H	10.05126443362762	16.00779433397243	14.67395322605771
H	11.09485946308740	14.72669755667588	14.04157925824474
H	11.46533499954207	15.46814027769760	15.60693120583895
C	-2.21335193419684	16.72836899530170	11.46965113512368
C	-1.96795713890685	17.77468808698050	12.57363236529517
H	-2.04649587109243	17.31277341361050	13.56387846666584
H	-2.70908400334118	18.58046490183233	12.50750293853165
H	-0.97318153684947	18.22314787327264	12.49059040949725
C	-3.64876724987525	16.20980438073626	11.61614669367187
H	-3.89475343451955	15.47817690444827	10.83882999841611
H	-4.35108623560355	17.04523201226969	11.52228124249623
H	-3.81242854327388	15.74475830389809	12.59443652338986
C	9.26543070028815	14.71704992011756	16.97818280854382
H	8.67187475102332	14.00722842779044	17.56448635117552
H	8.65253751505861	15.60458738085675	16.78644558746777
H	10.11776269763765	15.02563090262832	17.59342592174436
H	2.69176116119705	14.95482567939524	17.59052567079167
H	0.52306012193238	14.55944615613519	16.24277197253327
H	-0.36047989553696	13.04587638137108	16.42084476704390
C	-1.99776540101301	7.20113485009006	12.86105477968959
H	-1.39345329517182	6.84095362601149	12.02139913868880
H	-2.86199439973819	6.53526974510213	12.96004551945031
H	-1.40337426838514	7.11484318955071	13.77718474189780
C	-3.34174056251573	8.65409826674794	11.35390084020069
H	-3.76658350093004	9.64511306645253	11.16702008296482
H	-4.17090458206617	7.94126921303632	11.43971552686829
H	-2.73702986263649	8.37842241116147	10.48308722071220
C	-3.35950870018130	9.03993518308563	13.83627634087432
H	-2.76692827892713	9.04720143550288	14.75758553881075
H	-4.18668561709049	8.33066234782974	13.96193422949976
H	-3.78910591275926	10.03773847858633	13.70591124579000
C	-2.08873463953749	17.40429646947838	10.09080239918224
H	-1.09728983639192	17.84325154647526	9.94402621367583
H	-2.83063284331227	18.20576675817793	9.98917482547587
H	-2.25550315729433	16.67574504706513	9.28983158509553
C	1.46901023019972	13.16456937195858	17.58422938547713
H	1.62112864132509	12.08078803200513	17.67669401916388
H	0.95882602403314	13.50308965662181	18.49254061489133
C	9.41594293373683	9.07440953958215	8.05197720487052
H	9.49992434482860	8.52319435268373	8.99499795266731
H	10.16416199208931	8.68085665667448	7.35318736758103
H	8.42432290741023	8.87449580104607	7.63431563577261
C	9.51802306677298	11.32932626956074	6.94698040991729
H	8.53637016115996	11.18290871476197	6.48636914045009
H	10.27766660022591	10.97208984075665	6.24105521896912
H	9.66218611338775	12.40533513684860	7.09366372900634
C	11.06646127951765	10.77546737531575	8.81145710241519
H	11.29459140252807	11.83447990919163	8.97448334878590
H	11.78156705904933	10.38925260339197	8.07700627296956
H	11.23060590827190	10.23714211466449	9.75128194750360

155

[Cr(L^{MIC})$_2$]$^+$_r2SCAN3c.xyz

Cr	3.68656874240847	12.47524042271146	12.00743699854634
N	3.36576247014123	11.42674788771057	7.81492273483248
N	5.70474441939503	12.43564130085534	12.01212825820103
N	4.88479227147666	11.86282242162205	9.15958909862511
N	1.66708865041337	12.51870117005608	11.98918874939174
N	4.66840838018664	11.58090959913986	7.87257052594169
N	2.41175816236196	9.75437511519338	12.94714860275341
N	4.89088926298534	13.03995636655678	14.85951782084574
N	2.54912631496964	15.20348243802813	10.92056627338463
N	4.09649000656272	16.53973289031547	10.56755970320204
N	3.86279807773349	8.27668504352463	13.07575061530796
C	2.75221693334246	11.59126633123038	9.02151025068507
N	3.38472976187877	13.54690788985199	16.19391460723014
C	7.90704593774024	12.27444233332425	11.31123183890468
N	2.57368625772068	8.46210864492646	13.24170797631188
N	4.67979540843833	13.33673216261451	16.14379778903054
C	8.93192325337143	12.13062963830852	10.37927361098183
H	9.96263672826728	12.17077841289824	10.71495437205018
C	6.53588821103835	12.23843896371466	10.93537257040327
C	7.27056328203565	11.87946627332303	8.66472865402009
H	7.00851526004229	11.70813073877578	7.62842757011067
C	8.62446858552936	11.94242797162423	9.03411216772747
N	2.79589384001835	16.42145518578205	10.43282272894609
C	6.23580809139213	12.01154765127869	9.58474089663744
C	-0.54976484499938	11.94146679031049	12.34035203108687
C	3.75361503490215	11.90562780929750	9.94738893482989
C	6.53418601968150	12.58978108031464	13.09845903714903
C	-0.51960231681283	13.24103704697061	11.73355114452805
C	3.65896738065004	14.51422056562657	11.36283543323692
C	0.85905274735319	13.54391773454533	11.55791210932898
C	7.90824020103345	12.48848355359405	12.72972161659316
C	4.67939126205105	15.44327040225525	11.13122211458006
C	9.69295102684320	11.78699286700731	7.94911882248793
C	-1.58371576742129	11.13002773876518	12.78756996742659
H	-2.60987239766071	11.47652611442060	12.69847840436750
C	0.81515868235846	11.54413382403479	12.45006040064241
C	3.56453417778733	10.42063155009189	12.58571535461317
C	3.76237317757821	13.04199260574723	14.06563499003493
C	0.03450770254192	9.46995605400209	13.41292261804066
H	0.27932152571753	8.49481727469853	13.80968346839118
C	1.27189665877983	11.41151816744436	9.13047420081704
H	0.79624188300604	12.39468104943321	9.24235900552635
H	1.04156466317857	10.85848224469905	10.04461332259747
C	2.77121353930762	13.40328407619947	14.98480396478626
C	1.08413675956703	10.26767667735642	12.95460719553309
C	4.52216750901144	9.40479651702282	12.68664847580457
C	2.74440746243904	11.05987460053689	6.53481145858865
H	2.96213459089994	10.00037948118784	6.34929114456458
H	3.22546560351596	11.65125174597336	5.75019390689510
C	6.23607178226207	12.83118606083433	14.44374272045563
C	1.24377010616291	11.29819224208191	6.61689459522532
H	0.77614579207694	10.85069353059296	5.73414335629671
H	1.03303896271202	12.37531748233386	6.58680717088579
C	-1.30187059082561	9.88334293612514	13.35220916413063
C	8.92608295059615	12.59098801033482	13.66868773290375
H	9.96023589815722	12.50471453624904	13.34599869458069
C	0.69118280220635	10.69529577534237	7.90688763848711
H	0.93785752086667	9.62523943367763	7.94472824255913
H	-0.40003546918533	10.77198266762169	7.93101138741305
C	1.18900693183310	14.79144751915867	11.00992562049509
C	4.74842296293826	17.79043935458668	10.15503975963810
H	4.52142110923260	18.55211184693736	10.91183070000017
H	4.29780751583509	18.10284660623144	9.20841176241837
C	6.15207342831956	15.43713189887535	11.38867597434311
H	6.64285407915563	14.77831995269558	10.66060311766192
H	6.34610844653939	14.99954203435602	12.37156114586609
C	5.98971751711401	9.34671062820035	12.40428332811175
H	6.48395253164745	10.20905856176691	12.85489286590445
H	6.14089751577507	9.44252990097558	11.32016555707191
C	-1.52408963606151	14.11968602036268	11.33524993288096
H	-2.56178416275766	13.84120664264546	11.48458826815825
C	8.61795822371114	12.80081652359089	15.01486886770192
C	6.75548096593463	16.84216219216032	11.29513107981589
H	6.49228330986476	17.42744094254166	12.18701871365914
H	7.84663881288166	16.76244744280713	11.27711452198208
C	7.27087433828892	12.92350587619591	15.37565448405266
H	7.00444434122550	13.10863176900851	16.40638729264062
C	6.24890299526298	17.56251987487826	10.04719863034858
H	6.47372164615834	16.96503375031921	9.15390306497469
H	6.73618582666492	18.53443691714097	9.92053265276150
C	4.43520694993129	6.94770774332596	13.32530387652690
H	3.70354223732087	6.20623039248323	12.99200478013000
H	4.57208688820177	6.83471169299275	14.40849570035783
C	9.74523244549013	12.90353561075779	16.04637126861634
C	2.77374290458756	13.94821152373638	17.46846830786493
H	3.22202235631692	13.33882265625603	18.25873270845453
C	5.76634326906278	6.83667487405733	12.59709751874357
H	6.25441082246769	5.90789390062507	12.90916348841621
H	5.59440186227542	6.76794693531328	11.51480586003420
C	6.63242967062875	8.05297941641859	12.91667390196916
H	7.62656831320622	7.94638686221672	12.47209326011443
H	6.77637749725229	8.11827042746844	14.00386780017442
C	-1.18736030559407	15.33682750324227	10.74806591048790
C	0.17415490890285	15.65278996407983	10.60616462634206
H	0.45727738227582	16.60732029047357	10.18095986264398
C	-2.44528726196178	9.01237036169816	13.88101810001176
C	0.74769948299372	14.39967036800929	16.08146272286287
C	1.30166715966167	13.64865898389490	14.86640855152270
H	0.78127141356614	12.68806235327425	14.75935373037830
H	1.10269471617933	14.20547574575750	13.94724461327003
C	10.55362708869932	11.59173235343463	16.04785989225618
H	11.00815574721600	11.39391190373810	15.07212052532320
H	9.91113209221566	10.74135642899111	16.30057903066617
H	11.36023350030842	11.64505914948478	16.78755584568558
C	10.67233545380228	14.07517606431218	15.67092945022120
H	10.11550922360352	15.01816801930187	15.64932369937934

H 11.13128251267994 13.93135456858484 14.68790372464760
H 11.48064933649054 14.17004681663000 16.40479262580820
C -2.23246121766028 16.34553312951825 10.26481484459854
C -2.04815547431277 17.67981358019783 11.01305574505549
H -2.15848602194009 17.53661057472442 12.09330254950881
H -2.80120325345117 18.40466597945736 10.68409556397884
H -1.06234337961560 18.11798394296150 10.82902265348882
C -3.66380781389704 15.85431427715299 10.50957714966978
H -3.87057145903473 14.91907284511466 9.97774187453806
H -4.37341609957857 16.60418052456383 10.14597920289588
H -3.86303549469638 15.70126807714201 11.57591701456806
C 9.22060413073416 13.14442203415905 17.46664436060484
H 8.57630545490549 12.32624741757183 17.80709350114636
H 8.66193083893220 14.08393622713814 17.54094201621330
H 10.06586127066174 13.20830048509160 18.15918177987644
H 3.03977333504457 14.99723636696161 17.65057753827255
H 1.04427027492911 15.45680036020617 16.03849897410221
H -0.34579805672878 14.37354005571500 16.05113796005208
C -1.95582923210741 7.65882953208637 14.40882618651206
H -1.46550099753523 7.07043389797394 13.62539282451491
H -2.81126986527198 7.07958985987374 14.77074237581752
H -1.25953387137067 7.77493214536464 15.24666661512672
C -3.45895573336672 8.74839365504083 12.75156150590417
H -3.89722005864195 9.67589196579702 12.37107525584559
H -4.27847888703294 8.12144542814379 13.12020847534834
H -2.98076123341488 8.23072270169860 11.91312170488504
C -3.14809113115332 9.75460620246981 15.03435465244089
H -2.44569690005667 9.95123643287834 15.85163225347396
H -3.97311515272220 9.15012156283814 15.42778013315929
H -3.56146120819910 10.71323889425442 14.70534059509393
C -2.05365975448972 16.57863257944529 8.75238590262933
H -1.06308337981424 16.98125469881204 8.51811880239238
H -2.79988607640152 17.29359524168121 8.38818507121624
H -2.17744620827751 15.64145039541774 8.19892661816633
C 1.26407813684942 13.77980351602346 17.37844482958320
H 1.00317884114553 12.71385493490766 17.41321908269338
H 0.81265541512637 14.25396933763721 18.25568831940678
C 9.53796401668017 10.41327207495338 7.26889117914326
H 9.64820617713823 9.60478343013788 7.99962226279483
H 10.30400333568779 10.28436430365372 6.49593948290660
H 8.56007869179530 10.30469906452889 6.78941862796026
C 9.52061771879946 12.90038292933140 6.89805613524823
H 8.54215985102007 12.85053980221897 6.41001163688781
H 10.28673851168786 12.80849500675473 6.12002897889172
H 9.61839180956393 13.88821745710490 7.36123762043371
C 11.11226378955825 11.88178086720902 8.52027506713700
H 11.29572958674954 12.85207813102025 8.99478056881104
H 11.83913847314705 11.76856349490917 7.70973703567537
H 11.30956758734122 11.09137531206534 9.25271650811120
155

[Cr(L^MIC)₂]^2+_sUKS_PBE0.xyz
Cr 3.68291669359470 12.48545547630001 11.98907754622213
N 3.26545722080356 11.14270382830519 7.96185379223634
N 5.59884058249561 12.42872835235302 11.97966798445142
N 4.75161750621516 11.22731570121728 9.38084468685051
N 1.76794501491942 12.52509359839462 12.00011935309610
N 4.49185662569630 10.77969338615567 8.17552880571189
N 2.49788197152496 9.65695660080117 12.35433073492212
N 4.85940741439943 13.68609025700388 14.58000543275186
N 2.62096621588512 15.36237044240548 11.63327335524873
N 4.11333406294202 16.59884801067929 10.94477078321393
N 3.95579950713223 8.36314988521669 13.01259124021333
C 2.72111132173742 11.82522518726476 9.00654653635831
N 3.39728185248858 13.85771079938339 16.01642980149905
C 7.78511756798699 11.95868184989876 11.36350855810820
N 2.71706887072671 8.39213673777560 12.62699656021381
N 4.63966790633159 14.15032053314629 15.78715450093026
C 8.78255979631190 11.38677358154841 10.57839645394451
H 9.82468632567794 11.51327306474189 10.86019670527095
C 6.41681134187050 11.81608396022377 11.04389485627960
C 7.08176062515643 10.58165486132353 9.08379403651104
H 6.78591486606798 10.08503918623516 8.16383984226035
C 8.44030467834539 10.65980008153697 9.43371573650479
N 2.88180484433366 16.61672124143417 11.35027566038963
C 6.08134873083173 11.16391615839730 9.85551220524568
C -0.45224984093311 11.85105608050231 11.98250789822253
C 3.71089705304362 11.87409303539439 9.99360777376136
C 6.46369568037873 12.99184642736927 12.90396989296607
C -0.41951560146889 13.29683672012314 12.02521526780312
C 3.66571778993197 14.49776308418782 11.43928347514622
C 0.94945555923743 13.64380764056410 12.00157037477010
C 7.81917052098895 12.76149904400743 12.56592508429187
C 4.66038091646448 15.35233154560928 10.95389730393469
C 9.47901497423272 9.99372178005572 8.53287125467928
C -1.48543685047638 10.92494399604623 11.93243335490689
H -2.51762892646273 11.27016552011366 11.91095663492343
C 0.90296174954931 11.44210404008333 12.00163828150021
C 3.57582562705481 10.48054296051905 12.53639104014791
C 3.77534227851685 13.09835419718018 13.98139124858943
C 0.13728228355495 9.15301245346687 12.00598116160325
H 0.39342022166947 8.10022458463735 12.06281949769670
C 1.35920333882035 12.42158392318944 8.87165962650766
H 1.47802557667481 13.50386599260116 8.71067148772453
H 0.80806771542730 12.31256805391998 9.80479971052334
C 2.80246799458922 13.20471937218212 14.98035301645635
C 1.17971872623393 10.07797053046131 12.07282074229876
C 4.54403855951678 9.59030586419986 13.00611654225160
C 2.60635025869273 10.77056402784052 6.71198395149648
H 2.28719221378146 9.72441308166686 6.80745253295100
H 3.35912562524681 10.82369886089168 5.91970439054576
C 6.18513978800450 13.66489247463468 14.09196918407524
C 1.42122598408283 11.68487670550870 6.46493256956423
H 0.84158545982144 11.28041547639900 5.62765970966028
H 1.77542556419411 12.67783691582561 6.15343873320911
C -1.20191737254605 9.55100120237681 11.91083962204941
C 8.85424737987094 13.26641625802478 13.34065470396883
H 9.88601402451810 13.07490467870061 13.05065290493996
C 0.56861165271367 11.80258147237473 7.72165968863807
H 0.20511874554299 10.80753754884376 8.01843951185345
H -0.32106777041347 12.41095073604311 7.52580039637942
C 1.28715553683389 14.99677953284080 11.92487398360433
C 4.77154289607995 17.84650018498446 10.56391573796592
H 5.09678326373367 18.34409693897661 11.48688256455504
H 4.01706071905316 18.47815183413660 10.08559190653997
C 6.03219002699863 15.13654989243692 10.40612915590892
H 5.93267042851348 14.64520369091041 9.42646752984851
H 6.58168744278658 14.44106505757178 11.04102809844947
C 5.94838695481661 9.75233552864836 13.48155737688067
H 6.02504181170281 10.67741530996721 14.06247953461226
H 6.60794401404350 9.88054904007207 12.61266843862951
C -1.41761697995389 14.26696425528460 12.07268203080486
H -2.46052393894690 13.96170343181865 12.09583979438828
C 8.57277661571644 14.01290071649899 14.49504949178979
C 6.81231324404151 16.44170966099918 10.26596752677953
H 7.17658033386993 16.76019274482369 11.25387469930301
H 7.70149507942922 16.26686253108723 9.65052342658968
C 7.23119125144971 14.18073862479189 14.85921144444450
H 6.97469178099489 14.69457019779200 15.77957681665403
C 5.95183443292195 17.54312821682688 9.66039493345892
H 5.59291536453068 17.24291231025384 8.66548492493748
H 6.52736707216223 18.46523248076533 9.52209814656523
C 4.54983440362064 7.10100373434362 13.44990557901881
H 4.25554539859572 6.33109365415952 12.72980984683057
H 4.09936250789440 6.84829929888279 14.41821412193779
C 9.71941666476157 14.58209379955814 15.32906811796142
C 2.77658829124758 14.26545085991532 17.27497923244572
H 3.53615370485796 14.17066177649493 18.05681343017673
C 6.05577891222375 7.25216544768217 13.56685993345328
H 6.44864652281358 6.37646575764568 14.09541813237859
H 6.51283267251471 7.24922019105981 12.56685190221129
C 6.40859597873172 8.54428298403832 14.29373645541844
H 7.48932445607647 8.60866248301310 14.46002731799249
H 5.93812199668892 8.55127850417140 15.28798800334868
C -1.07443310703997 15.62210117409846 12.08723073031283
C 0.28614136925094 15.96005455036934 11.98948403915584
H 0.58278076800158 17.00328997748966 11.92504803044066
C -2.34610292792182 8.54282880531470 11.81628429501800
C 0.67204478504221 13.34694604151996 16.29304773452986
C 1.41138781941613 12.68468952983257 15.13325593270716
H 1.47295223465403 11.59754810248529 15.29304437342775
H 0.85514287656042 12.82410267124731 14.20696795401370
C 10.62248040803434 13.42873563640526 15.79571312037694
H 11.06290930373452 12.88106645819127 14.95426770714192
H 10.06285160929829 12.71304214337067 16.41042378093618
H 11.44985968303213 13.81901837356333 16.40049319424885
C 10.53128290965368 15.55454222250213 14.45863980824572
H 9.90878034162291 16.38800526525181 14.11010244986522
H 10.95871270540030 15.06039892243113 13.57807195745621
H 11.36349144299521 15.97434888109209 15.03657601395767
C -2.11297449582004 16.73994303145022 12.16417449767430
C -1.82742043524426 17.59737795324781 13.40685846823426
H -1.89282265408231 16.99720901422825 14.32299140926472
H -2.55970018527781 18.41028567486509 13.48414725381241
H -0.83134288305442 18.05459778567027 13.37138432972740
C -3.53679710087748 16.19042125953942 12.26794216476240
H -3.81574278879157 15.59935641097581 11.38664690367437
H -4.24768606743212 17.02156014142460 12.33713857473272
H -3.67334487835806 15.57014558958520 13.16270197813257
C 9.21771378265932 15.33418714620134 16.56291211868843
H 8.66139287010422 14.67925594341612 17.24528418561962
H 8.57801969424963 16.18494468310714 16.29612582074398
H 10.07164464351735 15.73333395872534 17.12196258347926
H 2.51387659384985 15.32754982691134 17.18369414062101
H 0.36150151395684 14.36094364314515 16.00021904841514
H -0.24765379696859 12.78958713442837 16.50173867926537
C -1.84439505705383 7.09801483996765 11.82478817887424

H	-1.17501354967455	6.89019816176600	10.98048519101347
H	-2.69551471163765	6.41284264151213	11.74055191570719
H	-1.31976614446817	6.84955682353418	12.75598762002531
C	-3.10994544270489	8.78882579538624	10.50541946697177
H	-3.53272143549327	9.79939464184412	10.45952265112647
H	-3.94119824190327	8.07951592563674	10.41116957377424
H	-2.45375940834095	8.65692636751957	9.63598840005476
C	-3.29117203038109	8.74017089941948	13.01203407245432
H	-2.76493780629483	8.58274164986979	13.96148389893546
H	-4.11891355247865	8.02263750322910	12.96111744131846
H	-3.72945202434546	9.74479798160898	13.03134770851056
C	-2.01398317793417	17.60818047335658	10.89961659483632
H	-1.02937355983055	18.07983516597464	10.79826684578348
H	-2.76084779824340	18.41050975500679	10.93328402295905
H	-2.19870006303810	17.01233986279173	9.99736538644423
C	1.54663034627572	13.41741438478782	17.53822697740805
H	1.84959631971174	12.40650537701719	17.84608339402413
H	1.00134124699476	13.85348474206366	18.38270145304677
C	9.16585493366771	8.49299874696955	8.42957089180283
H	9.20339553471329	8.01317191459197	9.41548275055663
H	9.90177405059721	7.99666844621370	7.78545058274366
H	8.17450068605140	8.30640365361702	7.99961045328529
C	9.41600508321508	10.63409661884679	7.13689524288566
H	8.43267005485759	10.50870492925019	6.66804110480289
H	10.15771111984812	10.17116169374220	6.47476323962478
H	9.63217455018894	11.70827590029366	7.18642115222172
C	10.89784108764516	10.15918303277027	9.08031848134479
H	11.19907696990487	11.21290222195710	9.13102605584278
H	11.60905947288520	9.65218924995791	8.41843819686483
H	11.00830520876408	9.71580990766738	10.07793739734478

155

[Cr(L^{MIC})$_2$]_sUKS_PBE0.xyz

Cr	3.68738861108582	12.49064533211046	12.00802451469015
N	3.31892676109819	11.48727596047965	7.87519485786315
N	5.70129528520874	12.45384727501396	12.00787064236385
N	4.85030619712426	11.86253465109131	9.20028810712164
N	1.67152976353564	12.52968086494954	12.00203515845997
N	4.61168267606491	11.60054762982782	7.91851290203261
N	2.45132592853417	9.79823290694811	12.94290428222185
N	4.87332980630536	13.08291859700323	14.81548390783392
N	2.56655444400548	15.18964355236609	10.96344549326266
N	4.11761377886877	16.48565716984239	10.56895454350741
N	3.92856302758712	8.37909956755570	13.15702714974427
C	2.71712429934449	11.65801677691167	9.07806614453032
N	3.35953377751266	13.54103248570723	16.13468767432044
C	7.89757065914243	12.26452577892071	11.30595823501655
N	2.64450407686558	8.52672192975115	13.27978902992725
N	4.64618839730730	13.37158153589351	16.09317674383602
C	8.91142417296635	12.08341047393328	10.36359173561259
H	9.94965099280806	12.12343745306100	10.68561194325090
C	6.52014487805038	12.22923096498796	10.94460044814300
C	7.22134299468459	11.80615302030573	8.67616992634701
H	6.93143413118472	11.61374419971616	7.64694102934489
C	8.58399103495337	11.85833718398959	9.02732569086008
N	2.82568883132658	16.39880741440398	10.47444561459085
C	6.19518445912351	11.97682648038136	9.59990987525539
C	-0.54095888018193	11.93046219272476	12.32297023265104
C	3.73515841571955	11.94375014964525	10.01504558638255
C	6.52528229951949	12.63070060849639	13.07889509453728
C	-0.51372601386801	13.24853118726104	11.75189767120181
C	3.66759219282507	14.45642719632714	11.36943906614905
C	0.86939180373503	13.55159927509281	11.59461742317667
C	7.90305073014545	12.51663175174612	12.72055291315401
C	4.69934653710269	15.38481075628518	11.10581906283895
C	9.63421901899307	11.65748037367299	7.93307144467215
C	-1.56737403171294	11.08670171164779	12.73807873150269
H	-2.60164643733478	11.41967875822443	12.65143499615090
C	0.83065580245805	11.54849591717862	12.43012135106587
C	3.58758034166139	10.50638273850319	12.59333750675252
C	3.75564307493044	13.04089819914758	14.00084337927237
C	0.07898983336405	9.43883910583877	13.33364974770913
H	0.34946792396019	8.45783586717984	13.70797613011265
C	1.23586007632671	11.51973421362663	9.19221059088895
H	0.78963996910123	12.51041322834028	9.35585128958647
H	0.99733658816313	10.93982263856717	10.08897314835342
C	2.75135097491407	13.38095198243816	14.93370982655764
C	1.12336536785206	10.26539807497414	12.91700565984998
C	4.57007623474367	9.50434564787874	12.75659590833127
C	2.68413887099642	11.16148898308490	6.60930528557905
H	2.85625066682683	10.09521166920451	6.40825169110316
H	3.19500470169674	11.73556727762192	5.82940313928789
C	6.21132159180353	12.89708863159172	14.42033123831616
C	1.19892865223948	11.46326567440999	6.68251223752593
H	0.71333714090206	11.05535408372371	5.78758903623109
H	1.03868839161873	12.55121289549218	6.66846428592420
C	-1.26843911789902	9.82649617808574	13.26517703515757
C	8.91576715949907	12.63443379198059	13.66911371895420
H	9.95526044067312	12.53165139212312	13.35758388599705
C	0.61443246282466	10.87201472558960	7.95843328342449
H	0.79579848850045	9.78615933305471	7.96970732622280
H	-0.47309573983404	11.00740326625364	7.98673273602044
C	1.21589273735822	14.80795340305882	11.06657581306035
C	4.77096854310453	17.71394150791424	10.14901873699664
H	4.58969429097271	18.47491789758705	10.92041785120718
H	4.28015306960728	18.04748778697103	9.22887325019529
C	6.17758697545321	15.35183807660558	11.30488490424053
H	6.62812634998371	14.67054672107122	10.56985134527290
H	6.40145875259030	14.91684841951452	12.28358112613287
C	6.04028588501128	9.46800883044280	12.50106062901613
H	6.51387915059199	10.34128071163371	12.95502367860955
H	6.21134181071048	9.56759443100110	11.41932938876049
C	-1.51216411842530	14.14446336429027	11.36660252686485
H	-2.55542426055288	13.86687746700821	11.49937261211468
C	8.59613776506092	12.87471351703124	15.00866555276935
C	6.81196794062472	16.73351247438144	11.17751674272497
H	6.61579916505644	17.32536726307858	12.08485770394812
H	7.90052383093815	16.62708388442401	11.10304959152324
C	7.24225901635877	13.00854395742720	15.35409159235924
H	6.95418639790059	13.21354536105654	16.37901949652900
C	6.25806461688283	17.47348869834437	9.96736561437861
H	6.43107662593691	16.87986583883827	9.05788833322968
H	6.75495354834674	18.44015307778773	9.82026157849259
C	4.52281489412460	7.08825009501895	13.45685762951025
H	3.80321433250434	6.32177746280455	13.15226859883478
H	4.65417282776949	7.01387644685112	14.54532329369288
C	9.71272618678688	12.97691111272973	16.05125613433336
C	2.73960581817300	13.91258478798409	17.39517677293622
H	3.22476838373715	13.32830827396559	18.18387511484677
C	5.85700442987079	6.96420416643222	12.74545311452638
H	6.35519444726243	6.04799171166750	13.08533817156366
H	5.69132471893231	6.85920316265993	11.66316720059632
C	6.70377355331695	8.19780945635128	13.02736315414649
H	7.69928534483869	8.09730726692153	12.57924562409032
H	6.85917117793052	8.28462821249498	14.11359968948349
C	-1.16335247290295	15.37521954795963	10.81222302433794
C	0.20449733567638	15.68220511000875	10.68026398311204
H	0.50958320216713	16.64169909194737	10.27188277999720
C	-2.40010442622647	8.91573734821665	13.74925067871908
C	0.68359908536495	14.27058950348685	16.04031528516775
C	1.27767939669666	13.57830842888229	14.81737794016496
H	0.79068705808465	12.60448261757850	14.66940650736220
H	1.06348406606005	14.15390896833359	13.91195826454911
C	10.47600571933814	11.64397398626952	16.10352094506051
H	10.92417927240837	11.39742095505824	15.13376393301784
H	9.80221446076068	10.82234127459758	16.37699119071241
H	11.28395812327486	11.68741041514616	16.84652799892454
C	10.67963678748621	14.10426195831464	15.66010755413686
H	10.15693013388331	15.06804253130888	15.62000695396365
H	11.13041351509862	13.92957669883507	14.67618129812791
H	11.49541796685286	14.18738352467545	16.39111802702419
C	-2.19783220043694	16.40177168084575	10.34701308258303
C	-2.00842679140117	17.71371132838997	11.12375073456804
H	-2.14009784559409	17.55011140304474	12.20060647009023
H	-2.74120854945661	18.46492042407577	10.79921106776313
H	-1.00792116296726	18.13494392970442	10.97130475649174
C	-3.62994346290525	15.91477215740842	10.57528008361948
H	-3.83839849067052	14.98995467217005	10.02280653111057
H	-4.34214427596778	16.67492967637691	10.23034738242733
H	-3.83005004208967	15.73093907067722	11.63828732974016
C	9.17337646273336	13.27523264230868	17.45182916486194
H	8.49748606718885	12.48617660142601	17.80400881368676
H	8.63159055905989	14.22869422714919	17.48442211068710
H	10.00591067953282	13.34110240434074	18.16368965002808
H	2.95826738634943	14.97314704218801	17.58054671306347
H	0.91021367393218	15.34759271298894	16.01211474700979
H	-0.40866201300706	14.18029344524215	16.01313097862235
C	-1.88523313860185	7.55804923417719	14.23177196420023
H	-1.36674624152613	7.01381321536826	13.43250959434983
H	-2.72727146749792	6.93827427657568	14.56503465299659
H	-1.19475347979836	7.66148018372498	15.07795114001535
C	-3.39224926798460	8.67097249019765	12.60253253771382
H	-3.83177095125262	9.60676293952685	12.23828834230418
H	-4.21465456120098	8.02320925966462	12.93506472853642
H	-2.89457685813952	8.18428794170149	11.75447967496920
C	-3.12914761733537	9.59810759050080	14.91730631126140
H	-2.44105285932250	9.77284282708558	15.75383755698855
H	-3.95692876464655	8.97273220207350	15.27877192570467
H	-3.54494243669821	10.56829684914249	14.62054206984586
C	-2.01087638186583	16.66602222621542	8.84523657455246
H	-1.00691583352075	17.04733078400674	8.62459324705778
H	-2.73845232583762	17.40717347953423	8.48754768988988
H	-2.15047423368594	15.74310384444775	8.26837508089495
C	1.24238423468463	13.67554162187406	17.32600930346301
H	1.03378405370518	12.59618581937521	17.35834130659802
H	0.77559426388732	14.11947610100458	18.21378843731709
C	9.44814737475040	10.27398995120726	7.29098719305470

H	9.56459883822418	9.47969838856584	8.03891186041391
H	10.19148792558599	10.11072967240222	6.49883359937425
H	8.45319967073400	10.16641444500319	6.84345518767326
C	9.46544157897367	12.74475199356083	6.86071107170703
H	8.47163444832285	12.70565664739333	6.39943815734554
H	10.21101246560821	12.62348164246647	6.06316890674459
H	9.59028189626813	13.74325113753713	7.29806810022442
C	11.05843246953144	11.74207420843425	8.48440669304127
H	11.26200328546911	12.72173537668320	8.93473155198599
H	11.78207328472544	11.59272127124058	7.67316257851900
H	11.24634868435678	10.97121047463532	9.24227470215726

155

[Cr(L^{MIC})$_2$]$^{2+}$_t_PBE0.xyz

Cr	3.67745827514905	12.48548592274240	12.00554289142522
N	3.31327189107698	11.32586956312788	7.87411015101820
N	5.64998558512251	12.43534908507272	12.00328241573701
N	4.80203567007059	11.45580253736011	9.28762041401373
N	1.70647394249731	12.53200605078562	12.00766752350887
N	4.58028434189834	11.11554739380505	8.03743736268766
N	2.45855187432403	9.67811897463392	12.59107228608072
N	4.86211443668629	13.46079919975196	14.71876230623777
N	2.59522948844500	15.33129783900366	11.35001236652041
N	4.10769826402958	16.56906143484940	10.70925360325127
N	3.92999774509020	8.32569961030153	13.07840329904338
C	2.70186884492452	11.79815210778616	8.99510163973408
N	3.38776008259884	13.66064875641100	16.13910738551902
C	7.83714563403857	12.06665188043217	11.34950007355710
N	2.65266907602956	8.41209750793509	12.88658284244352
N	4.66222568041713	13.81091650255465	15.96997282397075
C	8.84342640683790	11.61423536684360	10.50597518309637
H	9.88302084001322	11.71252264269871	10.80752705987616
C	6.46578175026269	11.95702983801517	11.00743841065812
C	7.15450840608965	10.97144449207245	8.91748335476795
H	6.86976275519796	10.56825117216373	7.95005387048788
C	8.51254309316974	11.03517618048232	9.27437217585067
N	2.83505622897289	16.55896054876600	10.94609774129634
C	6.13958158668260	11.43511377583375	9.74792304714987
C	-0.50895570983445	11.87537071374086	12.12760841168220
C	3.69749700273397	11.89027499643950	9.97486057188111
C	6.49090968991492	12.87831339772521	12.99776965120289
C	-0.47156130059209	13.30729549129600	11.93265088359571
C	3.69324978877570	14.51025924280379	11.39457005066506
C	0.90475218687337	13.63755289950556	11.84962225315848
C	7.85666647083273	12.69797198054391	12.64914924335157
C	4.70510893918873	15.36996708514280	10.95033559072481
C	9.56195291391238	10.49930402127744	8.30374064158574
C	-1.54611946739144	10.96543280961088	12.24930773272795
H	-2.57707153844486	11.31135831227459	12.19865703182712
C	0.85151811871149	11.46849858278950	12.17912847947244
C	3.59295298162263	10.44916491624868	12.57034955064480
C	3.73424923330446	13.08017129849735	14.03578513260372
C	0.07374656788364	9.20818943546392	12.52274237828491
H	0.32608921070561	8.16822279649543	12.69747972011908
C	1.25214500496984	12.15759764816635	8.95454472102375
H	1.16637217593626	13.25210626041734	9.01414547873469
H	0.75213873385956	11.76367470835870	9.84155121188061
C	2.74930457229172	13.21853025188731	15.02074263254638
C	1.12730563222174	10.11725081130036	12.40937046273868
C	4.57375382653755	9.51539768834841	12.92168608443647
C	2.69349027949908	11.04533918747048	6.58123041328526
H	2.50010690141935	9.96578355992470	6.53092777773077
H	3.42825279852831	11.29440342225118	5.80982206524711
C	6.19646634362072	13.41452098242356	14.25480880761715
C	1.40639733271971	11.83783755434170	6.45579324717785
H	0.87959223052601	11.50524754125945	5.55443156256407
H	1.63563253055178	12.90376626228105	6.31411602254604
C	-1.26714462386839	9.59984119505647	12.43426269787496
C	8.88132387802125	13.09826996431201	13.49006087126742
H	9.91691767281668	12.94845990415079	13.18960724803754
C	0.55105722836996	11.63975206698542	7.69951107508210
H	0.32483935112165	10.56985090743913	7.81758643567432
H	-0.41313921188950	12.14803525918531	7.59039185674025
C	1.24939486539962	14.97196102260446	11.59513135562325
C	4.74605014073034	17.79609745465580	10.23884909832330
H	4.92304068128112	18.43619606427797	11.11302204160899
H	4.02892166643638	18.30357032450532	9.58675019284723
C	6.16076955064074	15.19514618566028	10.66323755208845
H	6.26437061771634	14.53659964865924	9.78882734871868
H	6.63808026556574	14.67427654535315	11.49566221222454
C	6.04990051017478	9.59032202542203	13.13413345953347
H	6.29203834543892	10.51042417673652	13.67359495637223
H	6.54678557590778	9.66659066090989	12.15718579453027
C	-1.46586392682559	14.27210108438060	11.82881243668041
H	-2.50975039855995	13.97848376114193	11.90286182951129
C	8.58389031597493	13.69454639734564	14.72817911304262
C	6.87661416802351	16.52181771981182	10.41292015209349
H	7.08585497353473	17.01603410675528	11.37306644524586
H	7.84981104805724	16.32349861456713	9.95089203252854
C	7.23808485229164	13.82756161180054	15.08846024206598
H	6.97292296346609	14.24414726821876	16.05389999215312
C	6.04673370129421	17.45188497601741	9.53904922394138
H	5.83560565190676	16.97906793944702	8.56918364430309
H	6.58451108178668	18.38269634749724	9.32687288771825
C	4.51376624209924	7.04391047686409	13.47004723111254
H	4.03526419465473	6.26774182838342	12.86491157920816
H	4.24340099801306	6.86953467450965	14.51946551432339
C	9.71993927527311	14.15105141164145	15.64007375721105
C	2.78857545274893	13.96729690835021	17.43572227516746
H	3.51671774440363	13.68620317500621	18.20238613280676
C	6.01972989499468	7.08615299159658	13.29214279736928
H	6.44870875928979	6.20164957839411	13.77613695417277
H	6.27410629939972	7.01729979900802	12.22474741865310
C	6.58445782100516	8.37143643977500	13.88224392862255
H	7.67883032365318	8.36948930574603	13.83356012247913
H	6.32038488537555	8.43523543130691	14.94824772342869
C	-1.11719006608026	15.61417568690277	11.63085157323622
C	0.24607830897107	15.93088217343551	11.50497939147486
H	0.54588988085648	16.95673035883723	11.31232613809663
C	-2.41427090410516	8.59722867025044	12.53298212318811
C	0.61544051501910	13.47024224917566	16.33055200529336
C	1.28479061317530	12.92692044496741	15.06890190200704
H	1.14835146789430	11.83794650088061	15.00395871716851
H	0.79828126338793	13.34853662277659	14.18719065257870
C	10.59361767224570	12.93640628684395	15.99469315029372
H	11.03928265837640	12.47296827065160	15.10665086802751
H	10.00994333537520	12.17045209252555	16.51966907562702
H	11.41601310764379	13.24354983594104	16.65212696194006
C	10.56549366878692	15.19609478218600	14.89437542674254
H	9.96379407221781	16.07263688380242	14.62423862098223
H	11.00534422368348	14.79139747939755	13.97516283990951
H	11.39142412245470	15.53686688816560	15.53049894812264
C	-2.15097011619954	16.73369475787392	11.53525146661774
C	-1.89588109597779	17.74093011453006	12.66862985223560
H	-1.98360907856982	17.26064189158912	13.65097614133069
H	-2.63128065248704	18.55327885386674	12.62323201617258
H	-0.89949835898813	18.19357725092145	12.60030686380809
C	-3.58019151788669	16.20424336261195	11.66412790828956
H	-3.82885827296820	15.49383535372084	10.86581559925775
H	-4.28899416632565	17.03665572718108	11.58948061378678
H	-3.75297231928473	15.71754596669896	12.63223026158886
C	9.20376293640432	14.77501500415512	16.93732906516744
H	8.62063000985633	14.06186372023856	17.53340031929414
H	8.58517595607651	15.66162708476029	16.74939057773987
H	10.05135798736740	15.09504424160288	17.55388275292442
H	2.64274602915508	15.05422724151878	17.48786790412700
H	0.43524398119325	14.54940872186049	16.21778244158529
H	-0.36914417137114	13.00377284829238	16.44473400648721
C	-1.91649509655684	7.17168897158142	12.77838876821668
H	-1.27942631529338	6.81124877161304	11.96106351091465
H	-2.77253299859673	6.49088928812695	12.84603247792381
H	-1.35903683560941	7.08747836439252	13.71974439285631
C	-3.20166635658858	8.62158730628424	11.21199452185313
H	-3.63000527913099	9.60967484240902	11.00598005353821
H	-4.03104850198387	7.90516881932900	11.25383136171334
H	-2.56005002906190	8.34687614197090	10.36565608901477
C	-3.33831744663491	8.99805620457695	13.69397037017560
H	-2.79790140577454	8.99623434772486	14.64851064304781
H	-4.16946940005075	8.28727319300309	13.77639863948528
H	-3.77411690375999	9.99399897041195	13.55302914352341
C	-2.01065795158841	17.43791476548287	10.17601345951774
H	-1.01974242981416	17.88780508241193	10.04361844720393
H	-2.74996321597187	18.24355149662057	10.09090124108874
H	-2.17894071192428	16.73772623122503	9.34863265534815
C	1.46942877410852	13.23104217226170	17.56797417882988
H	1.65343521486021	12.15575716583514	17.70500307877314
H	0.96360649850437	13.58348806941932	18.47380151271651
C	9.29448879826707	9.00649495149484	8.05363143547772
H	9.36502515748960	8.43218467345036	8.98558156081481
H	10.03488491100069	8.60271931856955	7.35250399876457
H	8.30200389183020	8.83162885207950	7.62170900806082
C	9.46364041898892	11.27323544290915	6.97922757716708
H	8.48292728283156	11.15707836622137	6.50284829730604
H	10.21759789243268	10.90693935853690	6.27196268107370
H	9.64000328618075	12.34474228322541	7.13408295552331
C	10.98137053916999	10.65503782829780	8.85291171862762
H	11.24497897762344	11.70677816666585	9.02052294174771
H	11.70086370985111	10.25331867076280	8.13055038039582
H	11.12260224907592	10.10497401974737	9.79173404482171

155

[Cr(L^{MIC})$_2$]_t_PBE0.xyz

Cr	3.68670645430994	12.49198487393432	12.00005673418788
N	3.33620682493816	11.45074615181250	7.85941642238277
N	5.70420205630733	12.45453744905797	12.00625866144735

N 4.86876088930125 11.81897750121531 9.19283654003968
N 1.66751858680841 12.53006567671805 11.99503290594683
N 4.63486438811502 11.54044536986568 7.91124938543091
N 2.44108754757903 9.77380599928345 12.90549012326859
N 4.87763587668385 13.12166914956420 14.81897711183700
N 2.55216483472940 15.21661145173666 10.98388202257737
N 4.10591379418381 16.50863717394504 10.56401894673725
N 3.92248264683383 8.35307762375751 13.11760437769098
C 2.72961343184322 11.65324881325130 9.05505065299343
N 3.35923473916366 13.56939577722461 16.14173895813495
C 7.89982025206566 12.25301816802328 11.31259385537033
N 2.63308127769016 8.49679616355277 13.22572212308623
N 4.65125879133682 13.41988746917297 16.09488665328740
C 8.92017121972956 12.05392226135710 10.38044201204977
H 9.95618738832797 12.10061766746307 10.70832028627349
C 6.52507154221138 12.21085069930065 10.94673161289416
C 7.24002142586859 11.74336612692984 8.68964751743876
H 6.95561882770623 11.53301208766257 7.66237826322383
C 8.59994939239002 11.80193086657551 9.04713001673904
N 2.80755804197165 16.43071415327253 10.50062372073987
C 6.20630141646723 11.93243112132154 9.60349551287569
C -0.54182276632995 11.92673586860763 12.31414866218077
C 3.74280574635497 11.92833596510989 9.99095501345793
C 6.52428936917373 12.65003157315110 13.07827191079398
C -0.51588005670737 13.25537227439044 11.76752156992590
C 3.66353451970495 14.48028543166854 11.35619786502818
C 0.86536261878095 13.56175447713038 11.60807783057014
C 7.90187595127954 12.52993644361361 12.72268891442168
C 4.69217605082300 15.39704389765600 11.07388784832740
C 9.65681008576674 11.58153732668588 7.96293691237094
C -1.57122846519552 11.07959735056945 12.71515182156588
H -2.60417106147824 11.41814265051014 12.63525213348833
C 0.82861811953813 11.54034527963709 12.41095758936657
C 3.58449396951385 10.48244062318058 12.58242593252775
C 3.75333410583591 13.05844655493456 14.01579756378471
C 0.06958606129200 9.41691824813733 13.28038499257272
H 0.33776261447804 8.42973081512416 13.63986202075120
C 1.24305310607163 11.56135932033391 9.15609861634506
H 0.82823945770939 12.56967121771314 9.29309864502557
H 0.97679832993404 11.00852015112567 10.06169185327324
C 2.74926485512502 13.38618659612632 14.94437142873018
C 1.11794288328728 10.24699024827886 12.87788253416423
C 4.56538851899189 9.48709083256318 12.74362038195185
C 2.70663207254647 11.11451612726539 6.59517438735373
H 2.85540879570856 10.04067070346881 6.41479994212032
H 3.23790119715752 11.66133243381957 5.80900423135453
C 6.21221704063232 12.93846014863944 14.41751591775932
C 1.22839297268297 11.45325496769888 6.64692083055675
H 0.74113990433198 11.03721471736004 5.75663695121783
H 1.09520284907763 12.54420702191616 6.60676554335948
C -1.27609286157821 9.80886290354102 13.21906281657495
C 8.91624599435658 12.66399183553519 13.66722058138367
H 9.95498279719767 12.55525304693387 13.35536970989291
C 0.61643742843965 10.90549933504034 7.92934282809733
H 0.76793929394473 9.81562935020305 7.96568854376342
H -0.46728273476888 11.07041906826687 7.94415386717389
C 1.20720164445489 14.83067269658764 11.10208479062237
C 4.75532410001556 17.73834702895916 10.14609460622513
H 4.59908279821781 18.48988070918559 10.93255945648186
H 4.24327113332338 18.08881770567285 9.24373207159578
C 6.17657990305854 15.34682592481500 11.22191320722671
H 6.59304168505370 14.67674182359078 10.45704520604245
H 6.42931666209274 14.89004403549878 12.18309059010999
C 6.04159155086793 9.46206185651162 12.52120737817411
H 6.49646985631603 10.34015045703293 12.98527950945193
H 6.23674409693211 9.56111619667880 11.44378207269111
C -1.51924871784502 14.15787825432415 11.41054431485421
H -2.56081202054494 13.87561542773901 11.54611809351125
C 8.59879136265683 12.92804092950909 15.00297919340061
C 6.81760685306974 16.72594229129021 11.10154222276893
H 6.65100422091462 17.30132859606631 12.02517030000156
H 7.90280169945637 16.61310469117523 10.99467228575988
C 7.24589409198006 13.06641654908534 15.34726679525899
H 6.95904725712966 13.28663816786322 16.36940552867625
C 6.23588213065577 17.49269940312569 9.92143251056776
H 6.38145052406600 16.91644905437808 8.99616144992003
H 6.73504457770846 18.45911880466776 9.78050644243656
C 4.51455222823776 7.06040570213077 13.40985736284419
H 3.81121187771301 6.29556124557035 13.06501334382843
H 4.60895708848925 6.96257591946576 14.50050351653353
C 9.71694033526546 13.04736439000885 16.04190486321042
C 2.73993464842646 13.94306698168633 17.40098126534517
H 3.24187182321018 13.37808041965399 18.19344564857032
C 5.87260415304402 6.95721856207417 12.74120184728405
H 6.36805678493905 6.04185475942492 13.08727304156834
H 5.74361466688137 6.86256216954055 11.65298723174291
C 6.69905749685591 8.19517281555757 13.06202087805369
H 7.70960445215417 8.10682889988299 12.64619866358199
H 6.81776262383996 8.27520891628404 14.15343267173283
C -1.17603944476965 15.40161747046630 10.88191455223168
C 0.18966995326390 15.71171509026823 10.74409504657805
H 0.49059754802370 16.67906070106449 10.35144134062689
C -2.41034511765792 8.89298375147854 13.68711463329794
C 0.66764664408681 14.23847233175709 16.05468144014336
C 1.26988341016260 13.54617607091374 14.83578881532645
H 0.80896854303813 12.55771905215314 14.70237850071726
H 1.03466014469536 14.10586748125000 13.92599297822693
C 10.47815851428457 11.71431243017718 16.11670271567645
H 10.92327596638287 11.44926095343365 15.15041566298636
H 9.80392950456606 10.89885820376397 16.40704679734380
H 11.28821321200318 11.77016343488526 16.85656804410511
C 10.68538611957004 14.16558604897554 15.62885646131239
H 10.16482413264962 15.12973108536996 15.57300006127732
H 11.13341091675154 13.97256896427038 14.64712794338983
H 11.50291569843358 14.25950715528675 16.35655311917577
C -2.21565199043434 16.43652621603532 10.44742047833006
C -2.01468612531130 17.73603503310743 11.24183417992251
H -2.13254091982064 17.55592475587881 12.31765550335043
H -2.75093318589422 18.49261158119751 10.93833305713269
H -1.01592970222169 18.15892976936498 11.08291676301475
C -3.64500873837771 15.94715333822268 10.68751752394574
H -3.86287924725905 15.03278384324834 10.12149535276803
H -4.36103250763206 16.71423087180660 10.36663921895963
H -3.82976289649817 15.74423890760706 11.74985145926857
C 9.18013675281092 13.37167931104212 17.43758221165421
H 8.50332386696049 13.59031345496113 17.80481675781097
H 8.64012341829678 14.32657792175746 17.45391264563868
H 10.01374454795284 13.44858266801794 18.14705747597115
H 2.93713900028102 15.01016487536379 17.57397914588183
H 0.86835612934407 15.32006988885806 16.01155490432225
H -0.42229396386481 14.12229661580275 16.03505123765616
C -1.89861324741659 7.52801748606985 14.15186091949051
H -1.38023788459301 6.99354125847884 13.34597419363035
H -2.74203410696673 6.90530274397034 14.47598273821366
H -1.20870864128797 7.61921341971478 14.99991465398828
C -3.39931239425539 8.66665448799296 12.53392462304511
H -3.83586561728768 9.60865846223608 12.18229938392871
H -4.22386497940186 8.01541174483518 12.85413927100101
H -2.89970257274849 8.19186599714786 11.68029472082716
C -3.14168831396297 9.55983013019800 14.86255133310955
H -2.45588833548695 9.72148638839403 15.70360046823309
H -3.97158151836515 8.93082071100497 15.21269171342329
H -3.55508146007809 10.53482882403609 14.57854699073873
C -2.04836018774649 16.72396975225081 8.94751371564041
H -1.04717020199659 17.10855087080118 8.72001084976265
H -2.78047066499490 17.47047397417716 8.61079845534557
H -2.19543899431391 15.81001936869044 8.35840123974233
C 1.24769673466572 13.67289700619415 17.34422209068685
H 1.06325603415049 12.58974668642793 17.39231656352909
H 0.77649182253168 14.11833826455679 18.22889228846919
C 9.47216203908858 10.18858350328259 7.34136017343334
H 9.58117668221935 9.40587407724237 8.10251266571940
H 10.22097858925634 10.01129278121203 6.55743882271513
H 8.48025844662275 10.07631938092191 6.88831414790740
C 9.49680175010934 12.65238411560504 6.87293544110946
H 8.50587274828291 12.60750607564724 6.40604484608621
H 10.24733711423211 12.51819483264653 6.08208427587190
H 9.62018035740027 13.65727834133252 7.29585548324975
C 11.07755294673064 11.67215335869803 8.52226966442034
H 11.28040961679907 12.65872562194550 8.95755117613634
H 11.80630587843920 11.50761561291527 7.71856699554893
H 11.25842017770898 10.91366645661209 9.29423610559119
155

[Cr(L[MIC])$_2$]$^{2+}$_t_r2SCAN3c.xyz
Cr 3.67626406637430 12.48924171527703 11.98881893774395
N 3.36881985115031 11.28360207452356 7.84480067634700
N 5.65636542310861 12.43191105366881 11.99394708208315
N 4.82942354664021 11.33646515388155 9.31476280203576
N 1.69853899799408 12.53232224360154 11.98452521747534
N 4.62356733010206 10.96837647563103 8.04962921603004
N 2.43742026508961 9.64843804534785 12.42560534388359
N 4.88477117286733 13.57663139307596 14.66637076997828
N 2.56600853132621 15.37121046700475 11.47833357031890
N 4.05749673714273 16.61926889475955 10.76049555766216
N 3.90348975595832 8.30117099382620 13.00469815152509
C 2.76850529300647 11.84347081047069 8.93523899512222
N 3.42718673495971 13.69969640372651 16.13528905394151
C 7.84874371595952 12.00513846095791 11.37492095767133
N 2.63029764175633 8.35726886996069 12.69941921662465
N 4.69634429904056 13.95135378494593 15.93246914656129
C 8.85553040988509 11.47286244942911 10.58212266953341
H 9.89098178474345 11.58497109759975 10.88343814092370
C 6.47989241923428 11.87531237997140 11.03274808869115
C 7.17248591915827 10.72805148235178 9.03130404484593
H 6.90080788242005 10.25960703708141 8.09387235177541
C 8.52433322342093 10.79311827704514 9.40493867391168

N	2.79194974488596	16.62945395356948	11.09809419514279
C	6.15943728333519	11.27968221813515	9.80794543321570
C	-0.52207253921815	11.86772835151376	12.04958754749386
C	3.73474803521600	11.88482888897778	9.94711660126012
C	6.50580016056372	12.94632422822826	12.95820871751678
C	-0.48573693862379	13.30867609833147	11.98102590294532
C	3.65840852703740	14.53451945494622	11.40091832822928
C	0.88753752798417	13.65200514788469	11.91908663073626
C	7.86901892787676	12.73787061992126	12.61756213560355
C	4.64532399463252	15.39652564708446	10.90873730516247
C	9.57257741303709	10.14505023778633	8.50155307870586
C	-1.56040982775248	10.95523641067086	12.09177049333224
H	-2.58772673629342	11.30622906376006	12.07326807981791
C	0.83602602574779	11.45215963091459	12.06861008693788
C	3.55967368790079	10.43965130897790	12.53459191116867
C	3.76288755729921	13.08729902716107	14.03085979853494
C	0.05288373028195	9.17447486995647	12.22787848410734
H	0.29304749822783	8.12516152561736	12.32636300088336
C	1.36124430926847	12.34100097817945	8.83106833854146
H	1.38640564224443	13.44014633506087	8.81312407774262
H	0.80089465743500	12.06005053521776	9.72296857134750
C	2.80019220750515	13.17377061386999	15.04298825085865
C	1.10754687219048	10.08975797874208	12.20625247419648
C	4.52434917612638	9.51528126156711	12.94640256726897
C	2.75672443860139	10.99586464533026	6.53611673573994
H	2.47055195925238	9.93672214388601	6.52854161718872
H	3.52551870594769	11.15218188178335	5.77456319756527
C	6.21649116905573	13.55847408296776	14.17849198848219
C	1.54090575414677	11.89057841481434	6.35001386136360
H	1.00806972340882	11.56291769891001	5.45229734705265
H	1.86130474605441	12.92623264731409	6.17721024868360
C	-1.28302757114713	9.57988743971494	12.15672523039907
C	8.89550759904455	13.20974123192649	13.41488304893111
H	9.92726125386321	13.04113617343201	13.12118606764355
C	0.64433392919141	11.81122754743438	7.58307477778007
H	0.34231847019516	10.76812107931911	7.74748610770232
H	-0.27378505147374	12.38521103273075	7.42794397855020
C	1.22492925305747	15.00064847245715	11.76018976947076
C	4.69220812412076	17.86705703249110	10.30096145240202
H	4.94949099213786	18.45596300134887	11.19014957311094
H	3.94558778001206	18.41981681488528	9.72428484390839
C	6.06442589550720	15.20077079956468	10.47747188023278
H	6.06323394463198	14.62232815030963	9.54228795831251
H	6.59638199328394	14.59726161346471	11.21319893898575
C	5.96388848675573	9.63289026862021	13.33118465210980
H	6.10448436861907	10.54796605640693	13.91413346783008
H	6.57371451484312	9.74617158254928	12.42562300148662
C	-1.48317651943494	14.27358784421250	11.97340760333883
H	-2.52247757056440	13.96982408567050	12.02861375440737
C	8.59982616302414	13.90876325849389	14.59697125847933
C	6.79858365869458	16.53067922609129	10.26821450407390
H	7.06497973965614	16.96145657126315	11.24273801173601
H	7.73653676605305	16.34208404018844	9.73798380925752
C	7.25924543242739	14.05204840118039	14.96621230936054
H	7.00618635645098	14.53532508873611	15.89946652821125
C	5.93471927044004	17.52394008229867	9.49434808397227
H	5.64594773648930	17.10128552187137	8.52323677887250
H	6.48064317045777	18.45078872152222	9.29421154935014
C	4.49057281631089	7.01115772377881	13.41049453724034
H	4.11881051065981	6.24676738998649	12.72225103834946
H	4.11406204438929	6.78340763738250	14.41523720245709
C	9.73944908797971	14.46739452798620	15.44671303812750
C	2.82960047159663	14.01199265020515	17.44504244453069
H	3.59127504192359	13.81948103163614	18.20542714992763
C	6.00792635895946	7.12323654052577	13.40634622927338
H	6.41602453720184	6.23710265916333	13.90161590057840
H	6.38244296338812	7.11960719675325	12.37448304693932
C	6.44219934061440	8.40546621825415	14.11435485451004
H	7.53133321147663	8.44136772267012	14.20875891328790
H	6.03146048283698	8.42200343207784	15.13280274007623
C	-1.13751447337274	15.62690633749842	11.89740299079856
C	0.22034948737945	15.96208411059647	11.77487964088222
H	0.50501710029679	17.00035638171041	11.66106286208209
C	-2.43691806131827	8.57827253212255	12.17406087047300
C	0.68129506666401	13.29853505510974	16.39828970660336
C	1.37111621815492	12.74341544051363	15.14612167060430
H	1.34471963458043	11.64427479359707	15.15619534526671
H	0.82370579499136	13.05703481188633	14.25714466393324
C	10.66114176119115	13.31086952174499	15.88367192929586
H	11.10674634247167	12.79289136145121	15.02894537639495
H	10.11040111283444	12.57597449358838	16.48012364599942
H	11.48071620939108	13.70225471630546	16.49507900582378
C	10.54473631224565	15.47255839573609	14.59872692471288
H	9.91024538808439	16.30079281414243	14.26529696661152
H	10.98572223523356	15.00261814481127	13.71408941100655
H	11.36328943530902	15.88840269447912	15.19522230401437
C	-2.17672206311402	16.74781517780413	11.91629971383849
C	-1.88683838766757	17.68173721321301	13.10761809153255
H	-1.93552908632131	17.13362836440599	14.05461820238675
H	-2.63114178204352	18.48409283003184	13.14012135109439
H	-0.89990255973127	18.14890049271667	13.03268745376802
C	-3.60454488146569	16.20846328382295	12.06015725607756
H	-3.88872371505273	15.57009072059235	11.21640581131729
H	-4.30643246310917	17.04706109249053	12.08385084782045
H	-3.73670544484019	15.64495609668234	12.99036409806520
C	9.23767632585232	15.18618155068037	16.70379895998854
H	8.69108989639930	14.51041188145322	17.37085870457132
H	8.59415975790532	16.03889501894566	16.46020571694434
H	10.09410387116649	15.57320736955129	17.26363076386597
H	2.59088410853321	15.08281008553883	17.45537861517593
H	0.42792103280275	14.35543481910071	16.23998010480539
H	-0.26216211447519	12.76678418835989	16.55223826908373
C	-1.95208209597554	7.12566563973759	12.23476929776461
H	-1.33323924133127	6.86416611093448	11.36920726801477
H	-2.81701394784005	6.45596081127866	12.23225469764214
H	-1.38494945180901	6.92079711470423	13.14951501285816
C	-3.27454585397494	8.75976113087611	10.89245758392627
H	-3.70461714293488	9.76352141090926	10.82187806841529
H	-4.10280564350977	8.04375761881596	10.88496115030875
H	-2.66479952941034	8.58574583438347	9.99947399050319
C	-3.31933429921460	8.84964525809961	13.40905148518876
H	-2.74221705780887	8.73772490341846	14.33300229218347
H	-4.14948855959556	8.13621227607659	13.43688035037330
H	-3.74909706254006	9.85594306778771	13.39499188842809
C	-2.08704477449923	17.54643261310729	10.60082201002897
H	-1.10836096340442	18.01934802245241	10.47286055544149
H	-2.84130689252243	18.34000031790724	10.59731044514556
H	-2.26988144616269	16.89850716787964	9.73710211189702
C	1.57539578389064	13.17179395291389	17.62933253499034
H	1.84964550658831	12.12194569607826	17.79558805947153
H	1.05952582362019	13.51819935124668	18.52990602113408
C	9.27436109192469	8.63656532043444	8.39043466219187
H	9.31146993872383	8.15814193477556	9.37511264907118
H	10.02143466946473	8.15610957101891	7.75014509665223
H	8.28941095107471	8.44334510211807	7.95362505190933
C	9.50912934242236	10.78750336223803	7.10158039082015
H	8.53527656598507	10.64234704346190	6.62365531067913
H	10.26638009604904	10.33597681386274	6.45240033208587
H	9.70543058959550	11.86338583812025	7.15718168616859
C	10.99399061778613	10.31931163420275	9.04815523115967
H	11.28129186180392	11.37463580082460	9.11113266910635
H	11.70270928351861	9.82944251943110	8.37416516031321
H	11.11264229281144	9.86098429639619	10.03617092366071

155

[Cr(L^MIC)₂]_t_r2SCAN3c.xyz

Cr	3.68669530374565	12.47761362765367	12.01116303273460
N	3.35542958653439	11.45780981275357	7.83619717062443
N	5.71741695509312	12.43629770546365	12.01550132050562
N	4.89460456682894	11.86025317631334	9.17737267381755
N	1.65424701217372	12.52188099279102	11.99676595125523
N	4.66597317120956	11.58988026605987	7.87200947708387
N	2.40272698773851	9.76056247096273	12.93182074213575
N	4.90320307134070	13.05335316970368	14.84987016112196
N	2.53599738364254	15.19568555651430	10.93285952426287
N	4.10155650382757	16.51090687039112	10.54947115101577
N	3.87524329427576	8.29874672087940	13.08000053010121
C	2.75491921929271	11.62021131483771	9.04565665784719
N	3.37915815518644	13.53770202513024	16.18024834546350
C	7.91943529482174	12.26001629979919	11.31718627381220
N	2.57543876383751	8.45302550691775	13.22730173938513
N	4.68219267220223	13.34756344717018	16.14996904808591
C	8.94387563167075	12.09621233555381	10.38638026495862
H	9.97517253567088	12.13675002614998	10.72111423069292
C	6.54581432389829	12.22387636254449	10.94409226753649
C	7.27339705639413	11.83112565675544	8.67758179226990
H	7.00606687316471	11.65014627746958	7.64409863904739
C	8.62855658854706	11.88804601744534	9.04596144129311
N	2.79268173172518	16.42336482381581	10.42800999144141
C	6.23499182752199	11.98377091800194	9.59382031734637
C	-0.56282831695237	11.93850996544252	12.33757519905398
C	3.76056941914082	11.91895243413360	9.98191081518947
C	6.54508327972745	12.60450222296579	13.09693234692260
C	-0.53287752904014	13.24754547601265	11.75338368528552
C	3.65072960867757	14.48235482347532	11.36312265964983
C	0.84797831694319	13.55002797580360	11.58039889524514
C	7.92148224736153	12.49790901158869	12.73111496203292
C	4.67475719097439	15.41155680977773	11.10905855250427
C	9.69294553211522	11.70731699104854	7.95998510805321
C	-1.59744348915320	11.11534564782008	12.76468012193626
H	-2.62455098802249	11.46149260237391	12.67923975919618
C	0.80490100139185	11.54281688587223	12.44272674211257
C	3.55958994429760	10.45126981756167	12.58508373990998
C	3.77111878556428	13.03692097410926	14.04138063966771
C	0.02856362859523	9.44828502048110	13.36302319465961
H	0.27842725566437	8.46803964804586	13.74330026128146
C	1.27401486672505	11.45193161614187	9.16248561810572

H	0.80392966541937	12.43433697414685	9.30209782473060
H	1.04724727576259	10.88109733961087	10.06665595156433
C	2.77644190072036	13.38881198687269	14.97019279507664
C	1.08268868806824	10.25583287934673	12.92645483044372
C	4.52359577774010	9.43484013925347	12.70810574772546
C	2.72238821870475	11.11187134469958	6.56250368127690
H	2.91644584776057	10.04940904310840	6.36216090445129
H	3.20909005278994	11.69990992530179	5.77782762494953
C	6.23885179358160	12.86287080675006	14.44148286407562
C	1.22642502058622	11.37624038389581	6.64974754499618
H	0.74632533974162	10.95220595749831	5.76103773862710
H	1.03678137083865	12.45790019208504	6.64159448836035
C	-1.30952088776248	9.85810693078654	13.30207518490584
C	8.93987193715543	12.61757099703161	13.66899359154288
H	9.97469896493429	12.52714864045688	13.34791493603591
C	0.67160223748581	10.76425488858401	7.93393397361085
H	0.90298578194246	9.68975974633857	7.95247000490056
H	-0.41873623635197	10.85770304449732	7.96784505331412
C	1.18693890931115	14.80354825659676	11.04120548798657
C	4.76157183786672	17.74732023600360	10.12664295637875
H	4.55831350340446	18.51878488319716	10.88175079473855
H	4.30204784042641	18.06461426874733	9.18507806448619
C	6.14986681658706	15.39133001981807	11.34973160779266
H	6.62957648294224	14.71963871075688	10.62580369409337
H	6.34950713032347	14.96155572696237	12.33498514878282
C	5.99581802776301	9.39387875085638	12.44967360952274
H	6.47520281742189	10.25413228341053	12.92005657702600
H	6.16817017179740	9.50669903294440	11.37027315541889
C	-1.53776263932397	14.13360822808491	11.36926165038795
H	-2.57566626933857	13.85364988819781	11.51631488949748
C	8.62605283619697	12.85034199013111	15.01014693472485
C	6.77225079839668	16.78606581792291	11.24006199471083
H	6.52650689781573	17.38170098123605	12.13075494120672
H	7.86262777670702	16.69136006995452	11.20946934154336
C	7.27807216918383	12.97301895947090	15.36974362850599
H	7.00696515038948	13.17111110672440	16.39676242598517
C	6.25799803782874	17.50513111894547	9.99510600023348
H	6.46067582222708	16.89503575236258	9.10478554979091
H	6.75512022305904	18.47078329960689	9.85235125889854
C	4.45840901142682	6.98095265292527	13.33193345037594
H	3.73893479841405	6.23098453124729	12.98901843462832
H	4.58785903009362	6.86061200016416	14.41623421792073
C	9.75211267648072	12.97603638514343	16.04147532555885
C	2.75846270800201	13.92950468566291	17.44676654569665
H	3.21289678885329	13.32873690699231	18.24108070580908
C	5.79850860091043	6.88132272688581	12.61859524401165
H	6.29087875787644	5.95298464137227	12.92829270331548
H	5.63833947391316	6.82338017270289	11.53361188093764
C	6.64805316785361	8.10359658476508	12.95711848550302
H	7.64914651698017	8.01278943368595	12.52313726617774
H	6.77776355295269	8.15957611708071	14.04712313790050
C	-1.19471079002751	15.35696924030626	10.79863762373597
C	0.16752913303695	15.67031488013413	10.65259429756040
H	0.45468244685790	16.62630799911111	10.23302766515261
C	-2.45142866345917	8.97033557532956	13.80790670348967
C	0.73147402883230	14.35515593353815	16.06033978338798
C	1.30592238229355	13.62155999502551	14.84503645468698
H	0.79112680950340	12.66001274707175	14.71883831303763
H	1.10934224266126	14.18843247971764	13.93145547492683
C	10.57224894575658	11.67175253957950	16.06658320191820
H	11.02442044006143	11.46118845334396	15.09249570901890
H	9.93390344712292	10.82180738783643	16.33131421660335
H	11.37988365761541	11.74172178753826	16.80525941927009
C	10.67327444268874	14.14809899179852	15.65288865539716
H	10.10758029690797	15.08546459925925	15.61687271128757
H	11.12889852072254	13.99368677119874	14.66995767317709
H	11.48235743333978	14.25970594761032	16.38503895881817
C	-2.23694021353989	16.37671745949869	10.33063772779709
C	-2.04532461217049	17.70348806980188	11.09040842952590
H	-2.15264900081365	17.54766951572630	12.16935554511594
H	-2.79423059965439	18.43823581004551	10.77077876580285
H	-1.05522914933751	18.13303381452349	10.91049517994206
C	-3.67087290709564	15.89254464195604	10.57583792721719
H	-3.88299655909506	14.96345325275314	10.03545362809928
H	-4.37877071379960	16.65113819090213	10.22476216728843
H	-3.86286758652461	15.72506783681217	11.64133292335468
C	9.22674677020041	13.23397253567850	17.45877286882986
H	8.58412899167620	12.41799790257551	17.80701598223521
H	8.66019126305874	14.16969107489004	17.51738994109517
H	10.07169793526633	13.31205167763537	18.15155203413670
H	3.00321404312089	14.98373827298583	17.63443859860822
H	1.01288401474342	15.41729594774365	16.02699680903351
H	-0.36193705913679	14.31217224360739	16.02279309308551
C	-1.95992980276734	7.60586919941089	14.30573400916532
H	-1.46261837637310	7.04064254636088	13.50990958365103
H	-2.81446942880560	7.01462625426185	14.65256468815907
H	-1.26346754735911	7.70713089374006	15.14508771655796
C	-3.46321984949096	8.72621597442427	12.67231813324406
H	-3.90065695273545	9.66179629818673	12.31125553458490
H	-4.28230214948189	8.08698069856489	13.02331668531435
H	-2.97819417449996	8.23207308681227	11.82354367259932
C	-3.16213989658956	9.67906929491238	14.97727806910650
H	-2.46029459865081	9.85932580613816	15.79889407187918
H	-3.98746069463695	9.06339656768926	15.35532820033435
H	-3.57371974912785	10.64547681065992	14.66953366314295
C	-2.06709428021823	16.62733612588165	8.81993700463487
H	-1.07333172106859	17.02207302547158	8.58684921179522
H	-2.81036290163140	17.35194799688706	8.46587506921861
H	-2.19815227989999	15.69564025308538	8.25873499823938
C	1.25164013640246	13.73700256281310	17.35614090644781
H	1.00919307170471	12.66615916702190	17.38005816475334
H	0.78946740731315	14.19759862646373	18.23610258436366
C	9.52644843403750	10.32659707214930	7.29691452597833
H	9.63044355961631	9.52850562484543	8.04008848713228
H	10.28849580931604	10.17931970909637	6.52188746652274
H	8.54405166310479	10.22101916172051	6.82663286014719
C	9.53153837849178	12.80670951981477	6.89254483786992
H	8.54891884256114	12.76127381185517	6.41297667395912
H	10.29369750041986	12.69729541294265	6.11144456147093
H	9.63937252680392	13.79892350403532	7.34423665778405
C	11.11583502121992	11.79603755113684	8.52386457260881
H	11.30793318947956	12.77100357963351	8.98515171694223
H	11.83989259595484	11.66298323243494	7.71272694224696
H	11.30453543788298	11.01621731145648	9.26983690372673

155

[Cr(L^{MIC})$_2$]$^+$_wB97XV.xyz

Cr	3.67865893816803	12.47003903327972	11.95383155782779
N	3.41232008808503	11.39371910104920	7.76284901732925
N	5.69421546302679	12.43178671997256	11.98616179862866
N	4.89568466624583	11.60829183816400	9.17890100386531
N	1.66209715053738	12.51317582394415	11.91475690113203
N	4.69948246798581	11.31076488575356	7.91231194965266
N	2.41966081243609	9.67387154421519	12.66552851185368
N	4.83282601435467	13.26749938700893	14.77361791831910
N	2.56791395251721	15.29404687519805	11.10869791873484
N	4.10027800653170	16.56028186370468	10.56378701145642
N	3.88814920787271	8.25781415213910	12.96536144567052
C	2.76508756798253	11.73643086011547	8.91441289262185
N	3.32146680531641	13.50253863916096	16.15591154383428
C	7.90486528587060	12.15438046027015	11.34058219479703
N	2.59801934259121	8.39981003003648	12.93869387824783
N	4.61316144441905	13.55091874907339	16.03888595862763
C	8.94220095459442	11.78937972450597	10.47267021549221
H	9.97325139775158	11.86338615160169	10.81919821200965
C	6.53892036178663	12.07006671421508	10.96399704545090
C	7.28967685824064	11.27288947866198	8.80316470789851
H	7.02730434182236	10.93674671531675	7.80106715868209
C	8.64847729423593	11.32712685144487	9.19038942810841
N	2.80656127354164	16.50395479280624	10.65108537906416
C	6.25137750149705	11.64213898365691	9.65227543274399
C	-0.55993655961163	11.89308321337685	12.16961996537201
C	3.75521556912453	11.89434281576833	9.89399426336063
C	6.51398716460316	12.76634805645803	13.03996201473790
C	-0.52133878082371	13.29226454826452	11.79836572153548
C	3.68543371792263	14.52174641255143	11.33092379659019
C	0.85783420829061	13.59993866715114	11.65973354336582
C	7.89177814754744	12.63772745937975	12.70490173695381
C	4.70947105680032	15.40562328992551	10.96188207162575
C	9.72672223414957	10.87341079515685	8.19274929529985
C	-1.60467483810750	11.02081884748226	12.48406041380840
H	-2.63442570975235	11.38303023754670	12.44079220215167
C	0.80278247888239	11.48413712513179	12.22177505814122
C	3.57604495379329	10.40233902317160	12.49989065710646
C	3.70281192968997	13.02581799830315	14.02449556340586
C	0.01525206056989	9.29394904147206	12.90331780031931
H	0.26891280096216	8.27747397549236	13.19229368970936
C	1.27002037882689	11.91801365382568	8.90660192842824
H	1.04761845680276	12.99186576539408	9.00829123224870
H	0.83796933405214	11.43213014990131	9.78790138974677
C	2.69395345725816	13.19581709797382	14.98340616854228
C	1.06837383652315	10.15315810177731	12.58729259831224
C	4.55490940762474	9.42292334072107	12.71839159446233
C	2.81317480936051	11.11095040537926	6.45267667553652
H	2.74999216320555	10.01913301396716	6.34085537336102
H	3.50177734993563	11.49558454280452	5.69199102296294
C	6.19961058908013	13.19714835337035	14.34031339951182
C	1.43078606605811	11.74963722275091	6.38563761473076
H	0.93363169271646	11.41291984509706	5.46641061106406
H	1.52847726631469	12.84491415977678	6.31788332053975
C	-1.33199698192818	9.70280929849894	12.86503672351733
C	8.90517183451156	12.96724520076075	13.60817525213134
H	9.94867132293474	12.86535300064515	13.30148761602994
C	0.62303166394971	11.36740378713149	7.62825891155433
H	0.55062153142504	10.26911089853701	7.68851426529169
H	-0.40485607132623	11.74549579051244	7.55305428788324
C	1.19620553706228	14.92114617387154	11.30637539008135

C	4.73934009374368	17.79598184652382	10.09434935920653
H	4.73867485249191	18.51037205157490	10.92991271694768
H	4.11058724582063	18.20129640954893	9.29348350255731
C	6.20939732833839	15.29677530928251	10.89801188519582
H	6.47942276187592	14.62521400628013	10.06815310050207
H	6.58021388882529	14.82115981846906	11.81175693806336
C	6.06041626778930	9.42885176339910	12.70386063482099
H	6.42649413153623	10.31925922531675	13.22684408993619
H	6.40473595612914	9.51752679406426	11.66173270480047
C	-1.52293465249454	14.25668958559890	11.61799708457471
H	-2.56639008324780	13.96746182709334	11.74558820897274
C	8.58441160714086	13.43500654415969	14.88640090875742
C	6.88059310984060	16.66314072355397	10.70662178578817
H	6.87534757616386	17.21901437519895	11.65862961606576
H	7.93397837563051	16.51292997046990	10.43638872765617
C	7.22165360973942	13.53034173391153	15.22994771521710
H	6.93144081891707	13.86755690171629	16.22146312002772
C	6.16029537170826	17.48823572456451	9.63780106156378
H	6.13541684532126	16.93551365895769	8.68532060335853
H	6.67886358077458	18.43690361003959	9.44611021534448
C	4.46368091160160	6.94203134427412	13.27225599824622
H	3.84669510191828	6.19146259690917	12.76522810335076
H	4.37653950848920	6.78507209212662	14.35675454713274
C	9.70636310585245	13.83649930696924	15.85963201038080
C	2.70119152144357	13.78527699488437	17.45623413782600
H	3.35661364981575	13.36256809041674	18.22597625073735
C	5.92154602443990	6.91106633287635	12.82879677657401
H	6.38408697958160	5.99309206048231	13.21468802358675
H	5.97673261217259	6.86372469384688	11.72953147640302
C	6.64661570164871	8.15986370674787	13.33608827198455
H	7.71894910822009	8.10503466125360	13.10741067398371
H	6.56019375685053	8.21101282312419	14.43399155976503
C	-1.17783241877860	15.56746400166158	11.29142046053599
C	0.19409541994287	15.87006466101366	11.13751091150388
H	0.49695009621546	16.88294446133832	10.87706117111792
C	-2.48601352032293	8.75896438711417	13.24532133767834
C	0.53487782534168	13.62098026042829	16.22248904520495
C	1.19533224674391	13.05511542107625	14.95836998490212
H	0.94527221866599	11.98779055645349	14.85266271299322
H	0.79496988477576	13.55526848892812	14.07072228507915
C	10.64042047498845	12.63253587922683	16.09522472379239
H	11.10119116502133	12.28404285926710	15.16094928680224
H	10.08954589976352	11.78891084441327	16.53499387031031
H	11.45228286343463	12.90712418476584	16.78422556343099
C	10.51049450261623	14.99933188985074	15.24162634129672
H	9.86187090629987	15.86762343066237	15.05659204300772
H	10.96910313825450	14.71216664504063	14.28547349437101
H	11.31795218386484	15.31261832292396	15.91938409814792
C	-2.20892617551352	16.69495794008427	11.12093328628748
C	-1.96153183374170	17.76451831089681	12.20525537513912
H	-2.04936137598992	17.32664259258827	13.20994494413449
H	-2.69704744216266	18.57755183291163	12.11985477576818
H	-0.96020632067548	18.20883699339445	12.11792424073687
C	-3.64909138411746	16.17883894752253	11.26660872936889
H	-3.88309231930002	15.41041663995344	10.51598928134810
H	-4.35543209060571	17.00813332749910	11.12313893122660
H	-3.83309678121357	15.75761671030813	12.26527015794899
C	9.15740315637079	14.29466736812657	17.22015419951885
H	8.58521885313407	13.49845913274411	17.71771525980957
H	8.51177646018654	15.17931278320856	17.12382916549500
H	9.99129666213457	14.56528985582482	17.88227895821723
H	2.67610570295800	14.87670522385865	17.58529815144029
H	0.50508837670361	14.72170314155588	16.16955723481191
H	-0.50783767610929	13.28001479085355	16.26630157018899
C	-1.98518961475083	7.36148520892166	13.64339637002406
H	-1.44699197557373	6.87072118339072	12.81981314133702
H	-2.84066185432197	6.72347848903994	13.90454263316964
H	-1.32158176766848	7.39956605891176	14.51926025375993
C	-3.44278939388686	8.60862015671707	12.04551231854602
H	-3.86455058675482	9.57513809152001	11.73760667716192
H	-4.28143687946228	7.94579164035974	12.30372819096683
H	-2.92030933341625	8.17754890022290	11.17958464283040
C	-3.25106419540777	9.35903385917382	14.44314614955299
H	-2.58418433291731	9.48489514749401	15.30839747745318
H	-4.07846205018026	8.69887706840051	14.74146788491392
H	-3.67904441862587	10.34159775035458	14.20180543431355
C	-2.06286257635133	17.33078644183026	9.72329042438809
H	-1.06504409772678	17.76448500468325	9.57102183945742
H	-2.79780162873831	18.13808680780105	9.59205424602934
H	-2.23157147922101	16.58434945153007	8.93399060657339
C	1.29511128950834	13.19770864567882	17.48140060957378
H	1.35069024168164	12.09892816668113	17.53917347596458
H	0.78759430929669	13.54339669729045	18.39159254452590
C	9.49664010604243	9.38897576520213	7.84222452839595
H	9.55835565273209	8.76205881801602	8.74349419019227
H	10.25774709268113	9.03938777557180	7.12976032820725
H	8.51094238468532	9.22694295377569	7.38437155931294
C	9.64018208011690	11.72830309162266	6.91220783247667
H	8.66333851388714	11.63032982421943	6.41879969266958
H	10.40942725256327	11.41704006722592	6.19081186590089
H	9.79786954116113	12.79175397143065	7.14253815989019
C	11.14198819481591	11.02061593930308	8.77410404676280
H	11.37061821017344	12.06541827216552	9.02861403509451
H	11.88246032397619	10.69188458675155	8.03201996347022
H	11.28015034888710	10.40464201095746	9.67410622467076

14. References

[1] M. Moser, B. Wucher, D. Kunz, F. Rominger, *Organometallics* **2007**, *26*, 1024.

[2] P. Pinter, C. M. Schüßlbauer, F. A. Watt, N. Dickmann, R. Herbst-Irmer, B. Morgenstern, A. Grünwald, T. Ullrich, M. Zimmer, S. Hohloch et al., *Chem. Sci.* **2021**, *12*, 7401.

[3] B. Wittwer, N. Dickmann, S. Berg, D. Leitner, L. Tesi, D. Hunger, R. Gratzl, J. van Slageren, N. I. Neuman, D. Munz et al., *Chem. Commun.* **2022**, *58*, 6096.

[4] N. Sinha, J.-R. Jiménez, B. Pfund, A. Prescimone, C. Piguet, O. S. Wenger, *Angew. Chem. Int. Ed.* **2021**, *60*, 23722.

[5] H. E. Gottlieb, V. Kotlyar, A. Nudelman, *J. Org. Chem.* **1997**, *62*, 7512.

[6] S. Stoll, A. Schweiger, *J. Mag. Res.* **2006**, *178*, 42.

[7] G. M. Sheldrick, *Acta Cryst A* **2015**, *71*, 3.

[8] O. V. Dolomanov, L. J. Bourhis, R. J. Gildea, J. A. K. Howard, H. Puschmann, *J Appl Crystallogr* **2009**, *42*, 339.

[9] G. M. Sheldrick, *Acta Cryst. C* **2015**, *71*, 3.

[10] A. L. Spek, *Acta Crysta. C* **2015**, *71*, 9.

[11] D. Rehm, A. Weller, *Isr. J. Chem.* **1970**, *8*, 259.

[12] D. P. Hari, P. Schroll, B. König, *J. Am. Chem. Soc.* **2012**, *134*, 2958.

[13] A. Ripak, S. de Kreijger, R. N. Sampaio, C. A. Vincent, É. Cauët, I. Jabin, U. K. Tambar, B. Elias, L. Troian-Gautier, *Chem. Catal.* **2023**, *3*.

[14] J. Yu, L. Zhang, G. Yan, *Adv. Synth. Catal.* **2012**, *354*, 2625.

[15] F. Neese, *WIREs Comput Mol Sci* **2012**, *2*, 73.

[16] F. Neese, *WIREs Comput Mol Sci* **2018**, *8*.

[17] S. Grimme, A. Hansen, S. Ehlert, J.-M. Mewes, *J. Chem. Phys.* **2021**, *154*, 64103.

[18] C. van Wüllen, *J. Chem. Phys.* **1998**, *109*, 392.

[19] C. Adamo, V. Barone, *J. Chem. Phys.* **1999**, *110*, 6158.

[20] E. Caldeweyher, S. Ehlert, A. Hansen, H. Neugebauer, S. Spicher, C. Bannwarth, S. Grimme, *J. Chem. Phys.* **2019**, *150*, 154122.

[21] F. Weigend, R. Ahlrichs, *Phys. Chem. Chem. Phys.* **2005**, *7*, 3297.

[22] J.-D. Chai, M. Head-Gordon, *Phys. Chem. Chem. Phys.* **2008**, *10*, 6615.

[23] T. Yanai, D. P. Tew, N. C. Handy, *Chem. Phys. Lett.* **2004**, *393*, 51.

[24] F. Neese, F. Wennmohs, A. Hansen, U. Becker, *Chem. Phys.* **2009**, *356*, 98.

[25] R. Izsák, F. Neese, *J. Chem. Phys.* **2011**, *135*, 144105.

[26] F. Weigend, *Phys. Chem. Chem. Phys.* **2006**, *8*, 1057.

[27] D. A. Pantazis, X.-Y. Chen, C. R. Landis, F. Neese, *J. Chem. Theory. Comput.* **2008**, *4*, 908.

[28] G. L. Stoychev, A. A. Auer, F. Neese, *J. Chem. Theory. Comput.* **2017**, *13*, 554.

[29] G. Knizia, *J. Chem. Theory. Comput.* **2013**, *9*, 4834.

[30] V. N. Staroverov, G. E. Scuseria, J. Tao, J. P. Perdew, *J. Chem. Phys.* **2003**, *119*, 12129.

[31] V. Barone, M. Cossi, *J. Phys. Chem. A* **1998**, *102*, 1995.

[32] C. Angeli, R. Cimiraglia, S. Evangelisti, T. Leininger, J.-P. Malrieu, *J. Chem. Phys.* **2001**, *114*, 10252.

[33] S. K. Singh, J. Eng, M. Atanasov, F. Neese, *Coord. Chem. Rev.* **2017**, *344*, 2.

[34] Chemcraft - graphical software for visualization of quantum chemistry computations. Version 1.8, build 654. https://www.chemcraftprog.com.

[35] M. D. Hanwell, D. E. Curtis, D. C. Lonie, T. Vandermeersch, E. Zurek, G. R. Hutchison, *J. Cheminform.* **2012**, *4*, 17.

15. Author Contributions

Synthesis was carried out by PY, BW and AS. PY conducted spectroscopic investigations and analysis, photocatalysis experiments. Electrochemical investigations were carried out by PY and FRN. EPR spectroscopic investigations and simulations were carried out by DL. Theoretical investigations were performed by DM. Crystal structure solution and analysis was performed by FT and SH. The idea was conceived by SH and OSW. The manuscript was written by PY, BW, DM, OSW and SH and proof read by all authors.
